# Cupin‐Type Dimethylsulfoniopropionate Lyase from *Pelagibacter ubique* (DddK*_Pu_*) Catalyzes Aza‐Michael Addition of Primary and Secondary Amines to Acrylic Acid

**DOI:** 10.1002/anie.202505934

**Published:** 2025-09-14

**Authors:** Diletta Arceri, Angela Mourelle, Teodor Parella, Jordi Bujons, Carlos J. Moreno, Pere Clapés

**Affiliations:** ^1^ Department of Biological Chemistry Institute for Advanced Chemistry of Catalonia (IQAC‐CSIC) Jordi Girona 18–26 Barcelona 08034 Spain; ^2^ Servei de Ressonància Magnètica Nuclear Universitat Autònoma de Barcelona Campus Bellaterra Bellaterra Spain

**Keywords:** Acrylate, Aza‐Michael addition, Biocatalysis, Dimethylsulfoniopropionate lyase

## Abstract

The formation of carbon─nitrogen (C─N) bonds is a cornerstone of organic synthesis, underpinning the production of amines, imines, and nitriles found in numerous active ingredients. Among the methods for C─N bond formation, the aza‐Michael addition stands out as a powerful and versatile approach. Herein, we present a biocatalytic strategy for the efficient aza‐Michael addition of primary and secondary amines to acrylic acid, i.e., aza‐Michaelase activity, leveraging the promiscuity of dimethylsulfoniopropionate (DMSP) lyase from *Pelagibacter ubique* HTCC1062 (DddK*_Pu_*). In vivo DddK*_Pu_* catalyzes the β‐elimination of DMSP to sodium acrylate and dimethylsulfide (i.e., a retro sulfa‐Michael reaction). Here, we screened DddK*_Pu_* against a diverse library of 30 primary and 44 secondary amines. The wild‐type enzyme achieved 90%–100% conversion and 40%–86% isolated yields of *N*,*N*‐disubstituted‐β‐amino acids with secondary amines. For primary amines, the W26G variant proved optimal, furnishing 50%–100% conversion and 43%–81% isolated yields of *N*‐substituted‐β‐amino acids. Notably, the enzyme exhibited remarkable chemoselectivity: for pyrrolidin‐2‐ylmethanamine, the reaction occurred exclusively at the secondary amine, while for piperidin‐2‐ylmethanamine, it reacted selectively at the primary amine. These findings highlight DddK*_Pu_* as a versatile biocatalyst for the selective synthesis of β‐amino acids, expanding the toolbox for C─N bond formation.

## Graphical abstract

A dimethylsulfoniopropionate lyase (DddK) was identified as an efficient aza‐Michaelase for the addition of amines to acrylate. Screening 74 amines with wild‐type DddK and its W26G variant yielded *N*‐substituted and *N*,*N*‐disubstituted β‐amino acids in 30%–93% isolated yield. This versatile method enables access to novel β‐amino acids, which serve as valuable building blocks for pharmaceutical synthesis.

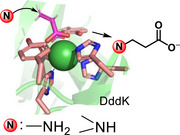

## Introduction

The formation of C─N bonds is an important chemical transformation in the preparation of amines, amino alcohols, amino acids, N‐heterocycles, nucleic acids, and materials science.^[^ ^1^, ^2^, ^3^, ^4^, ^5^, ^6^, ^7^, ^8^, ^9^, ^10^, ^11^, ^12^, ^13^, ^14^, ^15^, ^16^ ^]^ These compounds are used extensively as chiral building blocks in the pharmaceutical and agrochemical industries.^[^ ^17^, ^18^, ^19^ ^]^ In addition, the presence of chiral amines in active pharmaceutical ingredients is estimated to be around 40%, and this percentage is even higher if only the amino groups, chiral and achiral, are considered.^[^ ^20^, ^21^ ^]^

**Scheme 1:**
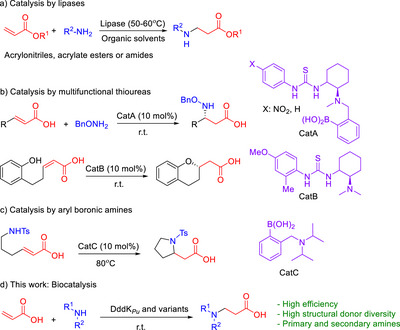
Strategies for the aza‐Michael reaction using acrylic acid and derivatives: a) catalysis by lipases;^[^ ^34^, ^35^, ^36^, ^37^, ^38^, ^39^, ^40^ ^]^ b) catalysis with multifunctional thioureas;^[^ ^41^, ^42^, ^43^, ^44^ ^]^ c) catalysis with arylboronic amines;^[^ ^41^, ^45^ ^]^ and d) this work: enzymatic catalysis using dimethylsulfoniopropionate (DMSP) lyase from *Pelagibacter ubique* HTCC1062 (DddK*_Pu_*) and variants thereof.

Apart from the chemical^[^ ^22^, ^23^ ^]^ and biocatalytic reductive amination^[^ ^24^, ^25^ ^]^ (i.e., iminoreductases, aminoreductases, and transaminases), ammonia lyases and aminomutases,^[^ ^14^, ^26^, ^27^, ^28^, ^29^ ^]^ the aza‐Michael reaction offers an interesting alternative to C─N bond synthesis. The aza‐Michael is used in inter‐ or intramolecular reactions for the preparation of various compounds such as β‐amino acids, β‐amino esters, β‐lactams, and N‐heterocycles.^[^ ^30^, ^31^, ^32^, ^33^ ^]^ Michael donors are usually primary and secondary amines, *N*‐silyloxycarbamates, azoles, hydrazines, and *N*‐hydroxycarbamates. Michael acceptors include α,β‐unsaturated acids, esters, amides, and nitriles (Scheme 1), α,β‐unsaturated phosphonates and sulfones, α‐nitroolefins, ketones and aldehydes, and vinyl‐substituted heterocycles.^[^ ^31^, ^33^ ^]^

Aza‐Michael reactions with acrylic acid derivatives, i.e., α,β‐unsaturated carboxylic acids as the sole EWG, are scarce (Scheme 1d).^[^ ^41^, ^43^, ^44^, ^45^, ^46^ ^]^ Carboxylic acids are poor EWGs and have weak coordination capacity with metal Lewis acids, which makes carboxyl‐directed olefinic C─H activation challenging.^[^ ^42^, ^47^ ^]^ The most commonly used are esters and amides derivatives of acrylic acid (Scheme 1a).^[^ ^48^, ^49^, ^50^ ^]^ Biocatalytic aza‐Michael C─N bond formation has been achieved by utilizing the promiscuity of various lipases and proteases in organic media (Scheme 1a). This transformation also employs α,β‐unsaturated aldehydes, ketones, acrylonitriles, acrylate esters, or amides at elevated temperatures (typically 50–60 °C).^[^ ^34^, ^35^, ^36^, ^37^, ^38^, ^39^, ^40^, ^51^ ^]^ Lipases and proteases possess an active site (e.g., the oxyanion hole formed by Thr40 and Gln106 in Cal‐B lipase),^[^ ^51^ ^]^ which preferentially accommodates neutral or partially polarized substrates such as acrylate esters or amides. However, the deprotonated carboxyl group of acrylic acid carries a full negative charge, which cannot be adequately stabilized within the hydrophobic and electrostatically constrained environment of the active site. Consequently, acrylic acid is not a suitable substrate for these hydrolytic enzymes, necessitating the use of its ester or amide derivatives for their recognition and activation. However, challenges such as competing aminolysis versus 1,4‐addition, prolonged reaction times, and a lack of stereoselectivity remain significant limitations.^[^ ^34^, ^35^, ^36^, ^37^, ^38^, ^39^, ^40^ ^]^ Therefore, the use of acrylic acid as a Michael acceptor in biocatalysis is unprecedented.

**Figure 1:**
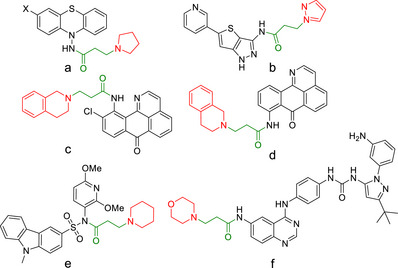
Examples of biologically active compounds incorporating *N*,*N*‐disubstituted‐β‐amino acid derivatives. For treatment of Alzheimer's disease a) *N*‐acylaminophenothiazines as neuroprotective agent;^[^ ^52^ ^]^ b) thieno[3,2‐*c*]pyrazol‐3‐amine derivatives as potent glycogen synthase kinase 3β inhibitors;^[^ ^53^ ^]^ c and d) hybrids of oxoisoaporphine–tetrahydroisoquinoline, novel multitarget inhibitors of inflammation and amyloid‐β aggregation.^[^ ^54^ ^]^ Others are e) quinazoline derivatives as inhibitors to overcome gatekeeper drug‐resistant mutations;^[^ ^55^ ^]^ and f) IMB105 with a potent in vitro antiproliferative activity against several human cancer cell lines, including drug‐resistant tumor cells.^[^ ^56^ ^]^

The compounds generated from the aza‐Michael addition of secondary amine to acrylic acid, i.e., *N*,*N*‐disubstituted‐β‐amino acids, constitute building blocks for the synthesis of important pharmacologically active molecules, e.g., for the treatment of Alzheimer's disease, such as neuroprotective agents (Figure 1a),^[^ ^52^ ^]^ glycogen synthase kinase 3β inhibitors (Figure 1b),^[^ ^53^ ^]^ and multitarget inhibitors of inflammation and amyloid‐β aggregation (Figure 1c,d).^[^ ^54^ ^]^ Other important compounds include inhibitors to overcome gatekeeper drug‐resistant mutations (Figure 1e),^[^ ^55^ ^]^ and IMB105 with potent in vitro antiproliferative activity against several human cancer cell lines, including drug‐resistant tumor cells (Figure 1f).^[^ ^56^ ^]^

**Figure 2:**
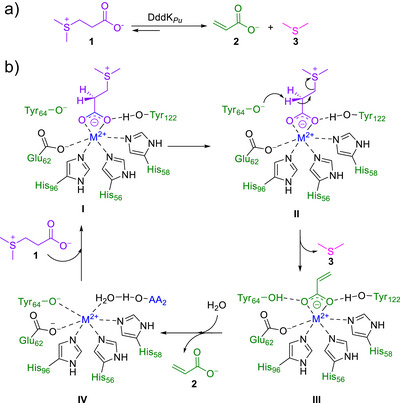
a) Natural reaction of DMSP lyase from *Pelagibacter ubique* (DddK*_Pu_*). b) Proposed reaction mechanism for the cleavage of DMSP by DddK*_Pu_*.^[^ ^58^ ^]^

**Figure 3:**
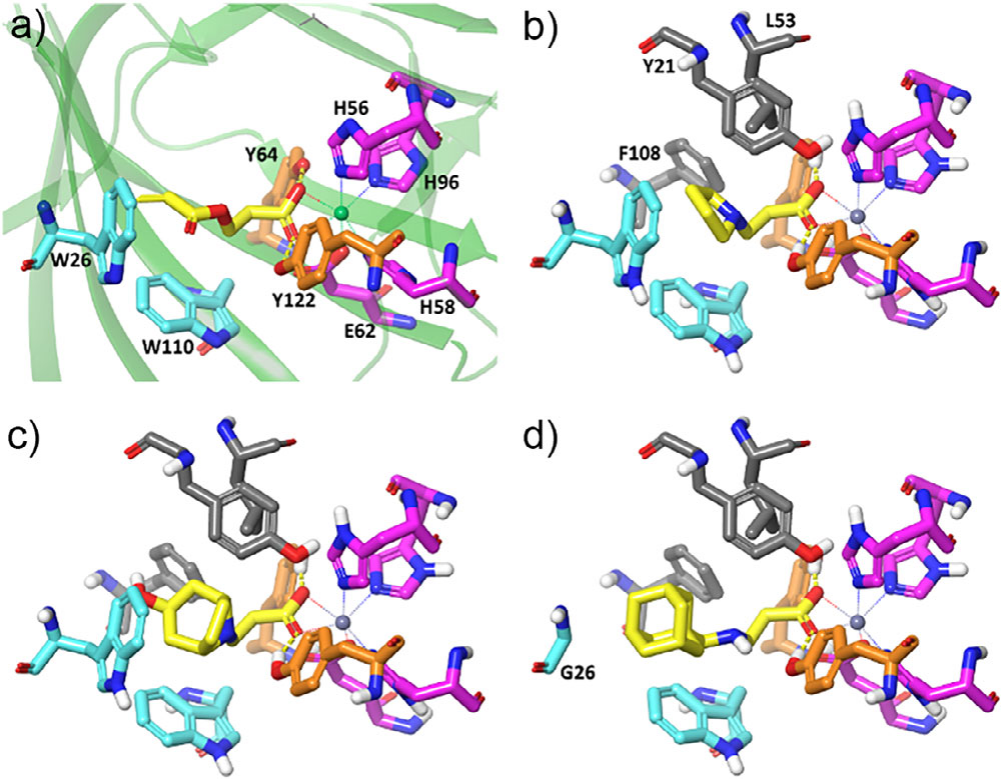
a) X‐ray structure of DMSP lyase from *Pelagibacter ubique* HTCC1062 (DddK*_Pu_*, PDB 5TFZ) showing the metal coordination site residues (magenta), diacrylate (yellow), essential tyrosines 64 and 122 (orange), and residues W26 and W110, which were mutated in this work (cyan). b and c) Lowest energy models of intermediate adducts (yellow) **7c** (b) and **7l** (c) bound into the active site of wild‐type DddK*_Pu_*. d) Lowest energy model of the intermediate adduct **5aa** bound into the active site of the W26G variant. Models were built as described in the Computational Methods section (Supporting Information) and optimized by QM/MM methods with QSite.^[^ ^60^, ^61^, ^62^ ^]^

**Scheme 2:**
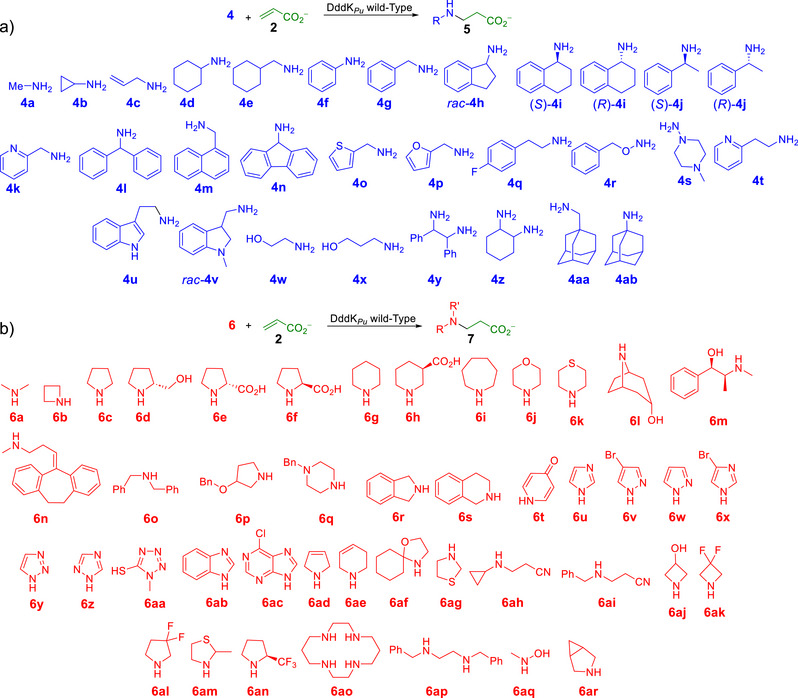
Panel of a) primary and b) secondary amines as nucleophiles for the aza‐Michael reactions catalyzed by the DddK*_Pu_* wild‐type.

In this paper, we report the promiscuous catalytic properties of dimethylsulfoniopropionate (DMSP) lyase from *Pelagibacter ubique* HTCC1062 (DddK*_Pu_*)^[^ ^57^ ^]^ for aza‐Michael reactions using acrylic acid as the Michael acceptor. In nature, DddK*_Pu_* catalyzes the cleavage of DMSP (**1**) to acrylate (**2**) and dimethylsulfide (**3**) (Figure 2a) via a β‐elimination mechanism, i.e., a retro sulfa‐Michael reaction (Figure 2b).^[^ ^57^, ^58^, ^59^ ^]^ The structure of DddK*_Pu_*, either free (PDB 6A53) or complexed to diacrylate (Figure 3a, PDB 5TFZ), has been reported.^[^ ^57^, ^58^, ^59^ ^]^ Based on these structural data and the proposed catalytic mechanism (Figure 2b), we hypothesized that DddK*_Pu_* should be able to activate acrylic acid (Figure 2b intermediate III) through coordination with the metal center. Therefore, we hypothesized that the enzyme could also catalyze Michael additions with acrylic acid as the acceptor. To test this, we evaluated DddK*_Pu_* as a catalyst for the aza‐Michael addition of primary (**4**) and secondary amines (**6**) as donor substrates to acrylate (**2**) (Scheme 2a,b).

## Results and Discussion

### Characterization of Dimethylsulfoniopropionate Lyase from *Pelagibacter ubique* HTCC1062 (DddK*_Pu_*)

**Figure 4:**
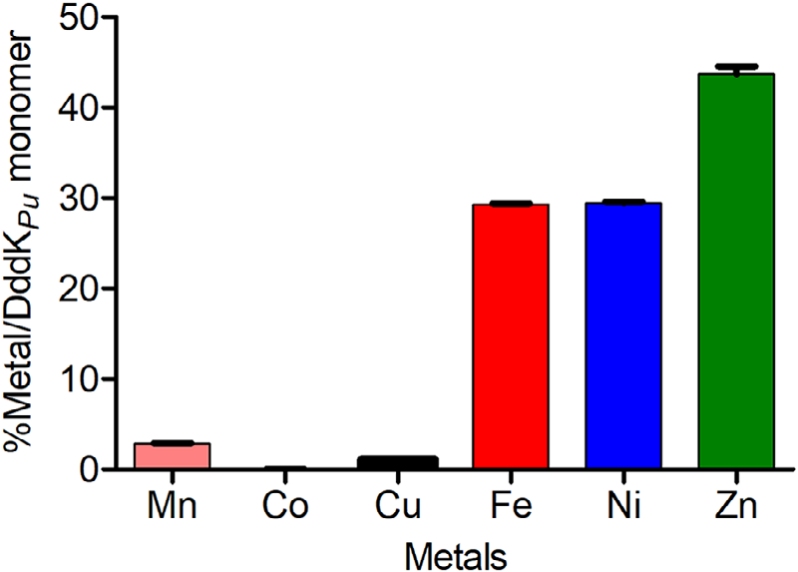
Metal content of DddK*_Pu_* wild‐type determined by ICP‐MS and ICP‐OES for Fe. Protein sample (1.0 mg mL^−1^ in 5 mM HEPES buffer containing NaCl (100 mM) and glycerol 50% (v/v) at pH 7.5) was diluted with Milli‐Q water (5.1 mL). Then, the protein was precipitated with HNO_3_ for trace metal analysis (ppb) (2% v/v, final concentration in the mixture). The solution was stored at 4 °C for 12 h and the precipitate was removed by centrifugation at 15 000 g for 30 min. The supernatant was filtered (0.2 mm, SFCA+PF membrane) and the solution was used to probe the metal content by inductively coupled plasma‐mass spectrometry (ICP‐MS) and inductively coupled plasma‐optical emission spectroscopy (ICP‐OES). Error bars are the values of the standard error of the mean of three replicate measurements.

The wild‐type DddK*_Pu_*, with a His‐tag (DddK*_Pu_*) to facilitate its purification, was obtained as previously reported in the literature.^[^ ^57^ ^]^ The metal content analysis revealed that DddK*_Pu_* contained 37% of Zn, 28% of Ni, 29% of Fe, and minor amounts of Mn (3%), Cu (1%), and Co (0.02%) (Figure 4). The analysis showed that the protein was saturated with metals (i.e., total mmols of metals equal to mmol of enzyme) and that no losses occurred during isolation and purification. This metal distribution deviated from that previously reported by Schnicker et al., which included 40% Fe, 18% Zn, 7% Ni, and 4% Mn.^[^ ^57^ ^]^ The observed discrepancy can be attributed to the utilization of different *Escherichia coli* strains (M15 versus BL21 DE3) for enzyme production, varying cell lysis strategies, or distinct storage buffers. To investigate the influence of the HisTag on the metal content and activity, a variant was constructed by replacing the three His and the Glu of the metal binding site with Ala, i.e., DddK*_Pu_* H56A/H58A/E62A/H96A variant. The metal content analysis of this variant indicated that it contained 21% metal occupancy (i.e., (total mmol of metal/mmol of enzyme) × 100), with a metal distribution of Ni (11%), Zn (7.5%), and Cu (2.6%) (see Supporting Information).

The specific activity of DddK*_Pu_* was determined by the endpoint method using 10 different enzyme concentrations, using DMSP as substrate. The production of sodium acrylate (**2**) was measured by HPLC as described in the literature (see Supporting Information) (activity: 12.2 ± 0.4 U mg^−1^, where U is defined as the amount of enzyme that catalyzes the conversion of 1 µmol of DMSP to acrylate per minute at 25 °C in 50 mM triethanolamine buffer pH 8.0).^[^ ^57^, ^59^ ^]^

According to Schnicker et al.,^[^ ^57^ ^]^ the activity of DddK*_Pu_* as isolated was due to the presence of Zn^2+^, Ni^2+^, and Mn^2+^. In the same study, Ni^2+^ was found to be the metal cofactor that gave the best activity to DddK*_Pu_*. In our hands the enzymatic activity remained unaffected when Ni^2+^ (10 equiv) was added to the reaction, which suggests that no metal exchange probably occurred. Therefore, the activity that we observed was due mainly to the Zn^2+^ and Ni^2+^ present in the samples. In addition, the enzymatic activity was not affected by the addition of EDTA (100 mM), indicating the strong affinity of the protein for the metals under the reaction conditions. Importantly, no activity was detected for the DddK*_Pu_* H56A/H58A/E62A/H96A variant. Moreover, the addition of Ni^2+^ (10 equiv) to the reaction also resulted in no detectable activity. This suggests that for the natural substrate, the reaction occurs exclusively in the active site of the DddK*_Pu_* and that the His‐tag had no effect on the enzymatic catalysis.

### DddK*_Pu_* as Aza‐Michael Biocatalyst

**Figure 5:**
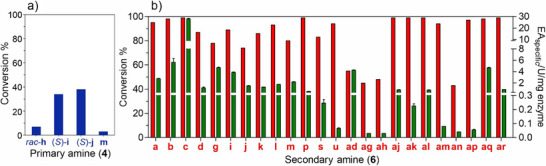
Conversions (24 h) and DddK*_Pu_* wild‐type aza‐Michaelase activity of the reactions of primary amines (**4**) a) only conversions and secondary amines (**6**) b) conversion (red bars) and activity (green bars) based on consumption of **2**. Assay mixture (0.5 mL) containing 50 mM TEA buffer pH 8.0, acrylate (**2**) (0.1 M), and Michael donor (**6** or **4**) (0.1 M). End‐point activity assays using 10 different enzyme concentrations after 15 min of reaction except **6ak**: measured after 30 min, **6ad**, **6am**, **6ap**: measured after 2 h, **6u**: measured after 3 h, **6ag**, **6ah**, and **6an**: measured after 24 h. One unit U of activity was defined as the amount of DddK*_Pu_* wild‐type, which catalyzes the consumption of 1 µmol of **2** per min at 25 °C in 50 mM triethanolamine buffer pH 8.0. Error bars are the values of the standard error of the mean of three independent experiments under identical reaction conditions.

We initiated our study to establish whether DddK*_Pu_* wild‐type could catalyze the aza‐Michael reaction using pyrrolidine (**6c**) as donor substrate under the established screening conditions (see Supporting Information), i.e., equimolar concentrations of sodium acrylate (**2**) and **6c** (100 mM) in 50 mM triethanolamine buffer pH 8.0, enzyme 2 mg mL^−1^ (0.13 mol%) and 0.5 mL of reaction volume. To our delight, DddK*_Pu_* indeed catalyzed the aza‐Michael reaction with quantitative conversions as determined by the consumption of **2**. We continued our study by evaluating the substrate scope of the DddK*_Pu_* wild‐type with a panel of primary (**4**) and secondary amines (**6**) as aza‐Michael donors (Scheme 2a,b). Indeed, DddK*_Pu_* wild‐type catalyzed the reactions, with the secondary amines being better accepted substrates than the primary amines (Figure 5a,b). Conventional chemical methods that use acrylate derivatives with electron‐withdrawing groups (EWGs) exhibit a consistent trend: cyclic secondary amines, such as pyrrolidine, piperidine, morpholine, and monosubstituted piperazine, are superior donors compared to their acyclic counterparts, while primary amines are less nucleophilic and exhibit poor donor capabilities.^[^ ^32^, ^63^ ^]^ DddK*_Pu_* catalysis resulted in conversions of ≥90% within 24 h for secondary amines such as the acyclic: dimethylamine (**6a**) and *N*‐methylhydroxylamine (**6aq**), cyclic: azetidine (**6b**), pyrrolidine (**6c**), tropine (**6l**), 3‐(benzyloxy)pyrrolidine (**6p**), azetidin‐3‐ol (**6aj**), 3,3‐difluoroazetidine (**6ak**), 3,3‐difluoropyrrolidine (**6al**), 2‐methylthiazolidine (**6am**), *N* ^1^,*N* ^2^‐dibenzylethane‐1,2‐diamine (**6ap**), and 3‐azabicyclo[3.1.0]hexane (**6ar**), and heterocyclic imidazole (**6u**). Acyclic: (1*R*,2*R*)‐(−)‐pseudoephedrine (**6m**), and cyclic: (*R*)‐pyrrolidin‐2‐ylmethanol (**6d**), piperidine (**6g**), azepane (**6i**), morpholine (**6j**), thiomorpholine (**6k**), tetrahydroisoquinoline (**6s**) were all converted in ≥74%. Acyclic: 3‐(cyclopropylamino)propanenitrile (**6ah**), cyclic: thiazolidine (**6ag**), (*S*)‐2‐(trifluoromethyl)pyrrolidine (**6an**), and heterocyclic: 2,5‐dihydro‐1*H*‐pyrrole (**6ad**), gave ≥40% conversion (Figure 5b). No consumption of acrylate was detected for the rest of secondary amines, including l‐ and d‐proline (**6e** and **6f**), (*R*)‐piperidine‐3‐carboxylic acid (**6h**), isoindoline (**6r**), pyridin‐4(1*H*)‐one (**6t**), pyrazoles, bromo imidazole (**6x**), triazoles (**6y** and **6z**), tetrazoles (**6aa**), 1*H*‐benzo[*d*]imidazole (**6ab**), and 6‐chloro‐9*H*‐purine (**6ac**).

Reactions with primary amines are less efficient, as observed with chemical methods.^[^ ^32^ ^]^ DddK*_Pu_* catalyzed the reaction using (*S*)‐1,2,3,4‐tetrahydronaphthalen‐1‐amine, ((*S*)‐**4i**), and (*S*)‐1‐phenylethan‐1‐amine, ((*S*)‐**4j**), resulting in a modest 34% and 38% conversion, respectively (Figure 5a). In control experiments performed with the same reaction components but without the addition of DddK*_Pu_*, no product was formed. Furthermore, the DddK*_Pu_* H56A/H58A/E62A/H96A variant did not catalyze the aza‐Michael addition of **6c**, the most active secondary amine to acrylate. This finding substantiates the conclusion that catalysis occurs exclusively within the active site of DddK*_Pu_*. Molecular docking was performed on the acrylate adducts (**7**) of selected secondary amines using the structure of diacrylate‐bound wild‐type DddK_*Pu*_ as target (PDB 5TFZ) (Figure S7). These modeled docked adducts are transient intermediates of the enzymatic reaction, which can provide clues about how the precursors (**6**) interact with and fit into the enzyme's active site cavity. These models suggest that the active site cavity of DddK*_Pu_* can accommodate relatively large and rigid substrates, such as **6l**, in addition to smaller and more reactive ones (e.g., **6c**) (Figures 3b,c and S7F–L). For bigger amines such as **6m** or **6p**, the docking results (Figure S7J–L) suggest that part of the substrate cannot be fully allocated inside the catalytic site and that it reaches the surface of the enzyme through a narrow hole delimited by Tyr122, which acts as a gate between open and closed enzyme forms.^[^ ^57^ ^]^ However, this possibility cannot be extended to the largest amines assayed, like **6n**, **6o**, or **6ao**, which are inactive. The apparent contrast between high conversions for some substrates (e.g., **6c** and **6g**) and no reactivity for structurally similar analogues (e.g., **6e**/**6f** or **6h**) can be explained considering that the inactive amines contain a carboxylate group that can compete with acrylate for binding into the metal site (Figure S7, panels E–G). In other cases, different esteroelectronic effects must be invoked to explain the observed results.

### Activity of DddK*_Pu_* Toward Secondary Amines

The specific activity of DddK*_Pu_* for the secondary amine substrates was measured in independent experiments (Figure 5b). Pyrrolidine (**6c**) was by far the most active secondary amine (28.0 U mg^−1^). This was followed by substrates with activities between 2.8 U mg^−1^ and 5.7 U mg^−1^, such as acyclic: **6a**, **6aq**, cyclic: **6b**, **6g**, and **6i**; heterocyclic: **6ad**; between 0.51 U mg^−1^ and 2.2 U mg^−1^ for acyclic: **6m**, and cyclic: **6d**, **6j**, **6k**, **6l**, **6p**, **6aj**, **6al**, and **6ar**. The lowest activities, ranging from 0.03 U mg^−1^ to 0.25 U mg^−1^, were found for acyclic: **6ah**, **6ap**, cyclic: **6s**, **6ag**, **6ak**, **6am**, and **6an**, and heterocyclic: **6u**. Notably, although the activity for **6u**, **6ak**, **6am**, and **6ap** is very low, the conversions were >90% after 24 h.

### Aza‐Michael Addition of Primary Amines to Sodium Acrylate Catalyzed by DddK*_Pu_* Variants

The low substrate tolerance exhibited by the DddK*_Pu_* wild‐type toward primary amines prompted us to identify potential beneficial mutations on its active site through a structure‐guided approach. We hypothesized that W26 and W110 might restrict an optimal binding approach of the more flexible primary amines to the activated enzyme‐acrylate acceptor (Figure 3a). Therefore, both residues were independently substituted with either G, A, V, M, or F.

**Figure 6:**
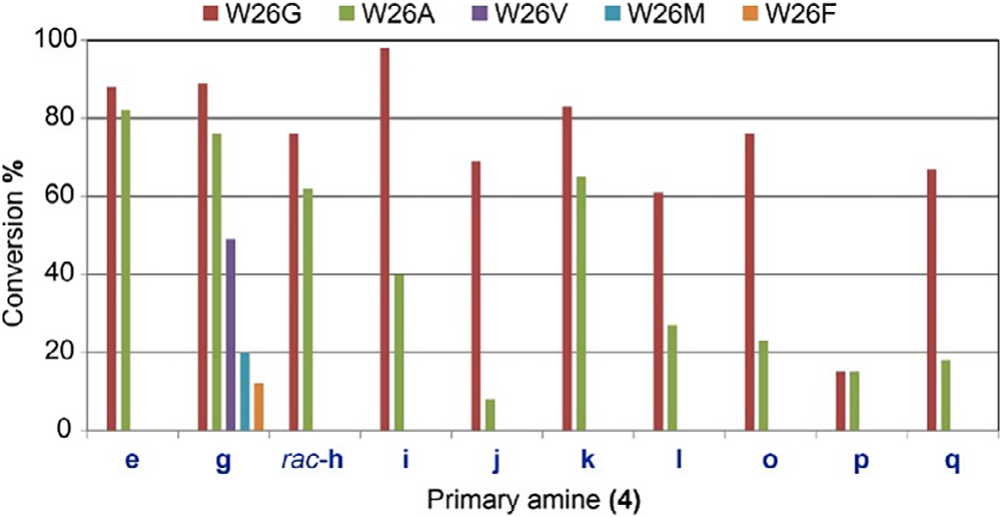
Conversions of the aza‐Michael addition of primary amines (**4**) to acrylate (**2**) catalyzed by DddK*_Pu_* W26X variants (X = G, A, V, M, F). Conversions were measured after 24 h based on the consumption of **2**. Assay mixture (0.5 mL) contained 50 mM TEA buffer pH 8.0, NiCl_2_ (1 mM), acrylate (**2**) (0.1 M), and Michael donor (**4**) (0.1 M).

The DddK*_Pu_* variant W26G was tested in the aza‐Michael addition of benzylamine (**4g**) to **2**, resulting in a 99% conversion (Figure 6, see **4g**). In contrast, no product was detected with either the W110A and W110V variants or the wild‐type. Substitution of W26 with G, A, V, M, and F showed that the conversion increased as the size of the amino acid decreased in the order G>A>V>M>F, with W26G rendering the best results (Figure 6 for **4g**). This suggests that bulky amino acid residues in this position are detrimental to the efficacy of the variant.

**Figure 7:**
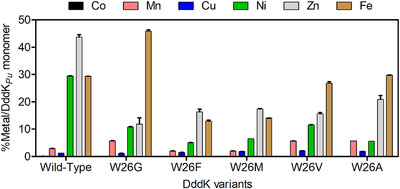
Metal content of DddK*_Pu_* wild‐type and W26X variants (where X = G, A, V, M, F) determined by ICP‐MS and ICP‐OES for Fe. Metal occupancy was 98% for wild‐type, 75% for W26G, 64% for W26A, 62% for W26V, 42% for W26M, and 38% for W26F. Error bars are the values of the standard error of the mean of three replicate measurements.

Substitution of W26 with G, A, V, M, or F was found to cause a variation in their metal content and distribution. Indeed, the metal occupancy after purification was 80% for W26G, 69% for W26A, 66% for W26V, 46% for W26M, and 41% for W26F. The Fe content was found to be the most abundant in W26G (46%), W26A (30%), and W26V (27%). Additionally, the Ni and Zn levels were lower than those in the wild‐type: 5.3–12.8 versus 29.4 for Ni and 14.3–26.7 versus 43.7 for Zn (Figure 7). Consequently, the reactions with these variants were performed with the addition of Ni^2+^ (10 equiv) in the reaction. In the case of W26G, its activity using the natural substrate DMSP was found to be 50‐fold lower than that of the wild‐type, i.e., 12.0 ± 0.7 versus 0.23 ± 0.01, and increased threefold with the addition of Ni^2+^, i.e., 0.23 ± 0.01 versus 0.80 ± 0.02 (see Supporting Information).

**Figure 8:**
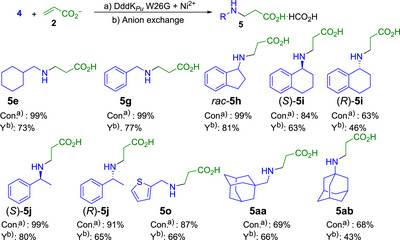
Preparative scale synthesis of *N*‐substituted‐β‐amino acid derivatives after W26G DddK*_Pu_* variant‐catalyzed aza‐Michael addition of primary amines **4** to **2**. ^a)^Conversion of acrylate to aza‐adduct. ^b)^Isolated yield.

**Figure 9:**
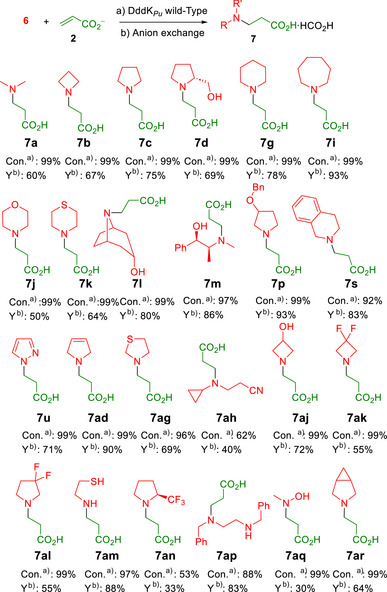
Preparative scale synthesis of *N*,*N*‐disubstituted‐β‐amino acid derivatives after wild‐type DddK*_Pu_*‐catalyzed aza‐Michael addition of secondary amines **6** to **2**. ^a)^Conversion of acrylate to aza‐adduct. ^b)^Isolated yield.

Screening of DddK*_Pu_* W26X variants against the selected primary amines (**4**) demonstrated that DddK*_Pu_* W26G variant was indeed the most effective in all cases (Figure 6). The primary amines **4e**, **4g**‐**k**, **4m**, **4n**, **4o**, and **4aa**‐**ab** were all converted. Benzylamine (**4g**) and its analogues, *rac*‐**4h** and **4i**‐**l**, including the nonaromatic cyclohexylmethanamine (**4e**), were converted in the range of 53%–99%. In addition, (*S*)‐**4i** and (*S*)‐**4j** gave better yields than their *R*‐counterparts suggesting some degree of enantiomeric discrimination. Highly sterically demanding amines such as ((3*r*,5*r*,7*r*)‐adamantan‐1‐yl)methanamine (**4aa**) and (3*s*,5*s*,7*s*)‐adamantan‐1‐amine (**4ab**) were also converted (68%–99%). Despite its large size, molecular docking showed that these can still fit in the larger cavity generated by the W26G mutation, as shown in Figure 3d for the adduct (**5aa**), derived from amine **4aa**. Methylamine moieties attached to heteroaromatic rings, **4k** and **4o** were converted (40%–88%) with the exception of furan‐2‐ylmethanamine (**4p**). The low molecular weight amines (**4a**‐**c**, **4w**‐**x**), diamines (**4y**, **4z**) and ethanamine moieties connected to aryl groups (**4q**, **4t**, **4u**, *rac‐* **4v**) were not substrates. The DddK*_Pu_* W26G and W26A variants were screened against secondary amines **6**. They showed similar conversions for amines tolerated by the wild‐type, while both remained inactive for those with no detected acrylate consumption. Thus, neither W26G nor W26A improved the performance toward secondary amines. The products were identified as formate salts of *N*‐substituted, primary amine‐derived (Figure 8), and *N*,*N*‐disubstituted‐β‐amino acid derivatives from secondary amines (Figure 9). It was noteworthy that the symmetric secondary diamine **6ap** yielded only the monosubstituted derivative even when 2 equiv of the acrylate acceptor were used.

### Mechanism of the Aza‐Michael Reactions

To date, different DddK*_Pu_* structures have been reported, among them the free enzyme (PDB 5TG0, with partial occupancy by Fe and Zn in the metal binding site) and a diacrylate‐bound form of the wild‐type enzyme (PDB 5TFZ, with Ni in the metal binding site).^[^ ^57^ ^]^ Despite both structures being quite similar (RMSD 1.1 Å), some differences were detected that allowed to get insight into the enzyme mechanism. In the free enzyme, unprotonated Tyr64 was coordinated to the iron, together with His58, Glu62 (bidentate), His96, and a water molecule, all of them at distances between 2.0 and 2.7 Å, resulting in a six‐coordinate metal site with octahedral geometry. In the ligand‐bound form, the ligands coordinated to Ni are His56, His58, Glu62 (monodentate), His96 and the carboxylate group of diacrylate (bidentate), all at 2.1–2.7 Å from the metal, resulting also in a six‐coordinate metal center with octahedral geometry (Figure 3a). The carboxylate group of diacrylate is also hydrogen bound to Tyr122 and it is, as well, at hydrogen bond distance of Tyr64 (O─O distance = 2.34 Å), therefore, both Tyr residues help anchor the carboxylate of diacrylate to the metal center. In addition, Tyr122 has been found to have two different dispositions in the free and ligand‐bound forms that determine an “open” and a “closed” form of the enzyme.^[^ ^57^ ^]^ Based on all the structural data, it was proposed that in the resting enzyme, the unprotonated Tyr64 and a water molecule coordinate the active site metal and that both ligands are displaced when the carboxylate‐containing DMSP natural substrate enters into the active site and binds to the metal in a bidentate form. In this arrangement, the tyrosinate oxygen atom is well‐poised for proton abstraction, promoting the cleavage of DMSP and constituting the essential catalytic base. In contrast, Wang and coworkers carried out a detailed theoretical study of the DddK_Pu_ mechanism^[^ ^58^ ^]^ where they proposed that for the reaction to take place, the carboxylate of DMSP should bind in monodentate form to the metal, together with the unprotonated Tyr64, such that this tyrosinate is close to the α‐methylene group of DMSP, from where it will abstract the proton that triggers the cleavage of DMSP. Furthermore, in a paper from Peng et al.^[^ ^59^ ^]^ the participation of a water molecule that acts as proton relay with Tyr64, allowing it to deprotonate, has been proposed. In the same paper, the authors also provide evidence that Tyr64 is not the only residue that may act as catalytic base since the Y64F mutant retains about 10% of enzyme activity, and Tyr122 is proposed as the compensating catalytic base. Indeed, in our experience, different mutants of Tyr64 and Tyr122 exhibit activity catalyzing the aza‐Michael addition of a variety of amines to acrylate, with efficiencies that sometimes are higher for the Tyr64 mutant (where residue 64 is unable to promote proton transfer) than for the corresponding Tyr122, depending on the amine substrate (results not shown). All of these indicate that DddK*_Pu_* activity and substrate tolerance depend on a number of factors and that the results might be difficult to predict.

**Figure 10:**
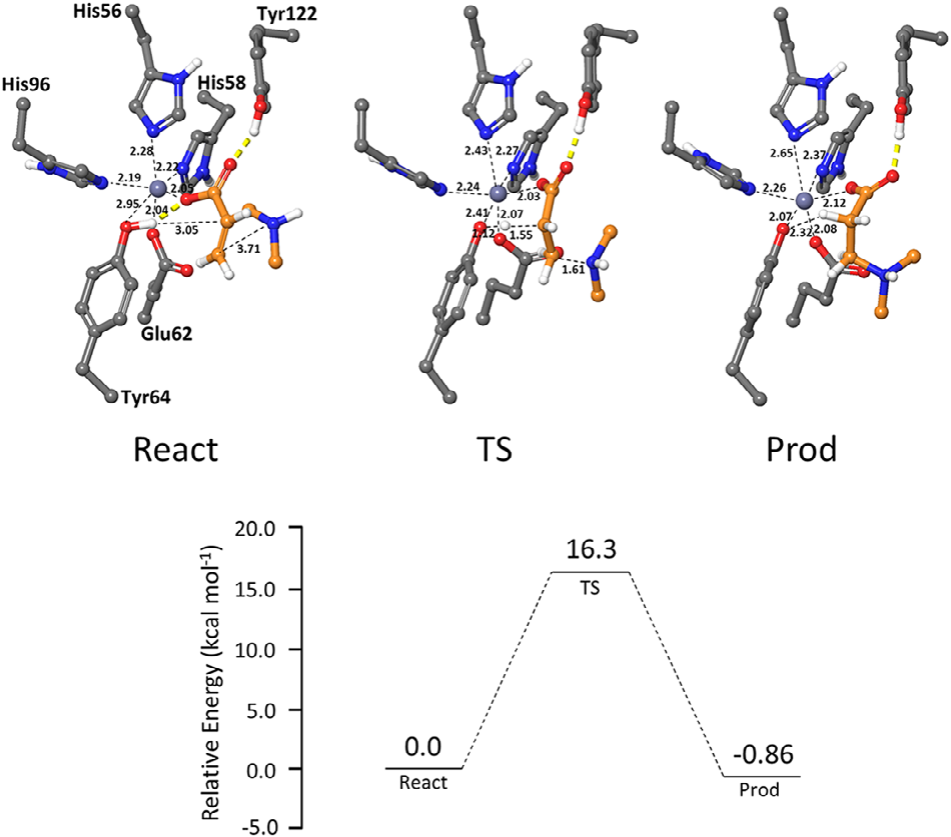
Optimized structures of stationary points in the Zn(II)‐DddK*_Pu_*‐catalyzed aza‐Michael addition of amine **6a** to **2** and the corresponding energy profile. The energies have been corrected for solvation, zero‐point vibrational, and dispersion effects.

For the aza‐Michael reactions studied here, the enzymatic mechanism of DMSP cleavage should be reversed in the synthetic direction and the essential catalytic group must be an acid capable of transferring a proton to the α‐carbon of the acrylate moiety once the reacting amine has attacked the β‐electrophilic position. With the aim of testing the possibility that the same Tyr64 could also act as proton donor, we performed DFT calculations using the same methodology and coordinates reported in the paper by Wang et al.^[^ ^58^ ^]^ to study the model reaction between amine **6a** and acrylate **2** to afford adduct **7a**, catalyzed by wild‐type DddK_Pu_ containing Zn(II), the most abundant cation in our protein samples (Figure 10). Satisfactorily, the reaction profile obtained suggests that the reaction is slightly exothermic, with a predicted activation barrier of 16.3 kcal mol^−1^, similar to those determined by Wang et al. for the natural reaction. Therefore, this supports the hypothesis that a reversed mechanism to that occurring with the natural DMSP substrate, with the same essential Tyr64 acting here as acid catalyst, might be operating in our reactions. Further studies to extend the applicability of this mechanism to other amines and catalytic metals are currently ongoing in our group and will be reported in due course.

### Preparative Synthesis

**Scheme 3:**
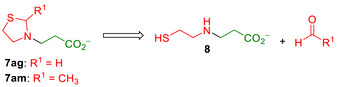
Decomposition of 3‐(thiazolidin‐3‐yl)propanoic acid (**7ag**) and 3‐(2‐methylthiazolidin‐3‐yl)propanoic acid (**7am**) to 3‐((2‐mercaptoethyl)amino)propanoic acid (**8**).

**Scheme 4:**
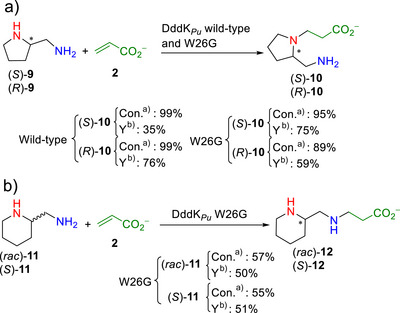
Chemoselectivity of the DddK*_Pu_* wild‐type and the W26G variant as biocatalysts of the aza‐Michael addition of a) (*S*)‐ and (*R*)‐pyrrolidin‐2‐ylmethanamine (*S*‐**9**, *R*‐**9**) and b) (*rac*)‐ and *S*)‐piperidin‐2‐ylmethanamine ((*rac*)‐**11** and (*S*)‐**11**) to **2**. ^a)^Conversion after 24 h. ^b)^Isolated yield.

Successful aza‐Michael reactions under our previously screened conditions were synthesized at a 100 mM scale in 10 mL reaction volume for product identification and characterization (Figures 8 and 9) (see Supporting Information). Reactions were generally run with 1.5 equiv of the amine donor to drive completion. However, equimolar conditions were used in instances where the concentration of dimethylformamide (DMF), introduced from amine stock solutions, exceeded 10% (v/v) in the enzymatic reaction, to mitigate potential inhibitory effects on enzyme activity. Isolated yields after purification by anion‐exchange chromatography typically ranged from 55% to 93% under nonoptimized conditions (see Supporting Information). In some cases, high substrate conversions resulted in low isolated product yields. This was probably due to the limited capacity of the column, influenced by factors such as the affinity of each compound for the ion exchange resin (e.g., **7aq**, an *N*‐methylhydroxylamine derivative, Figure 9). In addition, it may have resulted from possible side reactions of the acrylate during the reaction (i.e., formation of diacrylate)^[^ ^57^ ^]^ favored by the low activity of DddK*_Pu_* toward the amine (e.g., products **7ak**, **7al**, or **7j**, Figure 9). For both thiazolidine (**6ag**) and 2‐methylthiazolidine (**6am**), the DddK*_Pu_* catalysis exclusively formed the C─N bond. However, under the reaction conditions, the thiazolidine moiety of **7ag** and **7am** underwent partial or total decomposition yielding 3‐((2‐mercaptoethyl)amino)propanoic acid (**8**) (Scheme 3).^[^ ^64^, ^65^ ^]^ The NMR analysis revealed that the decomposition of **7ag** was 22% of the total acrylate converted, whereas that of **7am** was complete. Having established the DddK*_Pu_* wild‐type and the W26G variant as efficient aza‐Michael catalysts, we next investigated their chemoselectivity toward primary and secondary amines present in the same molecule. As model compounds we selected (*S*)‐ and (*R*)‐pyrrolidin‐2‐ylmethanamine and *rac*‐piperidin‐2‐ylmethanamine (Scheme 4).

For both enantiomers of (*S*)‐ and (*R*)‐pyrrolidin‐2‐ylmethanamine, both DddK*_Pu_* wild‐type and W26G chemoselectively catalyzed the aza‐Michael reaction of the secondary amine of the pyrrolidine to **2** in excellent conversions and yields (Scheme 4a). In contrast, (*rac*)‐piperidin‐2‐ylmethanamine ((*rac*)‐**11**) gave 57% conversion using the DddK*_Pu_* W26G variant, but it was not a substrate for the DddK*_Pu_* wild‐type (Scheme 4b). The isolated product was identified as (*rac*)‐3‐((piperidin‐2‐ylmethyl)amino)propanoic acid ((*rac*)‐**12**), indicating a high chemoselectivity of the enzyme toward the primary amine but with no enantiomeric discrimination (Scheme 4b). Using (*S*)‐piperidin‐2‐ylmethanamine (*S*)‐**11**,^[^ ^66^ ^]^ the corresponding (*S*)‐**12** gave a [α]_d_^20^ = +16.9 (*c* = 1 in MeOH).

## Conclusion

In conclusion, we demonstrate the synthetic capabilities of the DddK*_Pu_* wild‐type and the W26G variant as aza‐Michaelases for the addition of primary and secondary amines to sodium acrylate. Both enzymes show a broad substrate scope tolerating structurally diverse amine compounds. Both primary and secondary amines show high conversions, while secondary amines usually were better substrates than primary amines. Notably, the W26G variant displayed high chemoselectivity, targeting the secondary amine of pyrrolidin‐2‐ylmethanamine and the primary amine of 3‐((piperidin‐2‐ylmethyl)amino)propanoic acid. The resulting β‐amino acid derivatives are easily isolated and purified via anion exchange chromatography. This methodology is expected to be widely applicable to a variety of amines and will provide access to novel *N*‐substituted and *N*,*N*‐disubstituted β‐amino acid derivatives that can be used as building blocks for the synthesis of pharmaceutically active ingredients. DMSP lyases belong to the cupin superfamily, one of the most functionally diverse groups of metalloproteins.^[^ ^67^ ^]^ This study shows that they can activate acrylic acid derivatives via metal coordination, enabling key transformations such as Michael additions, MBH reactions, and Diels–Alder cycloadditions. These findings highlight the broader potential of cupin enzymes as versatile biocatalysts in synthetic chemistry.

## Supporting Information

The authors have cited additional references within the Supporting Information.^[^ ^57^, ^66^, ^68^, ^69^, ^70^ ^]^

## Conflict of Interests

The authors declare no conflict of interest.

## Supporting information



Supporting Information

## Data Availability

The data that support the findings of this study are available in the Supporting Information of this article.
