# Supplementary material for: Cupin‐Type Dimethylsulfoniopropionate Lyase from Pelagibacter ubique (DddK Pu ) Catalyzes Aza‐Michael Addition of Primary and Secondary Amines to Acrylic Acid
Source: Angew Chem Int Ed Engl. 2025 Sep 14;64(45):e202505934. doi: 10.1002/anie.202505934 (PMC12582016; doi:10.1002/anie.202505934)
Supplement: Supplementary file 1 — Supporting Information [file ANIE-64-e202505934-s001.pdf]

Supporting Information

Cupin-Type Dimethylsulfoniopropionate Lyase from *Pelagibacter Ubique* (Dddk$_{pu}$) Catalyzes Aza-Michael Addition of Primary and Secondary Amines to Acrylic Acid

Diletta Arceri[a], Angela Mourelle[a], Teodor Parella[b], Jordi Bujons[a], Carlos J. Moreno[a], and Pere Clapés[a]

[a]Institute for Advanced Chemistry of Catalonia, Dept. of Biological Chemistry, IQAC-CSIC, Jordi Girona 18-26, 08034 Barcelona, Spain.

[b]Servei de Ressonància Magnètica Nuclear. Universitat Autònoma de Barcelona, Bellaterra, Spain.

Content

1. Materials and General methods 3
   1.1. Materials 3
   1.2. General methods 5
      1.2.1. HPLC analysis 5
      1.2.2. HPLC monitoring 5
      1.2.3. NMR analysis 5
      1.2.4. Thin layer chromatography (TLC) analysis 5
      1.2.5. Column chromatography 6
      1.2.6. Specific rotation 6
2. Molecular Biology Techniques and Protein Expression and Purification 6
   2.1. Cloning Strategies of DddK$_{Pu}$ wt and Variants 6
   2.2. Sequence of the DddK$_{Pu}$ wt and variants using in this study 7
   2.3. Small-scale Protein Expression and Purification (1 L) 10
   2.4. Large Scale Protein Expression and Purification 13

2.4.1. DddK$_{Pu}$ wt Expression and Purification (4 L) 13

2.4.2. DddK$_{Pu}$ W26G Expression and Purification (5 L) 14

3. Electrospray Ionization Mass Spectrometry (ESI-MS) of DddK$_{Pu}$ wt and variants16

4. Metal Analysis of DddK$_{Pu}$ wt and variants 17

5. Activity of the DddK$_{Pu}$ wt and the W26G variant... 18

5.1. Activity of the DddK$_{Pu}$ wt and the W26G variant toward the natural substrate DMSP18

5.1. Activity of the DddK$_{Pu}$ W26G variant toward the natural substrate DMSP in the presence of Ni^{2+} 19

5.2. Aza-Michaelase activity of the DddK$_{Pu}$ wt toward secondary amines (**6**)....... 20

6. Synthesis of starting material (6p, (*S*)-11).. 21

7. Screening of aza-Michael addition of primary and secondary amines to acrylic acid catalyzed by DddK$_{Pu}$ 24

7.1. Stock solution preparation 24

7.2. Aza-Michael addition of **4a-ab** to **2** catalyzed by DddK$_{Pu}$ W26G variant...... 24

7.3. Aza-Michael addition of **6a-ar** to **2** catalyzed by DddK$_{Pu}$ wt 25

8. Enzymatic synthesis of *N*-substituted and *N*,*N*-disubstituted-3-aminopropanoate 25

8.1. Enzymatic synthesis of N-substituted-3-aminopropanoate 25

8.2. Enzymatic synthesis of N,N-disubstituted-β-aminoacids................................ 30

8.3. Enzymatic synthesis of 3-((piperidin-2-ylmethyl)amino)propanoic acid and 3-(2-(aminomethyl)pyrrolidin-1-yl)propanoic acid 39

9. Computational Methods. 41

10. NMR Spectra. 44

References 137

1. Materials and General methods

1.1. Materials

Prop-2-en-1-amine·hydrochloride (**4c**), 1-aminoindane·hydrochloride (*rac*-**4h**), O-benzylhydroxylamine·hydrochloride (**4r**), 4-methylpiperazin-1-amine (**4s**), 2-(2-Pyridyl)ethylamine (**4t**), Tryptamine (**4u**), 3-(Aminomethyl)-1-methylindole (*rac*-**4v**), 3-aminopropan-1-ol (**4x**), 1-benzylpiperazine (**6q**), 1*H*-imidazole (**6u**) were purchased from Merck. Cyclohexylmethanamine (**4e**), aniline (**4f**), diphenylmethanamine (**4l**), naphthalen-1-ylmethanamine (**4m**), furan-2-ylmethanamine (**4p**), dimethylamine (**6a**), azetidine (**6b**), pyrrolidine (**6c**), (*R*)-pyrrolidin-2-ylmethanol (**6d**), piperidine (**6g**), azepane (**6i**), morpholine (**6j**), (1R,2R)-(−)-Pseudoephedrine (**6m**), 1,2,3,4-tetrahydroisoquinoline (**6s)**, 4-bromo-1*H*-pyrazole (**6v**), 1*H*-pyrazole (**6w**), 1*H*-1,2,4-triazole (**6z**), 1-oxa-4-azaspiro[4.5]decane (**6af**), 3-(cyclopropylamino)propanenitrile (**6ah**) and triethanolamine hydrochloride (TEA) were purchased from Sigma-Aldrich.

Metylamine·hydrochloride (**4a**), cyclopropanamine (**4b**), cyclohexanamine (**4d**), benzylamine·hydrochloride (**4g**), (*S*)-1,2,3,4-tetrahydronaphthalen-1-amine ((*S*)-**4i**), (*R*)-1,2,3,4-tetrahydronaphthalen-1-amine ((*R*)-**4i**), (*S*)-1-phenylethan-1-amine ((*S*)-**4j**), (*R*)-1-phenylethan-1-amine ((*R*)-**4j**), pyridin-2-ylmethanamine (**4k**), thiophen-2-ylmethanamine (**4o**), 2-(4-fluorophenyl)ethan-1-amine (**4q**), ((3r,5r,7r)-adamantan-1-yl)methanamine (**4aa**), (3s,5s,7s)-adamantan-1-amine (**4ab**), *D*-proline (**6e**), *L*-proline (**6f**), , nortriptyline·hydrochloride (**6n**), dibenzylamine (**6o**), isoindoline·hydrochloride (**6r**), 1-methyl-1*H*-tetrazole-5-thiol (**6aa**), *N*-methylhydroxylamine·hydrochloride (**6aq**), 2 aminomethylpiperidine (*rac*-**10**) were purchased from TCI chemical.1,2-Diphenylethane-1,2-diamine (**4y**), D-nipecotic acid (**6h**), 2,5-dihydro-1*H*-pyrrole (**6ad**), thiazolidine (**6ag**), 3-(benzylamino)propanenitrile (**6ai**), 3-hydroxyazetidine·hydrochloride (**6aj**), 1,4,8,11-tetraazacyclotetradecane (**6ao**), (*S*)-pyrrolidin-2-ylmethanamine dihydrochloride ((*S*)-**9**), (*S*)-Piperidine-2-carboxylic acid hydrochloride (precursor (*S*)-**11**) were purchased from Indagoo Research Chemicals.

Thiomorpholine (**6k**), (1*R*,3r,5*S*)-8-azabicyclo[3.2.1]octan-3-ol (**6l**), pyridin-4(1*H*)-one (**6t**), 4-bromo-1*H*-imidazole (**6x**), 1*H*-1,2,3-triazole (**6y**), 1*H*-benzo[*d*]imidazole (**6ab**), 6-chloro-9*H*-purine (**6ac**), 1,2,3,6-tetrahydropyridine (**6ae**), (*S*)-2-(trifluoromethyl)pyrrolidine (**6an**), were purchased from Apollo Scientific. 9*H*-Fluoren-9-amine (**4n**), cyclohexane-1,2-diamine (**4z**), 3,3-difluoroazetidine (**6ak**), 3,3-difluoropyrrolidine·hydrochloride (**6al**), 3-azabicyclo[3.1.0]hexane·hydrochloride (**6ar**),

Sodium acrylate (**2**) were purchased from Fluorochem Ltd. 2-Methylthiazolidine (**6am**), N^1,N^2-dibenzylethane-1,2-diamine (**6ap**) were purchased from Thermo Scientific; 2-aminoethan-1-ol (**4w**) from Glentham Life Sciences; (2-carboxyethyl)dimethylsulfonium chloride (**1**) from abcr GmbH. 3-(Benzyloxy)pyrrolidine·hydrochloride (**6p**) was synthesized in our lab using procedure described in this work (see Chapter 6*)*. All reagents for Molecular Biology were supplied from ThermoFisher Scientific. Culture media components for *E. coli* were from Pronadisa (Madrid, Spain). Antibiotics and IPTG were from Carl Roth. Nickel Sepharose™ High Performance was from Cytiva™. Water for analytical HPLC was obtained from an Arium pro ultrapure water purification system (Sartorius Stedim Biotech) and the rest of solvents used in this work were of analytical grade or HPLC grade. Bacterial strains, oligonucleotides and plasmids used in this study are listed in **Table S1**.

Table S1. Strains, plasmids and oligonucleotides used in this study.

**Strains**	**Relevant genotype**
E coli M15 [pREP4]	*nal*^{s} , *str*^{s} , *rif*^{s} , *thi*$^-$, *lac*$^-$, *ara*$^+$, *gal*$^+$, *mtl*$^-$, F$^-$, *recA*$^+$, *uvr*$^+$, *lon*$^+$, Km^R.
**Oligonucleotides**	**Sequences**
Primer *dddK* wt (53)	5' AATATATAGGATCCATGATTTTTGTGAAGAATCTGGCGAGCG 3'<br>BamHI
Primer *dddK* wt (35)	5' TATATATATAAGCTTTTAGCCGCTCTTCTGCTTCGCCGGGAAATACTCCACT TCC 3'<br>HindIII
Primer *dddK* W26G (53)	TGG(W) → GGC(G)<br>5' CGCATCACCATCACCATCACGGATCCATGATTTTTGTGAAGAATCTGGCGAG CGTTCTGAGCCAAGAGTGGAGCAGCACCGAGAAGTATCCGGGCGTTCGT***GGC***A AGTTCCTGATCGACGCG 3'
Primer *dddK* W26A (53)	TGG(W) → GCG(A)<br>5' CGCATCACCATCACCATCACGGATCCATGATTTTTGTGAAGAATCTGGCGAG CGTTCTGAGCCAAGAGTGGAGCAGCACCGAGAAGTATCCGGGCGTTCGT***GCG***A AGTTCCTGATCGACGCG 3'
Primer *dddK* W26X (X=F, M, V) (53)	TGG(W) → NTK(X)<br>5' CGCATCACCATCACCATCACGGATCCATGATTTTTGTGAAGAATCTGGCGAG CGTTCTGAGCCAAGAGTGGAGCAGCACCGAGAAGTATCCGGGCGTTCGT***NTK***A AGTTCCTGATCGACGCG 3'
Primer *dddK* W110X (X=A, V) (35)	TGG(W) → CRC(X)<br>5' GGAGTCCAAGCTCAGCTAATTAAGCTTTTAGCCGCTCTTCTGCTTCGCCGGG AAATACTCCACTTCGCTGAAACGGTCGGTCGGGAAAAT***CRC***ATAGAACTCCAG GGTTTCTTTGCCG 3'
Primer *dddK* H56A/H58A/E62A (35)	GTG(56H) → CGC(56A), GTG(58H) → CGC(58A), TTC(62E) → CGC(62A)<br>5' GCCGTTGGTAACCACATAGAT***CGC***CGCCGGGCT***CGC***GTA***CGC***CAGGGTACGA TCGCCACC 3'
Primer *dddK* H96A (35)	GTG(H) → CGC(A)<br>5' GCCGTTGTTCTTCAGCGC***CGC***TTCCGCGTTGCCCGC3'
**Plasmids**	**Relevant genetic characteristics**
pQE-40	The vector encodes one multiple cloning site preceded by a T5 promoter, *lac* operator and ribosome binding site. The vector also carries the ColE1 replicon and ampicillin resistance gene.

pQE-40-*dddK*	*DddK wt* gene (429 bp) from *Pelagibacter ubique* HTCC1062 (obtained from the protein sequence, PDB: 5TFZ) cloned in pQE-40 (*BamH*I and *Hind*III).
pQE-40-*dddK* W26X *(X=A, F, G, M, V,)*	*DddK wt* gene as template (Gibson assembly).
pQE-40-*dddK* W110X *(*X=A, V*)*	*DddK wt* gene as template (Gibson assembly).
pQE-40-*dddK* H56A/H58A/E62A/ H96A	*DddK* H96A gene as template (Gibson assembly).

1.2. General methods

1.2.1. HPLC analysis

HPLC analysis was performed on a RP-HPLC XBridge® C18, 5 µm, 4.6 × 250 mm column (Waters). The solvent system used was: solvent (A): 0.1% (v/v) trifluoroacetic acid (TFA) in H_2O and solvent (B): 0.095% (v/v) TFA in acetonitrile/water 4:1, flow rate 1 mL min^{-1}, detection at 215 nm and column temperature at 30 °C.

1.2.2. HPLC monitoring

Reaction monitoring for primary (**4**) and secondary (**6**) amines were carried out as follows: samples from **4a-ab** reaction mixture (10 µL) and samples from the **6a-ar** reaction mixture (25 µL) were withdrawn and diluted with a mixture of methanol/water (1:1) (490 µL) and methanol/water (1:1) (225 µL) respectively. After centrifugation at maximum speed for 5 minutes, the samples were analyzed by HPLC as described above. Elution condition: isocratic 10% B over 10 minutes, except for **4f-n**, **4q-r**, **4t-v**, **6m-s**, **6ab**, **6ai**, **6ap**: gradient from 10 to 55% B over 15 min. Retention times are given in minutes. Substrate depletion was quantified by analyzing the peak areas of acrylate (**2**) using a calibration curve within the linear range of 9.3 mM to 200 mM.

1.2.3. NMR analysis

Routine ^{1}H (400 MHz) and ^{13}C (101 MHz) NMR spectra of compounds were recorded with a Varian Mercury-400 spectrometer. Full characterization of the described compounds was performed using typical gradient-enhanced 2D experiments: COSY, HSQC, NOESY and HMBC recorded under routine conditions.

1.2.4. Thin layer chromatography (TLC) analysis

TLC analysis was performed using precoated silica gel plates with or without fluorescent indicator UV254 (Macherey-Nagel GmbH & Co. KG, Layer: 0.20 mm silica gel 60). Stains were detected on TLC plates using UV 254 fluorescence or developed with

potassium permanganate ($KMnO_4$) stain ($KMnO_4$ (1.5 g L^{-1}), K_2CO_3 (10 g L^{-1}), and NaOH (10% aqueous, 1 mL) in water).

1.2.5. *Column chromatography*

Column chromatography was performed in a glass column (AFORA, 5880/2, 47x4.5) packed with silica gel (100 g, 35-70 µm, 200-500 mesh, Merck).

1.2.6. *Specific rotation*

Specific rotation values were measured with a Perkin Elmer Model 341 (Überlingen, Germany) (Na Lamp, 589 nm). Products (2 mg) were dissolved in MeOH (2 mL) and the samples were analyzed at room temperature 1.0 dm cell with polarized light (Na D line 589 nm) at 25 ºC.

2. Molecular Biology Techniques and Protein Expression and Purification

2.1. *Cloning Strategies of DddK$_{Pu}$ wt and Variants*

The coding sequence for DddK from *Pelagibacter ubique* HTCC1062 (obtained from the protein sequence, PDB: 5TFZ[1]) (DddK$_{Pu}$)was codon-optimized and inserted into a pQE-40 gene vector (digested with *BamHI* and *HindIII*) by the Gene Synthesis Services of GenScript. The same strategy was followed for cloning the DddK$_{Pu}$ variants (**Figure S1**).

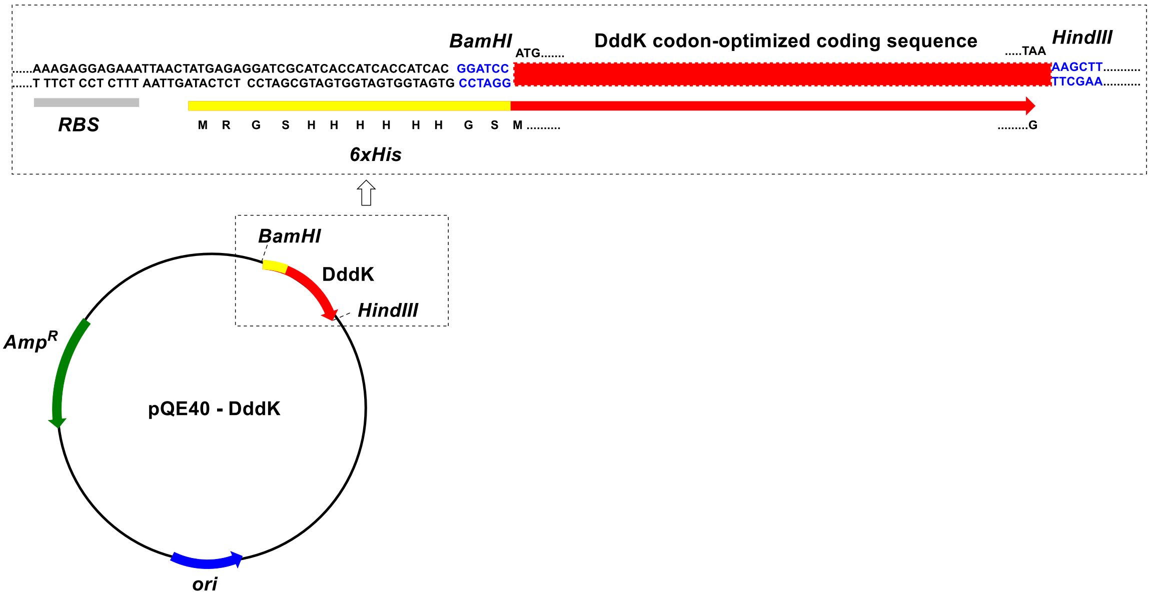

Figure S1. Strategy followed to clone the coding sequence of DddK$_{Pu}$ into a pQE-40 plasmid (QUIAGEN) using BamHI and HindIII as restriction enzymes. Protein was expressed with the additional N-terminal

sequence: **MRGSHHHHHHGS**, incorporated by the open reading frame of the pQE-40. **Ori**, high-copy-number ColE1/pMB1/pBR322/pUC origin of replication, **Amp**^{R}, β-lactamase expressing gene, **RBS**, strong bacterial ribosome binding site and **6xHis**, 6xHis affinity tag.

2.2. Sequence of the DddK$_{Pu}$ wt and variants using in this study

Codon-optimized coding sequence (**A**) and protein sequence after insertion into a pQE-40 (**B**). The mutated amino acid and the corresponding codon are highlighted in green. The additional *N*-terminal HisTag sequence incorporated by the open reading frame of the pQE-40 is highlighted in yellow.

DddK$_{Pu}$ **wt**

A

ATGAGAGGATCGCATCACCATCACCATCACGGATCC**ATG**ATTTTTGTGAAGAATCTGGCGAGCGTTCTGA
GCCAAGAGTGGAGCAGCACCGAGAAGTATCCGGGCGTTCGTTGGAAGTTCCTGATCGACGCGGATTTCGA
CGGTAGCAGCGGCCTGAGCCTGGGTTTTGCGGAGATTGCGCCGGGTGGCGATCTGACCCTGCACTACCAC
AGCCCGGCGGAAATCTATGTGGTTACCAACGGCAAGGGCATTCTGAACAAGAGCGGTAAACTGGAAACCA
TCAAGAAAGGTGACGTGGTTTACATTGCGGGCAACGCGGAACACGCGCTGAAGAACAACGGCAAAGAAAC
CCTGGAGTTCTATTGGATTTTCCCGACCGACCGTTTCAGCGAAGTGGAGTATTTCCCGGCGAAGCAGAAG
AGCGGCTAA

B

MRGSHHHHHHGS**M**IFVKNLASVLSQEWSSTEKYPGVRWKFLIDADFDGSSGLSLGFAEIAPGGDLHTLHY
HSPAEIYVVTNGKGILNKSGKLETIKKGDVVYIAGNAEHALKNNGKETLEFYWIFPTDRFSEVEYFPAKQ
KSG*

DddK$_{Pu}$ W26F

A

ATGAGAGGATCGCATCACCATCACCATCACGGATCC**ATG**ATGATTTTTGTGAAGAATCTGGCGAGCGTTC
TGAGCCAAGAGTGGAGCAGCACCGAGAAGTATCCGGGCGTTCGTTTTAAGTTCCTGATCGACGCGGATTT
CGACGGTAGCAGCGGCCTGAGCCTGGGTTTTGCGGAGATTGCGCCGGGTGGCGATCTGACCCTGCACTAC
CACAGCCCGGCGGAAATCTATGTGGTTACCAACGGCAAGGGCATTCTGAACAAGAGCGGTAAACTGGAAA
CCATCAAGAAAGGTGACGTGGTTTACATTGCGGGCAACGCGGAACACGCGCTGAAGAACAACGGCAAAGA
AACCCTGGAGTTCTATTGGATTTTCCCGACCGACCGTTTCAGCGAAGTGGAGTATTTCCCGGCGAAGCAG
AAGAGCGGCTAA

B

MRGSHHHHHHGS**M**IFVKNLASVLSQEWSSTEKYPGVRFKFLIDADFDGSSGLSLGFAEIAPGGDLTLHYH
SPAEIYVVTNGKGILNKSGKLETIKKGDVVYIAGNAEHALKNNGKETLEFYWIFPTDRFSEVEYFPAKQK
SG*

DddK$_{Pu}$ W26M

A

ATGAGAGGATCGCATCACCATCACCATCACGGATCC**ATG**ATGATTTTTGTGAAGAATCTGGCGAGCGTTC
TGAGCCAAGAGTGGAGCAGCACCGAGAAGTATCCGGGCGTTCGTATGAAGTTCCTGATCGACGCGGATTT
CGACGGTAGCAGCGGCCTGAGCCTGGGTTTTGCGGAGATTGCGCCGGGTGGCGATCTGACCCTGCACTAC
CACAGCCCGGCGGAAATCTATGTGGTTACCAACGGCAAGGGCATTCTGAACAAGAGCGGTAAACTGGAAA
CCATCAAGAAAGGTGACGTGGTTTACATTGCGGGCAACGCGGAACACGCGCTGAAGAACAACGGCAAAGA
AACCCTGGAGTTCTATTGGATTTTCCCGACCGACCGTTTCAGCGAAGTGGAGTATTTCCCGGCGAAGCAG
AAGAGCGGCTAA

B

MRGSHHHHHHGS**M**IFVKNLASVLSQEWSSTEKYPGVRMKFLIDADFDGSSGLSLGFAEIAPGGDLTLHYH
SPAEIYVVTNGKGILNKSGKLETIKKGDVVYIAGNAEHALKNNGKETLEFYWIFPTDRFSEVEYFPAKQK
SG*

DddK$_{Pu}$ W26V

A

ATGAGAGGATCGCATCACCATCACCATCACGGATCC**ATG**ATGATTTTTGTGAAGAATCTGGCGAGCGTTC
TGAGCCAAGAGTGGAGCAGCACCGAGAAGTATCCGGGCGTTCGTGTGAAGTTCCTGATCGACGCGGATTT
CGACGGTAGCAGCGGCCTGAGCCTGGGTTTTGCGGAGATTGCGCCGGGTGGCGATCTGACCCTGCACTAC
CACAGCCCGGCGGAAATCTATGTGGTTACCAACGGCAAGGGCATTCTGAACAAGAGCGGTAAACTGGAAA
CCATCAAGAAAGGTGACGTGGTTTACATTGCGGGCAACGCGGAACACGCGCTGAAGAACAACGGCAAAGA
AACCCTGGAGTTCTATTGGATTTTCCCGACCGACCGTTTCAGCGAAGTGGAGTATTTCCCGGCGAAGCAG
AAGAGCGGCTAA

B

MRGSHHHHHHGS**M**IFVKNLASVLSQEWSSTEKYPGVRVKFLIDADFDGSSGLSLGFAEIAPGGDLTLHYH
SPAEIYVVTNGKGILNKSGKLETIKKGDVVYIAGNAEHALKNNGKETLEFYWIFPTDRFSEVEYFPAKQK
SG*

DddK$_{Pu}$ W26A

A

ATGAGAGGATCGCATCACCATCACCATCACGGATCC**ATG**ATGATTTTTGTGAAGAATCTGGCGAGCGTTC
TGAGCCAAGAGTGGAGCAGCACCGAGAAGTATCCGGGCGTTCGTGCGAAGTTCCTGATCGACGCGGATTT
CGACGGTAGCAGCGGCCTGAGCCTGGGTTTTGCGGAGATTGCGCCGGGTGGCGATCTGACCCTGCACTAC
CACAGCCCGGCGGAAATCTATGTGGTTACCAACGGCAAGGGCATTCTGAACAAGAGCGGTAAACTGGAAA
CCATCAAGAAAGGTGACGTGGTTTACATTGCGGGCAACGCGGAACACGCGCTGAAGAACAACGGCAAAGA
AACCCTGGAGTTCTATTGGATTTTCCCGACCGACCGTTTCAGCGAAGTGGAGTATTTCCCGGCGAAGCAG
AAGAGCGGCTAA

B

MRGSHHHHHHGS**M**IFVKNLASVLSQEWSSTEKYPGVRAKFLIDADFDGSSGLSLGFAEIAPGGDLTLHYH
SPAEIYVVTNGKGILNKSGKLETIKKGDVVYIAGNAEHALKNNGKETLEFYWIFPTDRFSEVEYFPAKQK
SG*

DddK$_{Pu}$ W26G

A

ATGAGAGGATCGCATCACCATCACCATCACGGATCC**ATG**ATGATTTTTGTGAAGAATCTGGCGAGCGTTC
TGAGCCAAGAGTGGAGCAGCACCGAGAAGTATCCGGGCGTTCGTGGCAAGTTCCTGATCGACGCGGATTT
CGACGGTAGCAGCGGCCTGAGCCTGGGTTTTGCGGAGATTGCGCCGGGTGGCGATCTGACCCTGCACTAC
CACAGCCCGGCGGAAATCTATGTGGTTACCAACGGCAAGGGCATTCTGAACAAGAGCGGTAAACTGGAAA
CCATCAAGAAAGGTGACGTGGTTTACATTGCGGGCAACGCGGAACACGCGCTGAAGAACAACGGCAAAGA
AACCCTGGAGTTCTATTGGATTTTCCCGACCGACCGTTTCAGCGAAGTGGAGTATTTCCCGGCGAAGCAG
AAGAGCGGCTAA

B

MRGSHHHHHHGS**M**IFVKNLASVLSQEWSSTEKYPGVRGKFLIDADFDGSSGLSLGFAEIAPGGDLTLHYH
SPAEIYVVTNGKGILNKSGKLETIKKGDVVYIAGNAEHALKNNGKETLEFYWIFPTDRFSEVEYFPAKQK
SG*

DddK$_{Pu}$ W110V

A

ATGAGAGGATCGCATCACCATCACCATCACGGATCC**ATG**ATGAGAGGATCGCATCACCATCACCATCACG
GATCCATGATTTTTGTGAAGAATCTGGCGAGCGTTCTGAGCCAAGAGTGGAGCAGCACCGAGAAGTATCC
GGGCGTTCGTTGGAAGTTCCTGATCGACGCGGATTTCGACGGTAGCAGCGGCCTGAGCCTGGGTTTTGCG
GAGATTGCGCCGGGTGGCGATCTGACCCTGCACTACCACAGCCCGGCGGAAATCTATGTGGTTACCAACG
GCAAGGGCATTCTGAACAAGAGCGGTAAACTGGAAACCATCAAGAAAGGTGACGTGGTTTACATTGCGGG
CAACGCGGAACACGCGCTGAAGAACAACGGCAAAGAAACCCTGGAGTTCTATGUGATTTTCCCGACCGAC
CGTTTCAGCGAAGTGGAGTATTTCCCGGCGAAGCAGAAGAGCGGCTAA

B

MRGSHHHHHHGS**M**IFVKNLASVLSQEWSSTEKYPGVRWKFLIDADFDGSSGLSLGFAEIAPGGDLTLHYH
SPAEIYVVTNGKGILNKSGKLETIKKGDVVYIAGNAEHALKNNGKETLEFYVIFPTDRFSEVEYFPAKQK
SG*

DddK$_{Pu}$ W110A

A

ATGAGAGGATCGCATCACCATCACCATCACGGATCC**ATG**ATGAGAGGATCGCATCACCATCACCATCACG
GATCCATGATTTTTGTGAAGAATCTGGCGAGCGTTCTGAGCCAAGAGTGGAGCAGCACCGAGAAGTATCC
GGGCGTTCGTTGGAAGTTCCTGATCGACGCGGATTTCGACGGTAGCAGCGGCCTGAGCCTGGGTTTTGCG
GAGATTGCGCCGGGTGGCGATCTGACCCTGCACTACCACAGCCCGGCGGAAATCTATGTGGTTACCAACG
GCAAGGGCATTCTGAACAAGAGCGGTAAACTGGAAACCATCAAGAAAGGTGACGTGGTTTACATTGCGGG
CAACGCGGAACACGCGCTGAAGAACAACGGCAAAGAAACCCTGGAGTTCTATGCGATTTTCCCGACCGAC
CGTTTCAGCGAAGTGGAGTATTTCCCGGCGAAGCAGAAGAGCGGCTAA

B

MRGSHHHHHHGS**M**IFVKNLASVLSQEWSSTEKYPGVRWKFLIDADFDGSSGLSLGFAEIAPGGDLTLHYH
SPAEIYVVTNGKGILNKSGKLETIKKGDVVYIAGNAEHALKNNGKETLEFYAIFPTDRFSEVEYFPAKQK
SG*

DddK$_{Pu}$ H56A/H58A/E62A/ H96A

A

ATGAGAGGATCGCATCACCATCACCATCACGGATCC**ATG**ATGATTTTTGTGAAGAATCTGGCGAGCGTTC
TGAGCCAAGAGTGGAGCAGCACCGAGAAGTATCCGGGCGTTCGTTGGAAGTTCCTGATCGACGCGGATTT
CGACGGTAGCAGCGGCCTGAGCCTGGGTTTTGCGGAGATTGCGCCGGGTGGCGATCTGACCCTGGCGTAC
GCGAGCCCGGCGGCGATCTATGTGGTTACCAACGGCAAGGGCATTCTGAACAAGAGCGGTAAACTGGAAA
CCATCAAGAAAGGTGACGTGGTTTACATTGCGGGCAACGCGGAAGCGGCGCTGAAGAACAACGGCAAAGA
AACCCTGGAGTTCTATTGGATTTTCCCGACCGACCGTTTCAGCGAAGTGGAGTATTTCCCGGCGAAGCAG
AAGAGCGGCTAA

B

MRGSHHHHHHGS**M**IFVKNLASVLSQEWSSTEKYPGVRWKFLIDADFDGSSGLSLGFAEIAPGGDLTLAYA
SPAAIYVVTNGKGILNKSGKLETIKKGDVVYIAGNAEAALKNNGKETLEFYWIFPTDRFSEVEYFPAKQK
SG

2.3. *Small-scale Protein Expression and Purification (1 L)*

Expression: Homemade chemically competent cells (*E. coli* M-15[pREP-4] strain from QIAGEN, usually 10^4-10^5 CFU μg DNA^{-1}) were transformed with the corresponding plasmid following the typical heat-shock procedure[2]. The transformed cells were selected for antibiotic resistance by plating the culture in LB agar plates supplemented with ampicillin (200 μg mL^{-1}) plus kanamycin (50 μg mL^{-1}) and incubated at 37 °C

overnight. A portion of resulting lawns of *E. coli* were scraped off the plate and transferred into a baffled shaker flask (100 mL) containing LB medium (30 mL) supplemented with ampicillin (200 µg mL^{-1}) plus kanamycin (50 µg mL^{-1}), and grown at 37 °C on a rotary shaker at 200 rpm overnight (OD_{600} ≈ 2-3). An aliquot of the pre-culture (20 mL) was transferred into a baffled shaker flask (2 L) containing LB medium (1 L) supplemented with ampicillin (200 µg mL^{-1}), kanamycin (50 µg mL^{-1}) and antifoam SE-15 (0.04% v/v). The culture was incubated at 37 °C with shaking at 200 rpm. During the middle exponential phase growth (DO_{600} ≈ 0.5-0.8), the culture was cooled down to 20 ºC while the shaking was reduced to 100 rpm. After 1 h, the protein expression was induced with IPTG (0.1 mM final concentration) at 20 ºC and the agitation was increased to 200 rpm. After 12-16 h, the culture (1 L) was centrifuged (2500 g for 30 min at 4 °C) and the pellet (typically 4-5 g) was frozen overnight at – 20 ºC.

Purification: Cells were lysed using NZY Bacterial Cell Lysis Buffer (Nzytech, MB17801, 0.2 g of cells past mL^{-1} lysis buffer supplemented with lysozyme (100 µg mL^{-1}) and DNase (4 µg mL^{-1}) final concentration in the lysis mixture). The cell suspension was shaking at 25 ºC and 200 rpm for 30 minutes and the cellular debris was removed by centrifugation (35000 g for 30 min at 4 °C). The clear supernatant was applied by gravity flow into a His GraviTrapTM affinity column (Cytiva, 11003399), which was previously equilibrated with 10 mL (10 CV) of Buffer A (50 mM Tris/HCl buffer pH 7.4, 300 mM NaCl, and 20 mM imidazole). After sample application, the resin was washed with 10 mL (10 CV) of Buffer A and the protein was eluted with 10 mL (10 CV) of Buffer B (50 mM Tris/HCl buffer pH 7.4, 300 mM NaCl, and 500 mM imidazole). The purified protein (10 mL) was dialyzed against Buffer C (5 mM HEPES buffer pH 7.5, 100 mM NaCl, and 50% (v/v) glycerol, 3 x 1 L 24 h each). The blue dialyzed solution (3-4 mL) was stored at – 20 °C. Each purification step was analyzed by SDS-PAGE (**Figure S2**) and the protein concentration was determined by absorption at 280 nm using extinction coefficient calculated by ProtParam (https://web.expasy.org/protparam/) assuming all Cys residues are reduced (**Table S2)**.

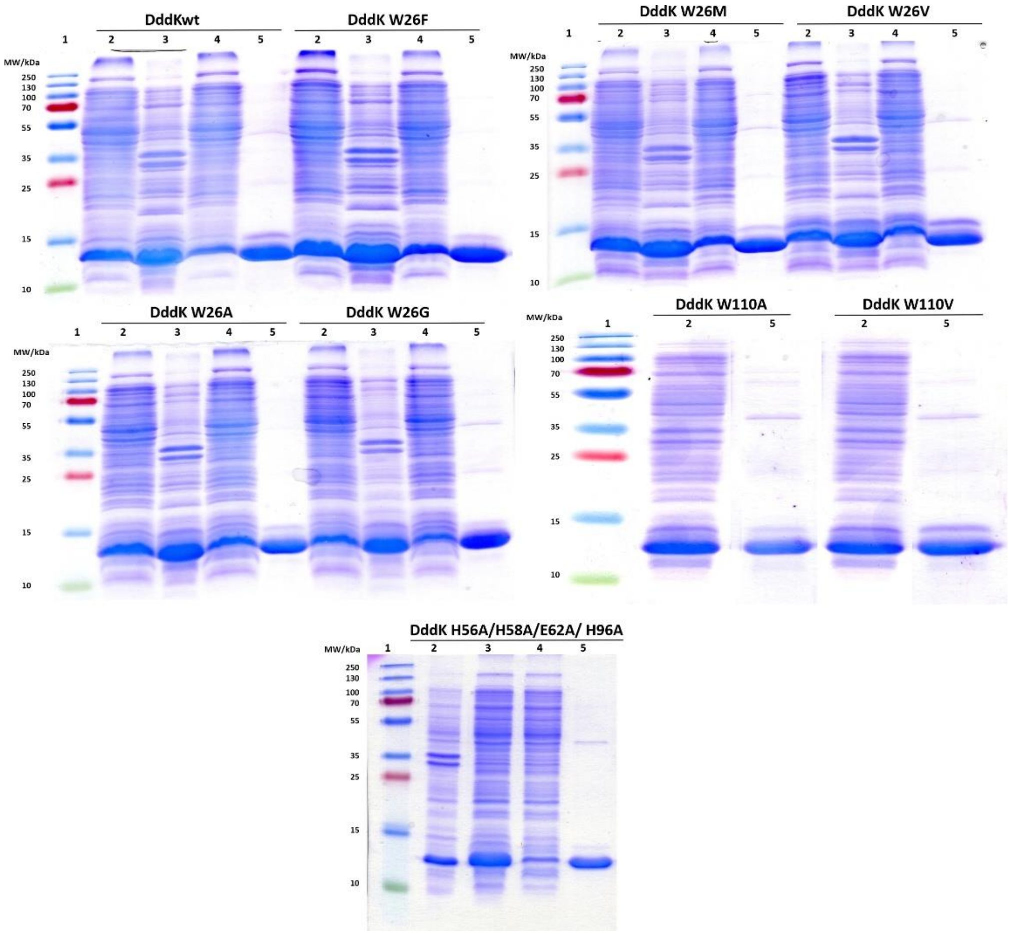

Figure S2. Analysis of DddK$_{Pu}$ wt and variants purification steps by Coomassie Blue-stained SDS-PAGE. In each case, the gel was loaded with samples from the lysis supernatant (lane 2), post-lysis pellet (lane 3), flow-through fraction (lane 4) and elution fraction (lane 5). The molecular masses of the proteins in the standard molecular weight marker are as indicated (lane 1, Thermo Scientific PageRuler Prestained Protein Ladder, 26619). The average molecular weight of DddK$_{Pu}$ wt and variants based on their sequences is 15.7 kDa. In our hands, the protein showed anomalous SDS-PAGE migration (Tris-glycine-SDS buffer and 15% gel). The monoisotopic molecular weight of DddK$_{Pu}$ wt and variants was determined by mass spectrometry (ESI-MS) (see section 3).

Table S2. Typical enzyme concentration obtained after expression and purification as describe above.

DddK$_{Pu}$ variants	Abs 0.1% (= 1 g L^{-1})[a]	C(protein)/mg mL^{-1}
**wt**	1.6	12.1
**W26F**	1.3	12.5
**W26M**	1.3	12.2
**W26V**	1.3	12.3
**W26A**	1.3	11.2
**W26G**	1.3	8.8
**W110V**	1.3	6.6
**W110A**	1.3	5.1
**H56A/H58A/E62A/H96A**	1.6	10.0

[a]Calculated by ProtParam (https://web.expasy.org/protparam/) assuming all Cys residues are reduced.

2.4. Large Scale Protein Expression and Purification

2.4.1. DddK$_{Pu}$ wt Expression and Purification (4 L)

Expression: Homemade chemically competent cells (*E. coli* M-15[pREP-4] strain from QIAGEN, typically 10^4-10^5 CFU µg DNA^{-1}) were transformed with the pQE-40- DddK$_{Pu}$ wt plasmid following the typical heat-shock procedure[2] The transformed cells were selected for antibiotic resistance by plating the culture in LB agar plates supplemented with ampicillin (200 µg mL^{-1}) plus kanamycin (50 µg mL^{-1}) and incubated at 37 °C overnight. A portion of resulting lawns of *E. coli* were scraped off the plates and transferred into a baffled shaker flask (500 mL) containing LB medium (100 mL) supplemented with ampicillin (200 µg mL^{-1}) plus kanamycin (50 µg mL^{-1}), and grown at 37 °C on a rotary shaker at 200 rpm overnight ($OD_{600} \approx$ 2-3). An aliquot of the pre-culture (20 mL) was transferred into a baffled shaker flask (2 L) containing TB medium (1 L) supplemented with ampicillin (200 µg mL^{-1}), kanamycin (50 µg mL^{-1}) and antifoam SE-15 (0.04% v/v). The culture was incubated at 37 °C with shaking at 200 rpm. During the middle exponential phase growth ($DO_{600} \approx$ 0.5-0.8), the culture was cooled down to 30 ºC while the shaking was reduced to 100 rpm. After 1 h, the protein expression was induced with IPTG (1 mM final concentration) at 30 ºC and at 30 ºC and shaking was increased to 200 rpm. After 12-16 h, the culture (4 L) was centrifuged (2500 g for 30 min at 4 °C) and the pellet (typically 30 g) was frozen overnight at – 20 ºC. **Purification:** Cells were resuspended in the lysis Buffer (400 mL, 50 mM sodium phosphate buffer pH 8.0, 300 mM NaCl, and 20 mM imidazole). The suspension (0.075 g of cells past mL^{-1} lysis buffer) was lysed using a cell disrupter (Constant Systems) and cellular debris was removed by centrifugation (35000 g for 30 min at 4 °C). The clear supernatant was applied at 3 mL min^{-1} into a cooled HiScale™ 16/40 column (Cytiva, 28964424) packed with Nickel Sepharose™ High Performance (50 mL bed volume, Cytiva, 17526802) previously equilibrated with 250 mL (5 CV) of lysis buffer. After sample application, the column was washed with 400 mL (8 VC) of lysis buffer at 6 mL min^{-1} and the protein was eluted with 200 mL (4 CV) of elution buffer (50 mM sodium phosphate buffer pH 8.0, 300 mM NaCl, and 500 mM imidazole) at a flow rate of 6 mL min^{-1}. The purified protein was dialyzed against buffer C (5 mM HEPES buffer pH 7.5, 100 mM NaCl, and

50% (v/v) glycerol, 3 x 1 L 24 h each). The blue dialyzed solution (50 mL) was stored at – 20 °C. Each purification step was analyzed by SDS-PAGE (**Figure S3**) and the protein concentration was determined by absorption at 280 nm using extinction coefficient calculated by ProtParam (https://web.expasy.org/protparam/) assuming that all Cys residues are reduced, DddK$_{Pu}$ wt Abs 0.1% (=1 g L^{-1}) =1.6 (**Table S3**).

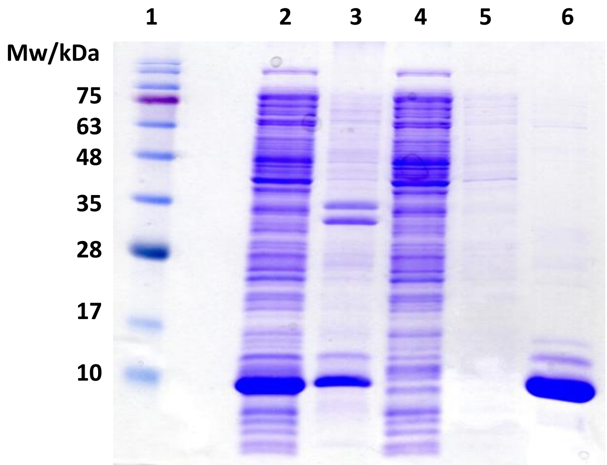

Figure S3. Analysis of DddK$_{Pu}$ wt purification steps by Coomassie Blue-stained SDS-PAGE. The gel was loaded with samples from the lysis supernatant (lane 2), post-lysis pellet (lane 3), flow-through fraction (lane 4), wash fraction (lane 5) and elution fraction (lane 6). The molecular masses of the proteins in the standard molecular weight marker are as indicated (lane 1, BlueStar Prestained Protein Marker, MWP03). The predicted molecular weight of DddK$_{Pu}$ wt based on its sequence is 15.8 kDa. In our hands, the protein showed anomalous SDS-PAGE migration (Tris-glycine-SDS buffer and 15% gel). The monoisotopic molecular weight of DddK$_{Pu}$ wt and variants was determined by mass spectrometry (ESI-MS) (see section 3).

Table S3. Expression and purification parameter of DddK$_{Pu}$ wt.

**V(culture)/L**	4
**m(Pellet *E. coli*)/g**	30
**V(after dialysis)/mL**	50
**C(protein)/mg mL^{-1}**	18
**m(protein)/mg**	900
**Protein yield**	30[a], 225[b]

[a]mg protein g^{-1} pellet, [b]mg protein L^{-1} culture

2.4.2. *DddK$_{Pu}$ W26G Expression and Purification (5 L)*

Expression: Homemade chemically competent cells (*E. coli* M-15[pREP-4] strain from QIAGEN, typically 10^4-10^5 CFU µg DNA^{-1}) were transformed with the pQE-40- DddK$_{Pu}$

W26G plasmid following the typical heat-shock procedure.[2] The transformed cells were selected for antibiotic resistance by plating the culture in LB agar plates supplemented with ampicillin (200 μg mL^{-1}) plus kanamycin (50 μg mL^{-1}) and incubated at 37 °C overnight. A portion of resulting lawns of *E. coli* were scraped off the plates and transferred into a baffled shaker flask (500 mL) containing LB medium (100 mL) supplemented with ampicillin (200 μg mL^{-1}) plus kanamycin (50 μg mL^{-1}), and grown at 37 °C on a rotary shaker at 200 rpm overnight (OD_{600} ≈ 2-3). An aliquot of the pre-culture (20 mL) was transferred into a baffled shaker flask (2 L) containing Terrific Broth Medium (1 L) supplemented with ampicillin (200 μg mL^{-1}), kanamycin (50 μg mL^{-1}) and antifoam SE-15 (0.04% v/v). The culture was incubated at 37 °C with shaking at 200 rpm. During the middle exponential phase growth (DO_{600} ≈ 0.5-0.8), the culture was cooled down to 20 ºC while shaking was reduced to 100 rpm. After 1 h, the protein expression was induced with IPTG (0.1 mM final concentration) at 20 ºC and shaking was increased to 200 rpm. After 12-16 h, the culture (5 L) was centrifuged (2500 g for 30 min at 4 °C) and the pellet (typically 26 g) was frozen overnight at – 20 ºC. **Purification:** Cells were suspended in 128 mL of NZY bacterial cell lysis Buffer (Nzytech, MB17801, 0.2 g of cells past mL^{-1} lysis buffer supplemented with lysozyme (100 μg mL^{-1}) and DNase (4 μg mL^{-1}) final concentration in the lysis mixture). The cell suspension was shaking at 25 ºC and 200 rpm for 30 minutes and the cellular debris was removed by centrifugation (35000 g for 30 min at 4 °C). The clear supernatant was redistributed onto eight His GraviTrapTM affinity column (Cytiva, 11003399, 16 mL of supernatant or 3.2 g pellet per column) previously equilibrated with 10 mL (10 CV) of Buffer A (50 mM Tris/HCl buffer pH 7.4, 300 mM NaCl and 20 mM imidazole). After sample application at gravity flow, each column was washed with 10 mL (10 CV) of buffer A and the protein was eluted with 10 mL (10 CV) of buffer B (50 mM Tris/HCl buffer pH 7.4, 300 mM NaCl, and 500 mM imidazole). The purified protein solutions were combined (80 mL total volume) and dialyzed against buffer C (5 mM HEPES buffer pH 7.5, 100 mM NaCl, and 50% (v/v) glycerol, 3 x 1 L 24 h each). The blue dialyzed solution (21 mL) was stored at – 20 °C. Each purification step was analyzed by SDS-PAGE (**Figure S4**) and the protein concentration was determined by absorption at 280 nm using extinction coefficient calculated by ProtParam (https://web.expasy.org/protparam/) assuming all Cys residues are reduced, $DddK_{Pu}$ W26G Abs 0.1% (= 1 g L^{-1}) =1.3 (**Table S4**).

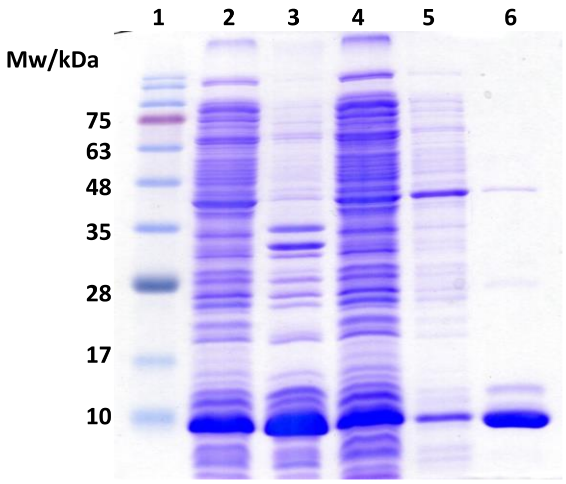

Figure S4. Analysis of DddK$_{Pu}$ W26G purification steps by Coomassie Blue-stained SDS-PAGE. The gel was loaded with samples from the lysis supernatant (lane 2), post-lysis pellet (lane 3), flow-through fraction (lane 4), wash fraction (lane 5) and elution fraction (lane 6). The molecular masses of the proteins in the standard molecular weight marker are as indicated (lane 1, BlueStar Prestained Protein Marker, MWP03). The predicted molecular weight of DddK$_{Pu}$ W26G based on its sequence is 15.7 kDa. In our hands, the protein showed anomalous SDS-PAGE migration (Tris-glycine-SDS buffer and 15% gel). The monoisotopic molecular weight of DddK$_{Pu}$ wt and variants was determined by mass spectrometry (ESI-MS) (see section 3).

Table S4. Expression and purification parameter of DddK$_{Pu}$ W26G.

**V(culture)/L**	5
**m(Pellet *E. coli*)/g**	26
**V(after dialysis)/mL**	21
**C(protein)/mg mL^{-1}**	8.2
**m(protein)/mg**	172
**Protein yield**	7^{a}, 34^{b}

^{a}mg protein g pellet^{-1}, ^{b}mg protein L culture^{-1}

3. Electrospray Ionization Mass Spectrometry (ESI-MS) of DddK$_{Pu}$ wt and variants

The glycerinated protein solution was diluted with H_2O containing HCOOH (0.1% v/v) (0.1% v/v) (150 µL, 1 mg protein mL^{-1} final concentration). This solution was applied to a PD MiniTrap G-10 column and eluted according to the manufacturer's protocol (Cytiva, 28918010). Samples of desalted protein were injected (5 µL) into an Acquity UPLC BEH C4, 300 Å, 1.7 µm, 2.1 x 50 mm column fitted to a UPLC (Aquity Premier, Waters, Milford, MA, USA) instrument. The solvent system used was: solvent A, H_2O/HCOOH

(0.1% v/v) and solvent B, 0.1% HCOOH in CH_3CN/H_2O (70:30, v/v). Gradient elution: 28 % B to 100 % B in 25 min, flow rate 0.2 mL min^{-1}. The UPLC was connected to an ESI-TOF mass spectrometer (Cyclic IMS, SELECT Series Waters, Milford, MA, USA), with a dual ESI source (LockSpray). The second spray provided the lock mass calibration with leucine enkephalin (m/z 556.2766). The ESI-TOF was operated in the V-optics mode to provide a mass resolution of at least 60,000 full width at half maximum (FWHM). The acquisition time per spectrum was set to 0.5 s, and the mass range was from 50 to 1400 Da. Data were acquired with a cone voltage of 25 V, capillary voltage of 3000 V, a desolvation temperature of 250 °C, and a source temperature of 100 °C. The desolvation gas flow was set at 800 L h^{-1} and the cone gas flow was set at 6 L h^{-1}. MassLynx 4.2 software (Waters. Milford, MA, USA) was used for data acquisition and processing. UniDec Version 6.0.4 software, kindly provided by Prof. Michael T. Marty (Department of Chemistry, Physical and Theoretical Chemistry Laboratory, University of Oxford Oxford OX1 3QZ, UK),[3] was used for molecular weight deconvolution from ESI-MS spectra of proteins (**Table S5**).

Table S5. Molecular weight determination by ESI-MS of DddK$_{Pu}$ wt and variants.

**DddK$_{Pu}$ variants**	**Theoretical Molecular Weight^a/Da**	**Experimental Molecular Weight/Da**	**Δ/Da**
	Monoisotopic	Monoisotopic	
**wt**	15811.96	15812.0	0.04
**W26F**	15772.95	15772.6	0.35
**W26M**	15756.92	15757.2	0.28
**W26V**	15724.95	15725.1	0.15
**W26A**	15696.92	15696.9	0.02
**W26G**	15682.90	15683.1	0.2
**W110V**	15724.95	15725.2	0.25
**W110A**	15696.92	15697.2	0.28
**H56A/H58A/E62A/H96A**	15555.89	15556.1	0.21

^{a}Monoisotopic molecular weight was calculated using ProtParam (https://web.expasy.org/protparam/).

4. Metal Analysis of DddK$_{Pu}$ wt and variants

The glycerinated protein solution was diluted with Milli-Q water and the protein was precipitated with commercial HNO_3 for trace metal analysis (6 mL final volume, 1 mg

mL^{-1} protein concentration and HNO_3 2 % (v/v) final concentration in the mixture). The solution was stored at 4 °C for 12 h and the precipitate was removed by centrifugation at 15 000 g for 30 minutes. The supernatant was filtered through a syringe filter (CORNING 28 mm, 0.2 mm, SFCA + PF membrane) and the solution was used to quantify the metal content (ICP-MS/AES service, Institute of Environmental Assessment and Water Research, IDAEA-CSIC). The content of Fe was determined by ICP-AES (Thermo Scientific iCAP PRO X Radial) while the content of Mn, Co, Ni, Cu and Zn was analyzed by ICP-MS (iCAP-RQ THERMO) (**Table S6**).

Table S6. Metal content of DddK$_{Pu}$ wt and variants.

	**Metal content[a] /% metal per DddK$_{Pu}$ monomer**					
**DddK$_{Pu}$ variants**	**Mn**	**Fe**	**Co**	**Ni**	**Cu**	**Zn**
**wt**	2.90 ± 0.03	29.7 ± 0.2	-[b]	29.4 ± 0.2	1.20 ± 0.02	43.7 ± 1.6
**W26F**	2.03 ± 0.04	13.4 ±0.6	-[b]	5.4 ±0.1	1.75 ± 0.06	19.7 ± 2.0
**W26M**	2.00 ± 0.02	14.3 ± 0.2	-[b]	7.00 ± 0.04	2.00 ± 0.05	20.7 ± 0.5
**W26V**	5.62 ± 0.04	27.40 ± 07	-[b]	12.4 ± 0.1	2.4 ± 0.03	18.4 ± 0.8
**W26A**	5.59 ± 0.01	30.4 ± 0.1	-[b]	5.90 ± 0.03	2.07 ± 0.01	25.1 ± 2.3
**W26G**	5.7 ± 0.1	47.0 ± 0.8	-[b]	11.6 ± 0.4	1.30 ± 0.06	14.3 ± 4.6
**H56A/H58A /E62A/H96A**	0.03 ± 0.02	<0.1	-[b]	9.5 ± 0.6	2.6 ± 0.1	6.7 ± 4.2

[a]Metal content is shown (± SD) from three replicate experiments. [b]Not detected.

5. Activity of the DddK$_{Pu}$ wt and the W26G variant

5.1. *Activity of the DddK*$_{\mathrm{Pu}}$ *wt and the W26G variant toward the natural substrate DMSP*

The activities of the DddK$_{Pu}$ wt and the W26G variant toward the natural substrate DMSP was determined using an endpoint activity assay with ten different enzyme concentrations, measured after 15 minutes of reaction. The reactions were carried out in 1.5 mL Eppendorf tubes placed on a vortex mixer set to 1000 rpm and maintained at 25 °C for 15 minutes. Each assay mixture (0.5 mL) contained TEA buffer (50 µL of a 0.5 M stock solution in water pH 8.0, 50 mM in the reaction) (see section 7.1), DSMP (**1**) (100 µL of a 0.5 M stock solution in 50 mM TEA buffer pH 8.0, 1 mmol, 1 eq, 0.1 M in the reaction), plain water (100 µL) and DddK$_{Pu}$ mixture (250 µL of ten different enzyme concentrations ranging from 2.0 µg mL^{-1} to 2.0 mg mL^{-1} in 50 mM TEA buffer pH 8.0).

were added. At the endpoint, samples were withdrawn and analyzed by HPLC as described in HPLC monitoring (see section 1.2.2).

One unit of activity was defined as the amount of DddK$_{Pu}$ required to convert 1 µmol of DMSP to acrylate (**2**) per minute at 30 °C. Measurements were performed in duplicate, with two independent experiments for each condition. The kinetic data were analyzed using GraphPad Prism software (version 5.0) and are reported in **Figure S5**.

5.1. *Activity of the DddK$_{Pu}$ W26G variant toward the natural substrate DMSP in the presence of Ni^{2+}*

The activity of DddK$_{Pu}$ W26G toward the natural substrate DMSP in the presence of Ni^{2+} was determined using an endpoint activity assay with ten different enzyme concentrations, measured after 15 minutes of reaction. The reactions were carried out in 1.5 mL Eppendorf tubes placed on a vortex mixer set to 1000 rpm and maintained at 25 °C for 15 minutes. Each assay mixture (0.5 mL) contained TEA buffer (50 µL of a 0.5 M stock solution in water pH 8.0, 50 mM in the reaction) (see section 7.1), DSMP (**1**) (100 µL of a 0.5 M stock solution in 50 mM TEA buffer pH 8.0, 1 mmol, 1 eq, 0.1 M in the reaction), plain water (100 µL) and 250 µL of a DddK$_{Pu}$ W26G variant and Ni^{2+} mixture. Ten different mixtures of enzyme and Ni^{2+} were prepared, each containing varying concentrations of the enzyme (ranging from 2.0 µg mL^{-1} to 2.0 mg mL^{-1}) and $NiCl_2$ (10 eq) in 50 mM TEA buffer pH 8.0. At the endpoint, samples were withdrawn and analyzed by HPLC as described in HPLC monitoring (see section 1.2.2).

One unit of activity was defined as the amount of DddK$_{Pu}$ required to convert 1 µmol of DMSP to acrylate (**2**) per minute at 30 °C. Measurements were performed in duplicate, with two independent experiments for each condition. The kinetic data were analyzed using GraphPad Prism software (version 5.0) and are reported in **Figure S5**.

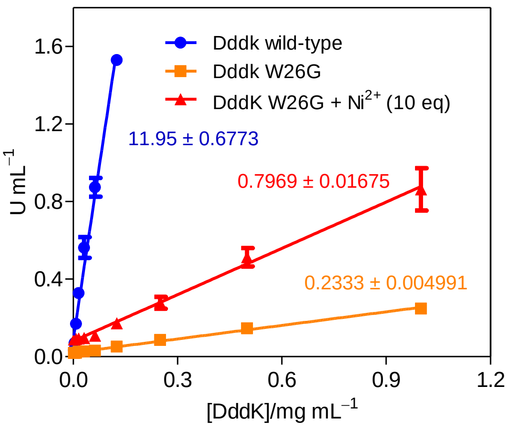

Figure S5. Activity of the DddK*Pu* wild type and the W26G variant toward DMSP with and without the addition of Ni^{2+} (10 eq). Error bars represent the standard error of the mean of two independent experiments under identical reaction conditions.

*5.2. Aza-Michaelase activity of the DddK*Pu* wt toward secondary amines (6)*

The aza-Michaelase activity of DddK*Pu* wt toward secondary amines was determined using an endpoint activity assay with ten different enzyme concentrations. Reaction times were as follows: 15 minutes for most substrates, except **6ak** (30 minutes), **6ad**, **6am**, and **6aq** (2 hours), **6u** (3 hours), and **6ag**, **6ah**, and **6an** (24 hours). The reactions were carried out in 1.5 mL Eppendorf tubes placed on a vortex mixer set to 1000 rpm and maintained at 25 °C for 15 minutes. Each assay mixture (0.5 mL) contained TEA buffer (50 µL of a 0.5 M stock solution in water pH 8.0, 50 mM in the reaction) (see section 7.1), **acrylate** (**2**) (100 µL of a 0.5 M stock solution in 50 mM TEA buffer pH 8.0, 1 mmol, 1 eq, 0.1 M in the reaction), **Michael donor** (**6**) (100 µL of a 0.5 M stock solution prepared as described in Section 7.1 1 mmol, 1 eq, 0.1 M in the reaction), and DddK*Pu* wt mixture (250 µL of ten different enzyme concentrations ranging from 15.0 µg mL^{-1} to 8.0 mg mL^{-1} in 50 mM TEA buffer pH 8.0) (see **Figure S6**, **B**). For substrates **6a-c**, **6i**, **6m** and **6l**, the enzyme concentration range was adjusted from 2.0 µg mL^{-1} to 2.0 mg mL^{-1} (see **Figure S6**, **A**). At the endpoint, samples were withdrawn and analyzed by HPLC as described in HPLC monitoring (see section 1.2.2). One unit of activity was defined as the amount of DddK*Pu* required to catalyze the consumption of 1 µmol of acrylate per minute at 30 °C. Measurements were performed in duplicate, with two independent experiments for each condition. The kinetic data were analyzed using GraphPad Prism software (version 5.0).

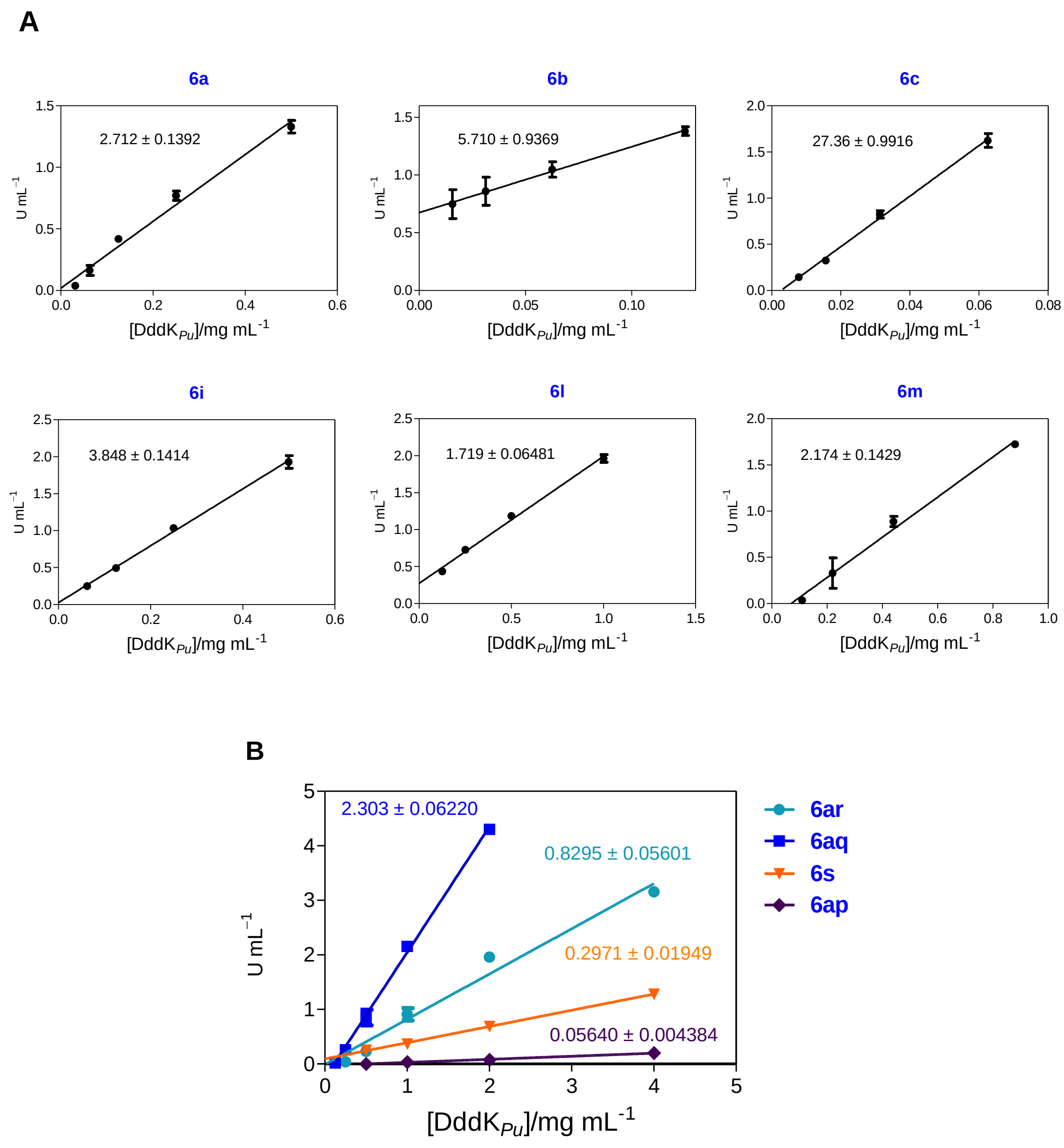

Figure S6. Activity of DddK$_{Pu}$ wild type toward secondary amines (6): A) all secondary amines, with activity determined using a range of enzyme concentrations from 2.0 µg mL^{-1} to 2.0 mg mL^{-1} B) selected examples of secondary amines, with activity determined using a range of enzyme concentrations from 15.0 µg mL^{-1} to 8.0 mg mL^{-1}. Error bars represent the standard error of the mean of two independent experiments under identical reaction conditions.

6. Synthesis of starting material (6p, (*S*)-11)

3-(Benzyloxy)pyrrolidine·hydrochloride (**6p**). Typical procedure: Step I: A dried three-necked round-bottomed flask was charged with anhydrous DMF (90 mL) under a nitrogen atmosphere and cooled to 0 °C. Tert-butyl 3-hydroxypyrrolidine-1-carboxylate (5 g, 26.7 mmol, 1 eq) and sodium hydride (769 mg, 32.0 mmol, 1.2 eq) were added under stirring conditions. After 15-30 minutes, benzyl bromide (1.4 mL, 26.7 mmol, 1

eq) was added dropwise to the reaction mixture. After The reaction was allowed to proceed for 12 h. The reaction was then quenched with water (100 mL) and transferred to an extraction funnel. An additional of 250 mL of water was added, and the product was extracted with ethyl acetate (3 x 100 mL). The combined organic phases were dried over anhydrous $MgSO_4$, filtered, and concentrated under reduced pressure. The crude product was purified by flash column chromatography on silica gel, eluted with a stepwise gradient of hexane/ethyl acetate (100:0, 1L; 98:2, 1L; 95:5, 1L; 90:10, 2,5L). Pure fractions were pooled, and the solvent was removed under vacuum to yield the product as a colorless oil (4.7 g, isolated yield: 64%). The spectral data were consistent with those reported in the literature.[4] ^{1}H NMR (400 MHz, $CDCl_3$) δ 7.40 – 7.25 (m, 5H), 4.59 – 4.45 (m, 2H), 4.15 – 4.09 (m, 1H), 3.59 – 3.34 (br m, 4H), 2.05 (ddt, *J* = 14.1, 8.0, 2 x 3.8 Hz, 1H), 1.97 – 1.92 (m, 1H), 1.46 (s, 9H). ^{13}C NMR (101 MHz, $CDCl_3$) δ 154.9, 137.9, 128.3, 127.5, 127.4, 79.0, 77.7, 70.7, 51.0, 43.7, 30.9, 28.3. **Step II**: The *tert*-butyl 3-(benzyloxy)pyrrolidine-1-carboxylate obtained in Step I (4.7 g, 16.9 mmol, 1 eq) was dissolved in dioxane (112 mL). The reaction was initiated by adding HCl/dioxane (4M, 38 mL, 1 M final concentration of HCl in the reaction). The mixture was stirred for 12 h. The solvent was removed under reduced pressure, and the residue was dissolved in methanol (200 mL). The solvent was again evaporated under reduced pressure, and the operation was repeated, affording the hydrochloride salt of the product as an orange oil (3.6 g, 99% yield). ^{1}H NMR (400 MHz, $CDCl_3$) δ 7.39 – 7.24 (m, 5H), 5.29 (s, 1H), 4.54 (d, *J* = 11.8 Hz, 1H), 4.47 (d, *J* = 11.8 Hz, 1H), 4.25 (tt, *J* = 2 x 4.4, 2 x 1.9Hz, 1H), 3.51 – 3.30 (m, 5H), 2.27 – 2.11 (m, 1H), 2.01 (dddd, *J* = 13.5, 10.6, 8.6, 4.7 Hz, 1H). ^{13}C NMR (101 MHz, $CDCl_3$) δ 137.2, 128.7, 128.1, 127.9, 76.5, 71.3, 49.8, 43.8, 31.0.

(*S*)-piperidin-2-ylmethanamine ((*S*)-**11**). Typical procedure: A 250 mL round bottom flask was charged with (*S*)-piperidine-2-carboxylic acid hydrochloride (10 g, 77.4 mmol, 1 eq) dissolved in anhydrous methanol (150 mL) and cooled to – 20 ºC. Thionyl chloride (5.6 mL, 77.4 mmol, 1 eq) was added dropwise while maintaining the temperature below 0 ºC. The reaction mixture was then warmed to room temperature and refluxed until sulfur dioxide ceased to evolve (2 h). The solution was then concentrated under reduced pressure, and residual thionyl chloride was removed by co-evaporated with dry toluene (2 x 10 mL). The crude product was re-dissolved in methanol (20 mL) and evaporated again to remove traces of toluene. The resulting white

solid was suspended in ethyl acetate (50 mL), and triethylamine (15.0 mL) was added in one portion. The mixture was stirred at room temperature overnight. The precipitate was filtered off and washed with ethyl acetate (2 x 30 mL). The combined filtrates were dried over anhydrous sodium sulfate, filtered, and concentrated under reduced pressure to afford methyl (*S*)-piperidine-2-carboxylate as a pale-yellow oil (8.6 g, 99% yield).

The obtained ester (8.6 g, 60.1 mmol, 1 eq) was dissolved in saturated methanolic ammonia (200 mL) in a 250 mL round-bottomed flask and the resulting mixture was stirred at 20 ºC for 72 h. The solution was then concentrated under reduced pressure to afford (*S*)-piperidine-2-carboxamide as a white solid in quantitative yield.

The amide (7.7 g, 60.0 mmol, 1 eq) was dissolved in dichloromethane (100 mL) and benzyl bromide (8.0 mL, 66.0 mmol, 1.1 eq) was added. The reaction was initiated by the dropwise addition of triethylamine (25.1 mL, 180.2 mmol, 3 eq). The mixture was stirred at 20 ºC for 12 h. The solvent was removed under reduced pressure, and the crude product was purified by flash column chromatography on deactivated silica gel, eluting with a step gradient of hexane/ethyl acetate (50:50, 250 mL, 40:60, 250 mL, 30:70, 250 mL, 20:80, 500 mL). Fractions containing the pure product were combined, and the solvent was evaporated under reduced pressure to afford (*S*)-1-benzylpiperidine-2-carboxamide as a white solid (5.1 g, 40% yield).

The protected amide was reduced using lithium aluminum hydride ($LiAlH_4$) (2.2 g, 58.3 mmol, 2.5 eq), which was previously suspended in anhydrous THF (200 mL) in a 500 mL round-bottom flask at 0 ºC. The amide solution (5.1 g, 23.2 mmol, 1.0 eq) in anhydrous THF (100 mL) was added dropwise at 0 °C, and the mixture was stirred at 20 °C for 12 h. The reaction was then placed in an ice bath and quenched by the slow addition of water (20 mL). The mixture was stirred at 20 ºC for 10 mins, then filtered through a silica pad. The filtrate was washed with THF (1 x 100 mL), and the solvent was removed under reduced pressure. The crude product was re-dissolved in ethyl acetate (100 mL), dried over anhydrous sodium sulfate, filtered, and concentrated under reduced pressure, affording a mixture of the desired product and starting material (4.3 g total). This mixture was subjected to a second reduction following the procedure described above, using fresh $LiAlH_4$ (1.8 g, 49.4 mmol, 2.5 eq), yielding the pure title compound as a yellow oil (3.8 g, 95% yield).

A suspension of the obtained (*S*)-(1-benzylpiperidin-2-yl)methanamine (3.8 g, 18.7 mmol, 1 eq) and 10% palladium on carbon (1g, 944.4 µmol, 0.05 eq) in methanol (80 mL) was hydrogenated at 1 atm at 4 ºC for 12 h. The reaction mixture was filtered

through a Celite pad and a nylon membrane (0.02 µm), and the solid was washed with methanol (3 x 30 mL). The filtrate was concentrated under reduced pressure to afford (*S*)-piperidin-2-ylmethanamine as a yellow oil (1.48 g, 70% yield). The spectral data were consistent with those reported in the literature[5]. $[\alpha]_{20}^{D}$ = – 2.7 (*c* = 1 in MeOH).

7. Screening of aza-Michael addition of primary and secondary amines to acrylic acid catalyzed by DddK$_{Pu}$

7.1. Stock solution preparation

Stock buffer solutions of triethanolamine (TEA, 0.5 M) were prepared at pH 8.0 or 8.5, adjusted with 1 M NaOH. Stock solutions of the primary amines (**4**), **9** and **11** were prepared at a concentration of 0.5 M in TEA buffer (50 mM, pH 8.5, adjusted with 1 M NaOH or HCl) and stored at 4 °C. For these, stock solutions of (*R*)-**4i**, **4m**, *rac*-**4v**, and **4y** included 40% (v/v) DMF, while those of **4b**, **4c**, **4d**, (*S*)-**4i**, **4l**, **4n**, and **4q** contained 50% (v/v) DMF. Stock solutions of the secondary amines (**6**) and **1** were prepared at a concentration of 0.5 M in TEA buffer (50 mM, pH 8.0, adjusted with 1 M NaOH or HCl) and stored at 4 °C. Among these, solutions of **6ao** and **6ap** contained 15% and 30% (v/v) DMF, respectively; those of **6ac** and **6af** contained 40% (v/v) DMF, while solutions of **6n**, **6o**, **6q**, **6r**, **6t**, **6aa**, and **6ab** contained 50% (v/v) DMF. Lastly, stock solutions of **2** were prepared at a concentration of 0.5 M in TEA buffer (50 mM) at pH 8.0 or 8.5, adjusted with 1 M NaOH or HCl, and stored at 4 °C.

*7.2. Aza-Michael addition of **4a-ab** to **2** catalyzed by DddK$_{Pu}$ W26G variant*

A screening of DddK$_{Pu}$ W26G catalyst was conducted to assay the panel of primary amines **4a-ab** as aza-Michael donor substrates and acrylic acid **2** as acceptor, monitoring the depletion of **2** after 24 h (see HPLC monitoring section 1.2.2). Reactions were carried out at analytical level as follows: The reactions (500 µL) were conducted in Eppendorf tubes (1.5 mL) and placed in a vortex mixer (100 rpm) at 25ºC. To a glycerinated preparation of DddK$_{Pu}$ W26G (2 mg protein mL^{-1} in the reaction, from a protein stock solution in 5 mM HEPES buffer pH 7.5, 100 mM NaCl and 50% (v/v) of glycerol), TEA buffer (50 µL of a 0.5 M stock solution in water pH 8.5, 50 mM in the reaction), the primary amines (**4a-ab**, stock solutions prepared as described in Section 7.1 1 mmol, 1 eq, 0.1 M in the reaction), $NiCl_2$ (5 µL of a 0.1 M stock solution, 1 mM in the reaction),

and plain water (to complete the volume till 0.5 mL) were added. The reaction was started by adding acrylic acid (**2**, 100 µL of a 0.5 M stock solution in 50 mM TEA buffer pH 8.5, 1 mmol, 1 eq, 0.1 M in the reaction). Samples were withdrawn immediately after acrylic acid addition (0 h) and after 24 h and analyzed by HPLC as described in HPLC monitoring (see section 1.2.2).

*7.3. Aza-Michael addition of **6a-ar** to **2** catalyzed by DddK$_{Pu}$ wt*

A screening of DddK$_{Pu}$ wt catalyst was conducted to assay the panel of secondary amines **6a-ar** as aza-Michael donor substrates and acrylic acid **2** as acceptor, monitoring the depletion of 2 after 24 h (see HPLC monitoring section 1.2.2). Reactions were carried out at analytical level as follows: The reactions (500 µL) were conducted in Eppendorf tubes (1.5 mL) and placed in a vortex mixer (100 rpm) at 25 ºC. To a glycerinated preparation of DddK$_{Pu}$ wt (2 mg protein mL^{-1} in the reaction, from a protein stock solution in 5 mM HEPES buffer pH 7.5, 100 mM NaCl and 50% (v/v) of glycerol), TEA buffer (50 µL of a 0.5 M stock solution pH 8.0, 50 mM in the reaction), the secondary amines (**6a-ar** stock solutions prepared as described in Section 7.1, 1 mmol, 1 eq, 0.1 M in the reaction), and plain water (to complete the volume till 0.5 mL) were added. The reaction was started by adding acrylic acid (**2**, 100 µL of a 0.5 M stock solution in 50 mM TEA buffer pH 8.0, 1 mmol, 1 eq, 0.1 M in the reaction). Samples were withdrawn immediately after acrylic acid addition (0 h) and after 24 h and analyzed by HPLC as described in HPLC monitoring (see section 1.2.2).

8. Enzymatic synthesis of *N*-substituted and *N,N*-disubstituted-3-aminopropanoate

8.1. Enzymatic synthesis of N-substituted-3-aminopropanoate

Synthesis of **3-(Benzylamino)propanoic acid** (**5g**).

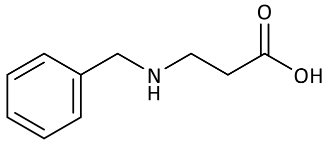

5g

Typical procedure: The reaction (1 mmol scale, 10 mL total volume) was conducted in an Erlenmeyer shake flask (100 mL) at 25 ºC and magnetically stirred at 250 rpm. To a solution of DddK$_{Pu}$ W26G variant (2.9 mL of a 6.9 mg mL^{-1} stock solution in 5 mM HEPES buffer pH 7.5, 100 mM NaCl and 50% (v/v) of glycerol, corresponding to 2 mg protein mL^{-1} in the reaction), plain water (1.0 mL, to complete the volume till 10 mL), TEA buffer (1 mL of a 0.5 M stock solution in water pH 8.5, 50 mM

in the reaction), $NiCl_2$ (100 µL of a 0.1 M stock solution, 10 µmol, 1 mM in the reaction) and acrylic acid **(2)** (2 mL of a 0.5 M stock solution in 50 mM TEA pH 8.5, 1 mmol, 1 eq, 0.1 M in the reaction) were added. The reaction was started by adding benzylamine (**4g**, 3 mL of a stock solution prepared as described in section 7.1, 1.5 mmol, 1.5 eq, 0.15 M in the reaction). The reaction was analyzed by HPLC as described in section 1.2.1. After 24 h, methanol (100 mL) was added and the mixture was filtered through Celite® and a nylon membrane filter of 0.2 µm pore size. The filter cake was washed with methanol (1 x 50 mL) and the organic solvent was removed under vacuum. The aqueous residue was purified by ionic exchange chromatography. The purification was performed on Macro-Prep HighQ (25 mL, BioRad) stationary phase in $OH^–$ form, packed into a glass column (C16/20, GE S11 Healthcare Life Science). The stationary phase was equilibrated with 0.5 M NaOH (4 CV) at 3 mL $min^{–1}$ and water till a neutral pH was reached. The crude fraction (50 mL final volume adjusted with water) was loaded onto the column at 1 mL $min^{–1}$. The column was washed with plain water (10 CV) at 3 mL $min^{–1}$ and the product was eluted isocratically with a solution of 0.5 M formic acid at 3 mL $min^{–1}$. Typical fraction size was 30 mL. Fractions were lyophilized and analyzed by NMR. Fractions of identical composition were pooled affording **5g** as a yellowish oil (174 mg, 77% yield). Retention time on HPLC: t_R = 6.1 (see section 1.2.1). ^{1}H NMR (400 MHz, D_2O) δ 8.35 (br s, 1H), 7.55 – 7.46 (m, 5H), 4.28 (s, 2H), 3.29 (t, *J* = 6.7 Hz, 2H), 2.69 (t, *J* = 6.7 Hz, 2H). ^{13}C NMR (101 MHz, D_2O) δ 176.2, 130.6, 129.7, 129.7, 129.3, 50.9, 43.1, 31.3. ESI-TOF m/z: Calcd for [M+H$^+$] $C_{10}H_{14}NO_2$: 180.1025, found [M+H$^+$]: 180.1020.

3-((Cyclohexylmethyl)amino)propanoic acid (**5e**).

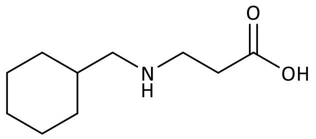

5e

The title compound was prepared as described for **5g**. Starting from **4e** ([**4e**] = [**4g**] = 0.15 M in the reaction mixture), **5e** was obtained as a transparent oil (168 mg, 73% yield). ^{1}H NMR (400 MHz, DMSO) δ 8.21 (s, NH), 2.98 (t, *J* = 7.7 Hz, 2H), 2.70 (d, *J* = 6.8 Hz, 2H), 2.43 (t, 2H), 1.70 (t, *J* = 14.6 Hz, 4H), 1.62 (d, *J* = 10.8 Hz, 3H), 1.17 (p, *J* = 12.3 Hz, 2H), 0.92 (q, *J* = 11.5 Hz, 2H). ^{13}C NMR (101 MHz, DMSO) δ 173.0, 52.5, 43.8, 34.7, 30.8, 30.0, 25.7, 25.1. ESI-TOF m/z: Calcd for [M+H$^+$] $C_{10}H_{20}NO_2$: 186.1494, found [M+H$^+$]: 186.1490.

3-((2,3-Dihydro-1*H*-inden-1-yl)amino)propanoic acid (*rac*-**5h**).

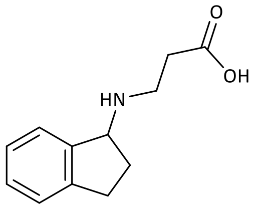

rac-**5h**

The title compound was prepared using a solution of DddK$_{Pu}$ W26G variant (1.5 mL of a 13 mg mL^{-1} stock solution in 5 mM HEPES buffer pH 7.5, 100 mM NaCl and 50% (v/v) of glycerol, corresponding to 2 mg protein mL^{-1} in the reaction mixture) and plain water (2.4 mL), following the procedure described for **5g**. Starting from *rac*-**4h** ([*rac*-**4h**] = [**4g**] = 0.15 M in the reaction mixture), *rac*-**5h** was obtained as a yellowish oil (204 mg, 81% yield). Retention time on HPLC: t_R = 10.3 (see section 1.2.1). ^{1}H NMR (400 MHz, D_2O) δ 8.34 (s, 1H), 7.55 (d, *J* = 7.6 Hz, 1H), 7.48 – 7.42 (m, 2H), 7.36 (ddt, *J* = 7.8, 6.0, 2.6 Hz, 1H), 4.89 (dd, 1H), 3.32 (td, *J* = 6.7, 2.7 Hz, 2H), 3.17 (dt, *J* = 16.0, 7.8 Hz, 1H), 3.02 (ddd, *J* = 16.8, 9.1, 4.2 Hz, 1H), 2.70 (t, *J* = 6.7 Hz, 2H), 2.58 (ddt, *J* = 14.8, 9.1, 7.6 Hz, 1H), 2.29 (ddt, *J* = 14.5, 8.0, 3.9 Hz, 1H). ^{13}C NMR (101 MHz, D_2O) δ 176.0, 167.6, 145.2, 136.1, 130.1, 127.1, 125.5, 125.3, 62.7, 41.0, 31.4, 29.7, 28.2. ESI-TOF m/z: Calcd for [M^+H^+] $C_{12}H_{16}NO_2$: 206.1181, found [M^+H^+]: 206.1178.

(*S*)-3-((1,2,3,4-Tetrahydronaphthalen-1-yl)amino)propanoic acid ((*S*)-**5i**).

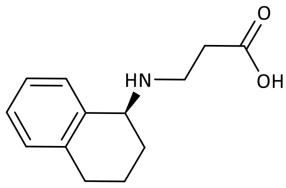

(*S*)-**5i**

The title compound was prepared using a solution of DddK$_{Pu}$ W26G variant (2.4 mL of a 8.2 mg mL^{-1} stock solution in 5 mM HEPES buffer pH 7.5, 100 mM NaCl and 50% (v/v) of glycerol, corresponding to 2 mg protein mL^{-1} in the reaction mixture) and plain water (2 mL), following the procedure described for **5g**. The reaction was initiated by adding (*S*)-**4i** (2 mL of a 0.5 M stock solution in 50 mM TEA H 8.5, 1.0 mmol, 1.0 eq, 0.1 M in the reaction mixture). (*S*)-**5i** was obtained as an orangish powder (168 mg, 63% yield). Retention time on HPLC: t_R = 11.6 (see section 1.2.1). ^{1}H NMR (400 MHz, D_2O) δ 8.46 (br s, 1H), 7.43 – 7.36 (m, 2H), 7.35 – 7.27 (m, 2H), 4.53 (t, *J* = 4.7 Hz, 1H), 3.36 (dt, 1H), 3.31 (dt, *J* = 6.4 Hz, 1H), 2.92 (dt, 1H), 2.82 (dt, 1H), 2.62 (t, *J* = 6.5 Hz, 2H), 2.22 (dq, *J* = 9.5, 4.9 Hz, 1H), 2.13 (ddt, *J* = 14.7, 9.8, 5.0 Hz, 1H), 1.97 – 1.82 (m, 2H). ^{13}C NMR (101 MHz, D_2O) δ 177.8, 138.7, 129.9, 129.7, 129.4, 129.4, 126.4, 55.8, 41.9, 31.7, 27.7, 24.5, 17.4. ESI-TOF m/z: Calcd for [$M+H^+$] $C_{13}H_{18}NO_2$: 220.1338, found [$M+H^+$]: 220.1332.

(*R*)-3-((1,2,3,4-Tetrahydronaphthalen-1-yl)amino)propanoic acid ((*R*)-**5i**).

The title compound was prepared using a solution of DddK*Pu* W26G variant (1.5 mL of a 13 mg mL^{-1} stock solution in 5 mM HEPES buffer pH 7.5, 100 mM NaCl and 50% (v/v) of glycerol, corresponding to 2 mg protein mL^{-1} in the reaction mixture) and plain water (3.4 mL), following the procedure described for **5g**. The reaction was started by adding (*R*)-**4i** (2 mL of a 0.5 M stock solution in 50 mM TEA pH 8.5, 1.0 mmol, 1.0 eq, 0.1 M in the reaction mixture). (*R*)-**5i** was obtained as a orangish powder (123 mg, 46% yield). Retention time on HPLC: t_R = 10.3 (see section 1.2.1). ^{1}H NMR (400 MHz, D_2O) δ 8.40 (s, 1-NH), 7.45 – 7.36 (m, 2H), 7.36 – 7.27 (m, 2H), 4.54 (t, *J* = 4.7 Hz, 1H), 3.34 (dp, *J* = 25.4, 6.3 Hz, 2H), 2.93 (dt, *J* = 17.4, 5.3 Hz, 1H), 2.88 – 2.77 (m, 1H), 2.64 (t, *J* = 6.6 Hz, 2H), 2.17 (dtd, *J* = 29.6, 9.8, 5.0 Hz, 2H), 1.97 – 1.84 (m, 2H). ^{13}C NMR (101 MHz, D_2O) δ 177.5, 138.7, 129.9, 129.7, 129.4, 129.4, 126.4, 55.8, 41.8, 31.5, 27.7, 24.5, 17.4. ESI-TOF m/z: Calcd for [M+H$^+$] $C_{13}H_{18}NO_2$: 220.1338, found [M+H$^+$]: 220.1333.

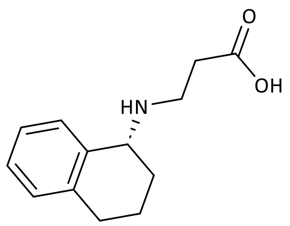

(*R*)-**5i**

(*S*)-3-((1-Phenylethyl)amino)propanoic acid ((*S*)-**5j**).

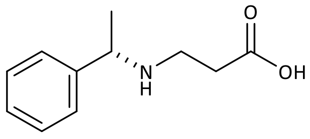

(*S*)-**5j**

The title compound was prepared as described for **5g**. Starting from (*S*)-**4j** ([(*S*)-**4j**] = [**4g**] = 0.15 M in the reaction mixture), (*S*)-**5j** was obtained as a yellowish oil (193 mg, 80% yield). Retention time on HPLC: t_R = 9.6 (see section 1.2.1). ^{1}H NMR (400 MHz, D_2O) δ 8.37 (br s, 1H), 7.58 – 7.45 (m, 5H), 4.45 (q, *J* = 6.9 Hz, 1H), 3.19 (dt, *J* = 13.0, 6.5 Hz, 1H), 3.06 (dt, *J* = 12.8, 6.9 Hz, 1H), 2.61 (t, *J* = 6.7 Hz, 2H), 1.70 (d, *J* = 6.9 Hz, 3H). ^{13}C NMR (101 MHz, D_2O) δ 176.3, 135.7, 129.7, 129.4, 127.5, 58.3, 41.8, 31.4, 18.2. ESI-TOF m/z: Calcd for [M+H$^+$] $C_{11}H_{16}NO_2$: 194.1181, found [M+H$^+$]: 194.1175.

(*R*)-3-((1-Phenylethyl)amino)propanoic acid ((*R*)-**5j**).

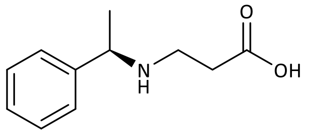

(*R*)-**5j**

The title compound was prepared starting from (*R*)-**4j** ([(*R*)-**4j**] = [**4g**] = 0.15 M in the reaction mixture) as described for **5g**. (*R*)-**5j** was obtained as a yellowish oil (154 mg, 65% yield). Retention time on HPLC: t_R = 9.9 (see section 1.2.1). ^{1}H NMR (400 MHz, D_2O) δ 7.57 – 7.45 (m, 5H), 4.44 (q, *J* = 6.9 Hz, 1H), 3.16 (dt, *J* = 13.0, 6.6 Hz, 1H), 3.03 (dt, *J* = 12.7, 6.9 Hz, 1H), 2.55 (t, *J* = 6.7 Hz, 2H), 1.69 (d, *J* = 6.9 Hz, 3H). ^{13}C NMR (101 MHz, D_2O) δ 177.1, 135.8, 129.7, 129.4, 127.5, 58.2, 42.1, 31.9, 18.2. ESI-TOF m/z: Calcd for [M+H$^+$] $C_{11}H_{16}NO_2$: 194.1181, found [M+H$^+$]: 194.1178.

3-((Thiophen-2-ylmethyl)amino)propanoic acid (**5o**).

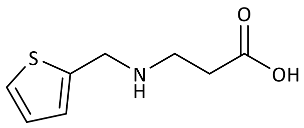

5o

The title compound was prepared using a solution of DddK*Pu* W26G variant (2.4 mL of a 8.2 mg mL^{-1} stock solution in 5 mM HEPES buffer pH 7.5, 100 mM NaCl and 50% (v/v) of glycerol, corresponding to 2 mg protein mL^{-1} in the reaction mixture), and plain water (1.5 mL), following the procedure described for **5g**. The reaction was started by adding **4o** ([**4o**] = [**4g**] = 0.15 M in the reaction mixture). The product was purified using a gradient elution from 0% to 100% 0.5 M formic acid over 80 minutes at a flow rate of 3 mL min^{-1}. **5o** was obtained as a yellow oil (152 mg, 66% yield). Retention time on HPLC: t_R = 6.6 (see section 1.2.1). ^{1}H NMR (400 MHz, D_2O) δ 8.39 (s, 1H), 7.58 (dd, *J* = 5.2, 1.0 Hz, 1H), 7.30 (dd, 1H), 7.14 (dd, *J* = 5.2, 3.5 Hz, 1H), 4.51 (s, 2H), 3.29 (t, *J* = 6.7 Hz, 2H), 2.65 (t, *J* = 6.7 Hz, 2H). ^{13}C NMR (101 MHz, D_2O) δ 176.8, 131.4, 130.9, 128.7, 127.8, 44.7, 43.0, 31.7. ESI-TOF m/z: Calcd for [M+H^+] $C_8H_{12}NO_2S$: 186.0589, found [M+H^+]: 186.0583.

3-((((3r,5r,7r)-Adamantan-1-yl)methyl)amino)propanoic acid (**5aa**).

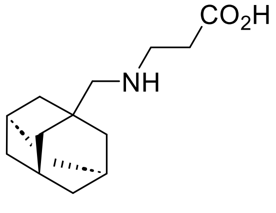

5aa

The title compound was prepared starting from **4aa** ([**4aa**] = [**4g**] = 0.15 M in the reaction mixture) as described for **5g**. The product was purified using a gradient elution from 0% to 100% 0.5 M formic acid over 80 minutes at a flow rate of 3 mL min^{-1}. **5aa** was obtained as a white powder (186 mg, 66% yield). ^{1}H NMR (400 MHz, D_2O) δ 3.22 (t, *J* = 6.6 Hz, 2H), 2.77 (s, 2H), 2.60 (t, *J* = 6.6 Hz, 2H), 2.05 – 1.98 (m, 3H), 1.75 (d, *J* = 11.5 Hz, 3H), 1.67 (d, *J* = 11.5 Hz, 3H), 1.59 (d, *J* = 2.9 Hz, 6H). ^{13}C NMR (101 MHz, D_2O) δ 178.6, 58.8, 45.3, 39.0, 35.8, 31.6, 31.4, 27.6. ESI-TOF m/z: Calcd for [M+H^+] $C_{14}H_{23}NO_2$: 238.1807, found [M+H^+]: 238.1805.

3-(((3s,5s,7s)-Adamantan-1-yl)amino)propanoic acid (**5ab**).

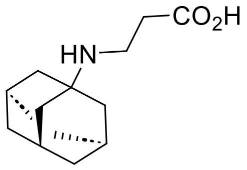

5ab

The title compound was prepared using a solution of DddK*Pu* W26G variant (2.3 mL of a 13 mg mL^{-1} stock solution in 5 mM HEPES buffer pH 7.5, 100 mM NaCl and 50% (v/v) of glycerol, corresponding to 3 mg protein mL^{-1} in the reaction mixture), and plain water (1.6 mL), following the procedure described for **5g**. The reaction was initiated by adding **4ab** ([**4ab**] = [**4g**] = 0.15 M in the reaction mixture). The product was purified using a gradient elution from 0% to 100% 0.5 M formic acid over 80 minutes at a flow rate of 3 mL min^{-1}. **5ab** was obtained as a white powder (116 mg,

43% yield). ^{1}H NMR (400 MHz, D_2O) δ 8.4 (br s, 1H), 3.3 (t, *J* = 6.7 Hz, 2H), 2.6 (t, *J* = 6.7 Hz, 2H), 2.2 (t, *J* = 3.4 Hz, 3H), 1.9 (d, *J* = 3.0 Hz, 6H), 1.8 (d, *J* = 3.3 Hz, 3H), 1.7 (d, *J* = 3.3 Hz, 3H). ^{13}C NMR (101 MHz, D_2O) δ 176.4, 57.5, 38.0, 35.9, 34.9, 31.8, 28.8. ESI-TOF m/z: Calcd for [M+H$^+$] $C_{13}H_{21}NO_2$: 224.1651, found [M+H$^+$]: 224.1648.

8.2. Enzymatic synthesis of N,N-disubstituted-β-aminoacids

Synthesis of **3-(pyrrolidin-1-yl)propanoic acid** (**7c**). Typical procedure: the reaction (1 mmol scale, 10 mL total volume) was conducted in a Erlenmeyer shake flask (100 mL) at 25 ºC and magnetically stirred at 250 rpm. To a solution of DddK*Pu* wt (2.5 mL of a 7.8 mg mL^{-1} stock solution in 5 mM HEPES buffer pH 7.5, 100 mM NaCl and 50% (v/v) of glycerol, 2 mg protein mL^{-1} in the reaction), plain water (1.5 mL, to complete the volume till 10 mL), TEA buffer (1 mL of a 0.5 M stock solution in water pH 8.0, 50 mM in the reaction) and acrylic acid **(2)** (2 mL of a 0.5 M stock solution in 50 mM TEA/HCl pH 8.0, 1 mmol, 1 eq, 0.1 M in the reaction) were added. The reaction was started by adding pyrrolidine (**6c**, 3 mL of a 0.5 M stock solution in 50 mM TEA/HCl pH 8.0, 1.5 mmol, 1.5 eq, 0.15 M in the reaction). The reaction was analyzed by HPLC as described in section 1.2.1. After 24 h, methanol (100 mL) was added and the mixture was filtered through Celite® and a nylon membrane filter of 0.2 µm pore size. The filter cake was washed with methanol (1 x 50 mL) and the organic solvent was removed under vacuum. The aqueous residue was purified by ionic exchange chromatography. The purification was performed on Macro-Prep HighQ (25 mL, BioRad) stationary phase in OH$^-$ form, packed into a glass column (C16/20, GE S11 Healthcare Life Science). The stationary phase was equilibrated with 0.5 M NaOH (4 CV) at 3 mL min^{-1} and water till a neutral pH was reached. The crude fraction (50 mL final volume adjusted with water) was loaded onto the column at 1 mL min^{-1}. The column was washed with plain water (10 CV) at 3 mL min^{-1} and the product was eluted isocratically with a solution of 0.5 M formic acid at 3 mL min^{-1}. Typical fraction size was 30 mL. Fractions were lyophilized and analyzed by NMR. Fractions of identical composition were pooled affording **6c** as a yellow powder (143 mg, 75% yield). ^{1}H NMR (400 MHz, $CDCl_3$) δ 3.2 (q, *J* = 6.3 Hz, 6H), 2.6 (t, *J* = 6.3 Hz, 2H), 2.1 – 2.0 (m, 4H).^{13}C NMR (101 MHz, $CDCl_3$) δ 53.8, 52.7, 23.7. ESI-TOF m/z: Calcd for [M+H$^+$] $C_7H_{14}NO_2$: 144.1025, found [M+H$^+$]: 144.1024.

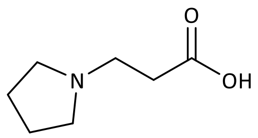

7c

3-(Dimethylamino)propanoic acid (**7a**).

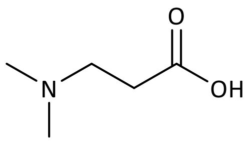

7a

The title compound was prepared as described for **7c**. Starting from **6a** ([**6a**] = [**6c**] = 0.15 M in the reaction mixture), **7a** was obtained as a yellowish oil (94 mg, 60% yield). ^{1}H NMR (400 MHz, $CDCl_3$) δ 3.1 (t, *J* = 6.4 Hz, 2H), 2.7 (s, 6H), 2.6 (t, *J* = 6.4 Hz, 2H). ^{13}C NMR (101 MHz, $CDCl_3$) δ 31.7, 55.2, 43.6, 43.6, 174.4. ESI-TOF m/z: Calcd for [M+H$^+$] $C_5H_{11}NO_2$: 118.0868, found [M+H$^+$]: 118.0864.

3-(Azetidin-1-yl)propanoic acid (**7b**).

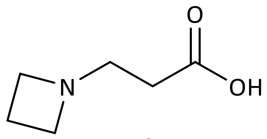

7b

The title compound was prepared using a solution of DddK$_{Pu}$ wt (2.4 mL of a 8.3 mg mL^{-1} stock solution in 5 mM HEPES buffer pH 7.5, 100 mM NaCl and 50% (v/v) of glycerol, corresponding to 2 mg protein mL^{-1} in the reaction) and plain water (1.6 mL), following the procedure described for **7c**. Starting from **6b** ([**6b**] = [**6c**] = 0.15 M in the reaction mixture), **7b** was obtained as a white oil (117 mg, 67% yield). ^{1}H NMR (400 MHz, $CDCl_3$) δ 4.0 (t, *J* = 8.1 Hz, 4H), 3.3 (t, *J* = 6.3 Hz, 2H), 2.5 (t, *J* = 6.3 Hz, 2H), 2.4 (d, *J* = 8.0 Hz,2H). ^{13}C NMR (101 MHz, $CDCl_3$) δ 175.6, 53.9, 52.7, 31.8, 16.2. ESI-TOF m/z: Calcd for [M+H$^+$]: $C_6H_{12}NO_2$: 130.0868, found [M+H$^+$]: 130.0861.

(*R*)-3-(2-(Hydroxymethyl)pyrrolidin-1-yl)propanoic acid (**7d**).

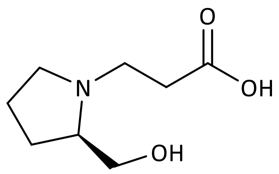

7d

The title compound was prepared using a solution of DddK$_{Pu}$ wt (2.4 mL of a 8.3 mg mL^{-1} stock solution in 5 mM HEPES buffer pH 7.5, 100 mM NaCl and 50% (v/v) of glycerol, corresponding to 2 mg protein mL^{-1} in the reaction mixture) and plain water (1.6 mL), following the procedure described for **7c**. Starting from **6d** ([**6d**] = [**6c**] = 0.15 M in the reaction), **7d** was obtained as a yellow oil (151 mg, 69% yield). ^{1}H NMR (400 MHz, $CDCl_3$) δ 3.95 (dd, *J* = 13.1, 3.0 Hz, 1H), 3.84 (s, 2H), 3.76 (dt, *J* = 13.3, 7.0 Hz, 1H), 3.43 (qd, *J* = 7.0, 2.9 Hz, 1H), 3.11 (dt, *J* = 12.5, 5.5 Hz, 1H), 2.90 (dt, *J* = 11.1, 7.5 Hz, 1H), 2.64 (td, *J* = 12.2, 6.6 Hz, 2H), 2.09 (ddt, *J* = 11.2, 9.3, 2.7 Hz, 2H), 1.98 (ddt, 2H). ^{13}C NMR (101 MHz, $CDCl_3$) δ 175.4, 70.7, 60.5, 55.0, 54.0, 53.9, 32.8, 26.4, 23.6. ESI-TOF m/z: Calcd for [M+H$^+$] $C_8H_{16}NO_3$: 174.1130, found [M+H$^+$]: 174.1126.

3-(Piperidin-1-yl)propanoic acid (**7g**).

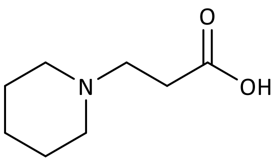

7g

The title compound was prepared as described for **7c.** Starting from **6g** ([**6g**] = [**6c**] = 0.15 M in the reaction mixture), **7g** was obtained as a yellow oil (158 mg, 78% yield). ^{1}H NMR (400 MHz, $CDCl_3$) δ 3.1 (t, *J* = 6.5 Hz, 6H), 2.6 (t, *J* = 6.6 Hz, 4H), 1.8 (p, *J* = 5.8 Hz, 1H), 1.6 (s, 2H). ^{13}C NMR (101 MHz, $CDCl_3$) δ 174.5, 53.9, 53.2, 30.2, 23.6, 22.6. ESI-TOF m/z: Calcd for [M+H$^+$] $C_8H_{16}NO_2$: 158.1181, found [M+H$^+$]: 158.1176.

3-(Azepan-1-yl)propanoic acid (**7i**).

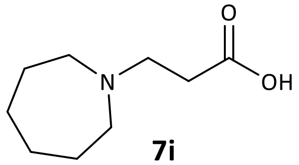

7i

The title compound was prepared as described for **7c.** Starting from **6i** ([**6i**] = [**6c**] = 0.15 M in the reaction mixture), **7i** was obtained as a yellow oil (160 mg, 94% yield). ^{1}H NMR (400 MHz, $CDCl_3$) δ 3.2 (dt, *J* = 14.8, 6.0 Hz, 6H), 2.6 (t, *J* = 6.6 Hz, 2H), 1.9 (h, *J* = 4.3 Hz, 4H), 1.7 (p, *J* = 2.8 Hz, 4H). ^{13}C NMR (101 MHz, $CDCl_3$) δ 174.5, 54.6, 54.2, 30.6, 27.2, 24.4. ESI-TOF m/z: Calcd for [M+H$^+$] $C_9H_{17}NO_2$: 172.1338, found [M+H$^+$]: 172.1332.

3-Morpholinopropanoic acid (**7j**).

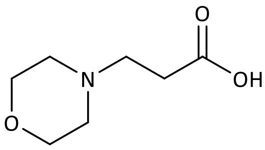

7j

The title compound was prepared as described for **7c**. Starting from **6j** ([**6j**] = [**6c**] = 0.15 M in the reaction mixture), **7j** was obtained as a white powder (102 mg, 50% yield). ^{1}H NMR (400 MHz, MeOD) δ 3.87 – 3.80 (m, 4H), 3.17 (t, *J* = 6.8 Hz, 2H), 3.09 (t, *J* = 4.8 Hz, 4H), 2.60 (t, *J* = 6.8 Hz, 2H). ^{13}C NMR (101 MHz, MeOD) δ 176.2, 65.9, 53.2, 55.1, 30,7. ESI-TOF m/z: Calcd for [M+H$^+$] $C_7H_{14}NO_3$: 160.0974, found [M+H$^+$]: 160.0968.

3-Thiomorpholinopropanoic acid (**7k**).

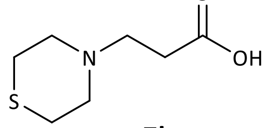

7k

The title compound was prepared as described for **7c**. Starting from **6k** ([**6k**] = [**6c**] = 0.15 M in the reaction mixture), **7k** was obtained as a yellowish oil (113 mg, 64% yield). ^{1}H NMR (400 MHz, $CDCl_3$) δ 3.19 (dd, *J* = 6.7, 3.6 Hz, 4H), 3.06 (t, *J* = 6.5 Hz, 2H), 2.91 – 2.84 (m, 4H), 2.62 (t, *J* = 6.5 Hz, 2H). ^{13}C NMR (101 MHz, $CDCl_3$) δ 174.1, 54.2, 54.1, 30.0, 26.1. ESI-TOF m/z: Calcd for [M+H$^+$] $C_7H_{14}NO_2S$: 176.0745, found [M+H$^+$]: 176.0741.

3-((1*R*)-3-Hydroxy-8-azabicyclo[3.2.1]octan-8-yl)propanoic acid (**7l**).

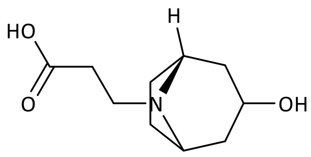

7l

The title compound was prepared as described for **7c**. Starting from **6l** ([**6l**] = [**6c**] = 0.15 M in the reaction mixture), **7l** was obtained as a white powder (195 mg, 80% yield). ^{1}H NMR (400 MHz, D_2O) δ 4.15 (t, *J* = 4.8 Hz, 1H), 4.00 (dq, *J* = 5.9, 2.9 Hz, 2H), 3.26 (t, *J* = 7.0 Hz, 2H), 2.72 (t, *J* = 7.0 Hz, 2H), 2.50 – 2.36 (m, 2H), 2.34 – 2.14 (m, 4H), 2.10 (d, *J* = 16.0 Hz, 2H). ^{13}C NMR (101 MHz, D_2O) δ 176.5, 61.6, 61.3, 48.1, 36.9, 30.6, 23.7. ESI-TOF m/z: Calcd for [M+H$^+$] $C_{10}H_{18}NO_3$: 200.1287, found [M+H$^+$]: 200.1281.

3-(((1*R*,2*S*)-1-Hydroxy-1-phenylpropan-2-yl)(methyl)amino)propanoic acid. (**7m**).

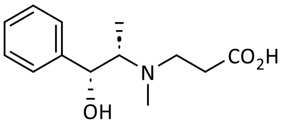

7m

The title compound was prepared starting from **6m** ([**6m**] = [**6c**] = 0.15 M in the reaction mixture), using a solution of DddK*Pu* wt (2.4 mL of a 8.3 mg mL^{-1} stock solution in 5 mM HEPES buffer pH 7.5, 100 mM NaCl and 50% (v/v) of glycerol, corresponding to 2 mg protein mL^{-1} in the reaction) and plain water (1.6 mL), according to the procedure described for **7c**. The product was purified using a gradient elution from 0% to 100% 0.5 M formic acid over 80 minutes at a flow rate of 3 mL min^{-1}. **7m** was obtained as a transparent oil (244 mg, 86% yield). Retention time on HPLC: t_R = 10.2 (see section 1.2.1). ^{1}H NMR (400 MHz, MeOD) δ 7.5 – 7.4 (m, 4H), 7.3 – 7.3 (m, 1H), 5.3 (d, *J* = 2.5 Hz, 1H), 3.6 (qt, *J* = 6.9, 4.1 Hz, 2H), 3.4 (dt, *J* = 12.8, 6.2 Hz, 1H), 3.0 (s, 3H), 2.8 – 2.6 (m, 2H), 1.2 (d, *J* = 6.9 Hz, 3H). ^{13}C NMR (101 MHz, MeOD) δ 176.4, 142.4, 129.6, 129.0, 127.0, 72.4, 67.4, 52.6, 38.0, 30.3, 7.2. ESI-TOF m/z: Calcd for [M+H$^+$] $C_{13}H_{20}NO_3$: 238.1443, found [M+H$^+$]: 238.1440.

3-(3-(Benzyloxy)pyrrolidin-1-yl)propanoic acid (**7p**).

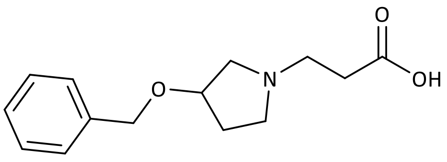

7p

The title compound was prepared using a solution of DddK*Pu* wt (2.4 mL of a 8.3 mg mL^{-1} stock solution in 5 mM HEPES buffer pH 7.5, 100 mM NaCl and 50% (v/v) of glycerol, corresponding to 2 mg protein mL^{-1} in the reaction mixture) and plain water (1.6 mL), following the procedure described for **7c**. Starting from **6p** ([**6p**] = [**6c**] = 0.15 M in the reaction), **7p** was obtained as a light yellow oil (274 mg, 93% yield). Retention time on HPLC: t_R = 12.6 (see section 1.2.1). ^{1}H NMR (400 MHz, $CDCl_3$) δ 7.35 – 7.20 (m, 5H), 4.50 – 4.39 (m, 2H), 4.24 (q, *J* = 3.9 Hz, 1H), 3.72 (dd, *J* = 12.6, 5.0

Hz, 1H), 3.65 – 3.56 (m, 1H), 3.30 (t, J = 6.3 Hz, 2H), 3.13 (t, J = 9.5 Hz, 2H), 2.60 (t, J = 6.4 Hz, 2H), 2.15 (dq, J = 9.2, 4.2 Hz, 2H). ^{13}C NMR (101 MHz, $CDCl_3$) δ 174.8, 137.3, 128.8, 128.3, 127.9, 71.5, 58.9, 53.4, 52.7, 32.4, 30.8. ESI-TOF m/z: Calcd for [M+H$^+$] $C_{14}H_{20}NO_3$: 250.1443, found [M+H$^+$]: 250.1438.

3-(3,4-Dihydroisoquinolin-2(1H)-yl)propanoic acid (**7s**).

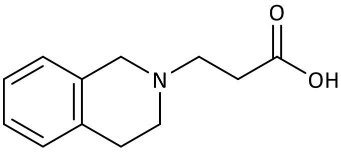

7s

The title compound was prepared following the procedure described for **7c**, starting from **6s** ([**6s**] = [**6c**] = 0.15 M in the reaction mixture). The product was purified using a gradient elution from 0% to 100% 0.5 M formic acid over 80 minutes at a flow rate of 3 mL min^{-1}. **7s** was obtained as a yellow oil (230 mg, 92% yield). Retention time on HPLC: t_R = 5.7 (see section 1.2.1). ^{1}H NMR (400 MHz, $CDCl_3$) δ 7.21 (p, J = 1.8 Hz, 2H), 7.14 (d, 1H), 7.07 (d, 1H), 4.17 (s, 2H), 3.30 (t, J = 6.2 Hz, 2H), 3.22 (t, J = 6.5 Hz, 2H), 3.07 (t, J = 6.2 Hz, 2H), 2.70 (t, J = 6.5 Hz, 2H). ^{13}C NMR (101 MHz, $CDCl_3$) δ 174.3, 131.5, 129.0, 128.9, 127.9, 127.1, 127.0, 53.2, 51.8, 49.2, 30.5, 25.7. ESI-TOF m/z: Calcd for [M+H$^+$] $C_{12}H_{16}NO_2$: 206.1181, found [M+H$^+$]: 206.1176.

3-(1H-Imidazol-1-yl)propanoic acid (**7u**).

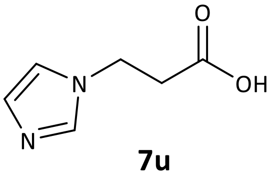

7u

The title compound was prepared using a solution of DddK$_{Pu}$ wt (2.4 mL of a 8.3 mg mL^{-1} stock solution in 5 mM HEPES buffer pH 7.5, 100 mM NaCl and 50% (v/v) of glycerol, corresponding to 2 mg protein mL^{-1} in the reaction mixture) and plain water (1.6 mL), following the procedure described for **7c**. Starting from **6u** ([**6u**] = [**6c**] = 0.15 M in the reaction mixture), **7u** was obtained as a white powder (133 mg, 71% yield). ^{1}H NMR (400 MHz, D_2O) δ 8.72 (s, 1H), 7.52 (s, 1H), 7.44 (s, 1H), 4.50 – 4.38 (m, 2H), 2.80 (t, J = 6.4 Hz, 2H). ^{13}C NMR (101 MHz, D2O) δ 177.4, 134.6, 121.7, 119.6, 46.2, 37.0. ESI-TOF m/z: Calcd for [M+H$^+$] $C_6H_9N_2O_2$: 141.0664, found [M+H$^+$]: 141.0659.

3-(2,5-Dihydro-1H-pyrrol-1-yl)propanoic acid (**7ad**).

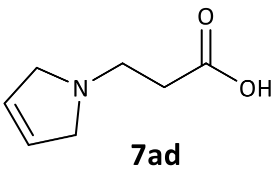

7ad

The title compound was prepared using a solution of DddK$_{Pu}$ wt (2.4 mL of a 8.3 mg mL^{-1} stock solution in 5 mM HEPES buffer pH 7.5, 100 mM NaCl and 50% (v/v) of glycerol, 2 mg protein mL^{-1} in the reaction) and plain water (1.6 mL), following the procedure described for **7c**. Starting from **6ad** ([**6ad**] = [**6c**] = 0.15 M in the reaction mixture), **7ad** was obtained as a brownish oil (170 mg, 90% yield). ^{1}H NMR (400 MHz,

CDCl3) δ 5.84 (s, 2H), 4.02 (s, 4H), 3.33 (t, J = 6.3 Hz, 2H), 2.63 (t, J = 6.3 Hz, 2H). ^{13}C NMR (101 MHz, CDCl3) δ 174.8, 125.5, 59.4, 52.7, 32.1. ESI-TOF m/z: Calcd for [M+H$^+$] $C_7H_{12}NO_2$: 142.0868, found [M+H$^+$]: 142.0863.

3-(Thiazolidin-3-yl)propanoic acid (**7ag**).

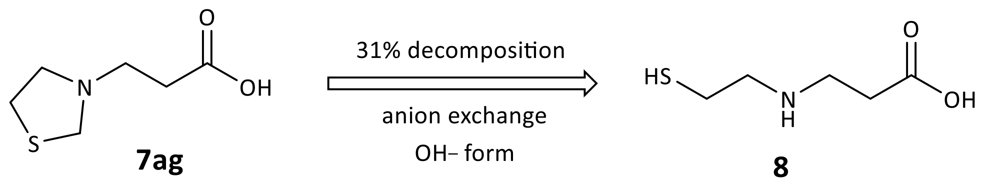

The title compound was prepared using a solution of DddK$_{Pu}$ wt (2.4 mL of a 8.3 mg mL^{-1} stock solution in 5 mM HEPES buffer pH 7.5, 100 mM NaCl and 50% (v/v) of glycerol, corresponding to 2 mg protein mL^{-1} in the reaction) and plain water (1.6 mL), following the procedure described for **7c**. Starting from **6ag** ([**6ag**] = [**6c**] = 0.15 M in the reaction mixture), **7ag** was obtained in a mixture with its degraded form **8** as a yellowish oil (**7ag**:**8** 69:31, 168 mg). **7ag**, 69%. ^{1}H NMR (400 MHz, MeOD) δ 4.01 (s, 2H), 3.08 (t, J = 6.4 Hz, 2H), 2.88 (t, J = 6.4 Hz, 2H), 2.73 (t, J = 7.0 Hz, 2H), 2.48 (t, J = 7.0 Hz, 2H). ^{13}C NMR (101 MHz, MeOD) δ 175.8, 60.0, 58.5, 49.8, 34.6, 29.7. **8**, 31%. ^{1}H NMR (400 MHz, MeOD) δ 3.34 (t, J = 6.7 Hz, 2H), 3.23 (t, J = 6.3 Hz, 2H), 3.03 (dt, J = 6.8 Hz, 2H), 2.61 (t, J = 6.3 Hz, 2H). ^{13}C NMR (101 MHz, MeOD) δ176.7, 47.0, 45.4, 35.0, 31.9, 29.7. ESI-TOF m/z: Calcd for [M+H$^+$] $C_6H_{12}NO_2S$: 162.0589 for **7ag**, found [M+H$^+$]: 162.0586. ESI-TOF m/z: Calcd for [M+H$^+$] $C_5H_{12}NO_2S$ for **8**: 150.0589, found [M+H$^+$]: 150.0591.

3-((2-Cyanoethyl)(cyclopropyl)amino)propanoic acid (**7ah**).

The title compound was prepared using a solution of DddK$_{Pu}$ wt (2.4 mL of a 8.3 mg mL^{-1} stock solution 5 mM HEPES buffer pH 7.5, 100 mM NaCl and 50% (v/v) of glycerol,

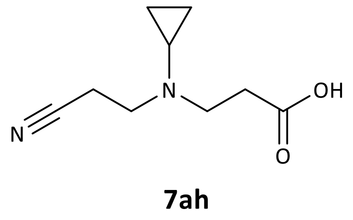

corresponding to 2 mg protein mL^{-1} in the reaction) and plain water (2.6 mL), according to the procedure described for **7c**. The reaction was started by adding **6ah** (2 mL of a 0.5 M stock solution in 50 mM TEA/HCl pH 8.0, 1.0 mmol, 1.0 eq, 0.1 M in the reaction mixture). The reaction time was extended to 48h. The product was purified using a gradient elution from 0% to 100% 0.5 M formic acid over 80 minutes at a flow rate of 3 mL min^{-1}. **7ah** was obtained as a light orange powder (90 mg, 40% yield).^{1}H NMR (400 MHz, CDCl3) δ 3.00 (t, J = 6.9 Hz, 4H), 2.59 (t, J = 6.9 Hz, 4H), 1.87 (tt, J = 6.5, 3.9 Hz, 1H), 0.66 – 0.52 (m, 4H). ^{13}C NMR (101 MHz, CDCl3) δ 176.4,

118.7, 50.9, 50.8, 36.0, 31.9, 15.8, 6.8. ESI-TOF m/z: Calcd for [M+H$^+$] $C_9H_{15}N_2O_2$:183.1134, found [M+H$^+$]: 183.1136/183.1130.

3-(3-Hydroxyazetidin-1-yl)propanoic acid (**7aj**).

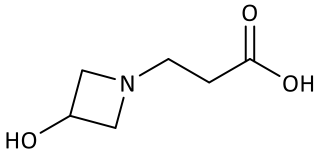

7aj

The title compound was prepared using a solution of DddK*Pu* wt (2.4 mL of a 8.3 mg mL^{-1} stock solution in 5 mM HEPES buffer pH 7.5, 100 mM NaCl and 50% (v/v) of glycerol, 2 mg protein mL^{-1} in the reaction) and plain water (1.6 mL), following the procedure described for **7c**. Starting from **6aj** ([**6aj**] = [**6c**] = 0.15 M in the reaction mixture), **7aj** was obtained as a white powder (138 mg, 72% yield). ^{1}H NMR (400 MHz, MeOD) δ 4.61 (tt, *J* = 6.9, 5.7 Hz, 1H), 4.41 – 4.31 (m, 2H), 3.94 – 3.85 (m, 2H), 3.37 (t, *J* = 6.4 Hz, 2H), 2.47 (t, *J* = 6.4 Hz, 2H). ^{13}C NMR (101 MHz, MeOD) δ 176.4, 64.5, 60.9, 54.1, 31.8. ESI-TOF m/z: Calcd for [M+H$^+$] $C_6H_{12}NO_3$: 146.0817, found [M+H$^+$]: 146.0811.

3-(3,3-Difluoroazetidin-1-yl)propanoic acid (**7ak**).

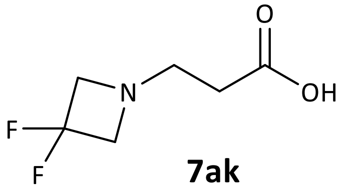

7ak

The title compound was prepared using a solution of DddK*Pu* wt (2.4 mL of a 8.3 mg mL^{-1} stock solution in 5 mM HEPES buffer pH 7.5, 100 mM NaCl and 50% (v/v) of glycerol, corresponding to 2 mg protein mL^{-1} in the reaction) and plain water (2.6 mL), following the procedure described for **7c**. Starting from **6ak** ([**6ak**] = [**6c**] = 0.15 M in the reaction mixture), **7ak** was obtained as a white oil (116 mg, 55% yield). ^{1}H NMR (400 MHz, $CDCl_3$) δ 3.87 (t, *J* = 11.8 Hz, 4H), 3.05 (t, *J* = 6.6 Hz, 2H), 2.47 (t, *J* = 6.4 Hz, 2H). ^{13}C NMR (101 MHz, $CDCl_3$) δ 174.3, 115.0, 64.4, 53.8, 32.4. ESI-TOF m/z: Calcd for [M+H$^+$] $C_6H_{10}F_2NO_2$: 166.0680, found [M+H$^+$]: 166.0674.

3-(3,3-Difluoropyrrolidin-1-yl)propanoic acid (**7al**).

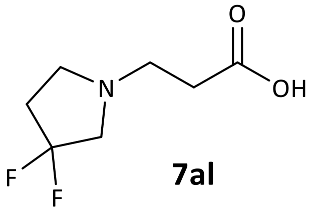

7al

The title compound was prepared using a solution of DddK*Pu* wt (2.4 mL of a 8.3 mg mL^{-1} stock solution in 5 mM HEPES buffer pH 7.5, 100 mM NaCl and 50% (v/v) of glycerol, corresponding to 2 mg protein mL^{-1} in the reaction) and plain water (1.6 mL), following the procedure described for **7c**. Starting from **6al** ([**6al**] = [**6c**] = 0.15 M in the reaction mixture), **7al** was obtained as a yellowish oil (125 mg, 55% yield). ^{1}H NMR (400 MHz, $CDCl_3$) δ 3.17 (t, *J* = 12.9 Hz, 2H), 3.00 (d, *J* = 29.1 Hz, 4H), 2.55 (t, *J* = 6.4 Hz, 2H), 2.38 (tt, *J* = 14.1, 7.0 Hz, 2H).

^{13}C NMR (101 MHz, $CDCl_3$) δ 175.0, 128.3, 61.0, 52.0, 51.7, 35.7, 32.6. ESI-TOF m/z: Calcd for [M+H$^+$] $C_7H_{12}F_2NO_2$: 180.0836, found [M+H$^+$]: 180.0832.

3-((2-Mercaptoethyl)amino)propanoic acid (**7am**).

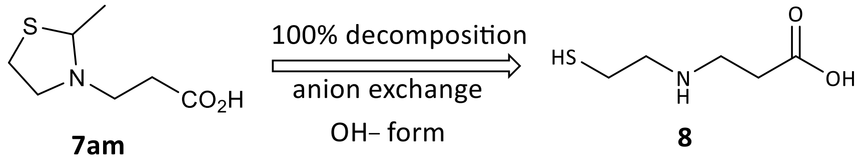

7am **8**

The title compound was prepared using a solution of DddK*Pu* wt (2.4 mL of a 8.3 mg mL^{-1} stock solution in 5 mM HEPES buffer pH 7.5, 100 mM NaCl and 50% (v/v) of glycerol, corresponding to 2 mg protein mL^{-1} in the reaction) and plain water (1.6 mL), following the procedure described for **7c**. Starting from **6am** ([**6am**] = [**6c**] = 0.15 M in the reaction mixture), **8** was the only detected product as a brown oil (195 mg, 88% yield). ^{1}H NMR (400 MHz, MeOD) δ 3.37 (t, *J* = 6.6 Hz, 2H), 3.28 – 3.18 (m, 2H), 3.07 (t, *J* = 6.6 Hz, 2H), 2.58 (t, *J* = 6.2 Hz, 2H). ^{13}C NMR (101 MHz, MeOD) δ 177.0, 46.6, 45.4, 34.8, 32.0. ESI-TOF m/z: Calcd for [M+H$^+$] $C_5H_{12}NO_2S$: 150.0589, found [M+H$^+$]: 150.0590.

(*S*)-3-(2-(Trifluoromethyl)pyrrolidin-1-yl)propanoic acid (**7an**).

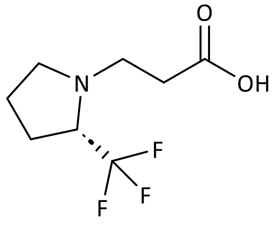

7an

The title compound was prepared starting from **6an** ([**6an**] = [**6c**] = 0.15 M in the reaction mixture) using a solution of DddK*Pu* wt (2.4 mL of a 8.3 mg mL^{-1} stock solution in 5 mM HEPES buffer pH 7.5, 100 mM NaCl and 50% (v/v) of glycerol, corresponding to 2 mg protein mL^{-1} in the reaction) and plain water (1.6 mL), according to the procedure described for **7c**. The reaction time was extended to 48h. The product was purified using a gradient elution from 0% to 100% 0.5M formic acid over 80 minutes at a flow rate of 3 mL min^{-1}. **7an** was obtained as a transparent oil (84 mg, 33% yield). ^{1}H NMR (400 MHz, MeOD) δ 3.31 – 3.20 (m, 1H), 3.23 – 3.11 (m, 2H), 2.85 (ddd, *J* = 12.9, 7.6, 6.2 Hz, 1H), 2.51 (t, 2H), 2.45 (d, *J* = 0.0 Hz, 1H), 2.02 (dtd, *J* = 13.2, 10.2, 7.9 Hz, 1H), 1.95 – 1.71 (m, 3H). ^{13}C NMR (101 MHz, MeOD) δ 176.0, 129.8, 127.0, 65.4, 55.0, 52.9, 27.1, 25.4. ESI-TOF m/z: Calcd for [M+H$^+$] $C_8H_{13}F_3NO_2$: 212.0895, found [M+H$^+$]: 212.0898.

3-(Benzyl(2-(benzylamino)ethyl)amino)propanoic acid (**7ap**).

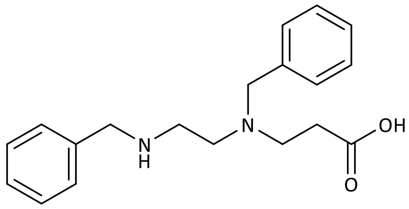

7ap

The title compound was prepared using a solution of DddK$_{Pu}$ wt (2.4 mL of an 8.3 mg mL^{-1} stock solution in 5 mM HEPES buffer pH 7.5, 100 mM NaCl and 50% (v/v) of glycerol, 2 mg protein mL^{-1} in the reaction) and plain water (2.6 mL), according to the procedure described for **7c**. The reaction was initiated by adding **6ap** (2 mL of a 0.5 M stock solution in 50 mM TEA/HCl pH 8.0, 1.0 mmol, 1.0 eq, 0.1 M in the reaction mixture). The product was purified using a gradient elution from 0% to 100% 0.5 M formic acid over 80 minutes at a flow rate of 3 mL min^{-1}. **7ap** was obtained as a white powder (298 mg, 83% yield). Retention time on HPLC: t_R = 12.0 (see section 1.2.1). ^{1}H NMR (400 MHz, $CDCl_3$) δ 7.32 – 7.21 (m, 6H), 7.15 (dd, *J* = 6.6, 2.8 Hz, 4H), 3.54 (s, 4H), 2.89 (t, *J* = 5.8 Hz, 2H), 2.64 – 2.56 (m, 4H), 2.53 (t, *J* = 5.2 Hz, 2H). ^{13}C NMR (101 MHz, $CDCl_3$) δ 178.5, 137.7, 131.1, 129.9, 129.8, 129.1, 129.1, 128.7, 127.9, 58.4, 48.3, 48.2, 47.5, 41.9, 33.8. ESI-TOF m/z: Calcd for [M+H$^+$] $C_{19}H_{25}N_2O_2$: 313.1916, found [M+H$^+$]: 313.1917.

3-(Hydroxy(methyl)amino)propanoic acid (**7aq**).

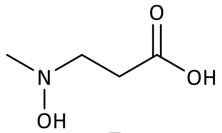

7aq

The title compound was prepared using a solution of DddK$_{Pu}$ wt (2.4 mL of an 8.3 mg mL^{-1} stock solution in 5 mM HEPES buffer pH 7.5, 100 mM NaCl and 50% (v/v) of glycerol, 2 mg protein mL^{-1} in the reaction) and plain water (1.6 mL), following the procedure described for **7c**. Starting from **6aq** ([**6aq**] = [**6c**] = 0.15 M in the reaction mixture), **7aq** was obtained as a brown oil (50 mg, 30% yield). ^{1}H NMR (400 MHz, D_2O) δ 3.20 (dt, *J* = 14.3, 6.7 Hz, 2H), 2.74 (s, 3H), 2.59 – 2.51 (m, 2H). ^{13}C NMR (101 MHz, D_2O) δ 178.4, 45.9, 33.5, 32.6. ESI-TOF m/z: Calcd for [M+H$^+$] $C_4H_{10}NO_3$: 120.0661, found [M+H$^+$]: 120.0659.

3-(3-Azabicyclo[3.1.0]hexan-3-yl)propanoic acid (**7ar**).

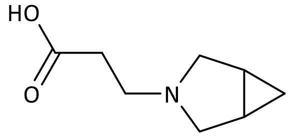

7ar

The title compound was prepared using a solution of DddK$_{Pu}$ wt (2.4 mL of a 8.3 mg mL^{-1} stock solution in 5 mM HEPES buffer pH 7.5, 100 mM NaCl and 50% (v/v) of glycerol, corresponding to 2 mg protein mL^{-1} in the reaction) and plain water (1.6 mL), following the procedure described for **7c**. Starting from **6ar** ([**6ar**] = [**6c**] = 0.15 M in the reaction mixture), **7ar** was obtained as a yellowish oil (129 mg, 64% yield). ^{1}H NMR (400 MHz, D_2O) δ 3.71 (d, *J* = 11.3 Hz, 2H), 3.44 – 3.33 (m, 4H), 2.63 (t, *J* = 7.2 Hz,

2H), 1.85 (dt, J = 7.6, 3.5 Hz, 2H), 0.86 (q, J = 7.7 Hz, 1H), 0.49 (dt, J = 7.1, 4.3 Hz, 1H). ^{13}C NMR (101 MHz, D_2O) δ 176.8, 55.6, 51.4, 32.1, 14.4, 6.4. ESI-TOF m/z: Calcd for [M+H$^+$] $C_8H_{14}NO_2$: 156.1025, found [M+H$^+$]: 156.1019.

8.3. Enzymatic synthesis of 3-((piperidin-2-ylmethyl)amino)propanoic acid and 3-(2-(aminomethyl)pyrrolidin-1-yl)propanoic acid

(*rac*)-3-((Piperidin-2-ylmethyl)amino)propanoic acid ((*rac*)-**12**).

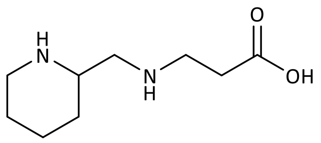

(*rac*)-**12**

The title compound was prepared starting from *rac*-**11** ([*rac*-**11**] = [**4g**] = 0.15 M in the reaction mixture) as described for **5g**. The product was purified using a gradient elution from 0% to 100% 0.5 M formic acid over 80 minutes at a flow rate of 3 mL min^{-1}. (*rac*)-**12** was obtained as a dark orange oil (118 mg, 50% yield). ^{1}H NMR (400 MHz, D_2O) δ 8.44 (br s, 1H), 3.66 – 3.55 (m, 1H), 3.53 – 3.46 (m, 1H), 3.46 – 3.19 (m, 4H), 3.07 (td, J = 12.6, 3.0 Hz, 1H), 2.61 (t, J = 6.5 Hz, 2H), 2.15 – 2.04 (m, 1H), 1.99 – 1.85 (m, 2H), 1.77 – 1.51 (m, 3H). ^{13}C NMR (101 MHz, D_2O) δ 177.8, 53.0, 48.7, 45.3, 45.1, 31.9, 26.2, 21.3, 20.9. $[\alpha]_{20}^{D}$ = 0 (c = 1 in MeOH). ESI-TOF m/z: Calcd for [M+H$^+$] $C_9H_{19}N_2O_2$: 187.1447, found [M+H$^+$]: 187.1443.

(*R*)-3-(2-(Aminomethyl)pyrrolidin-1-yl)propanoic acid ((*R*)-**10**).

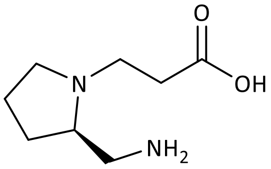

(*R*)-**10**

The title compound was prepared starting with (*R*)-**9** ([(*R*)-**9**] = [**6c**] = 0.15 M in the reaction mixture) using a solution of DddK$_{Pu}$ wt (1.2 mL of a 16.7 mg mL^{-1} stock solution in 5 mM HEPES buffer pH 7.5, 100 mM NaCl and 50% (v/v) of glycerol, corresponding to 2 mg protein mL^{-1} in the reaction) and plain water (2.8 mL), according to the procedure described for **7c**. The purification was performed on Macro-Prep HighQ (150 mL, BioRad) stationary phase in OH$^-$ form (24 cm of length), packed into a glass column (C26/40, GE S11 Healthcare Life Science). The stationary phase was equilibrated with 0.5 M NaOH (4 CV) at 6 mL min^{-1} and water till a neutral pH was reached. The crude fraction (50 mL final volume adjusted with water) was loaded onto the column at 2 mL min^{-1}. The column was washed with plain water (10 CV) at 6 mL min^{-1} and the product was eluted isocratically with a solution of 0.5 M formic acid at 6 mL min^{-1}. (*R*)-**10** was obtained as a yellowish oil (167 mg, 76% yield). ^{1}H NMR (400 MHz, D_2O) δ 3.8 (qd, J = 8.16, 5.28 Hz, 1H), 3.8 (dt, J = 11.78, 7.16 Hz, 1H), 3.6 (ddd, J = 14.48, 8.51, 6.19 Hz, 2H), 3.5 – 3.3 (m, 3H), 2.7 (dd, J = 7.23, 5.53

Hz, 2H), 2.5 (dtd, J = 13.24, 7.64, 5.43 Hz, 1H), 2.3 – 2.1 (m, 2H), 1.9 (dq, J = 13.30, 8.16 Hz, 1H). ^{13}C NMR (101 MHz, D_2O) δ 177.6, 65.0, 54.5, 52.5, 39.2, 31.9, 28.0, 21.9. ESI-TOF m/z: Calcd for [M+H$^+$] $C_8H_{15}NO_2$: 156.1022, found [M+H$^+$]: 156.1026.

The (*S*)-enantiomer, (*S*)-**10** was prepared starting from (*S*)-**9** following the same procedure described above (62 mg, 35% yield). NMR spectra were indistinguishable from that of (*R*)-**10**. Similarly, NMR spectra of (*R*)-**10** and (*S*)-**10** synthesized by DddK$_{Pu}$ W26G variant were identical to the same products obtained by DddK$_{Pu}$ wt catalyst with the procedure described above ((*R*)-**10**: 128 mg, 59% yield, (*S*)-**10**: 164 mg, 75% yield).

(*S*)-3-((Piperidin-2-ylmethyl)amino)propanoic acid ((*S*)-**12**).

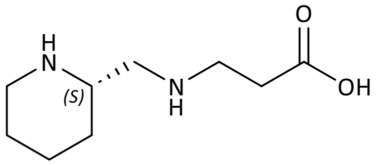

(*S*)-**12**

The title compound was prepared using a solution of DddK$_{Pu}$ W26G variant (3.5 mL of an 8.4 mg mL^{-1} stock solution in 5 mM HEPES buffer pH 7.5, 100 mM NaCl and 50% (v/v) of glycerol, corresponding to 3 mg protein mL^{-1} in the reaction mixture) and plain water (1.4 mL), following the procedure described for **5g**. The reaction was initiated by adding (*S*)-**11** ([(*S*)-**11**] = [**2**] = 0.1 M in the reaction mixture) (2 mL of a 0.5 M stock solution in 50 mM TEA/HCl pH 8.5, 1.0 mmol, 1.0 eq). The purification was performed on Macro-Prep HighQ (150 mL, BioRad) stationary phase in OH$^-$ form (24 cm of length), packed into a glass column (C26/40, GE S11 Healthcare Life Science). The stationary phase was equilibrated with 0.5 M NaOH (4 CV) at 6 mL min^{-1} and water till a neutral pH was reached. The crude fraction (50 mL final volume adjusted with water) was loaded onto the column at 2 mL min^{-1}. The column was washed with plain water (10 CV) at 6 mL min^{-1}, and the product was purified using a gradient elution from 20% to 100% 0.5 M formic acid over 60 minutes, followed by isocratic elution with 0.5 M formic acid, all at a flow rate of 6 mL min^{-1} (*S*)-**12** was obtained as an orange oil (119 mg, 51% yield). ^{1}H NMR (400 MHz, D_2O) δ 8.4 (s, 1H), 7.5 (s, 1H), 3.6 – 3.5 (m, 1H), 3.5 (d, J = 12.87 Hz, 1H), 3.5 – 3.2 (m, 4H), 3.1 (td, J = 12.55, 2.82 Hz, 1H), 2.6 (t, J = 6.36 Hz, 2H), 2.1 (d, J = 9.75 Hz, 1H), 2.0 – 1.9 (m, 2H), 1.6 (dt, J = 22.03, 12.64 Hz, 4H). ^{13}C NMR (101 MHz, D_2O) δ 177.9, 53.0, 48.7, 45.3, 45.1, 31.9, 26.2, 21.3, 20.9. $[\alpha]_{20}^{D}$ = +16.9 (c = 1 in MeOH). ESI-TOF m/z: Calcd for [M+H$^+$] $C_9H_{19}N_2O_2$: 187.1447, found [M+H$^+$]: 187.1443.

9. Computational Methods.

Protein complexes were modeled with the package Schrödinger Suite 2024-2,[6] through its graphical interface Maestro.[7] The reported crystal structure of wild-type DddK*Pu* complexed with a molecule of diacrylate in its active site (PDB 5TFZ[1]) was prepared using the Protein Preparation Wizard[8] included in Maestro for removing solvent molecules and ions, adding hydrogens, setting protonation states[9] and running a restrained minimization using the OPLS4 force-field.[10] The structure of variant W26G was generated from that using the Mutate Residue option without further modifications. The structures of selected compounds (substrates **2**, **6e** and **6h**, and aza-Michael adducts **5aa**, **7a**, **7c** **7g**, **7i**, **7l**, **7m** and **7p**) (Figure S7) were built within Maestro and energy optimized with MacroModel[11] using the same force field and GB/SA water solvation conditions.[12] These compounds were docked into the active site of chain A of the proteins with Glide,[13,14] using the diacrylate molecule to center the receptor grid and generating 10 poses for each ligand. Some of the complexes, i.e. those of adducts **7c**, **7l** and **5aa** (Figure 3 of main text), were then energy optimized using the QM/MM program QSite,[15-17] previously replacing the Ni(II) cation present in the crystal structure by Zn(II), which is the metal most abundant in our enzyme preparations. The QM/MM boundary was defined by placement of hydrogen caps between the Cα and Cβ atoms of the residues that coordinate the metal cation and those with side-chains closer to the ligands, ie. Y21, W26, L53, H56, H58, E62, Y64, H96, F108, W110 and Y122, for the wild-type enzyme and the same except residue 26 for the W26G variant. In addition, the ligands and the Zn(II) cation were also part of the QM region. Optimizations were accomplished using the hybrid B3LYP functional with the LACVP* basis for the metal and the 6-31G** basis for the rest of the QM atoms. The rest of the residues of the protein with atoms within 5 Å of the ligands were MM optimized using the OPLS4 force-field, and those beyond that limit were kept frozen. The complexes were ranked according to their QM/MM energies.

The reaction mechanism of the aza-Michael addition of the simplest amine **6a** to acrylate (**2**) catalyzed by DddK$_{Pu}$ was studied following the cluster methodology as reported by Wang and coworkers.[18] Briefly, the active site of DddK$_{Pu}$ was represented by only considering the side chains, including the α-carbon atoms, of residues His56, His58, Tyr64, Glu62, His96 and Tyr122, plus the metal cation, that in this case was Zn(II). Given that DMSP is analogous to adduct **7a** and DMS to **6a**, we used the reported coordinates

of the active-site model plus DMSP, the corresponding transition state of the cleavage reaction, and of the complex with the products of the cleavage reaction[18] as starting point to model the three stationary points of the reaction coordinate with our compounds, by simply replacing the S atom by NH. Then, the three stationary points were energy optimized using Jaguar[19-21] with the hybrid functional B3LYP-D3, that includes Grimme corrections for dispersion.[22-24] For geometry optimizations, the LANL2DZ basis was used for Zn^{2+}, 6-311+G** for the reactive nitrogen and the 6-31+G** for the rest of atoms of the reagents (**6a** and **2**), and 6-31G** for the atoms of the aminoacids. During the optimizations, the α-carbon atoms of the six amino acids included in the models where fixed in space to preserve the steric arrangement of the enzyme. Then, the optimized geometries were used to recalculate more accurate energies using single point calculations using larger basis sets, LANL2TZ for Zn^{2+} and cc-pVTZ(-f)+ for the rest of the atoms, and solvation effects using the CPCM model as implemented in Jaguar. Vibrational frequencies were calculated at the same level of theory to identify the nature of the stationary points and determine Zero-Point energies (ZPE). All complexes were characterized as minima (no imaginary frequencies) or transition state (a single imaginary frequency).

Table S7. DFT calculated energies for the stationary points determined along the reaction coordinate of the aza-Michael reaction between **6a** and **2**.

	Gas phase En. (Hartree)	Solvation En. (kcal mol^{-1})	ZPE (298.15 K) (kcal mol^{-1})	G (kcal mol^{-1})	Total G (Hartree)
Reagents	-2462.329918	-29.08	608.727	-55.077905	-2461.493956
TS	-2462.298543	-34.61	609.296	-53.524153	-2461.468015
Product	-2462.331112	-35.52	612.982	-52.998005	-2461.495330

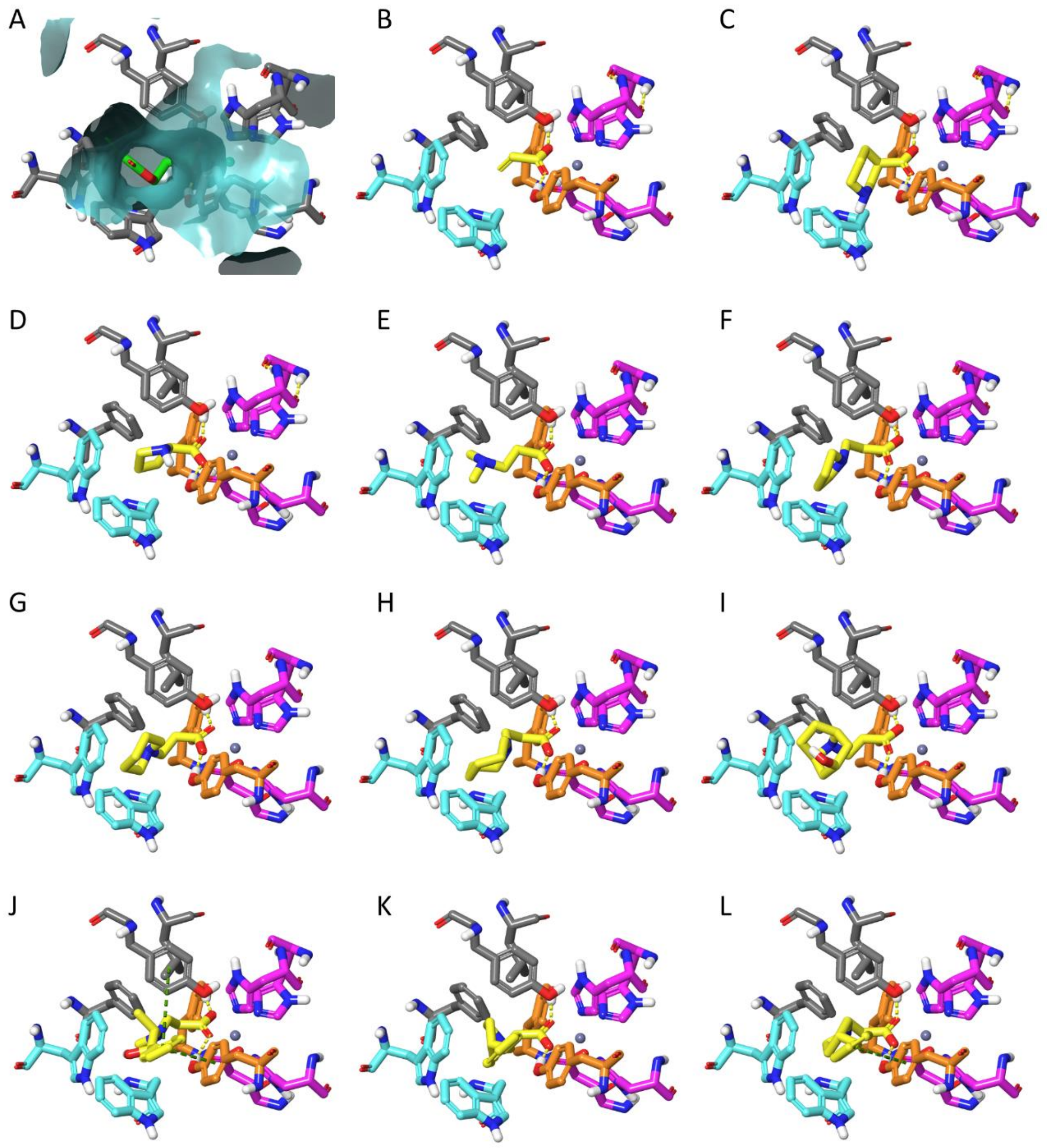

Figure S7. (A) Diacrylate bound DddK$_{Pu}$ active site cavity (PDB 5TFZ) showing the access channel that allows entrance/exit of substrates and products. (B-L) Best docked poses determined for acrylate **2** (B), amines **6e** (C) and **6h** (D), and adducts **7a** (E), **7c** (F), **7g** (G), **7i** (H), **7l** (I), **7m** (J), **(S)-7p** (K) and **(R)-7p** (L). Ligands are shown with yellow sticks and residues are colored as indicated in Figure 3 of main the text.

10. NMR Spectra.

a)

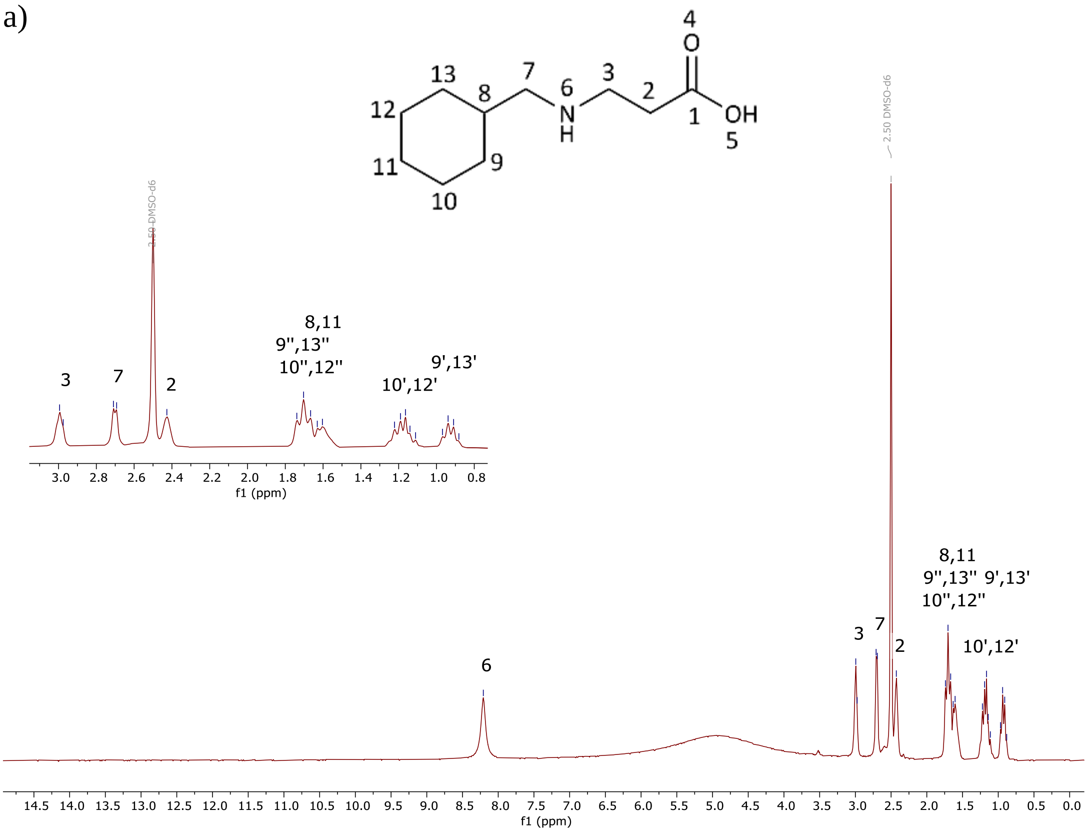

b)

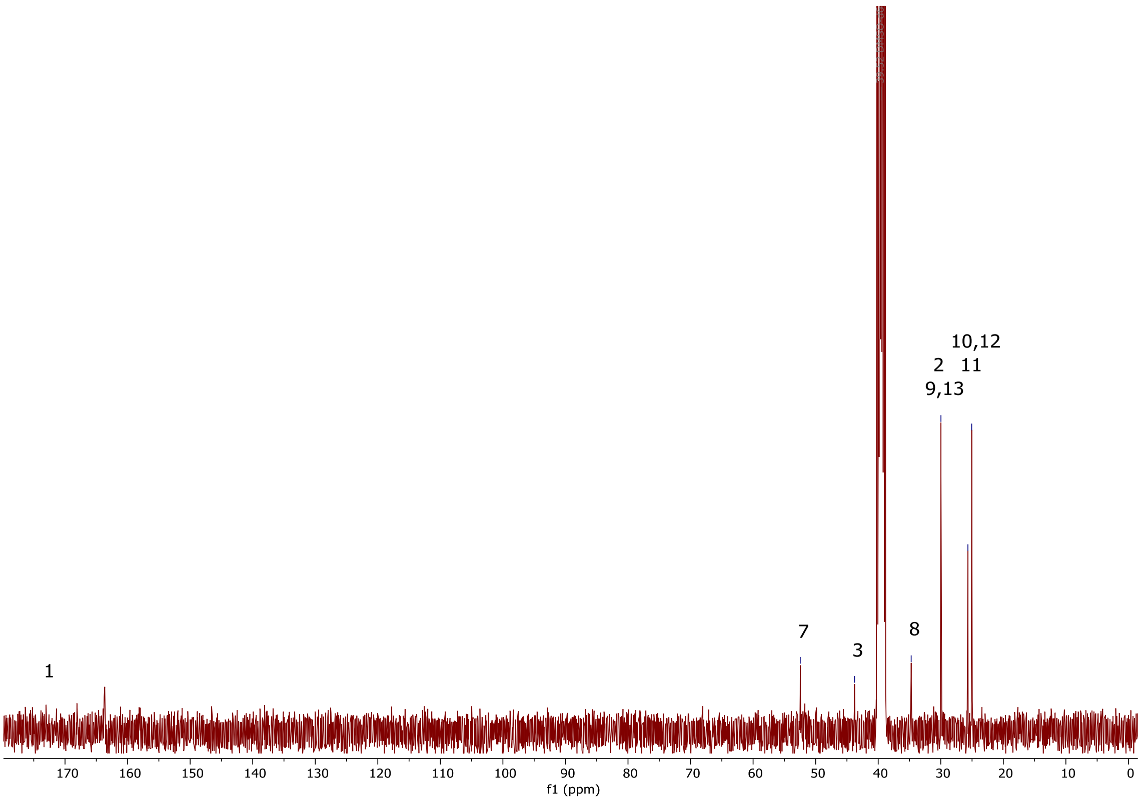

c)

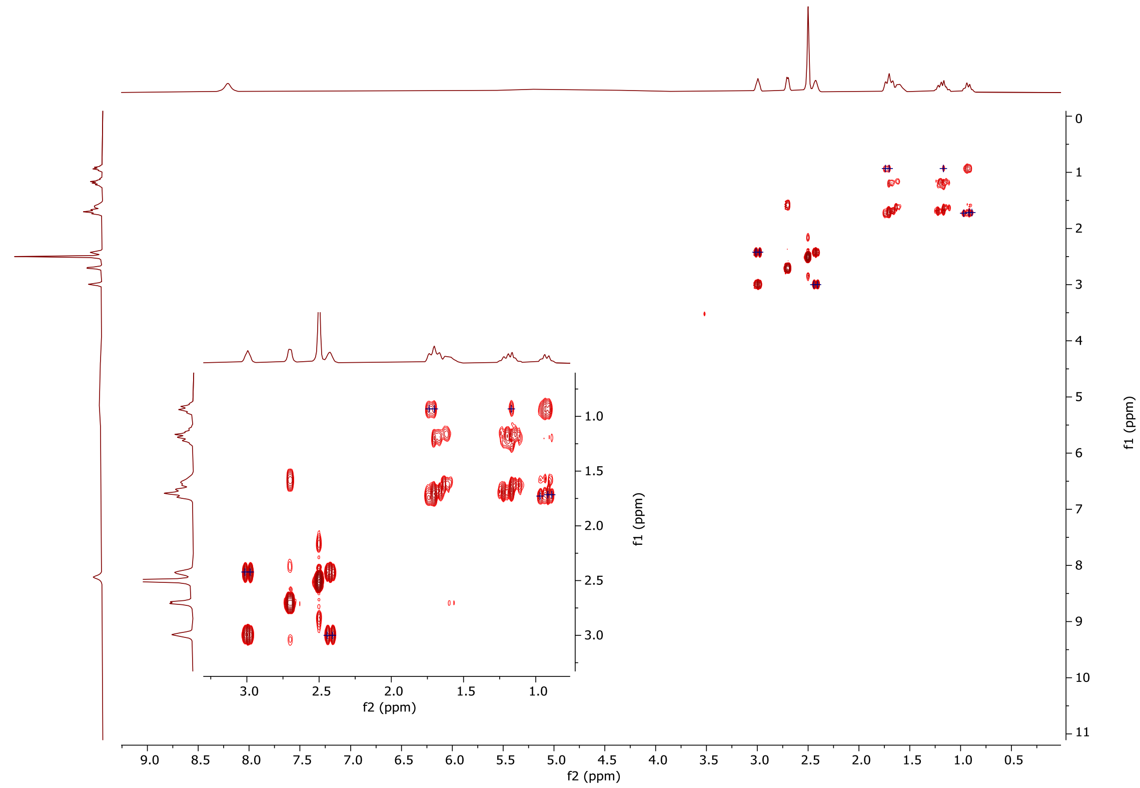

9.0 8.5 8.0 7.5 7.0 6.5 6.0 5.5 5.0 4.5 4.0 3.5 3.0 2.5 2.0 1.5 1.0 0.5
f2 (ppm)
0 1 2 3 4 5 6 7 8 9 10 11
f1 (ppm)
3.0 2.5 2.0 1.5 1.0
f2 (ppm)
1.0 1.5 2.0 2.5 3.0
f1 (ppm)

d)

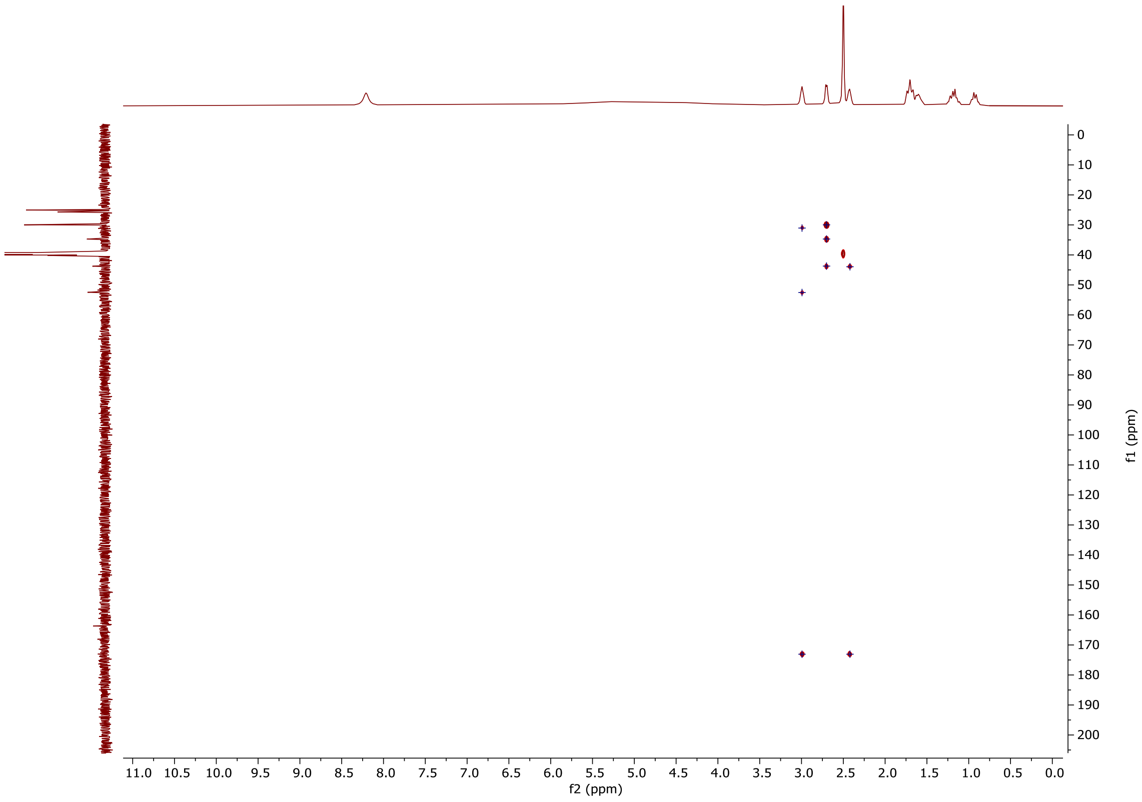

11.0 10.5 10.0 9.5 9.0 8.5 8.0 7.5 7.0 6.5 6.0 5.5 5.0 4.5 4.0 3.5 3.0 2.5 2.0 1.5 1.0 0.5 0.0
f2 (ppm)
0 10 20 30 40 50 60 70 80 90 100 110 120 130 140 150 160 170 180 190 200
f1 (ppm)

e)

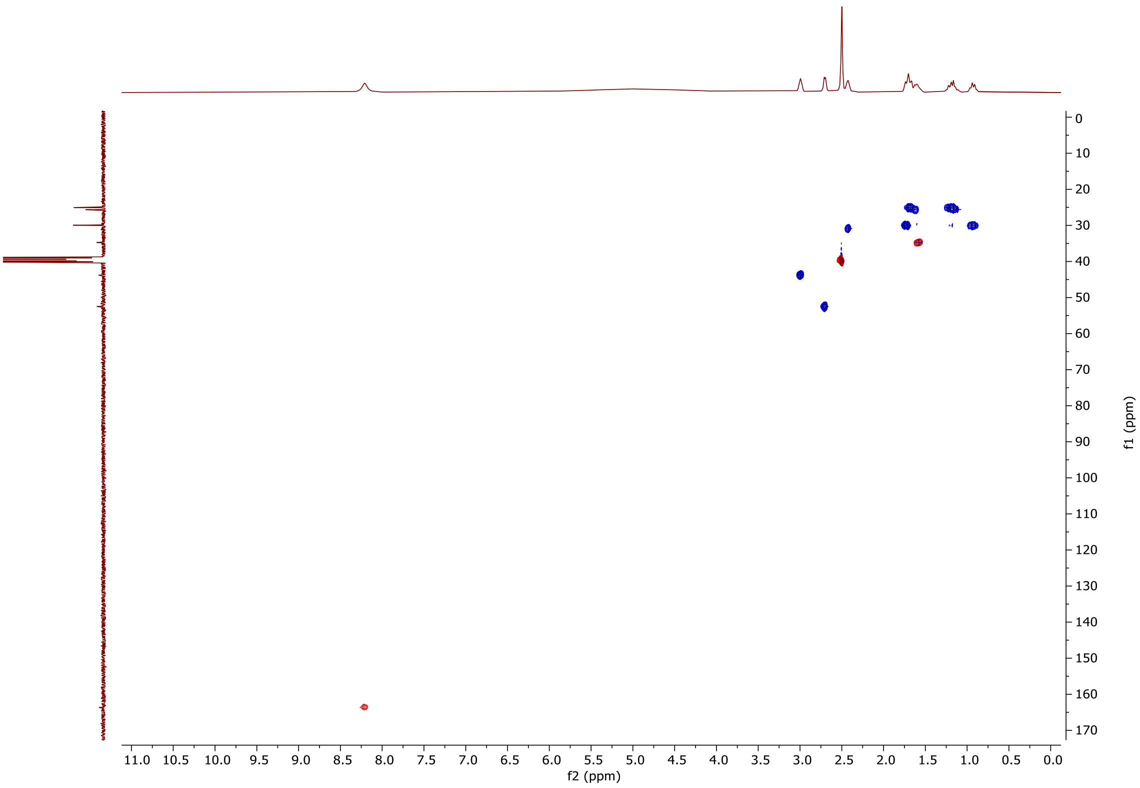

Figure S8. NMR spectra (DMSO) of **5e**: a) ^{1}H, b) ^{13}C, c) COSY, d) HMBC and e) HSQC.

a)

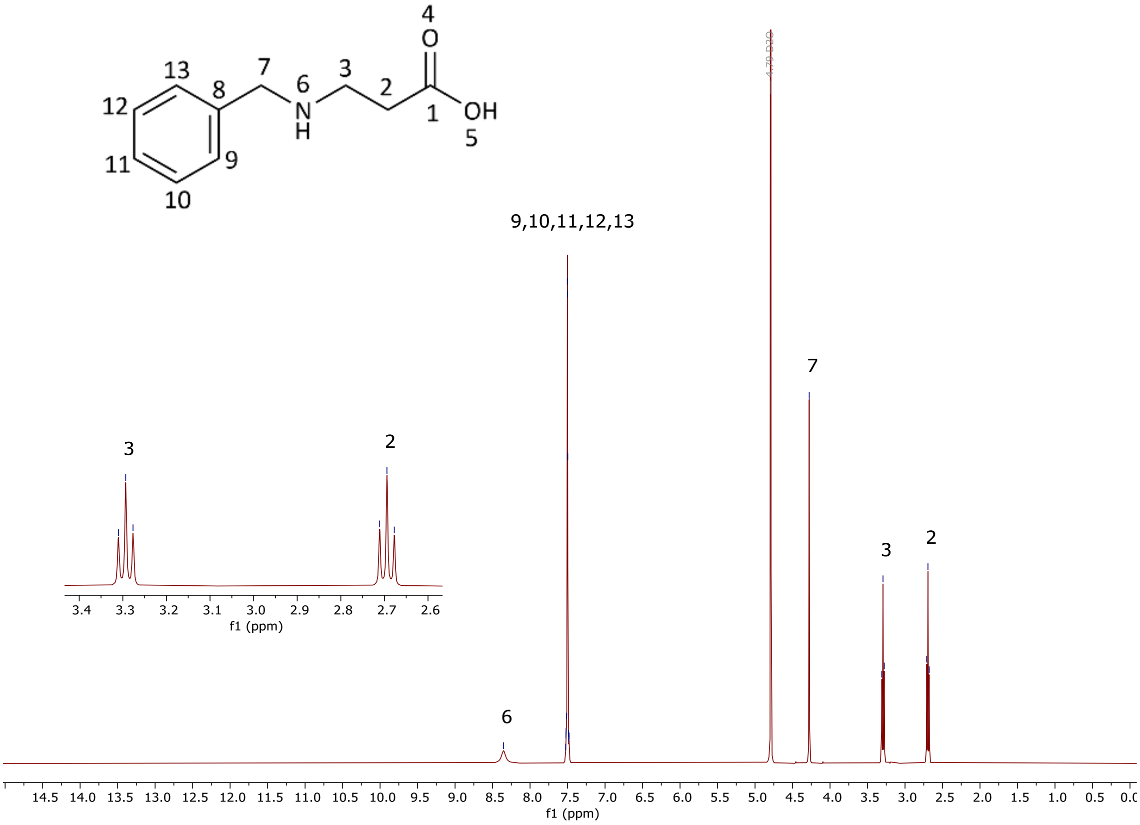

b)

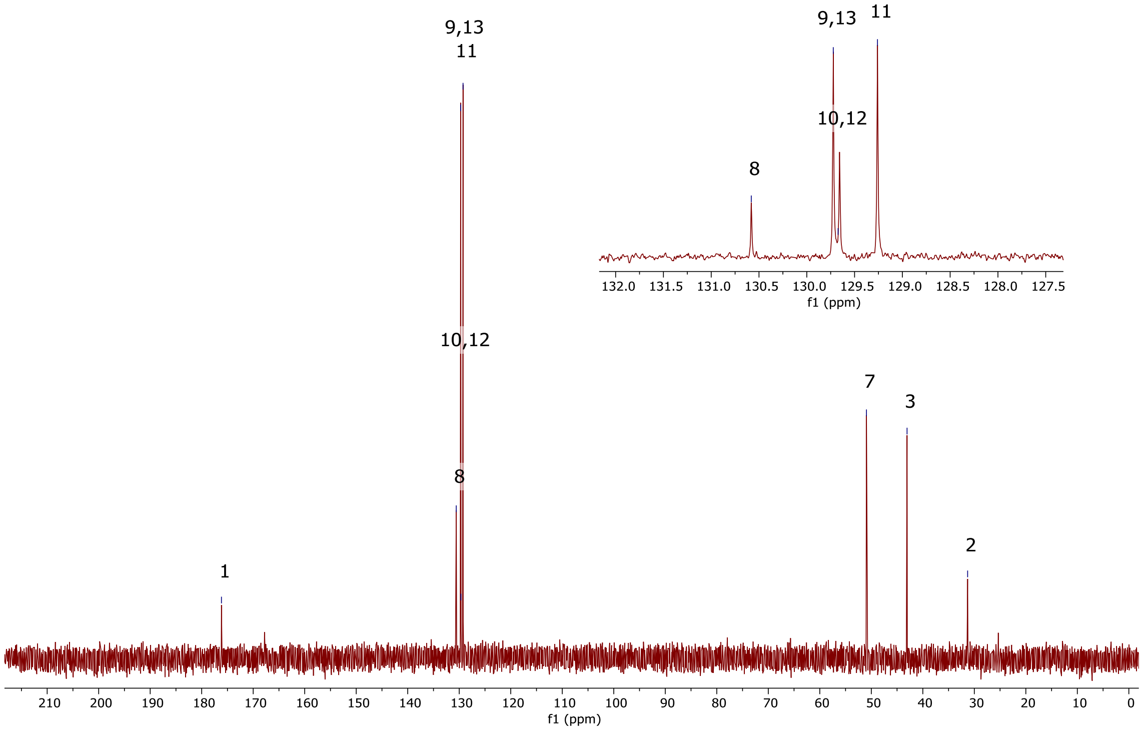
9,13
11
10,12
8
1
7
3
2
9,13
11
10,12
8
132.0 131.5 131.0 130.5 130.0 129.5 129.0 128.5 128.0 127.5
f1 (ppm)
210 200 190 180 170 160 150 140 130 120 110 100 90 80 70 60 50 40 30 20 10 0
f1 (ppm)

c)

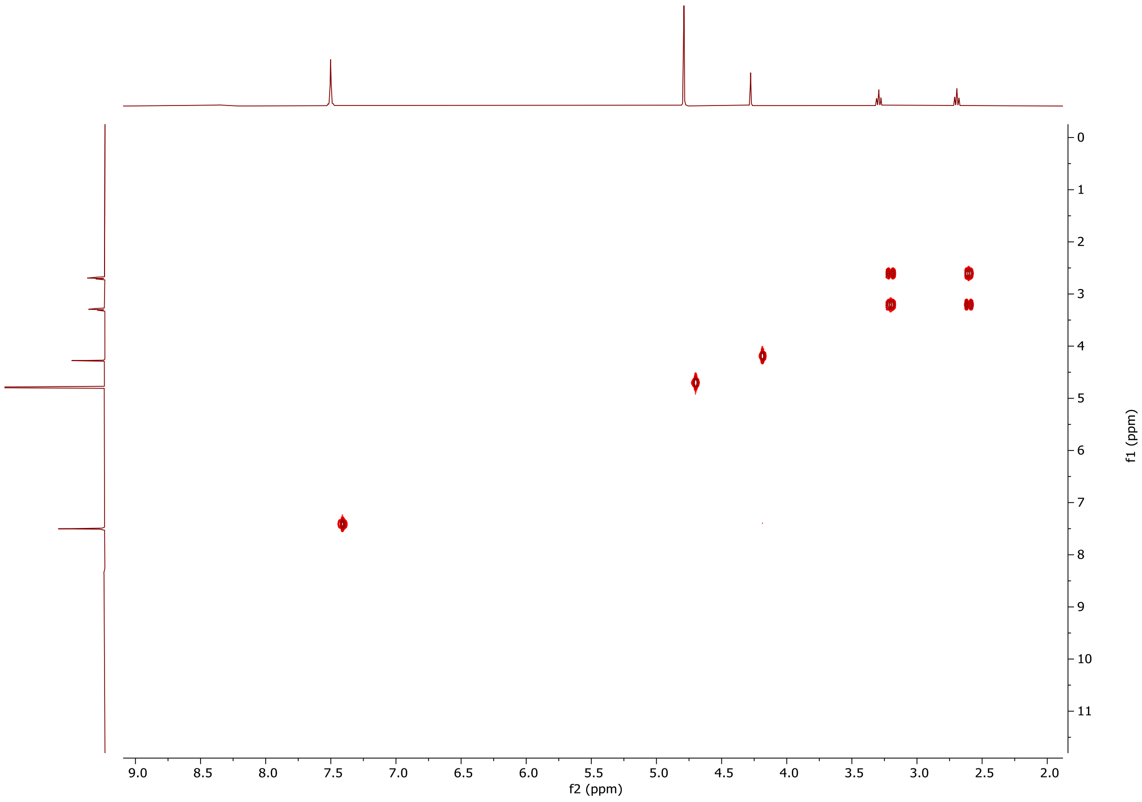
9.0 8.5 8.0 7.5 7.0 6.5 6.0 5.5 5.0 4.5 4.0 3.5 3.0 2.5 2.0
f2 (ppm)
0 1 2 3 4 5 6 7 8 9 10 11
f1 (ppm)

d)

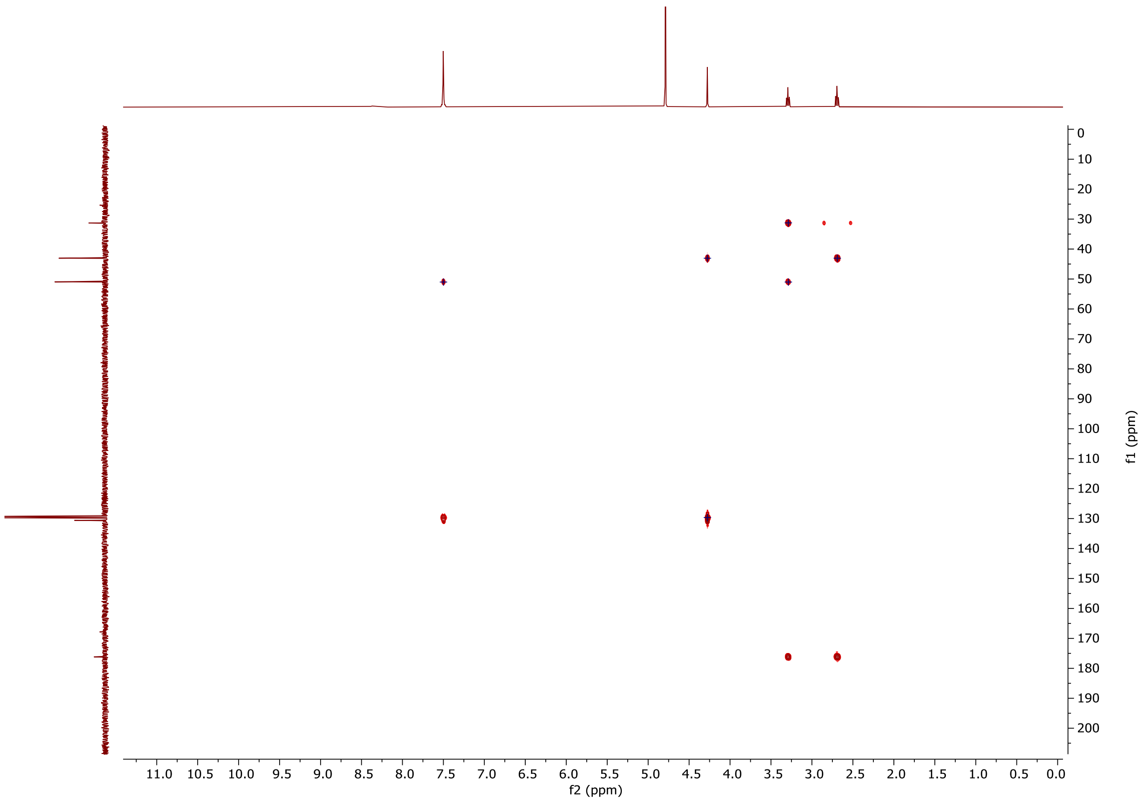

e)

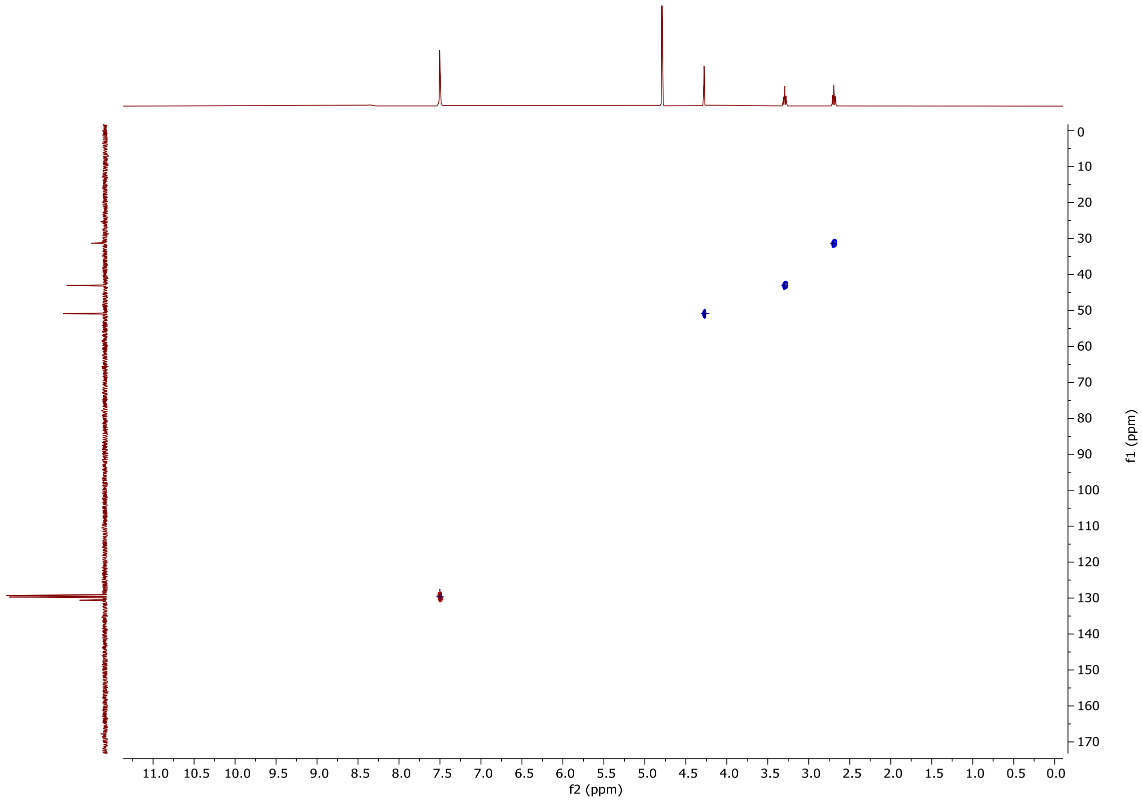

Figure S9. NMR spectra (D_2O) of **5g**: a) ^{1}H, b) ^{13}C, c) COSY, d) HMBC and e) HSQC.

a)

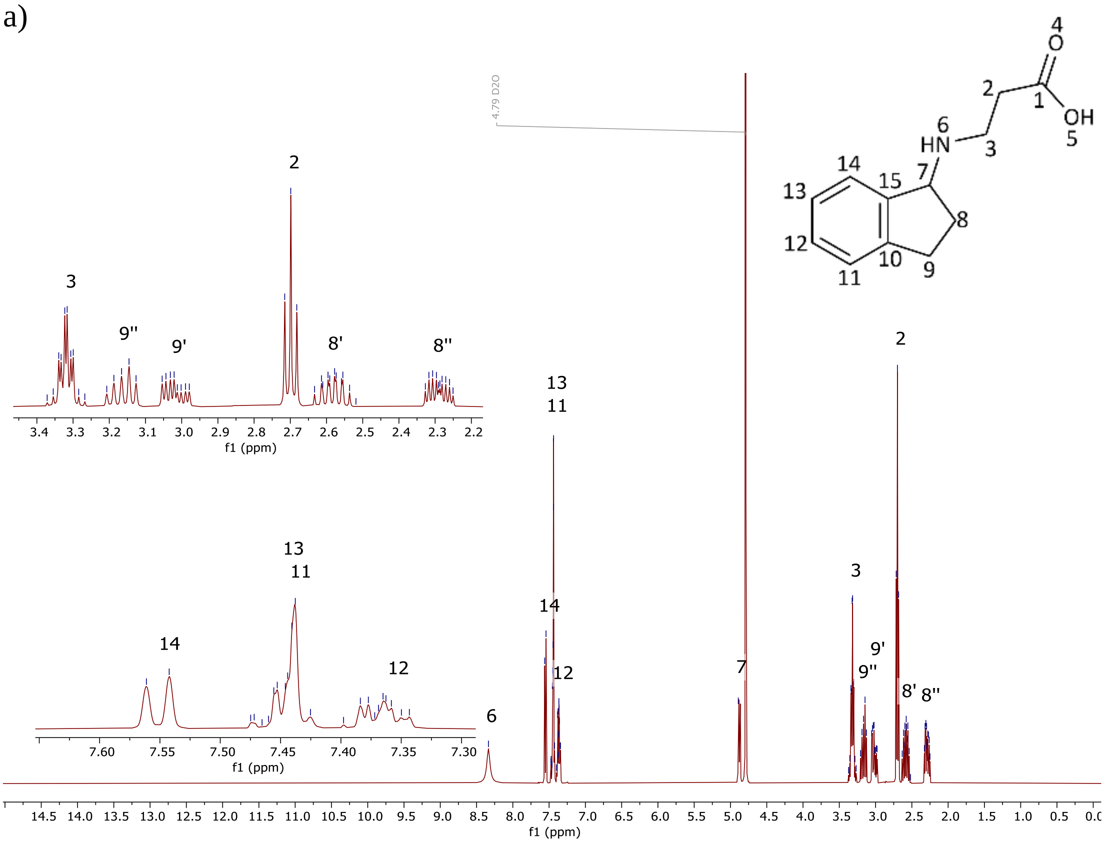
4.79 D2O
O
OH
HN
3
9''
9'
2
8'
8''
f1 (ppm)
13
11
14
12
6
7
f1 (ppm)

b)

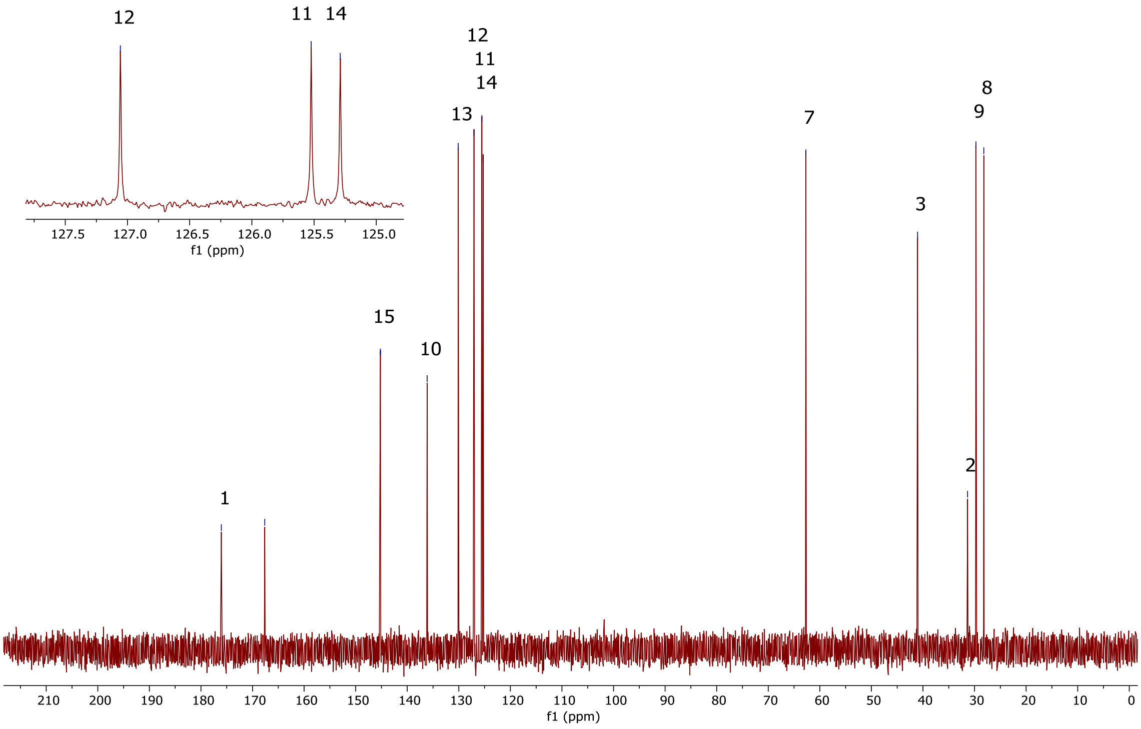
12
11 14
f1 (ppm)
13
15
10
1
7
3
2
8
9
f1 (ppm)

c)

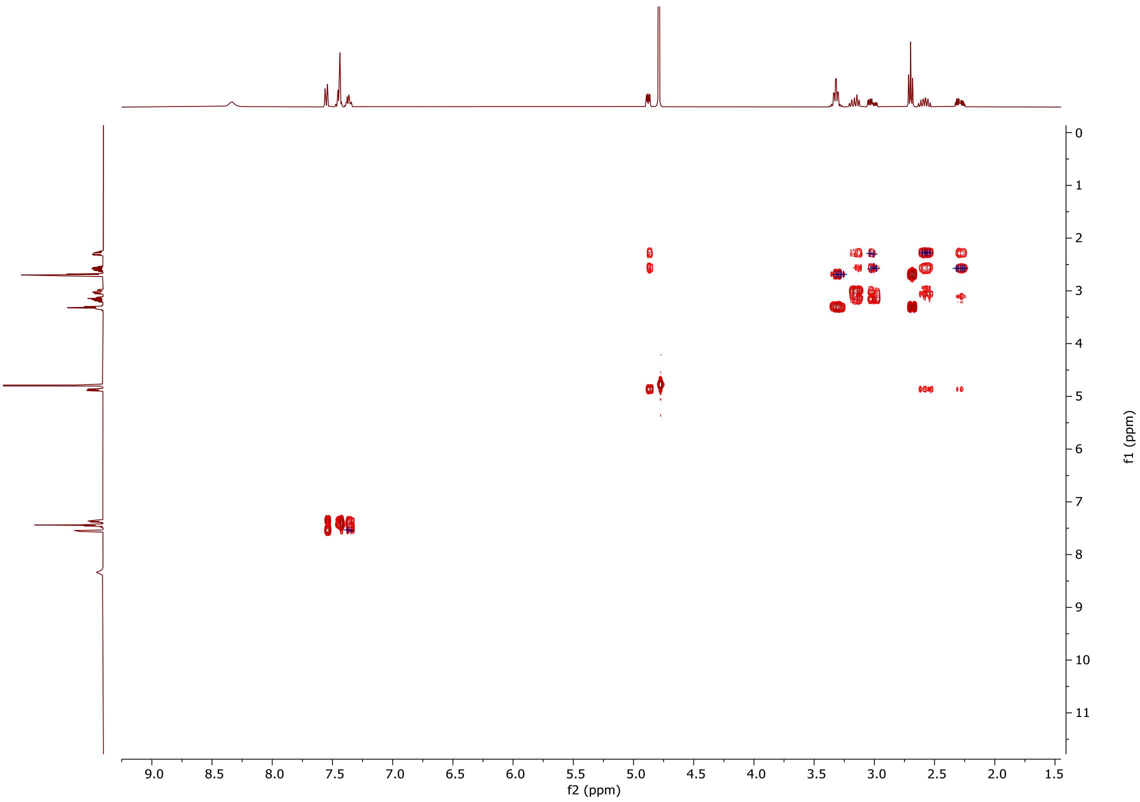

d)

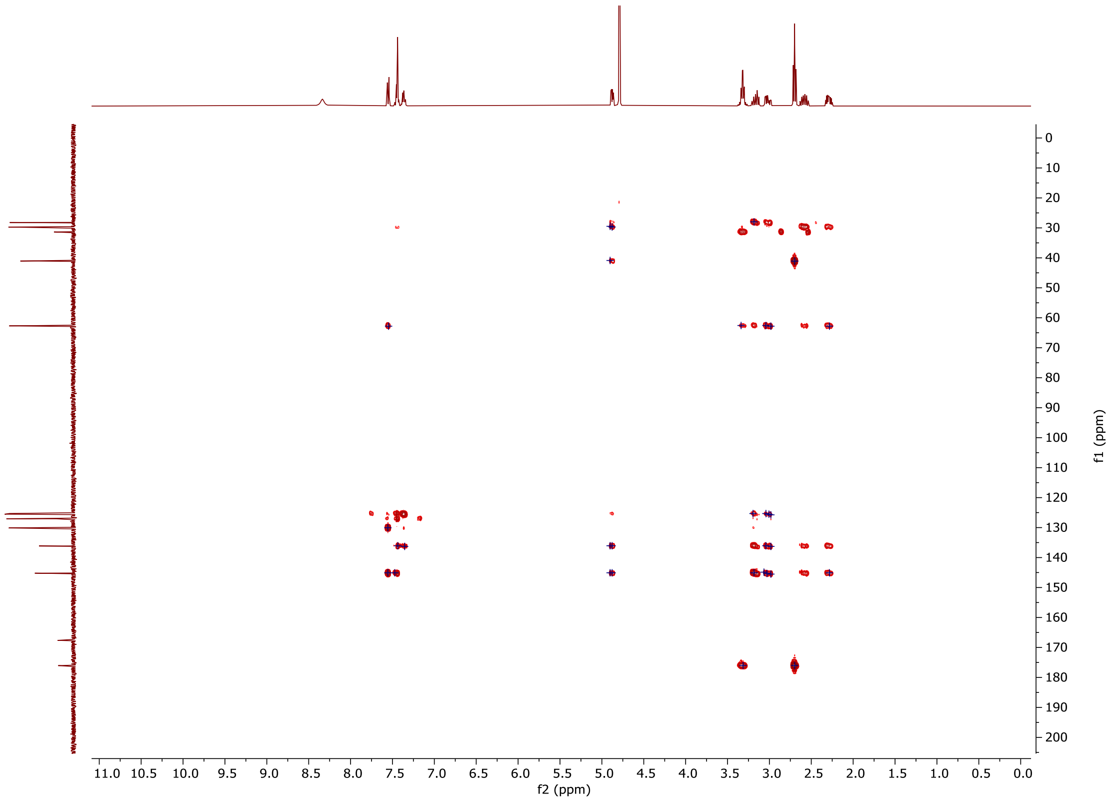

e)

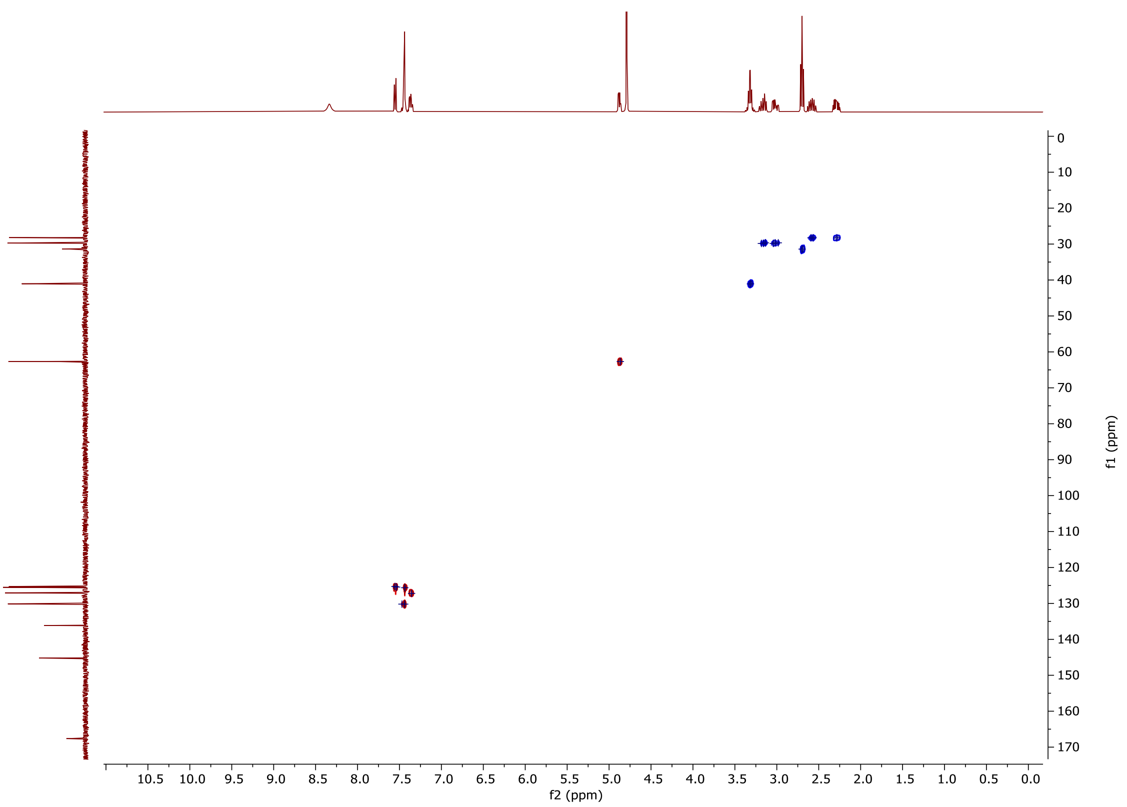

Figure S10. NMR spectra (D_2O) of *rac*-**5h**: a) ^{1}H, b) ^{13}C, c) COSY, d) HMBC and e) HSQC.

a)

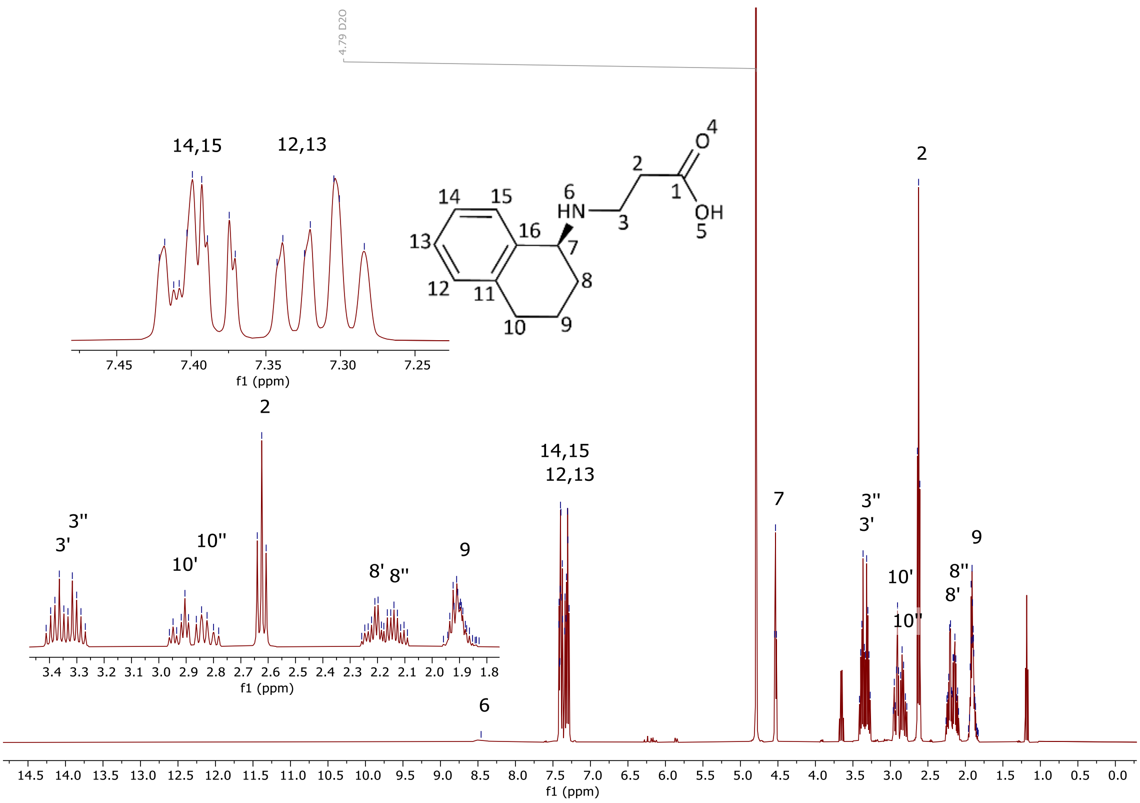

b)

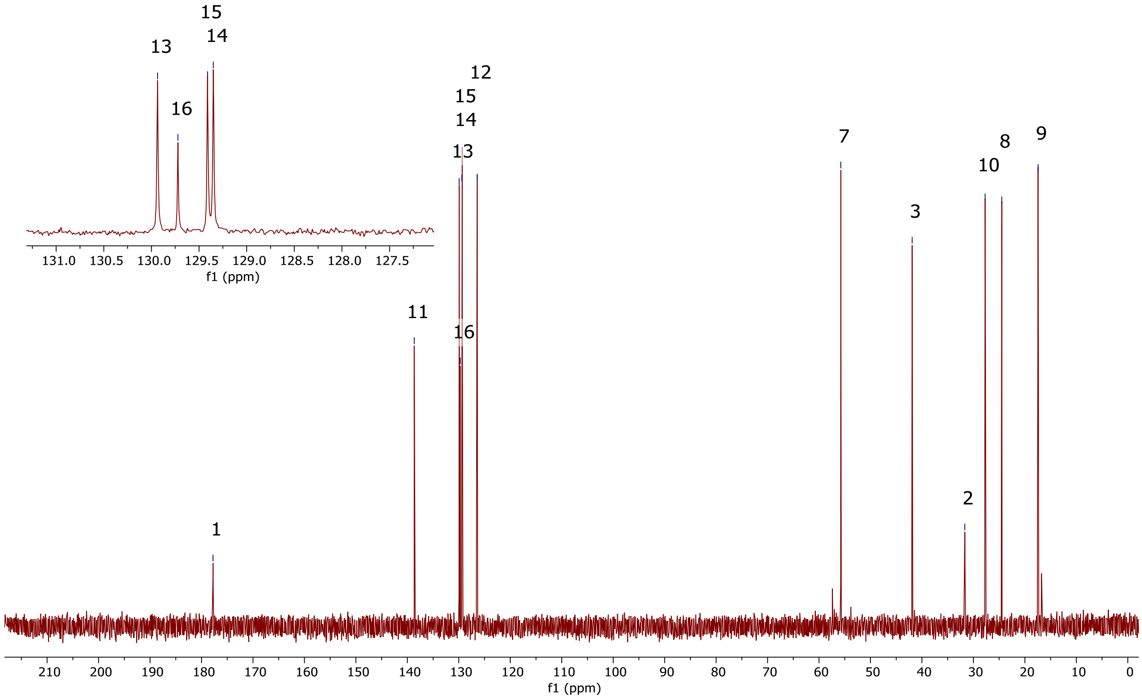
15
14
13
16
131.0 130.5 130.0 129.5 129.0 128.5 128.0 127.5
f1 (ppm)
12
15
14
13
11
16
1
7
3
2
10
8
9
210 200 190 180 170 160 150 140 130 120 110 100 90 80 70 60 50 40 30 20 10 0
f1 (ppm)

c)

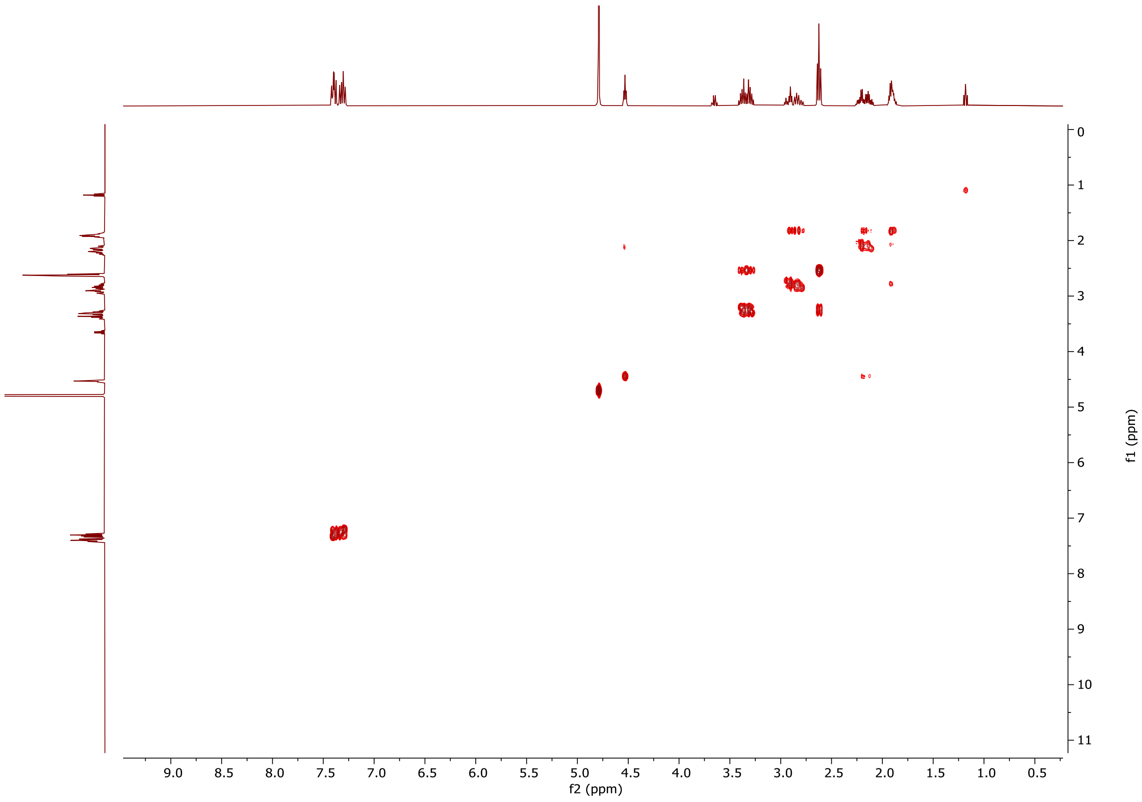
9.0 8.5 8.0 7.5 7.0 6.5 6.0 5.5 5.0 4.5 4.0 3.5 3.0 2.5 2.0 1.5 1.0 0.5
f2 (ppm)
0 1 2 3 4 5 6 7 8 9 10 11
f1 (ppm)

d)

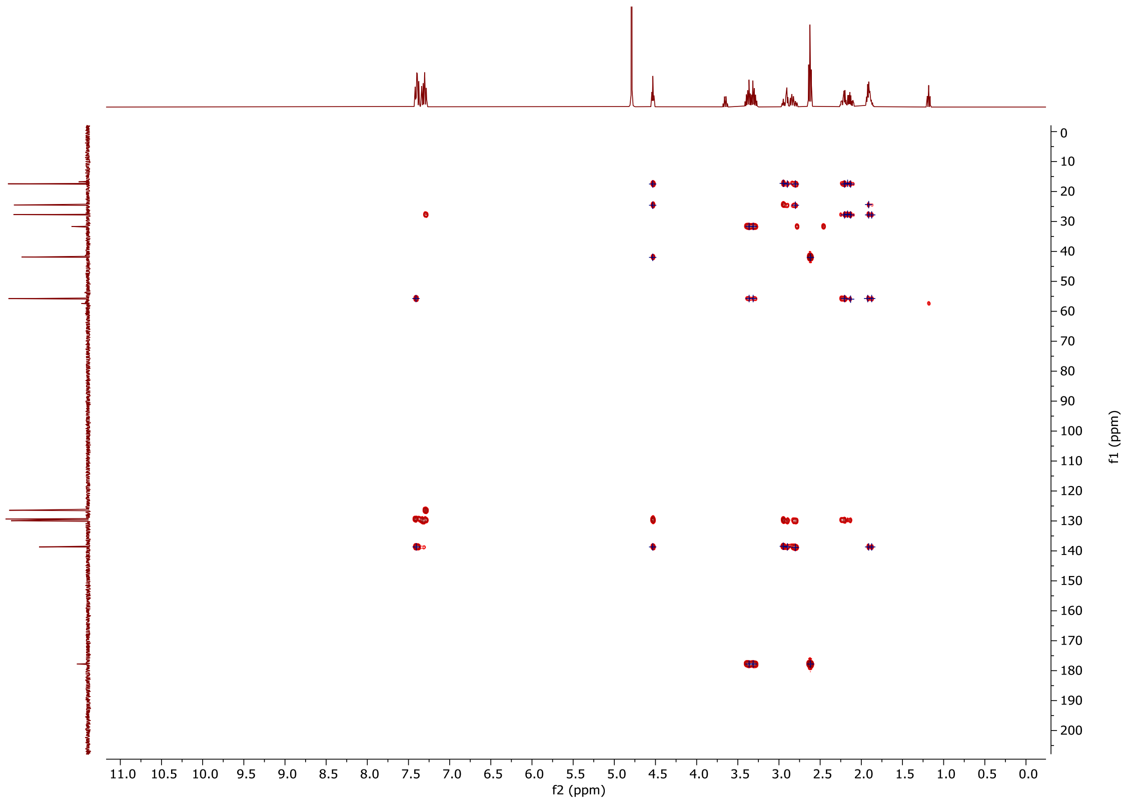

e)

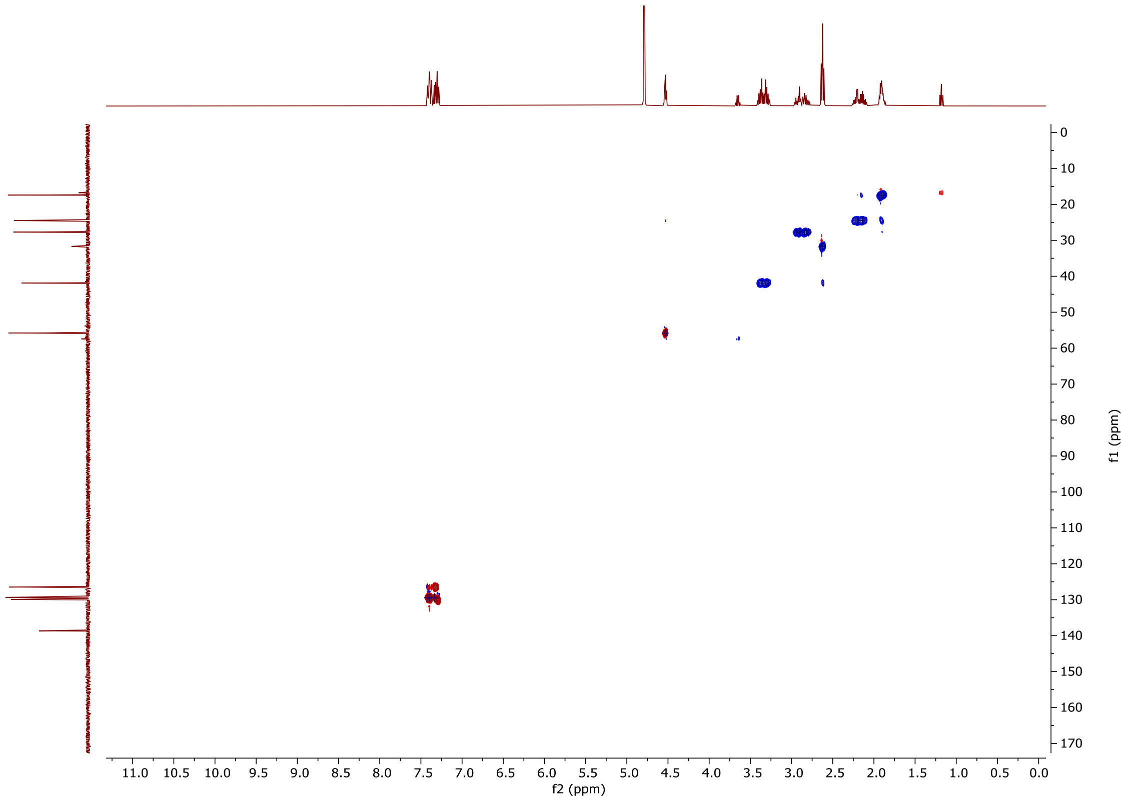

Figure S11. NMR spectra (D_2O) of (*S*)-**5i**: a) ^{1}H, b) ^{13}C, c) COSY, d) HMBC and e) HSQC.

a)

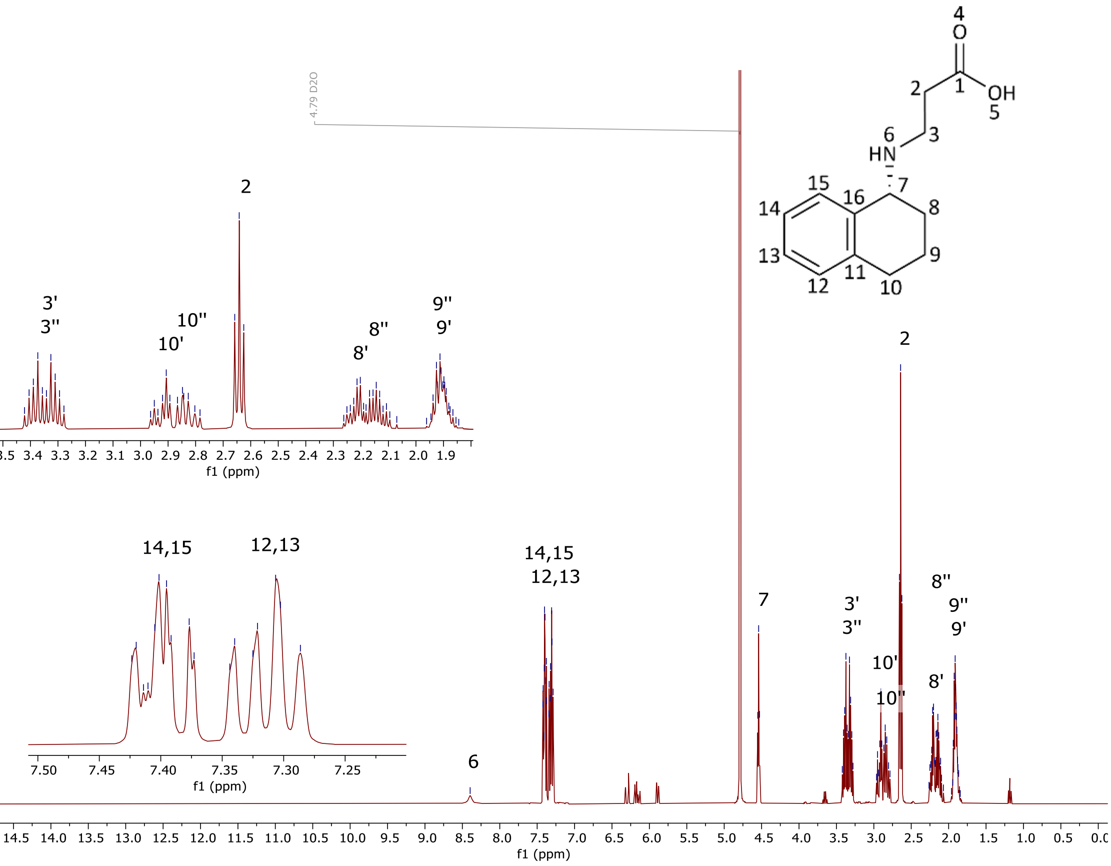

b)

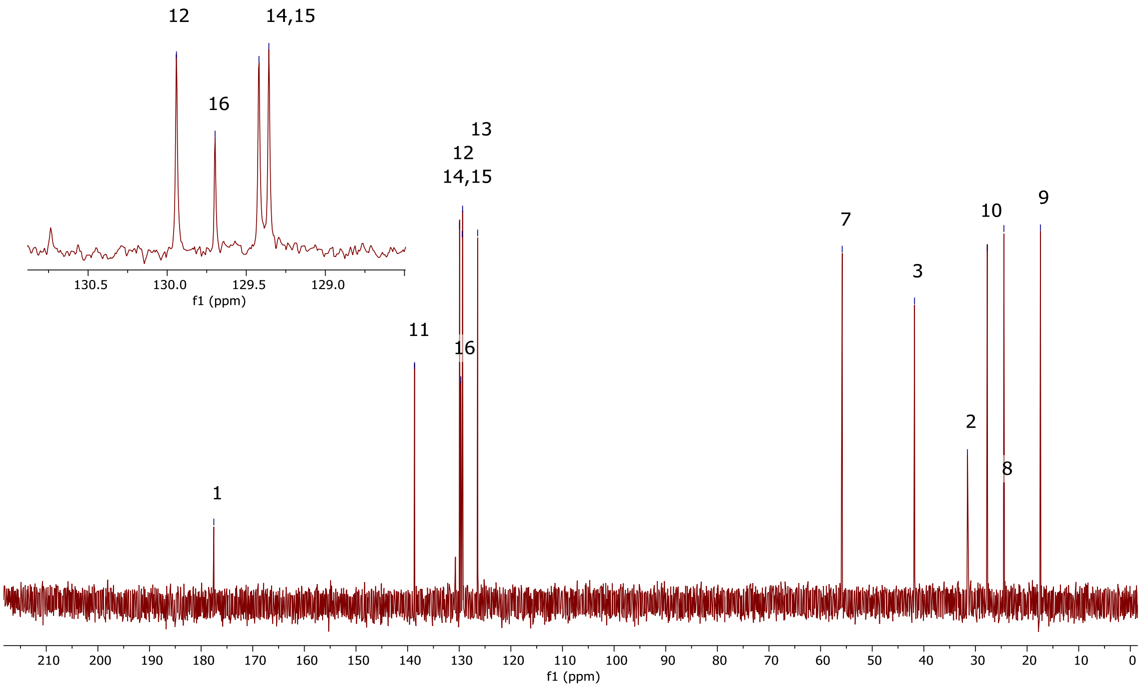

c)

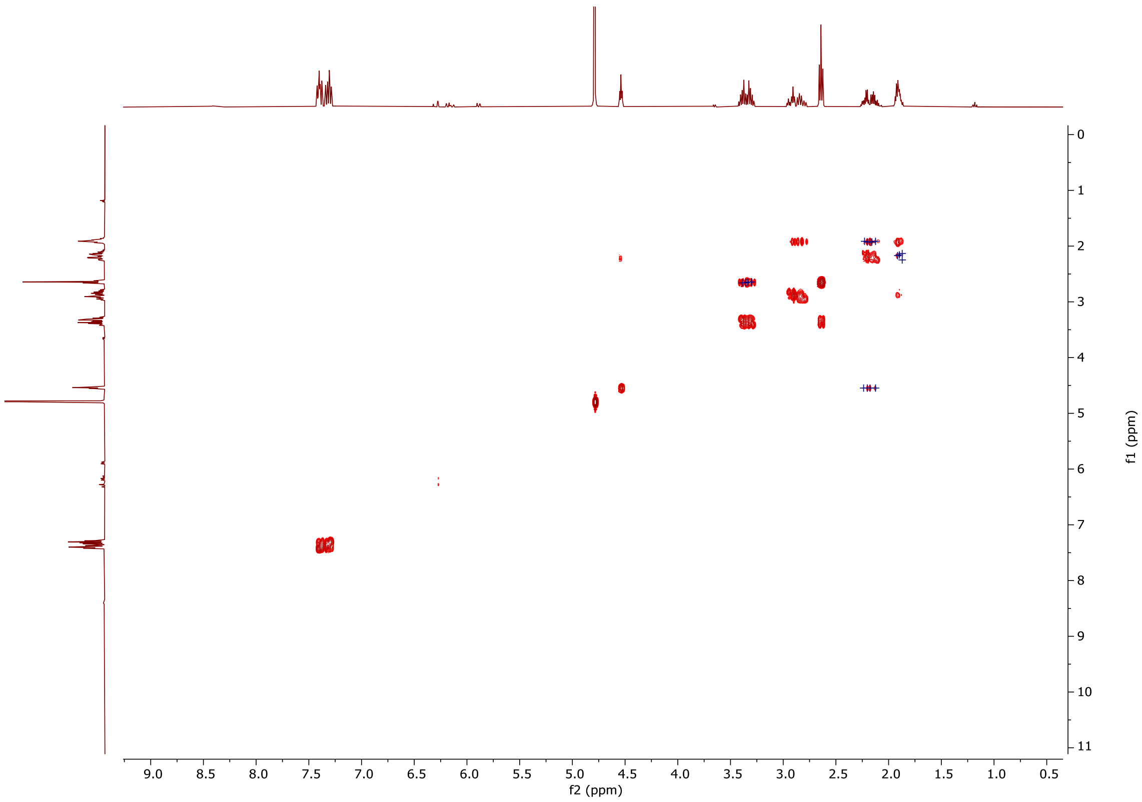

d)

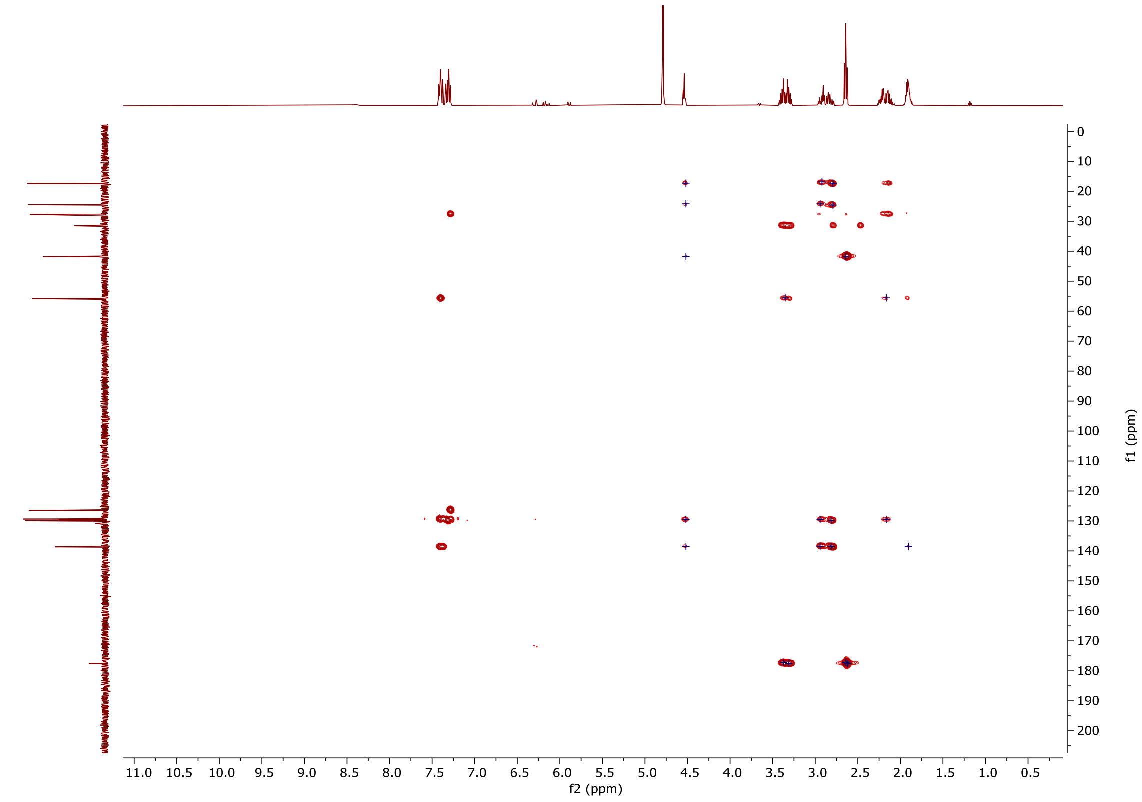

e)

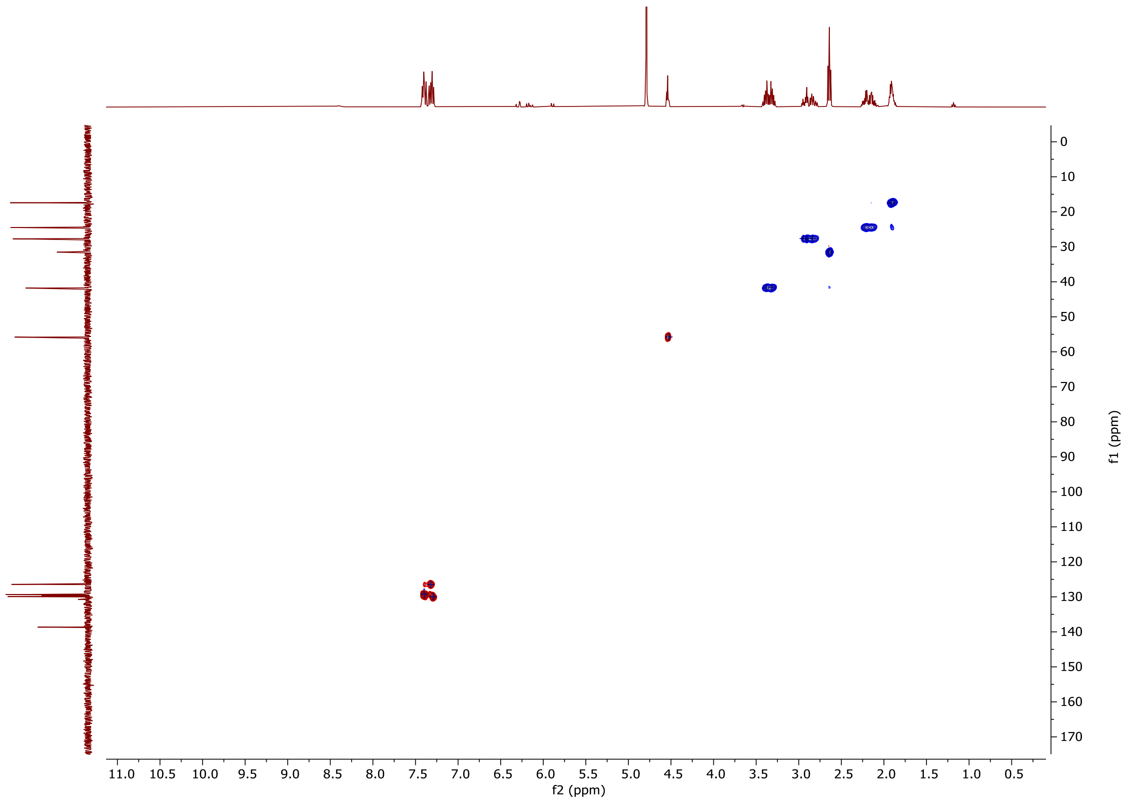

Figure S12. NMR spectra (D_2O) of (*R*)-**5i**: a) ^1H, b) ^{13}C, c) COSY, d) HMBC and e) HSQC.

a)

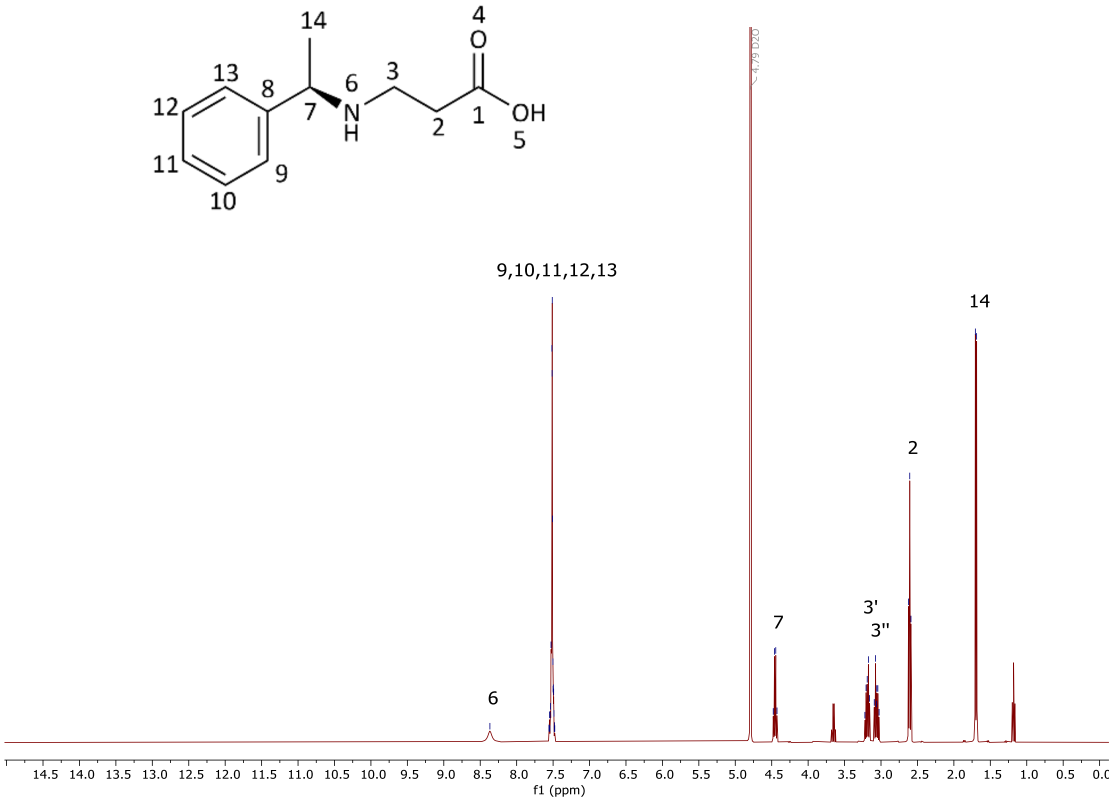

b)

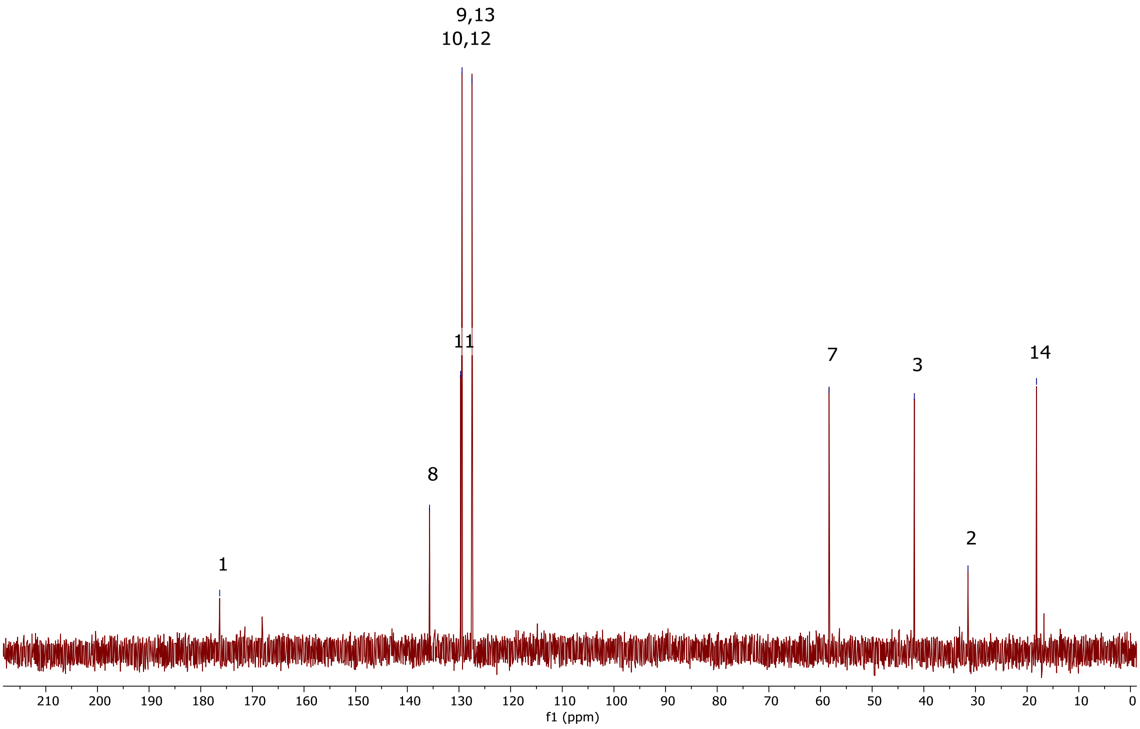
9,13
10,12
11
8
1
7
3
2
14
210 200 190 180 170 160 150 140 130 120 110 100 90 80 70 60 50 40 30 20 10 0
f1 (ppm)

c)

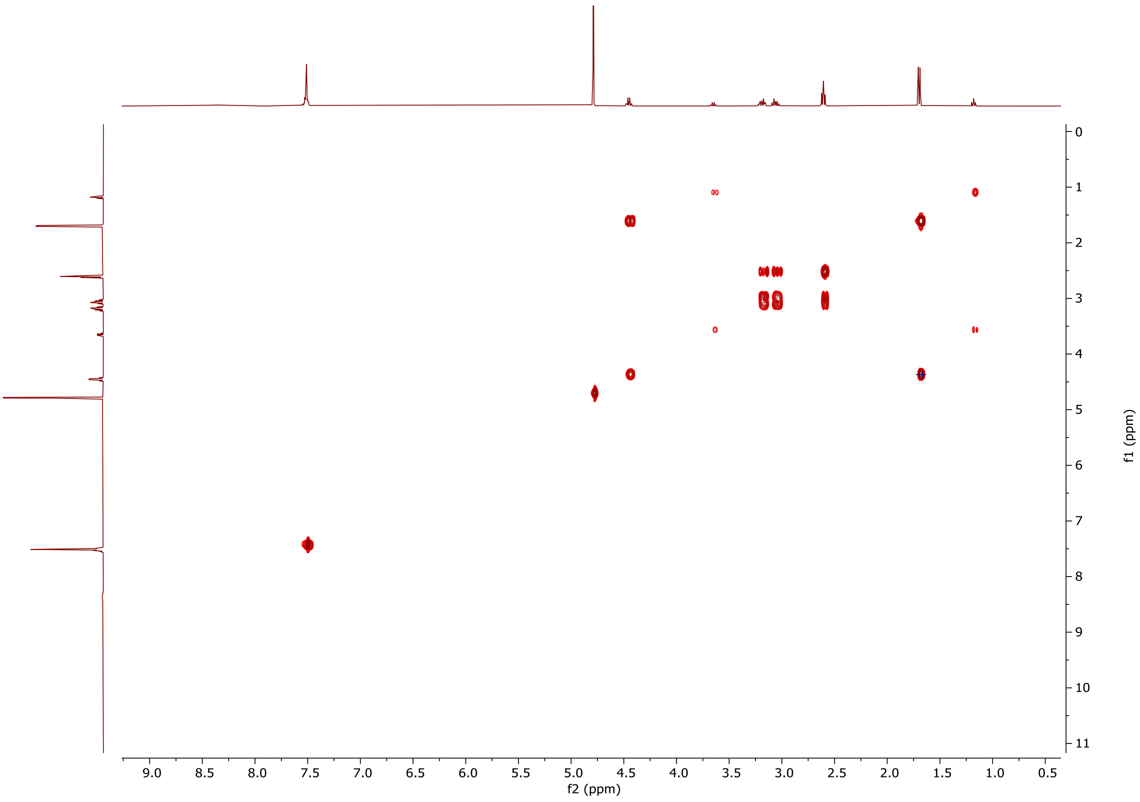
9.0 8.5 8.0 7.5 7.0 6.5 6.0 5.5 5.0 4.5 4.0 3.5 3.0 2.5 2.0 1.5 1.0 0.5
f2 (ppm)
0 1 2 3 4 5 6 7 8 9 10 11
f1 (ppm)

d)

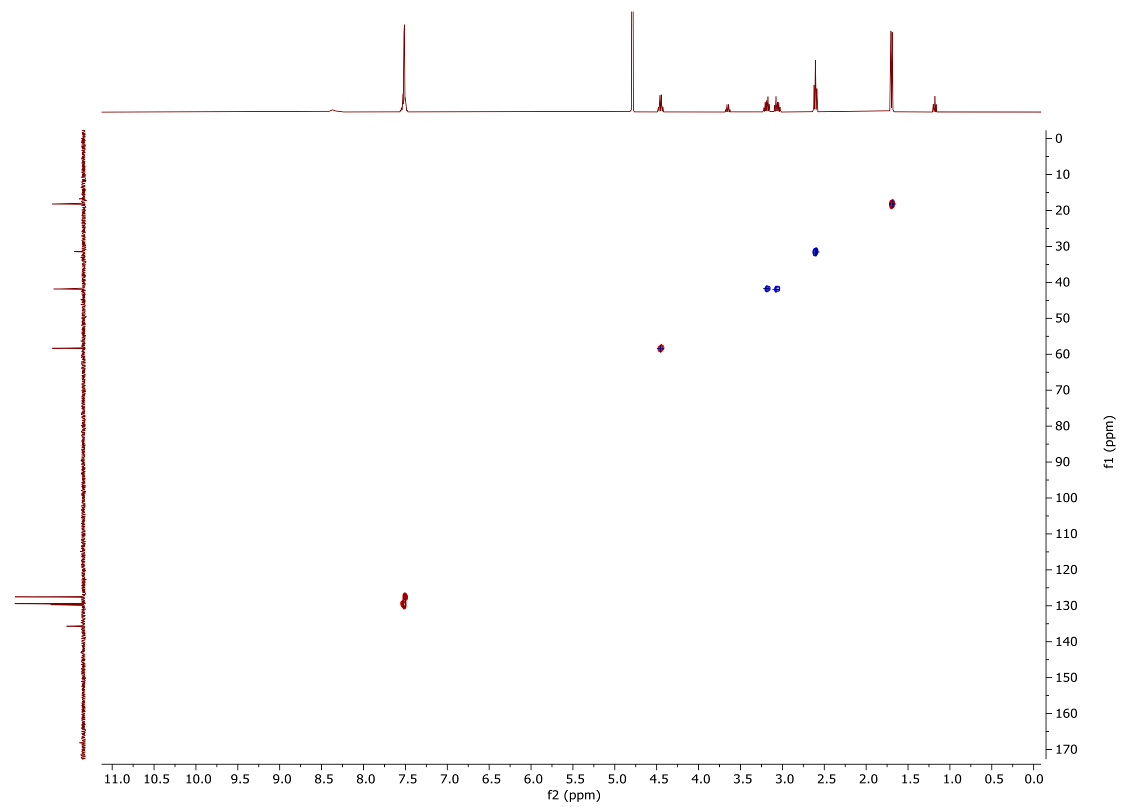

e)

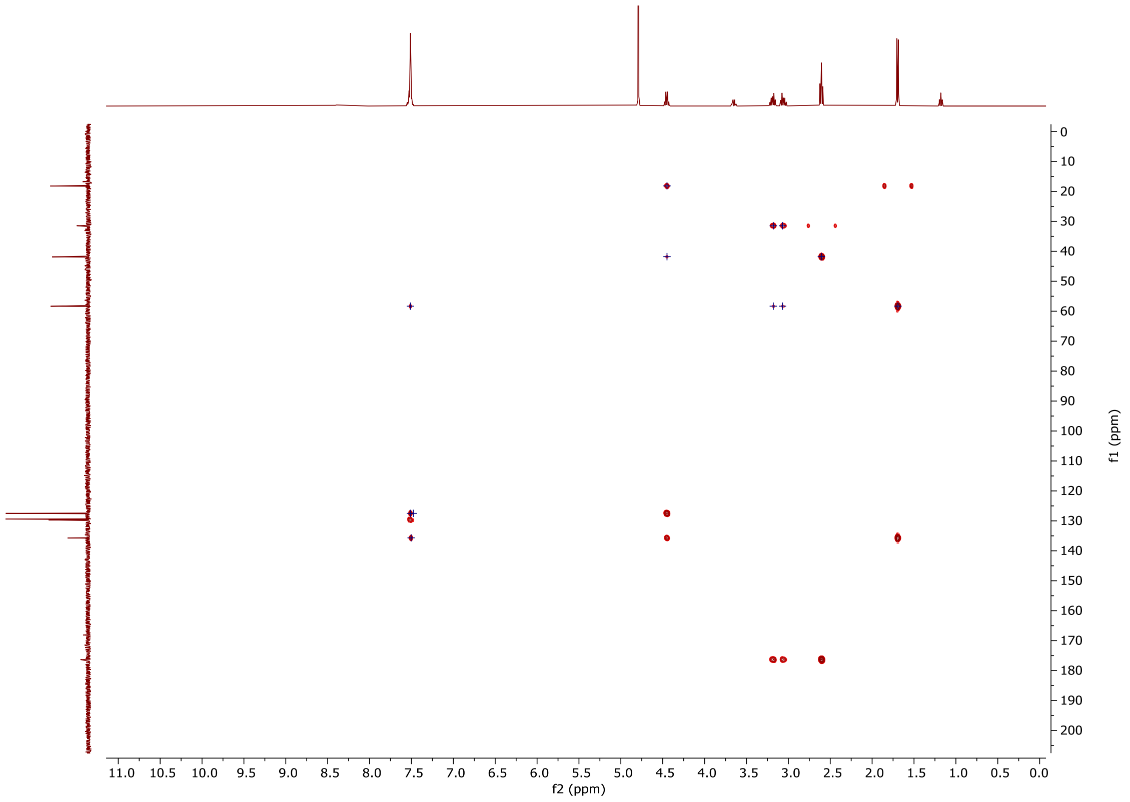

Figure S13. NMR spectra (D_2O) of (*S*)-**5j**: a) ^{1}H, b) ^{13}C, c) COSY, d) HMBC and e) HSQC.

a)

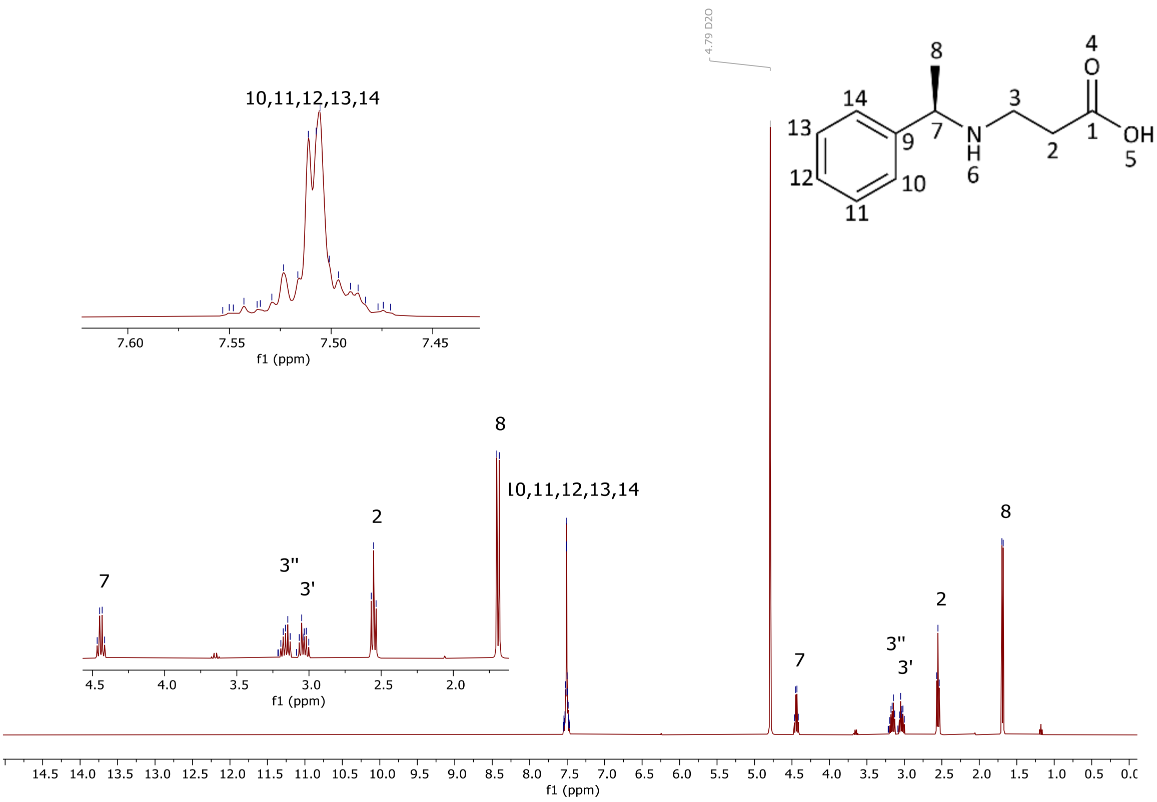
4.79 D2O
8
4
O
14
3
13
9
7
N
1
OH
H
12
2
5
10
6
11
10,11,12,13,14
7.60
7.55
7.50
7.45
f1 (ppm)
8
10,11,12,13,14
2
3''
7
3'
4.5
4.0
3.5
3.0
2.5
2.0
f1 (ppm)
7
3''
3'
2
8
14.5 14.0 13.5 13.0 12.5 12.0 11.5 11.0 10.5 10.0 9.5 9.0 8.5 8.0 7.5 7.0 6.5 6.0 5.5 5.0 4.5 4.0 3.5 3.0 2.5 2.0 1.5 1.0 0.5
f1 (ppm)

b)

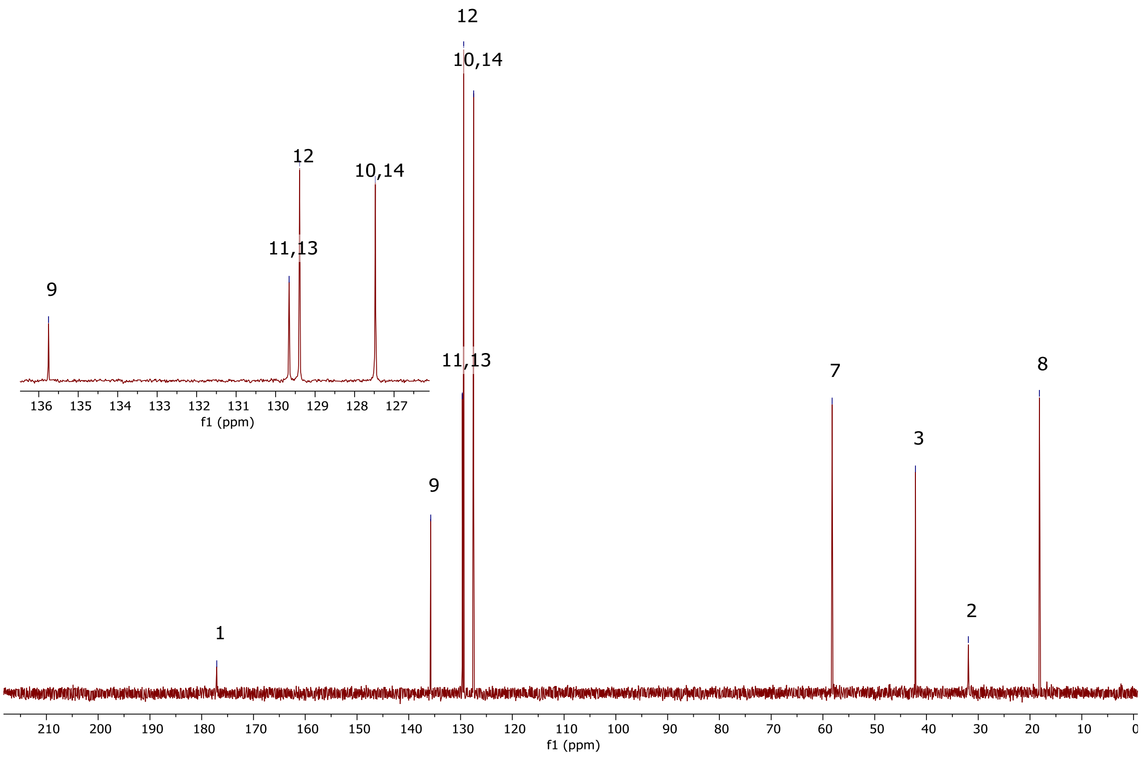
12
10,14
12
10,14
11,13
9
11,13
136 135 134 133 132 131 130 129 128 127
f1 (ppm)
7
8
3
9
2
1
210 200 190 180 170 160 150 140 130 120 110 100 90 80 70 60 50 40 30 20 10
f1 (ppm)

c)

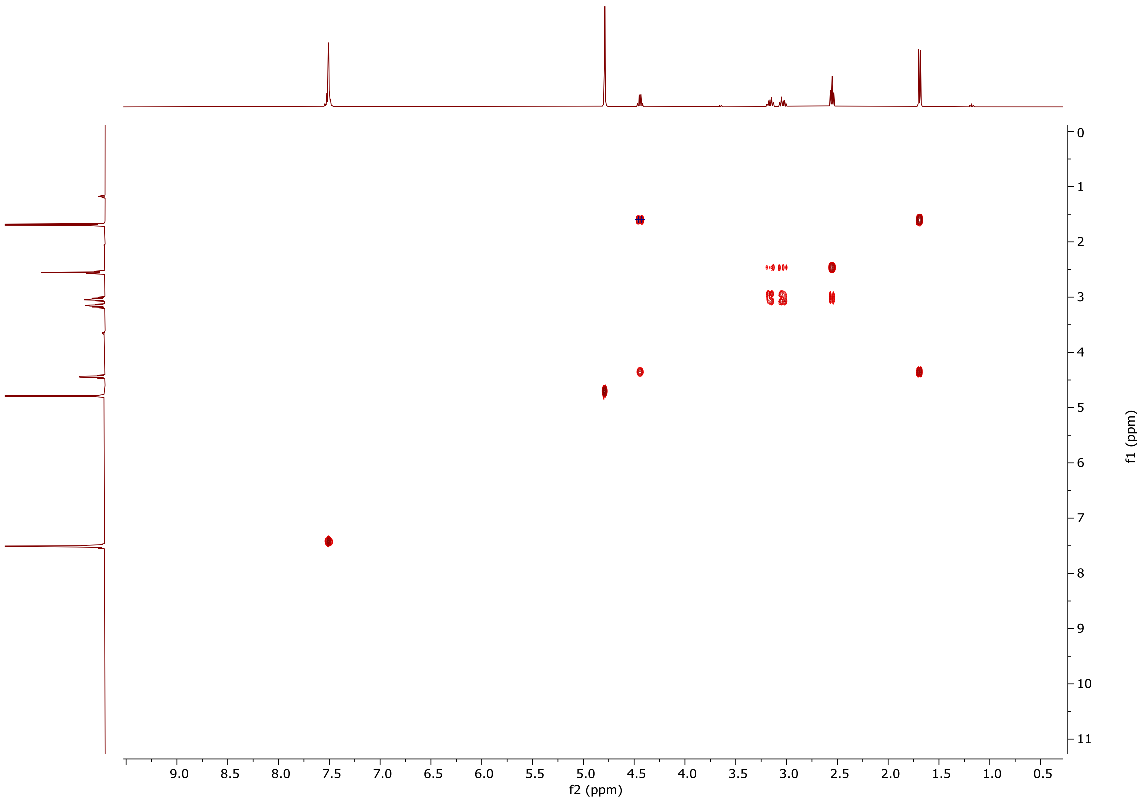

d)

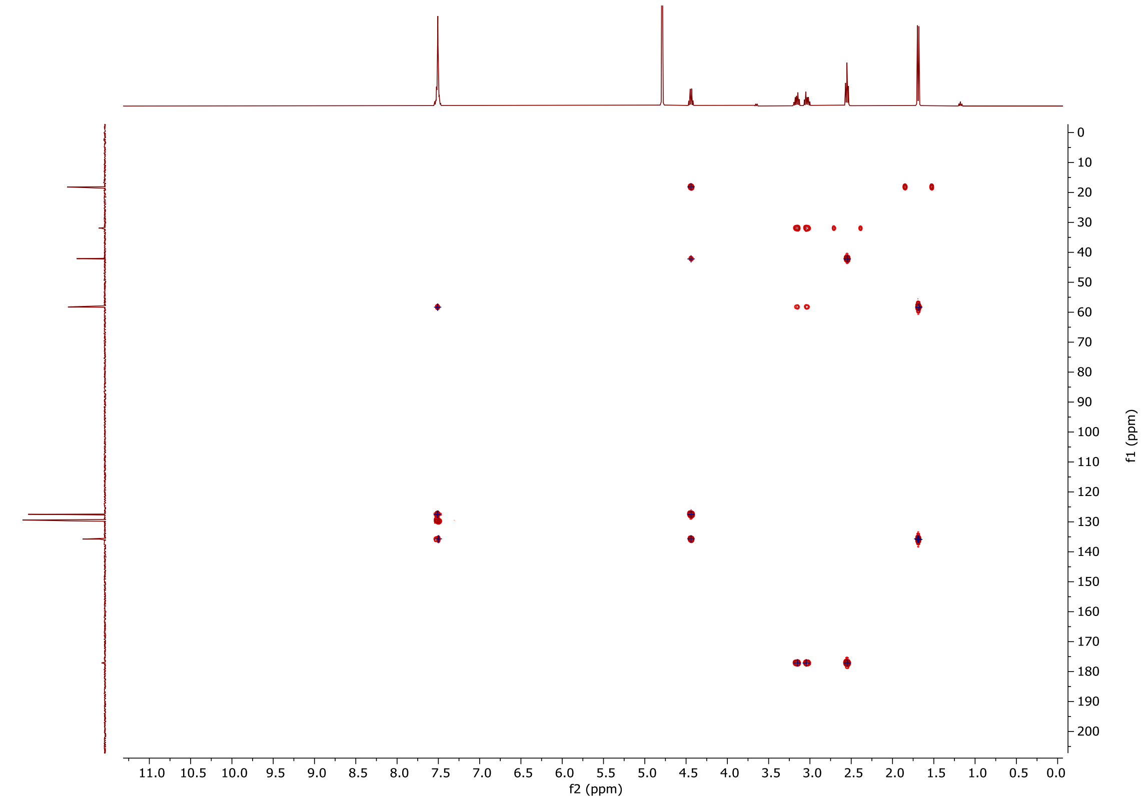

e)

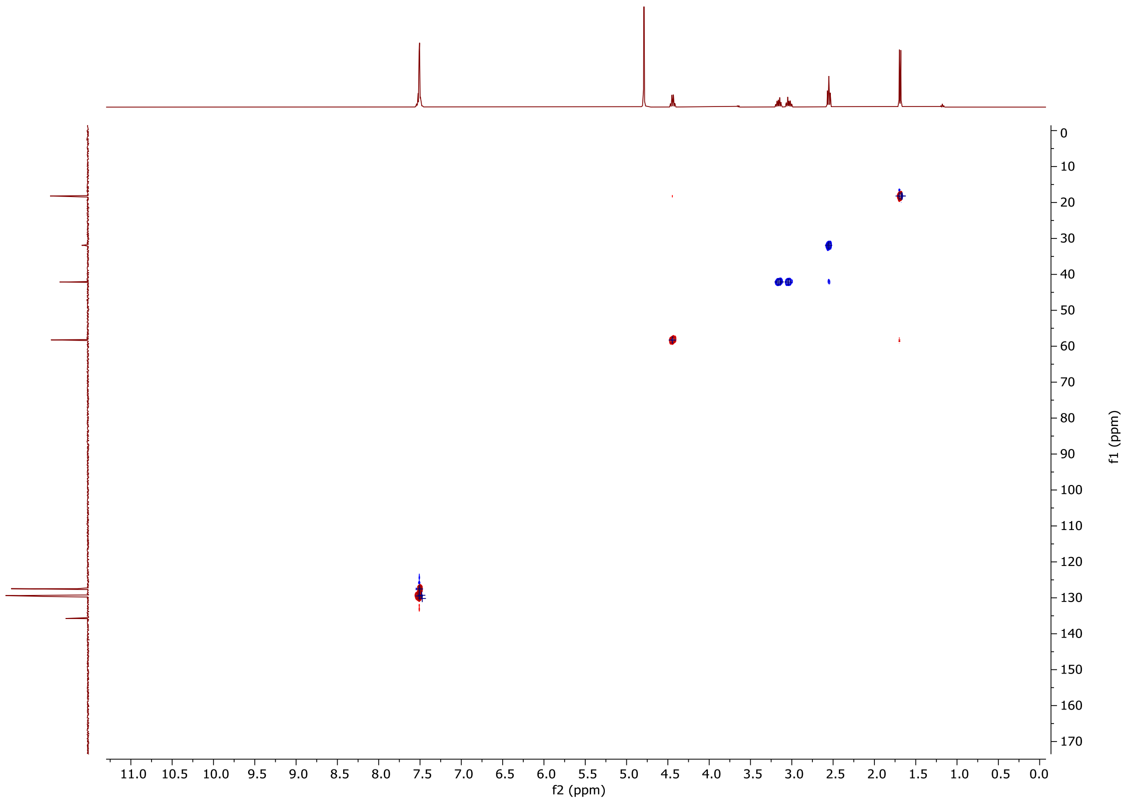

Figure S7. NMR spectra (D_2O) of (*R*)-**5j**: a) ^{1}H, b) ^{13}C, c) COSY, d) HMBC and e) HSQC.

a)

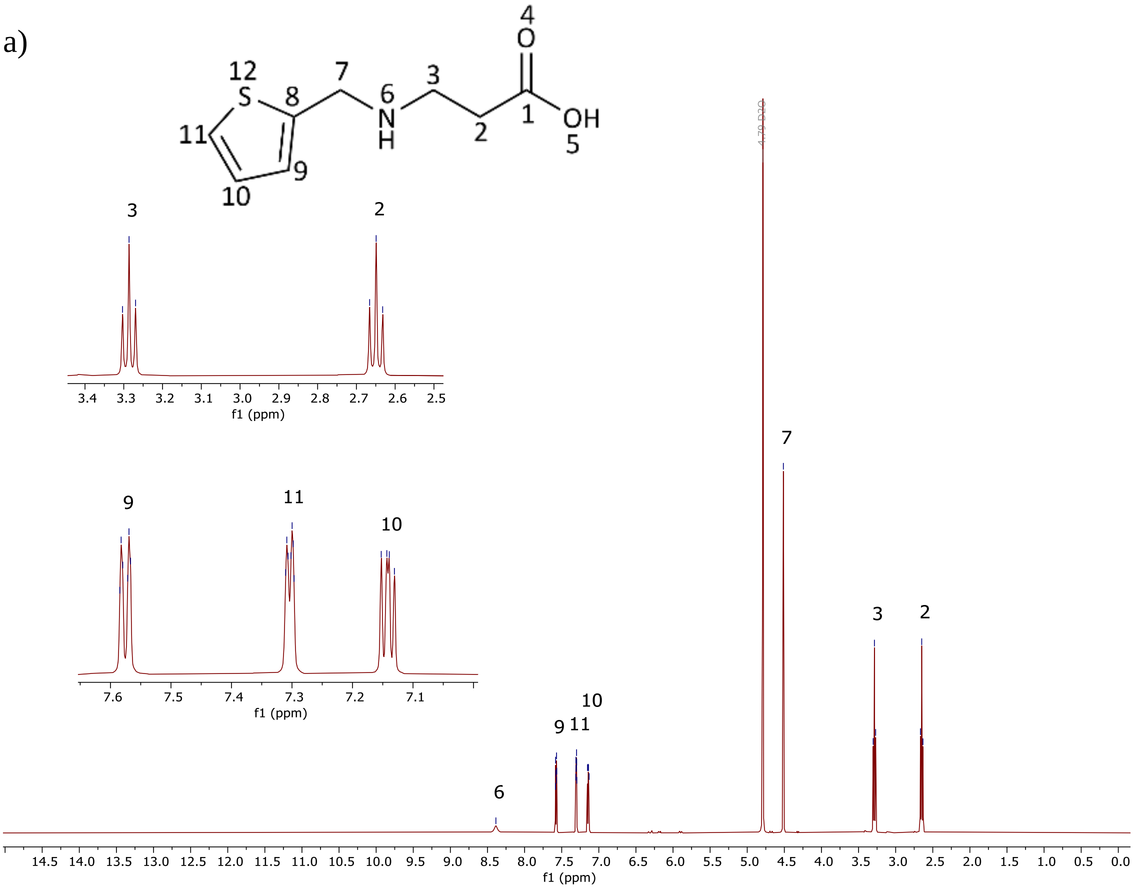

b)

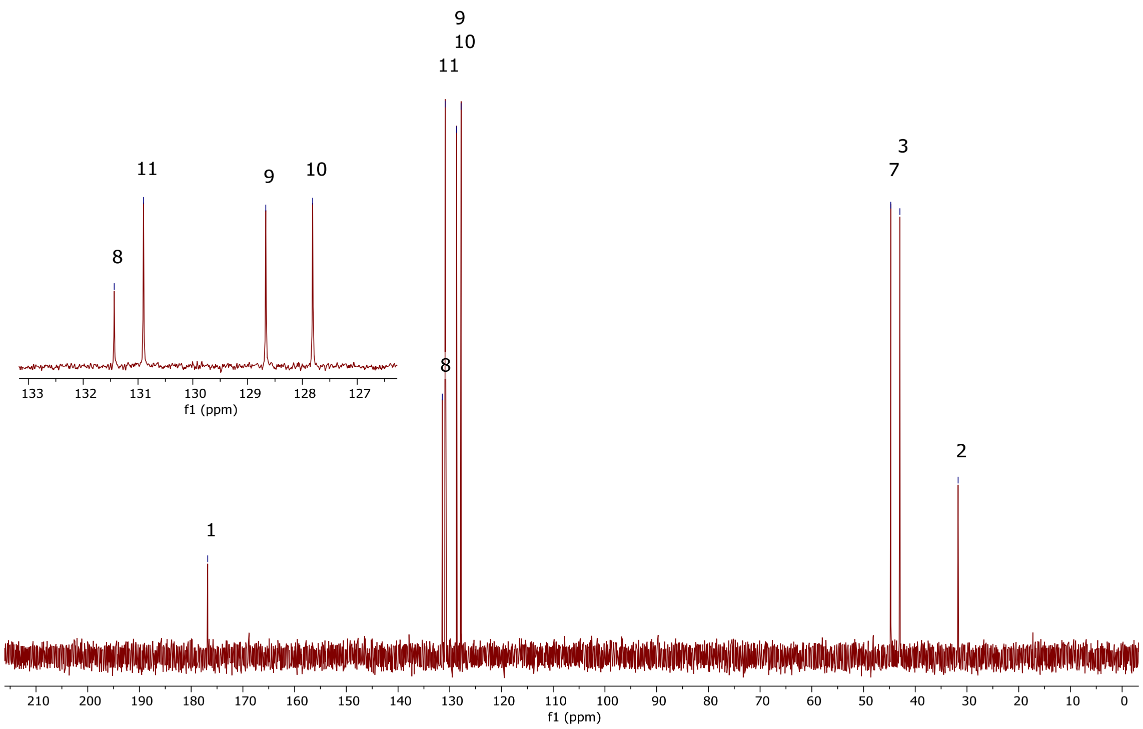
9
10
11
3
7
8
2
1
11
9
10
8
133 132 131 130 129 128 127
f1 (ppm)
210 200 190 180 170 160 150 140 130 120 110 100 90 80 70 60 50 40 30 20 10 0
f1 (ppm)

c)

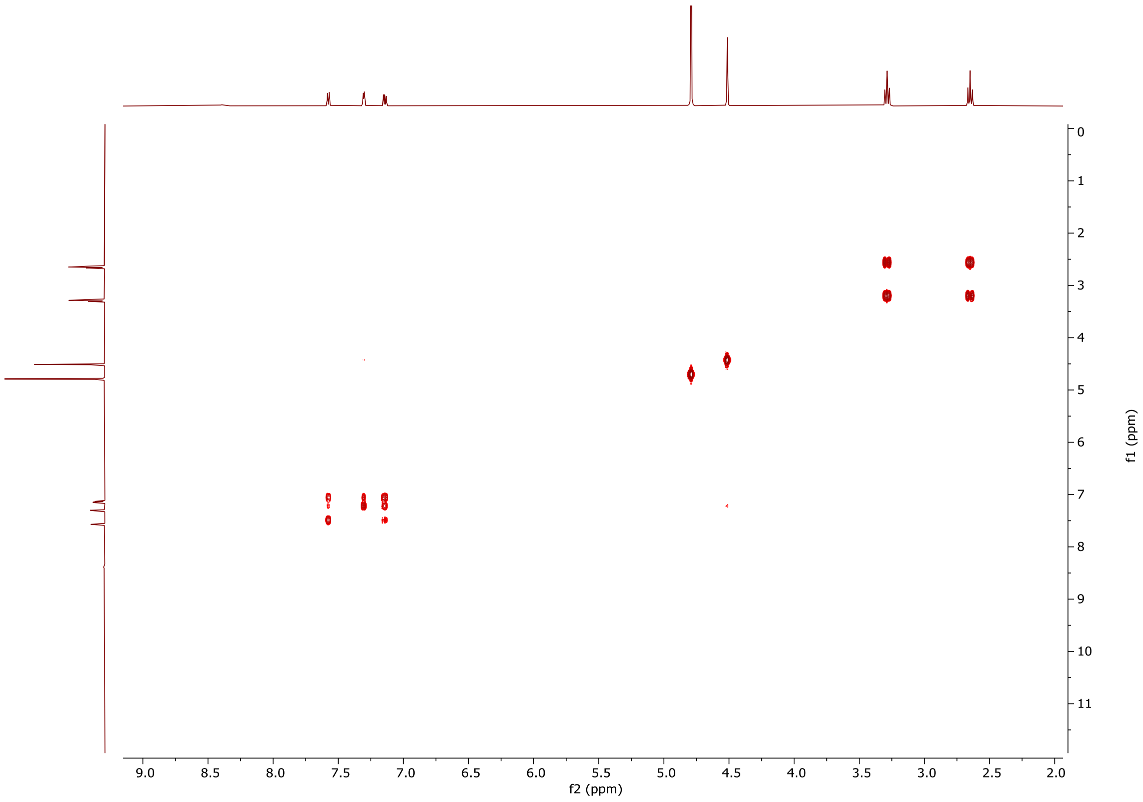
9.0 8.5 8.0 7.5 7.0 6.5 6.0 5.5 5.0 4.5 4.0 3.5 3.0 2.5 2.0
f2 (ppm)
0 1 2 3 4 5 6 7 8 9 10 11
f1 (ppm)

d)

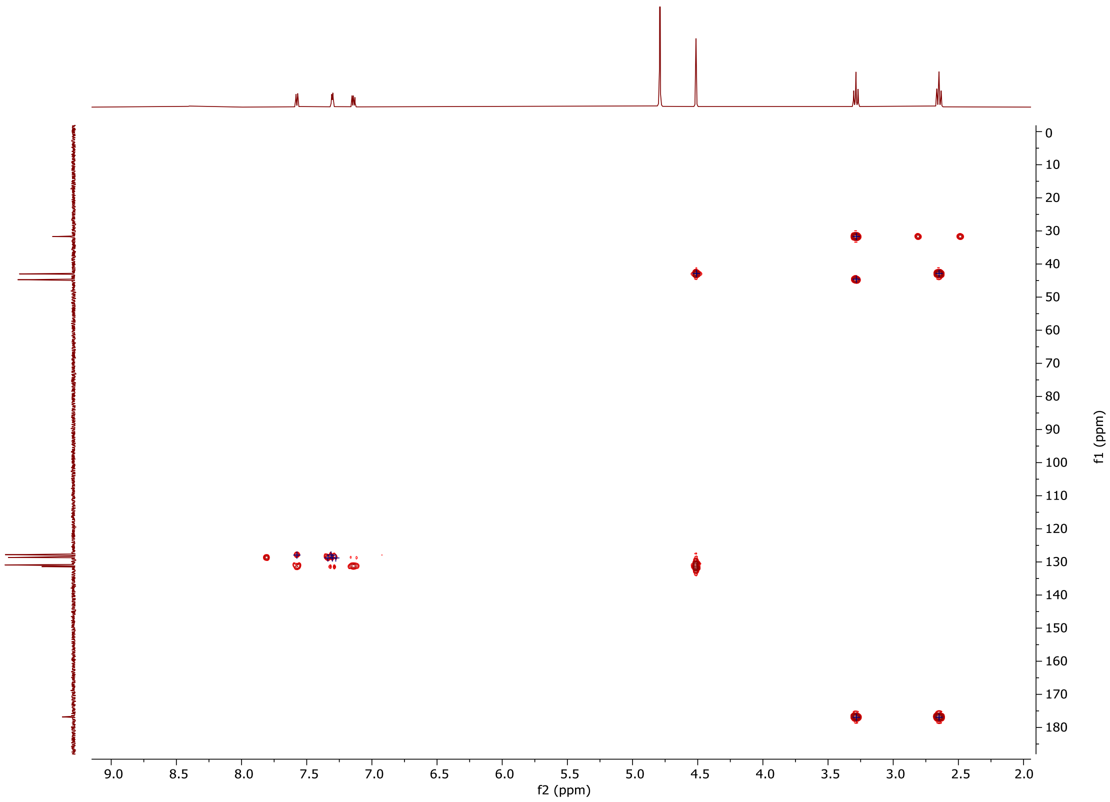

e)

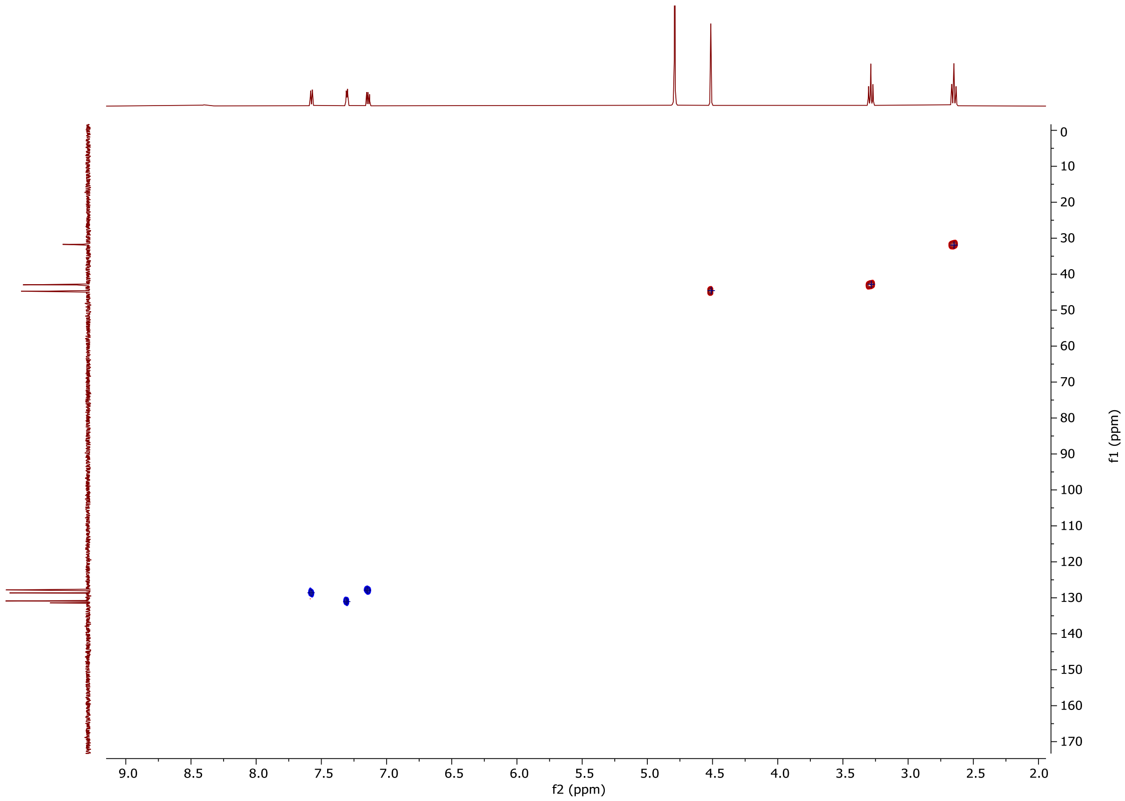

Figure S8. NMR spectra (D_2O) of **5o**: a) ^{1}H, b) ^{13}C, c) COSY, d) HMBC and e) HSQC.

a)

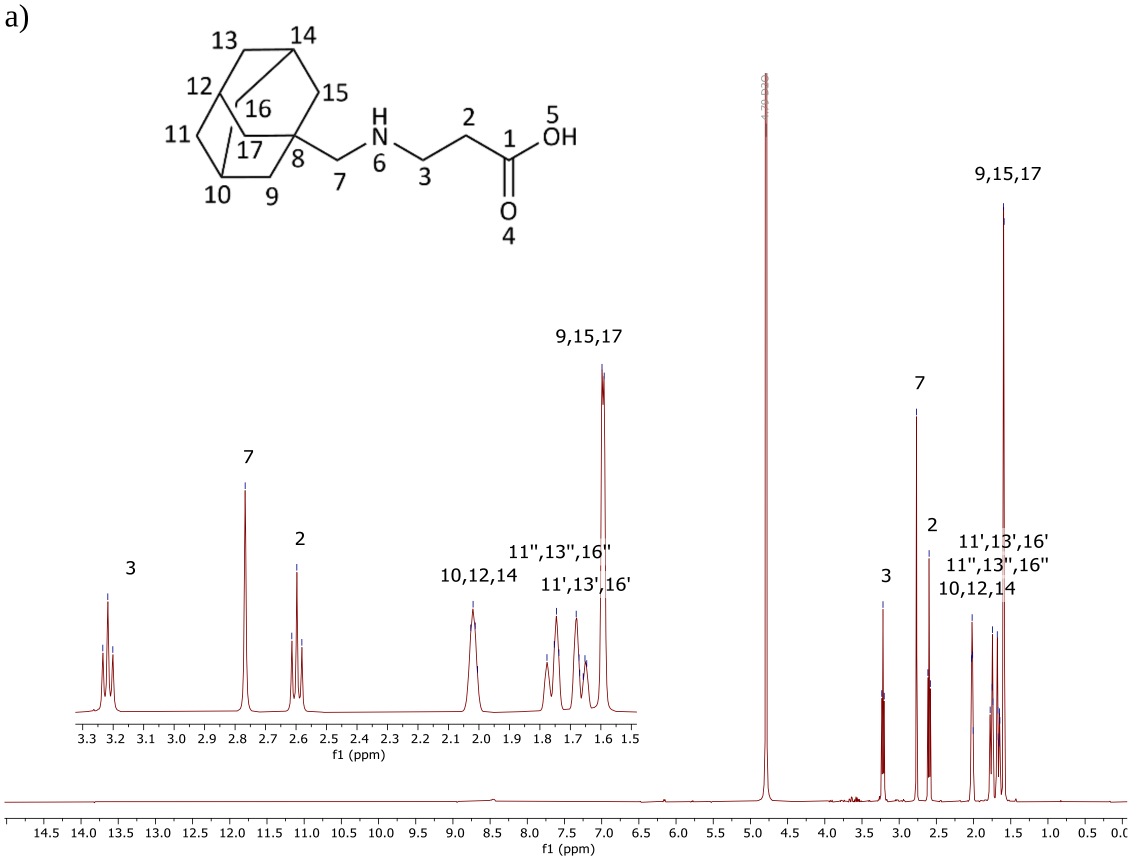
13
14
12
15
16
11
17
8
H
N
6
2
1
5
OH
10
9
7
3
O
4
9,15,17
7
2
3
10,12,14
11'',13'',16''
11',13',16'
f1 (ppm)

b)

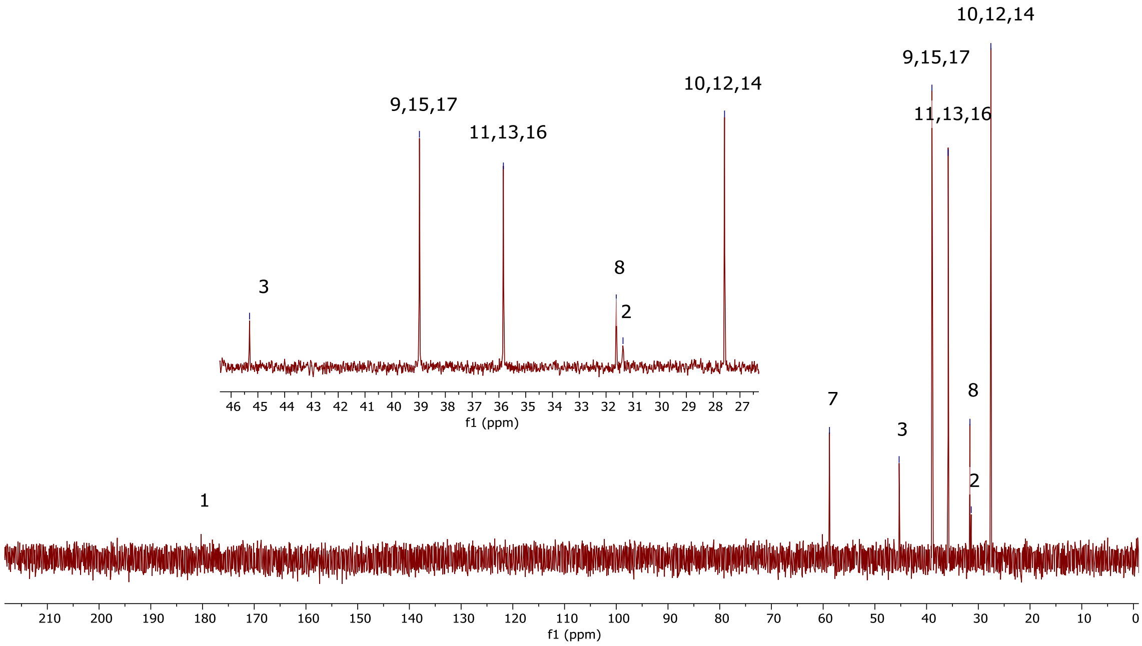
10,12,14
9,15,17
11,13,16
3
8
2
7
1
f1 (ppm)

c)

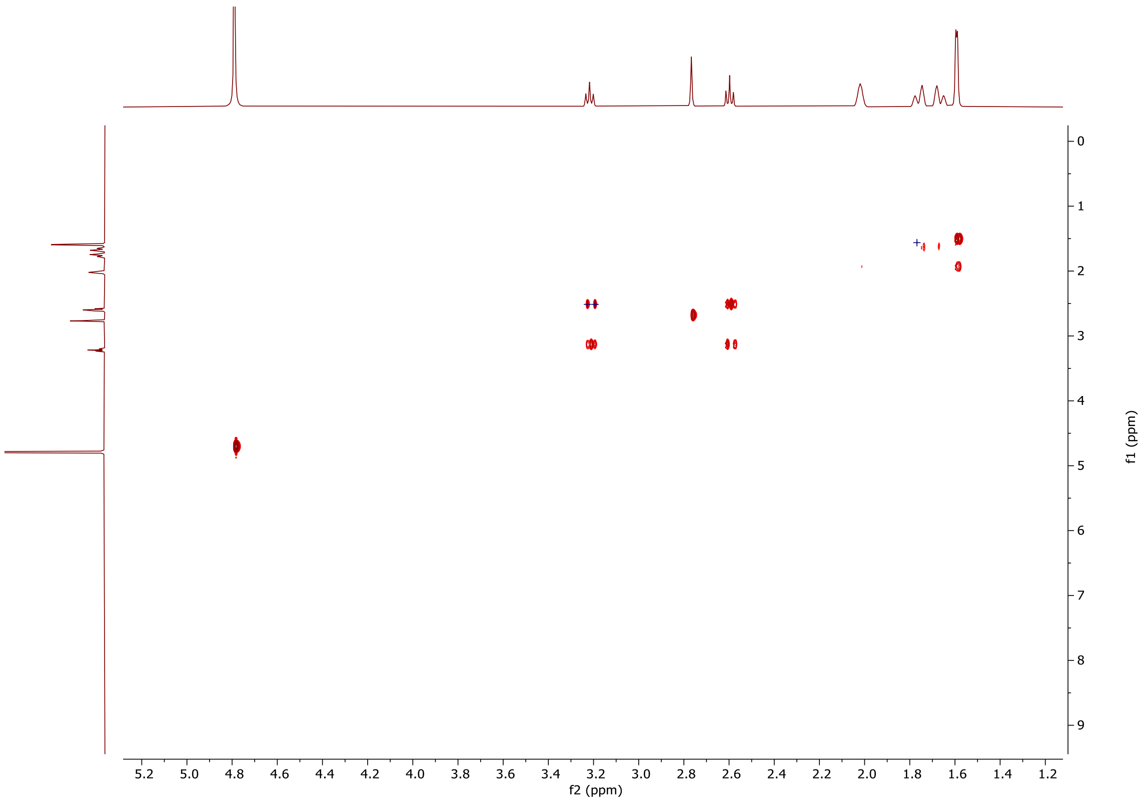

d)

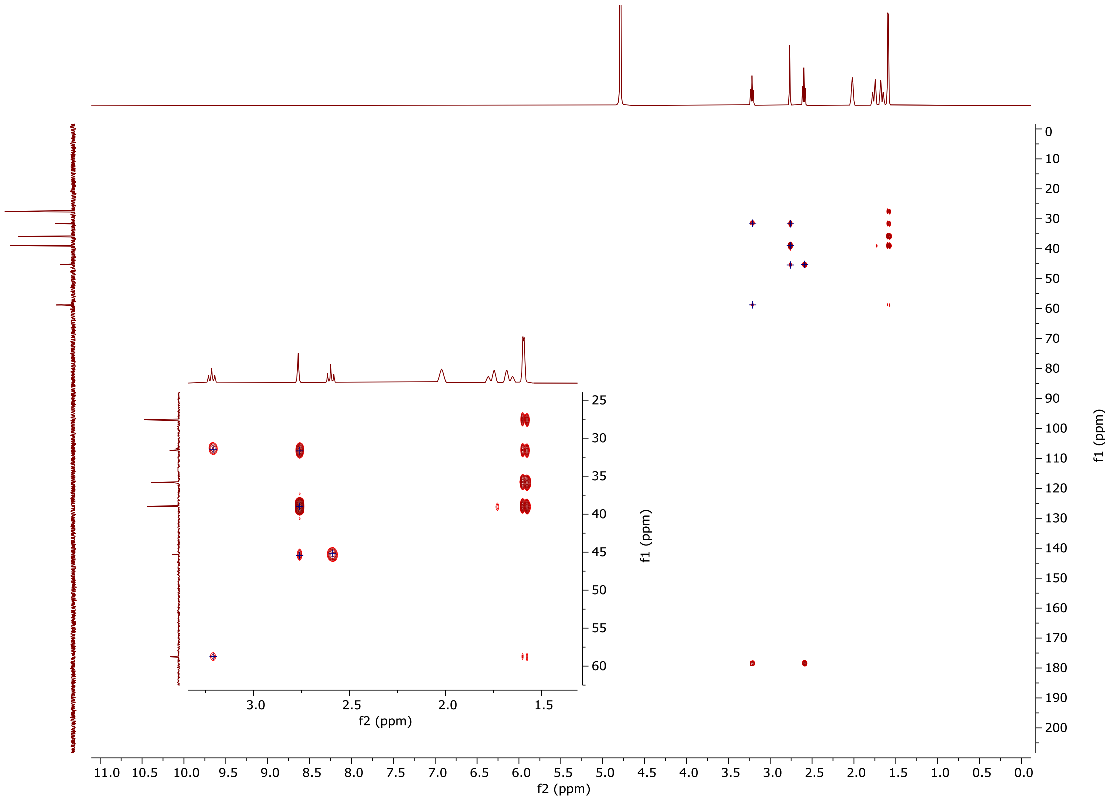

e)

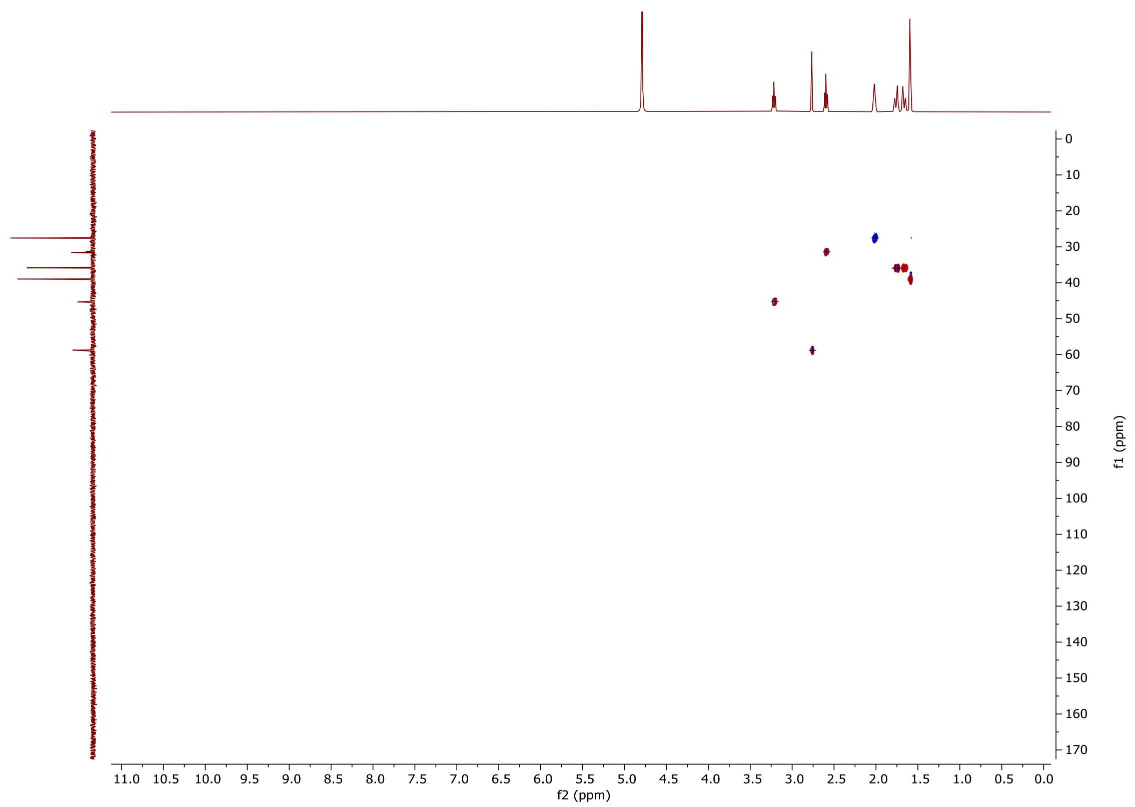

Figure S9. NMR spectra (D_2O) of **5aa**: a) ^{1}H, b) ^{13}C, c) COSY, d) HMBC and e) HSQC.

a)

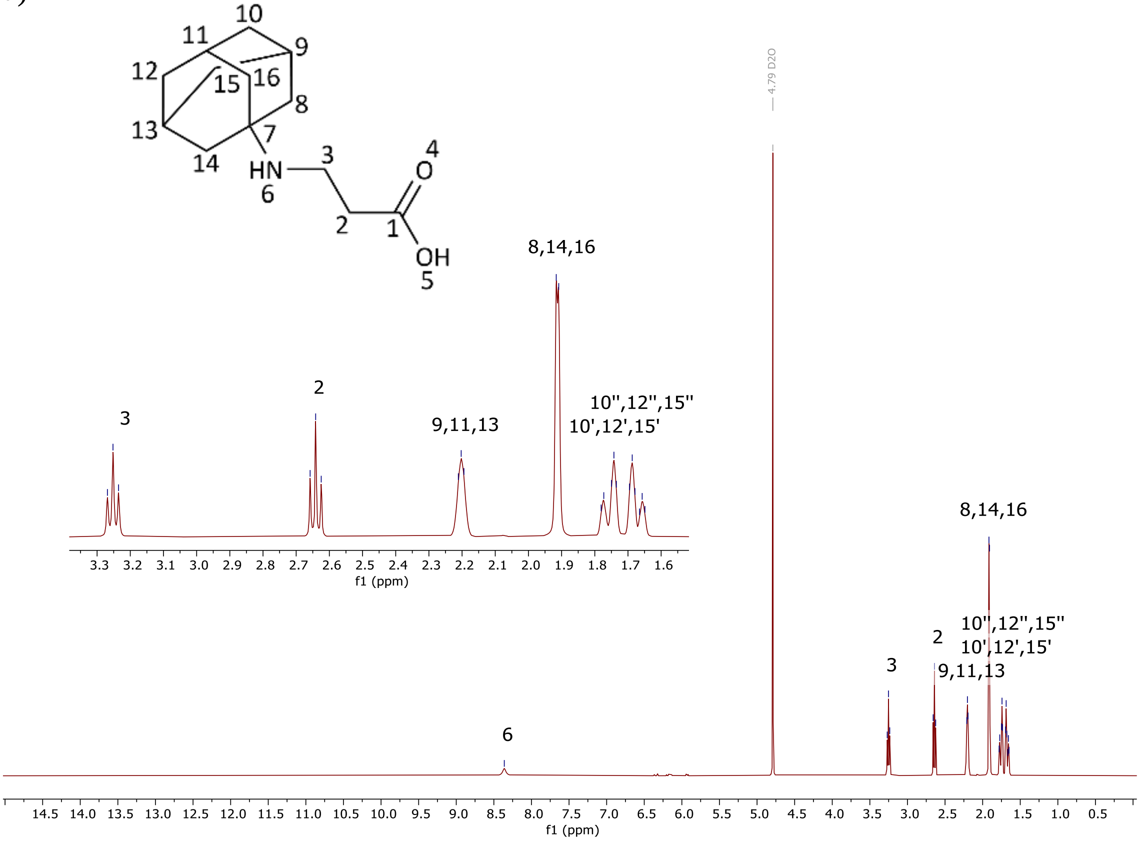

b)

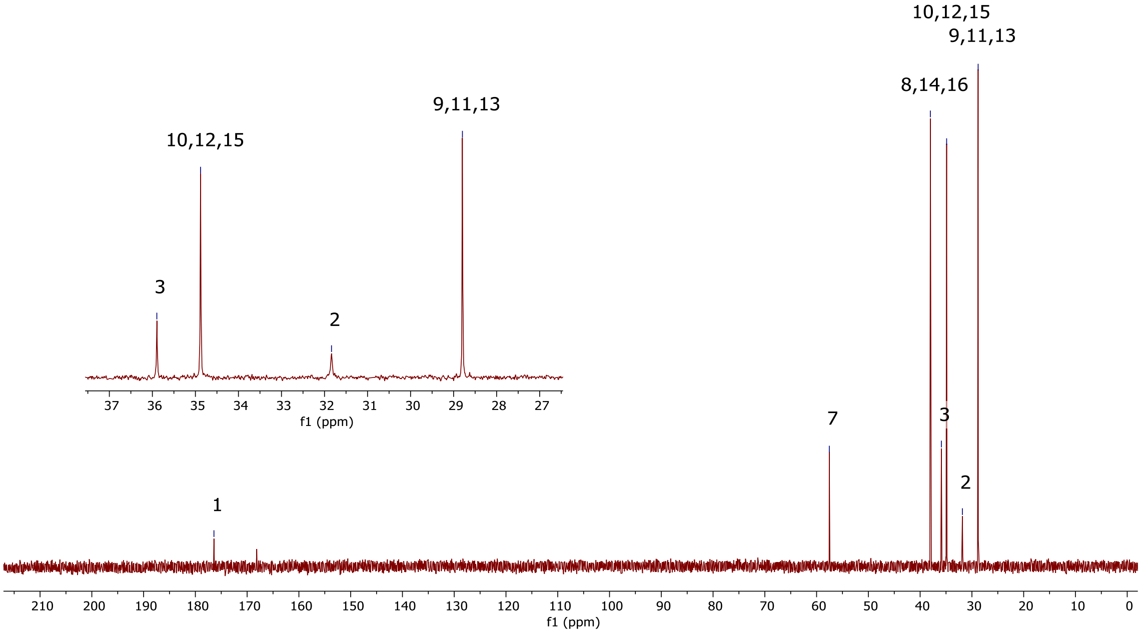
10,12,15
9,11,13
8,14,16
10,12,15
9,11,13
3
2
37 36 35 34 33 32 31 30 29 28 27
f1 (ppm)
7
3
2
1
210 200 190 180 170 160 150 140 130 120 110 100 90 80 70 60 50 40 30 20 10 0
f1 (ppm)

c)

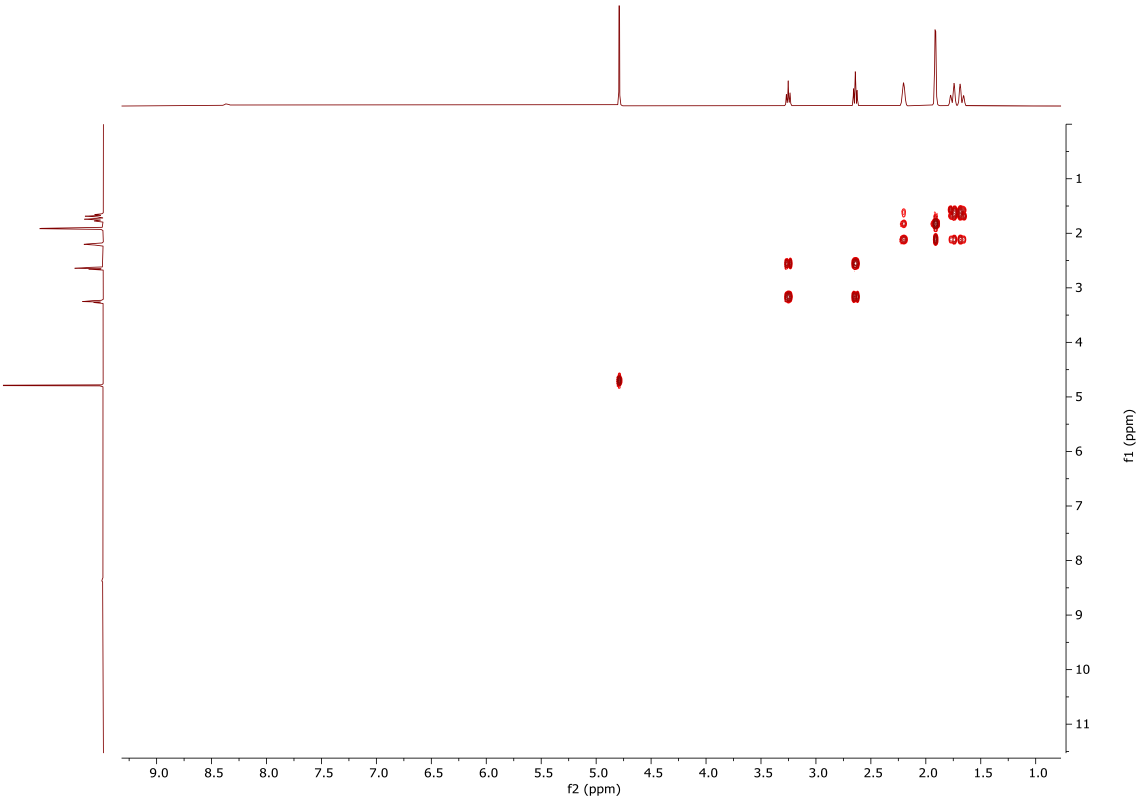
9.0 8.5 8.0 7.5 7.0 6.5 6.0 5.5 5.0 4.5 4.0 3.5 3.0 2.5 2.0 1.5 1.0
f2 (ppm)
1 2 3 4 5 6 7 8 9 10 11
f1 (ppm)

d)

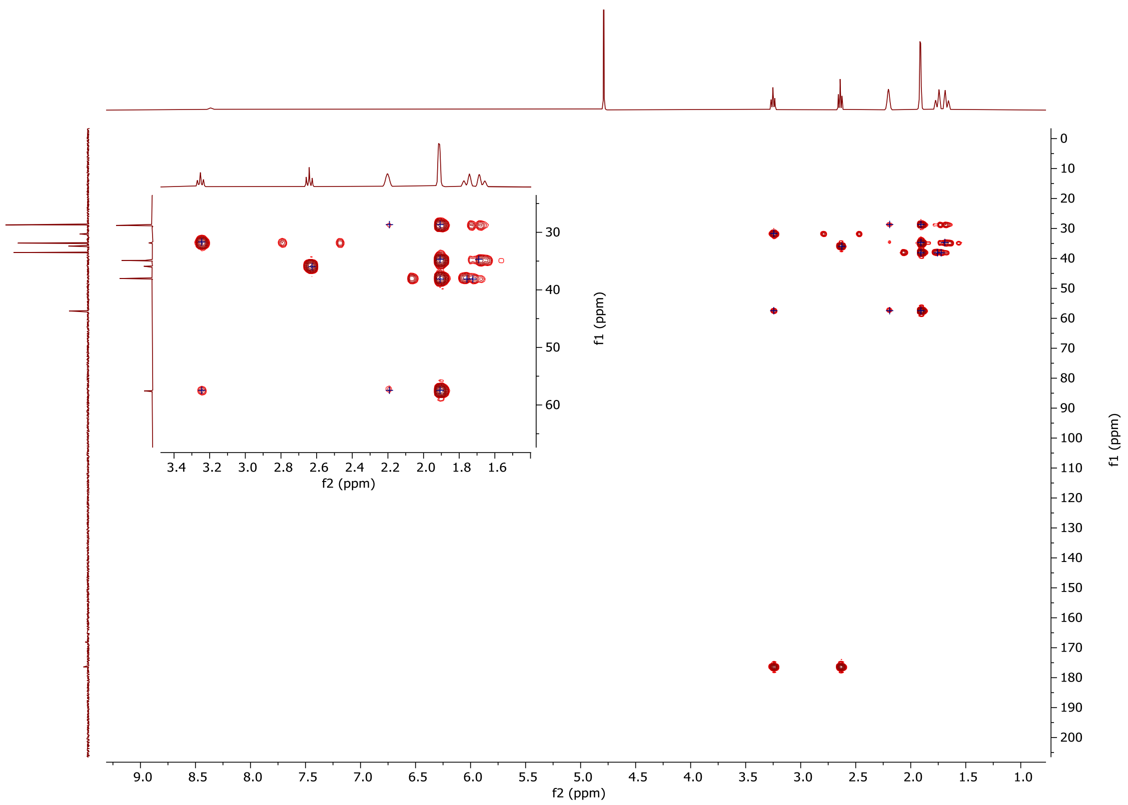

e)

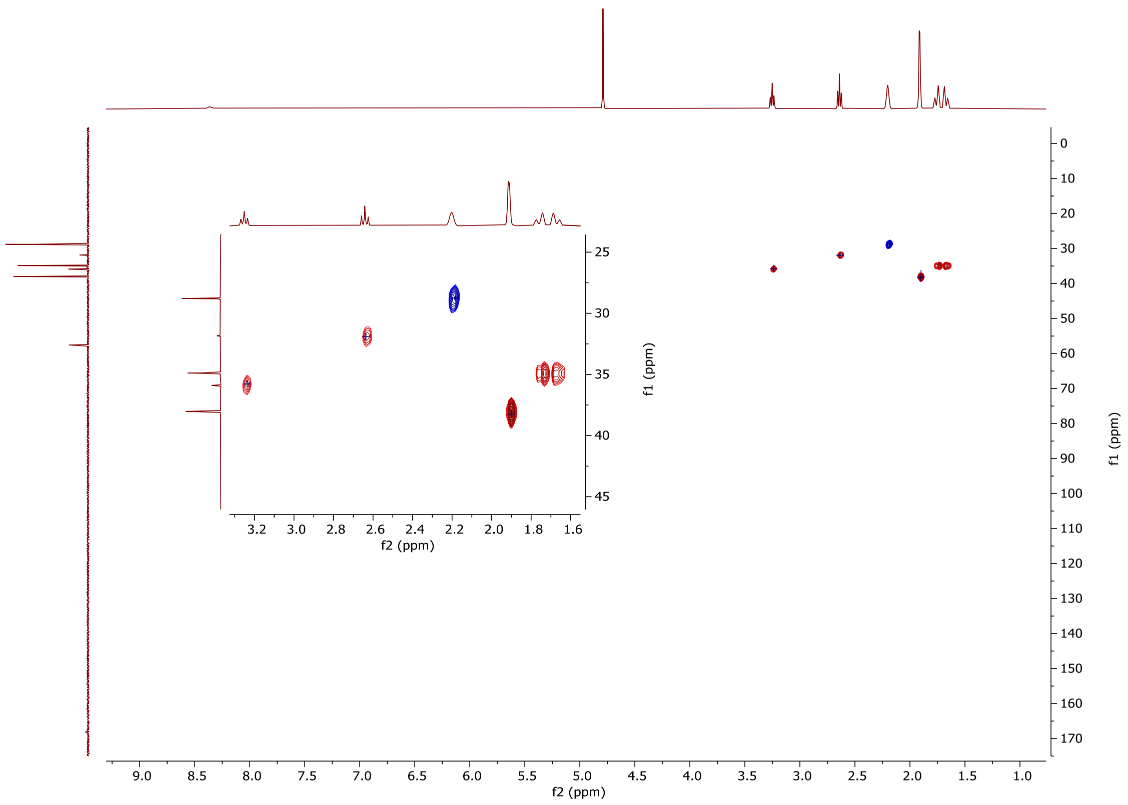

Figure S10. NMR spectra (D_2O) of **5ab**: a) ^{1}H, b) ^{13}C, c) COSY, d) HMBC and e) HSQC.

a)

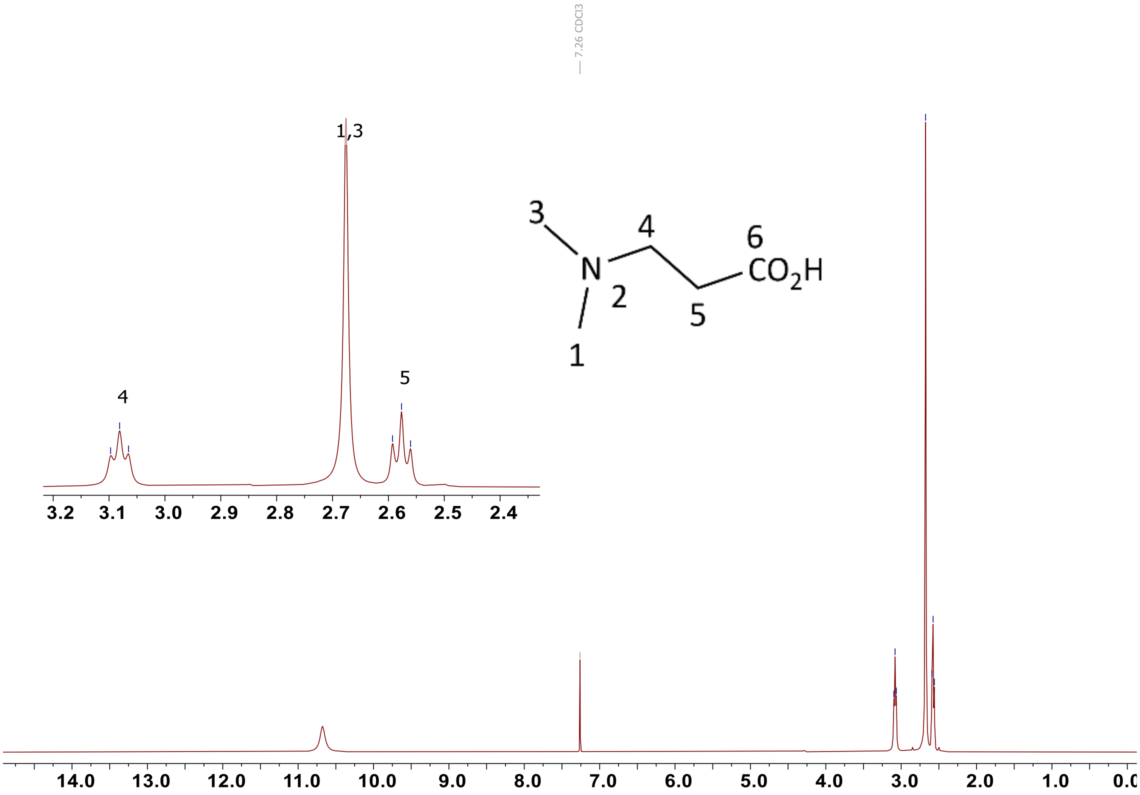
7.26 CDCl3
1,3
4
5
3.2
3.1
3.0
2.9
2.8
2.7
2.6
2.5
2.4
3
4
6
N
2
CO_2H
5
1
14.0
13.0
12.0
11.0
10.0
9.0
8.0
7.0
6.0
5.0
4.0
3.0
2.0
1.0

b)

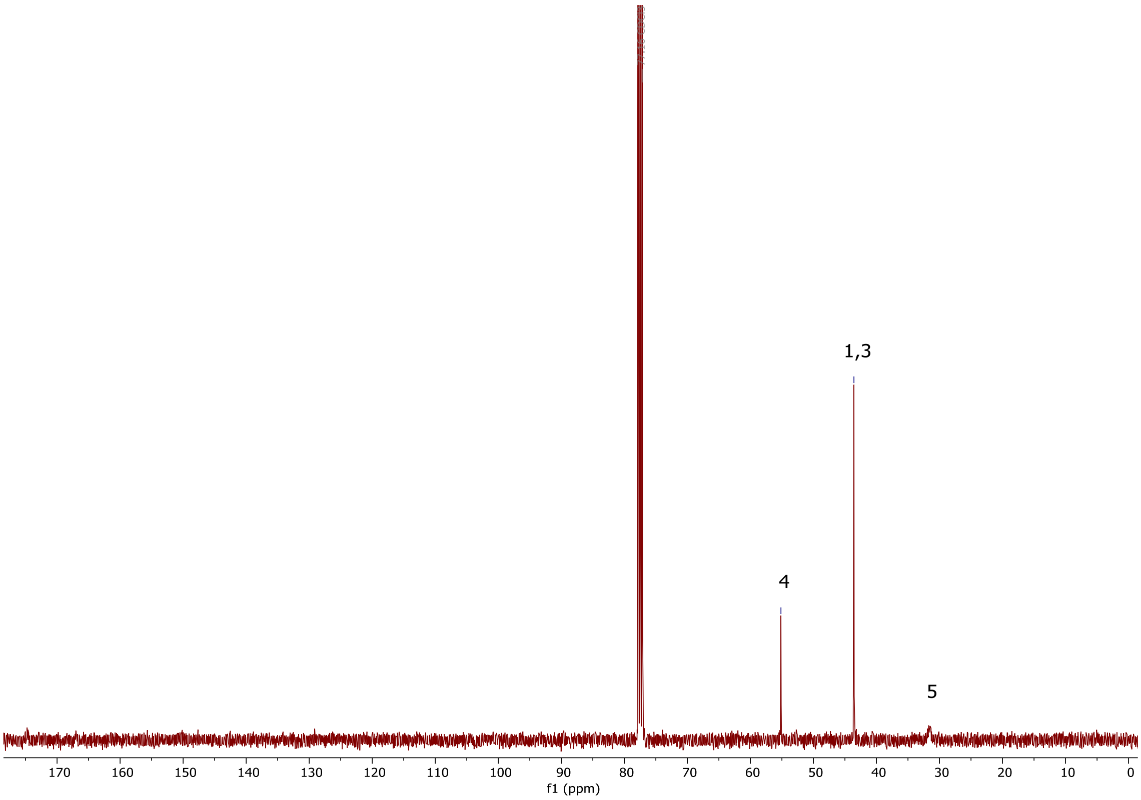
1,3
4
5
170
160
150
140
130
120
110
100
90
80
70
60
50
40
30
20
10
0
f1 (ppm)

c)

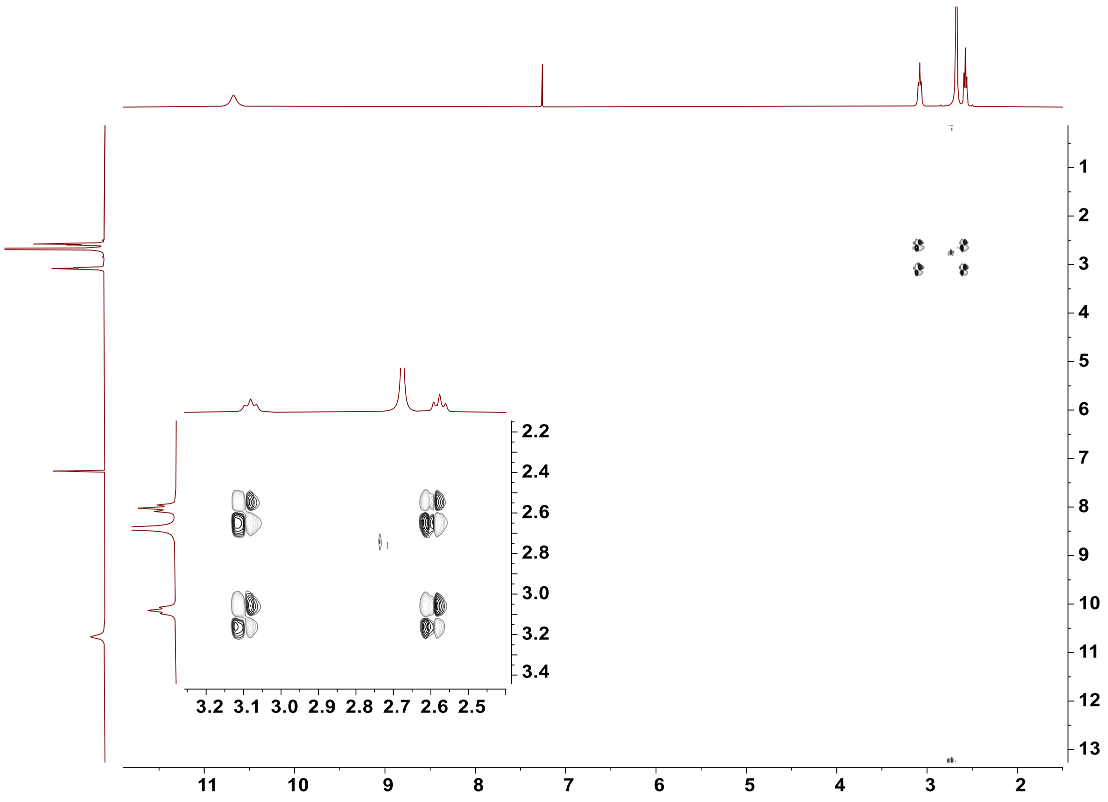

1
2
3
4
5
6
7
8
9
10
11
12
13
11
10
9
8
7
6
5
4
3
2
2.2
2.4
2.6
2.8
3.0
3.2
3.4
3.2 3.1 3.0 2.9 2.8 2.7 2.6 2.5

d)

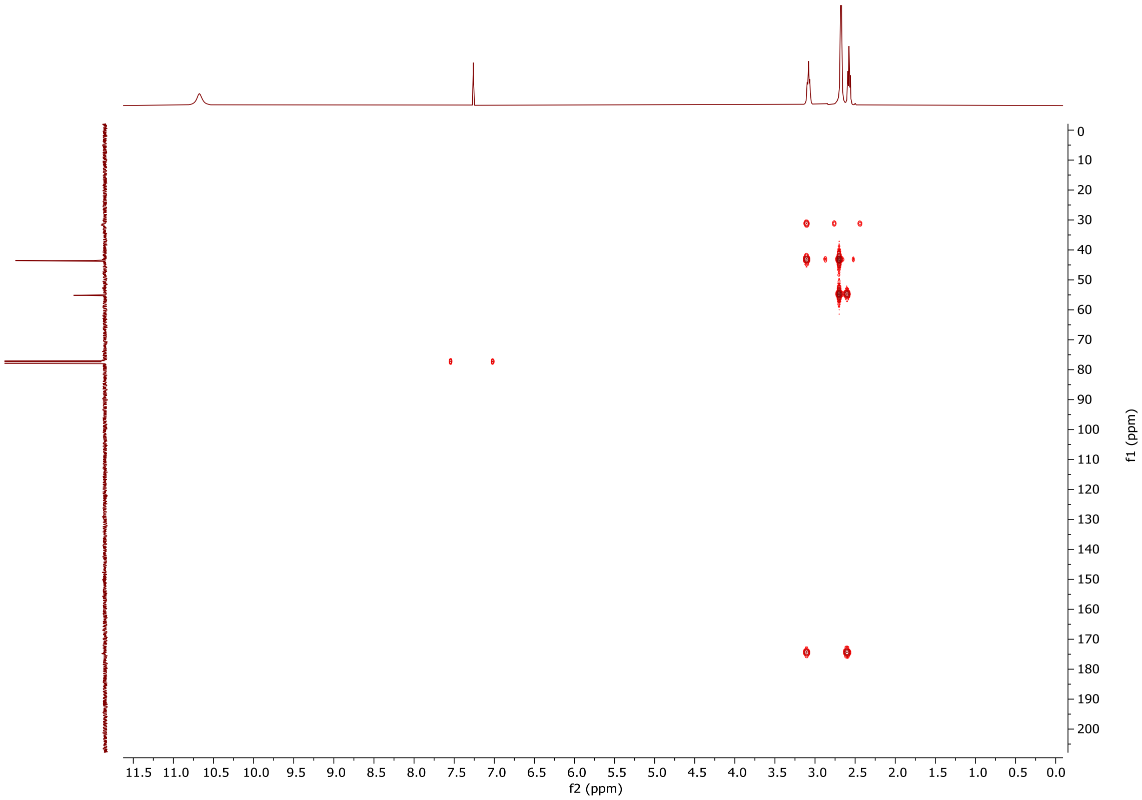

0
10
20
30
40
50
60
70
80
90
100
110
120
130
140
150
160
170
180
190
200
f1 (ppm)
11.5 11.0 10.5 10.0 9.5 9.0 8.5 8.0 7.5 7.0 6.5 6.0 5.5 5.0 4.5 4.0 3.5 3.0 2.5 2.0 1.5 1.0 0.5 0.0
f2 (ppm)

e)

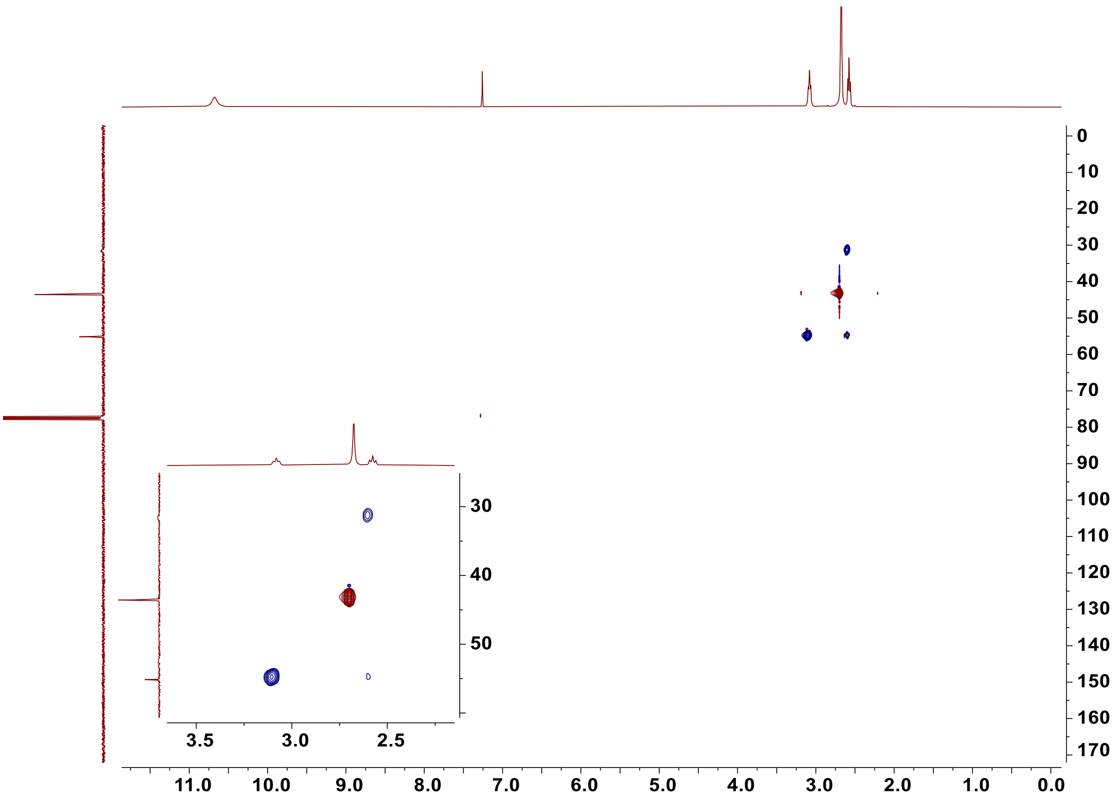

Figure S11. NMR spectra ($CDCl_3$) of **7a**: a) ^1H, b) ^{13}C, c) COSY, d) HMBC and e) HSQC.

a)

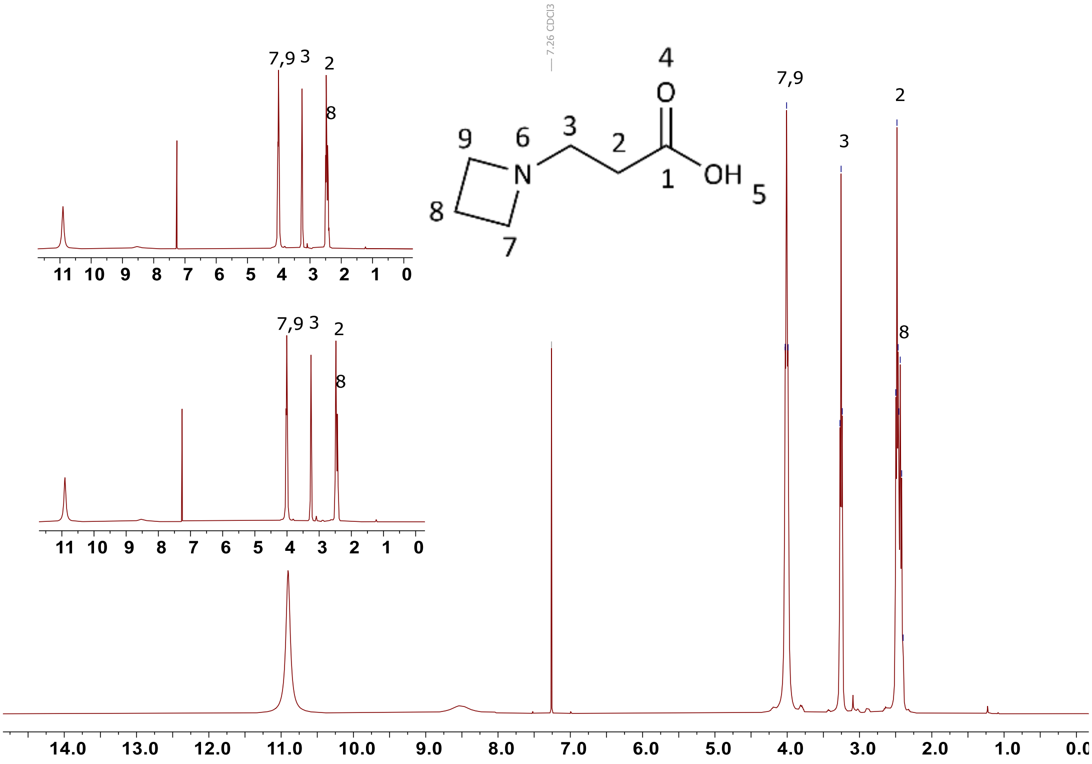

b)

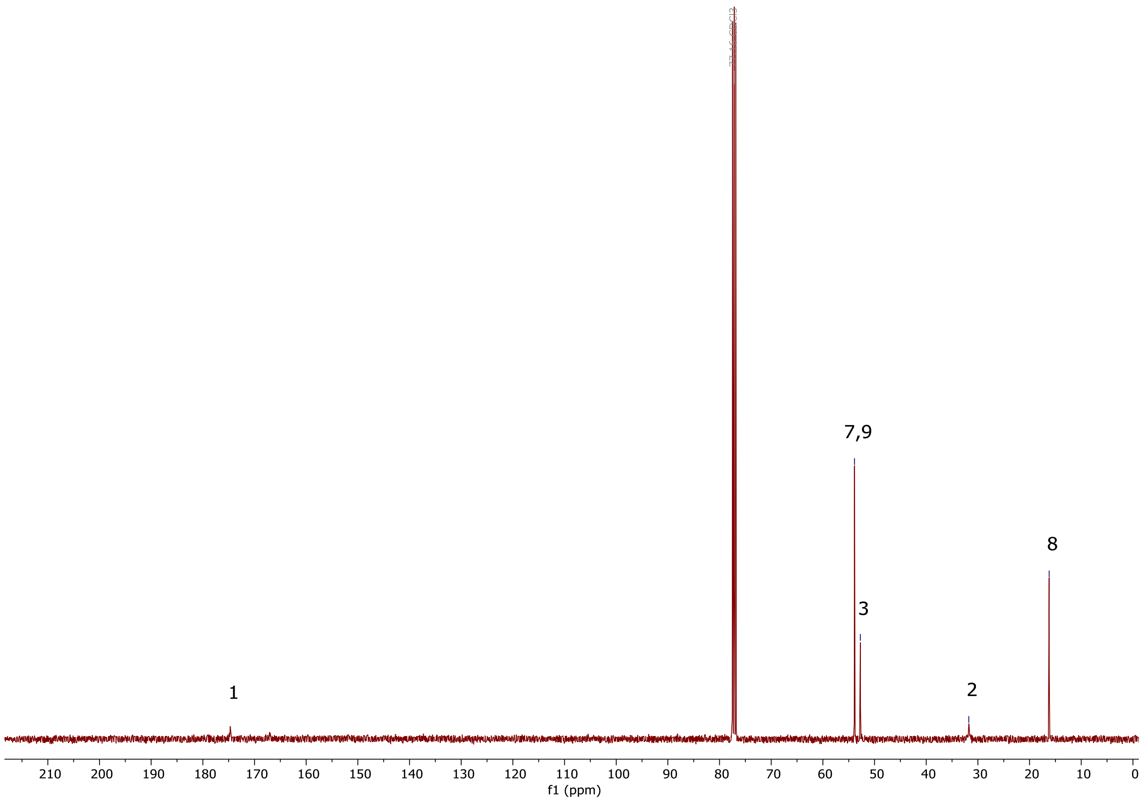
7,9
8
3
1
2
210 200 190 180 170 160 150 140 130 120 110 100 90 80 70 60 50 40 30 20 10 0
f1 (ppm)

c)

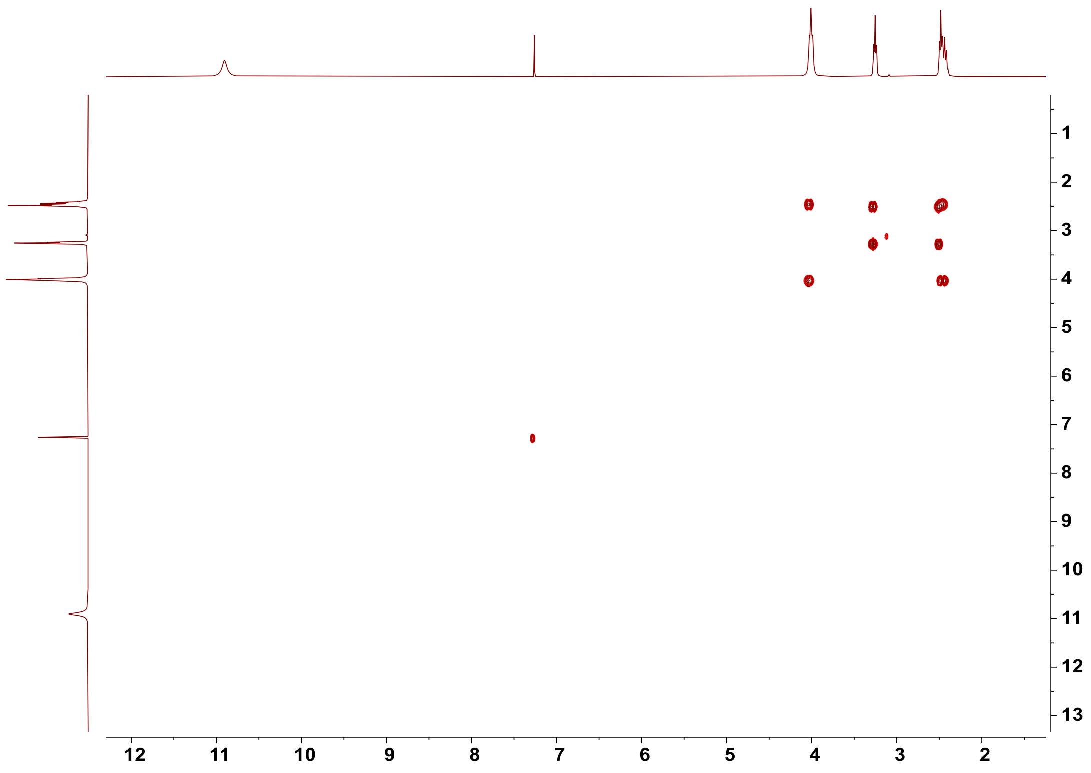
12 11 10 9 8 7 6 5 4 3 2
1 2 3 4 5 6 7 8 9 10 11 12 13

d)

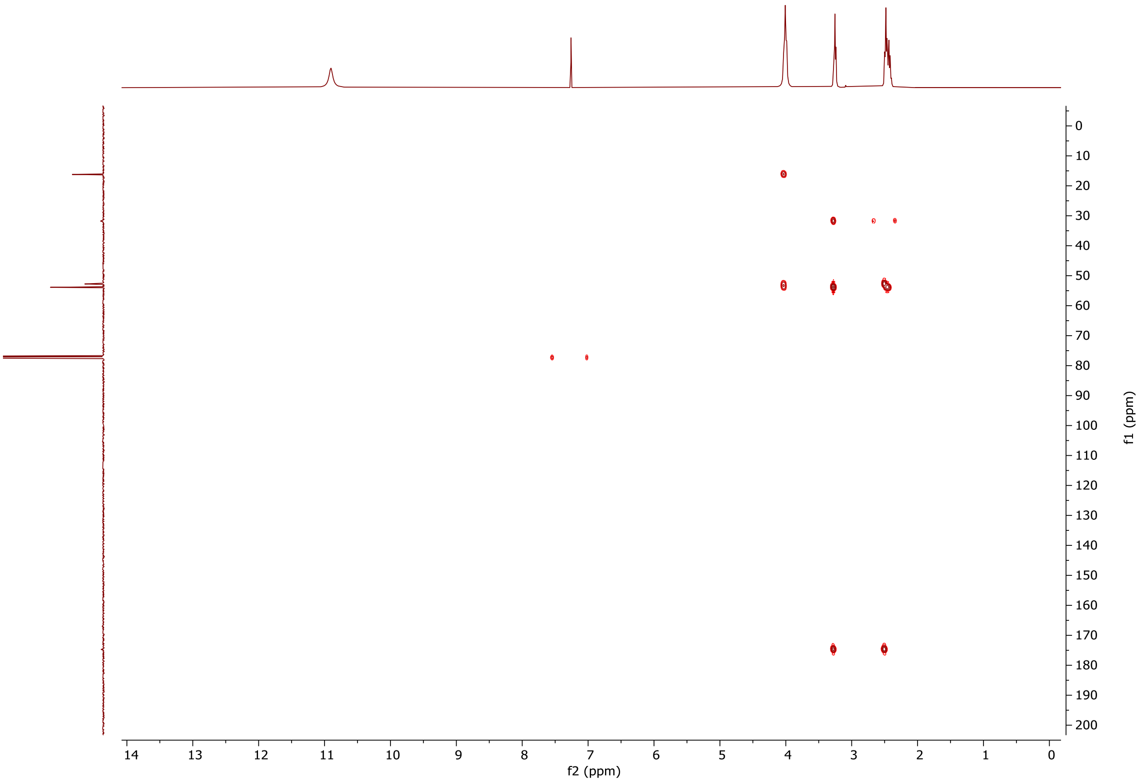

e)

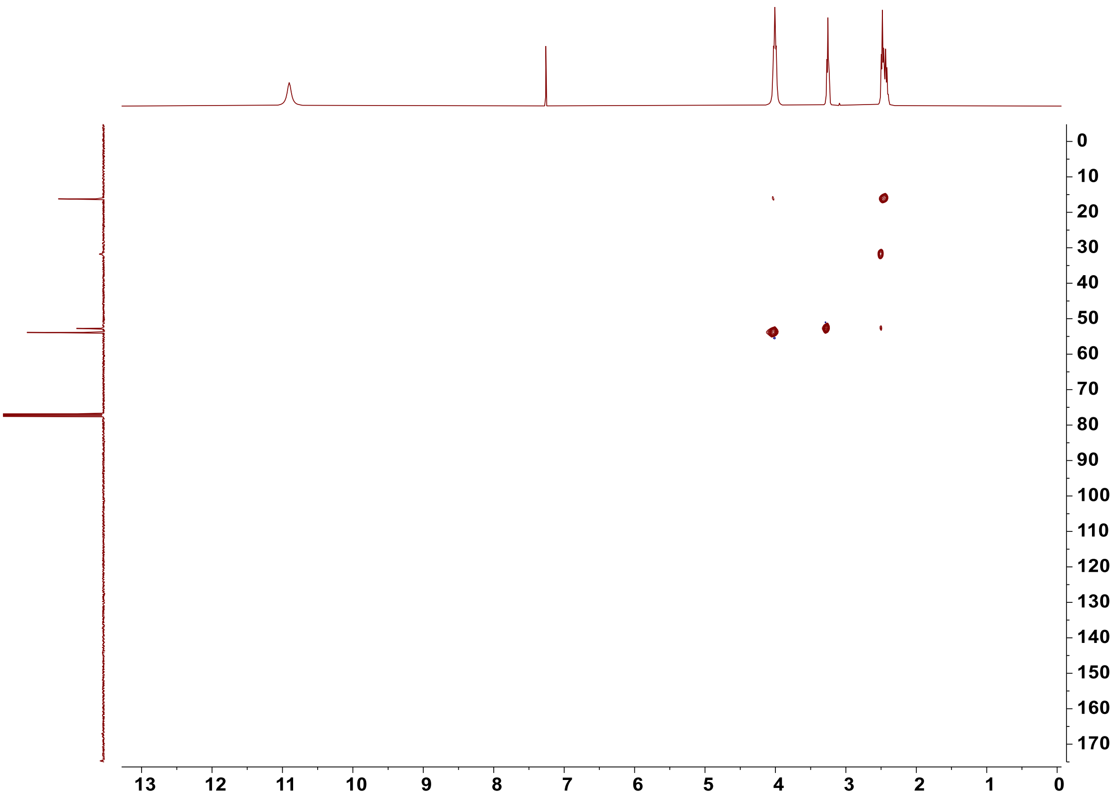

Figure S12. NMR spectra ($CDCl_3$) of **7b**: a) ^{1}H, b) ^{13}C, c) COSY, d) HMBC and e) HSQC.

a)

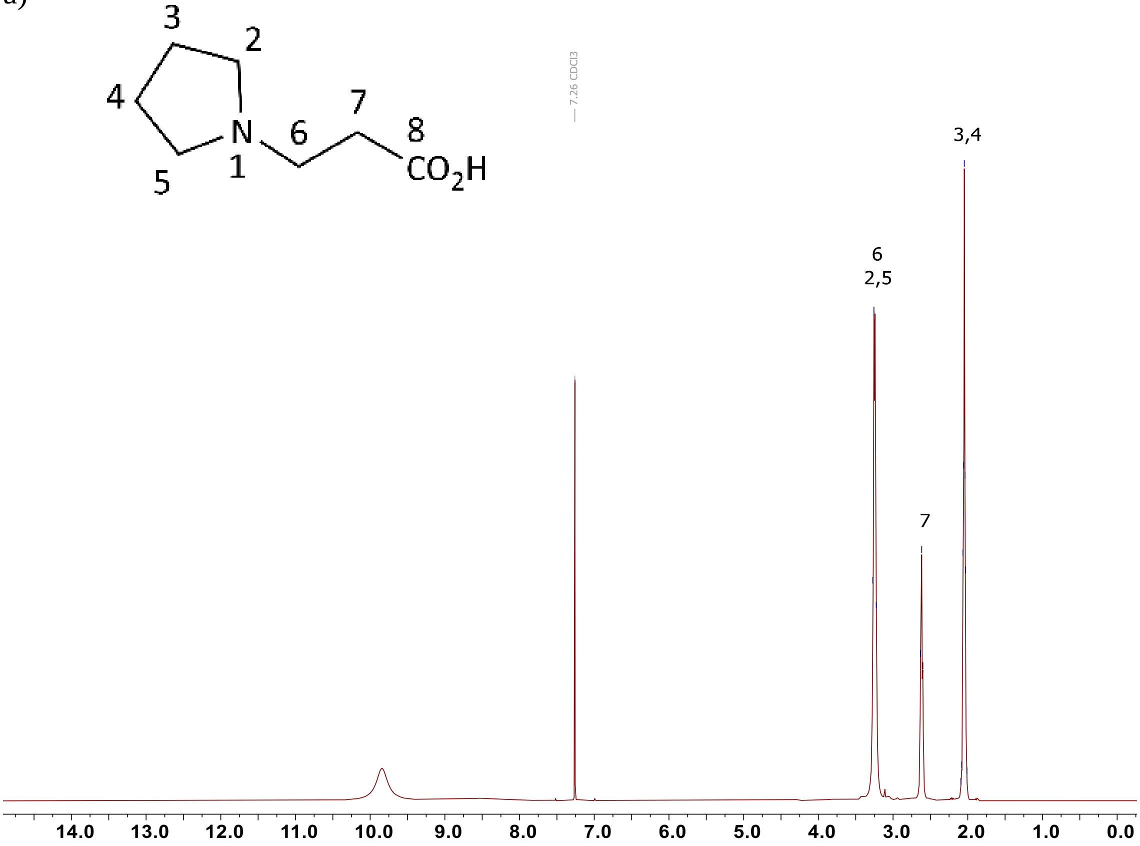
3
2
4
7
N
6
8
5
1
CO_2H
7.26 CDCl3
3,4
6
2,5
7
14.0 13.0 12.0 11.0 10.0 9.0 8.0 7.0 6.0 5.0 4.0 3.0 2.0 1.0 0.0

b)

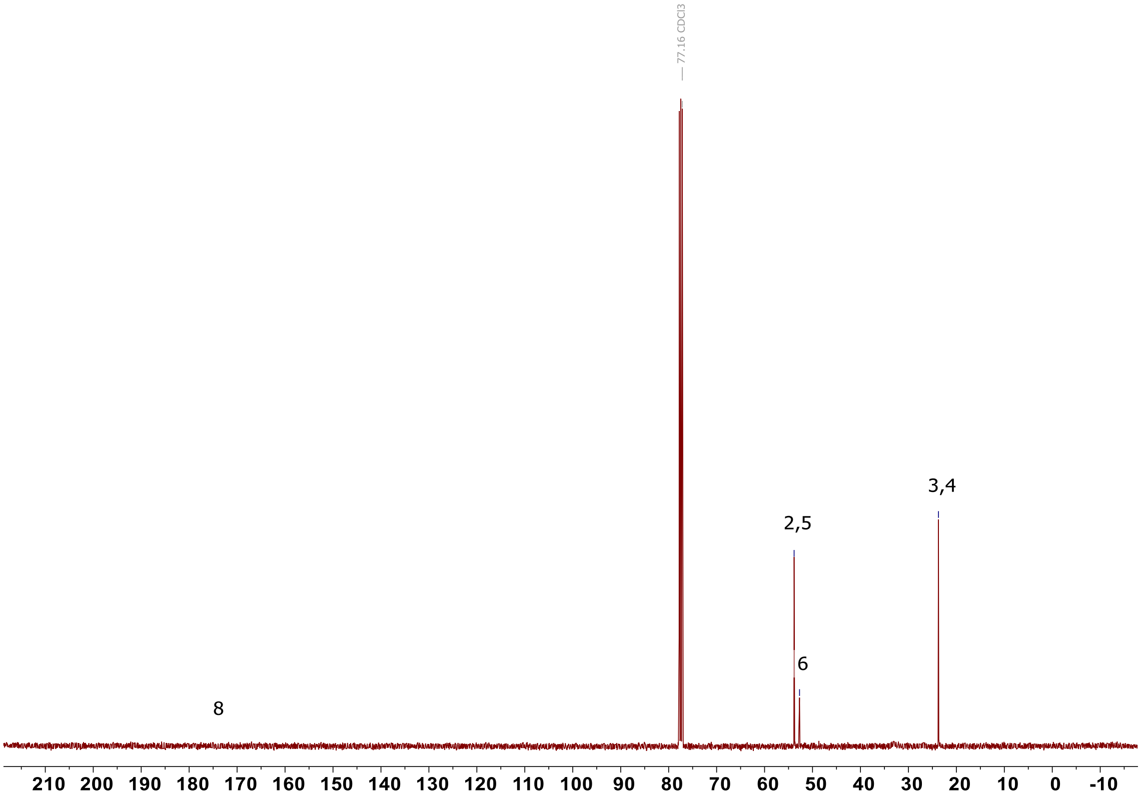
77.16 CDCl3
3,4
2,5
6
8
210 200 190 180 170 160 150 140 130 120 110 100 90 80 70 60 50 40 30 20 10 0 -10

c)

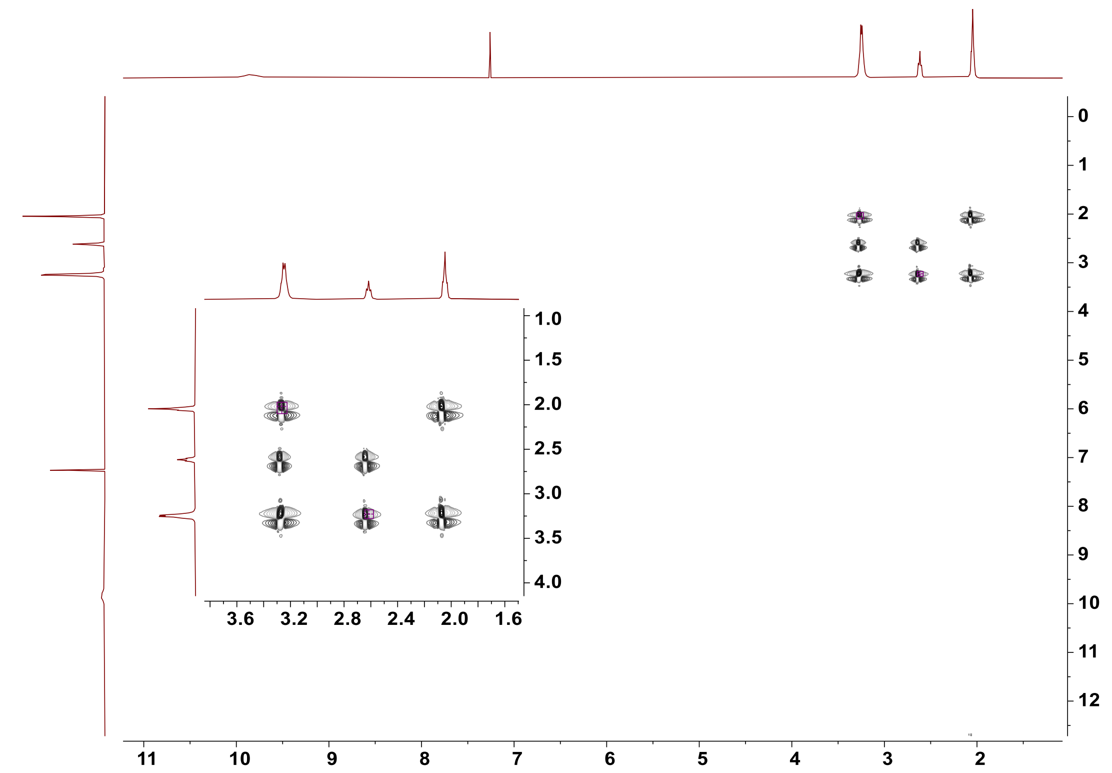

11 10 9 8 7 6 5 4 3 2
0 1 2 3 4 5 6 7 8 9 10 11 12
3.6 3.2 2.8 2.4 2.0 1.6
1.0 1.5 2.0 2.5 3.0 3.5 4.0

d)

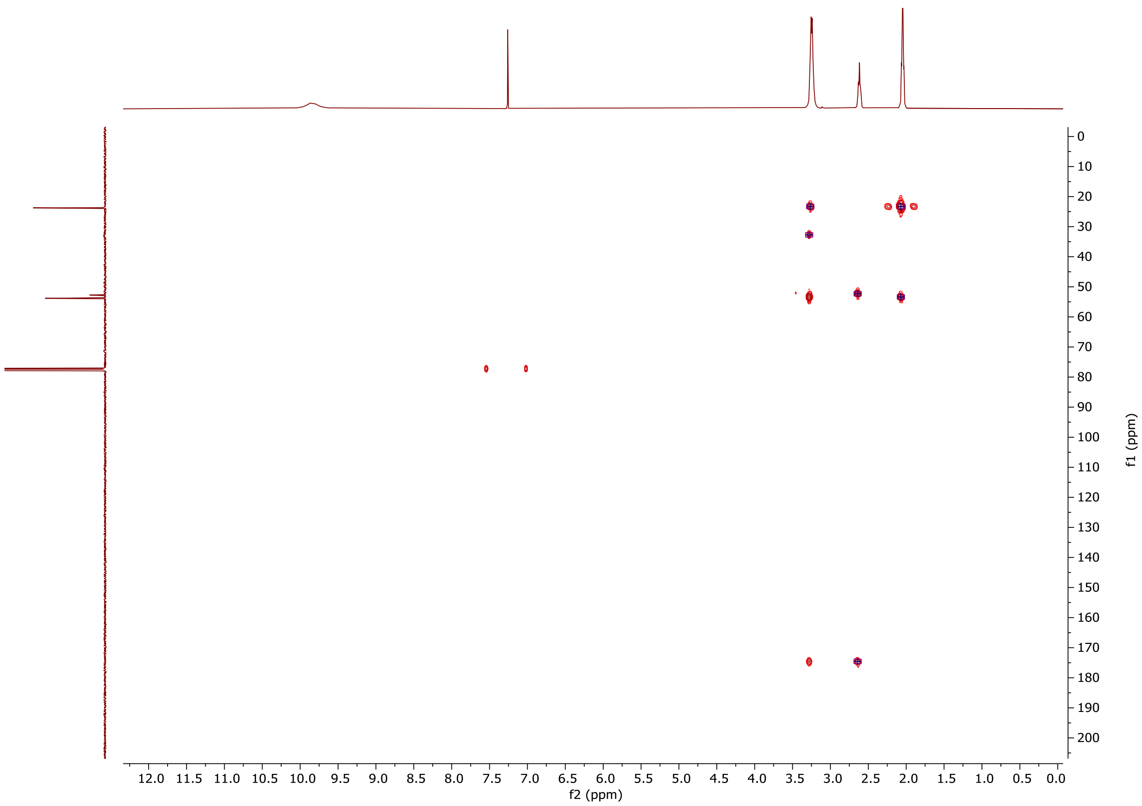

12.0 11.5 11.0 10.5 10.0 9.5 9.0 8.5 8.0 7.5 7.0 6.5 6.0 5.5 5.0 4.5 4.0 3.5 3.0 2.5 2.0 1.5 1.0 0.5 0.0
f2 (ppm)
0 10 20 30 40 50 60 70 80 90 100 110 120 130 140 150 160 170 180 190 200
f1 (ppm)

e)

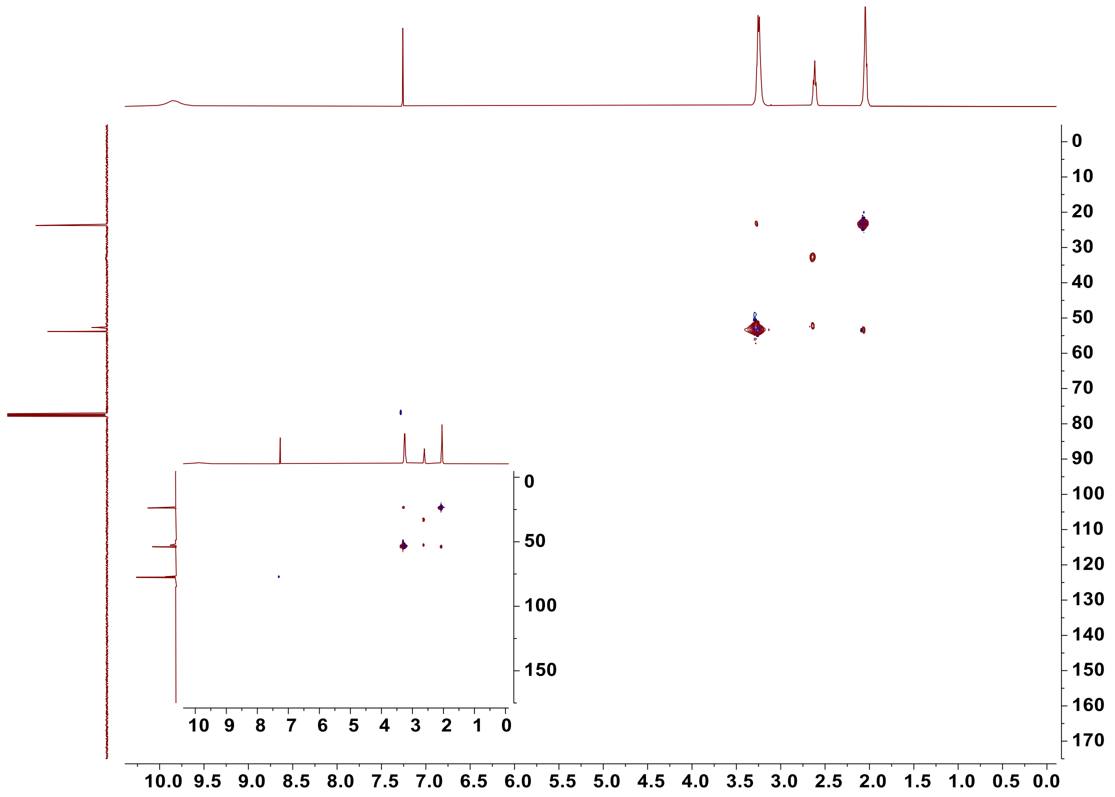

Figure S20. NMR spectra ($CDCl_3$) of **7c**: a) ^1H, b) ^{13}C, c) COSY, d) HMBC and e) HSQC.

a)

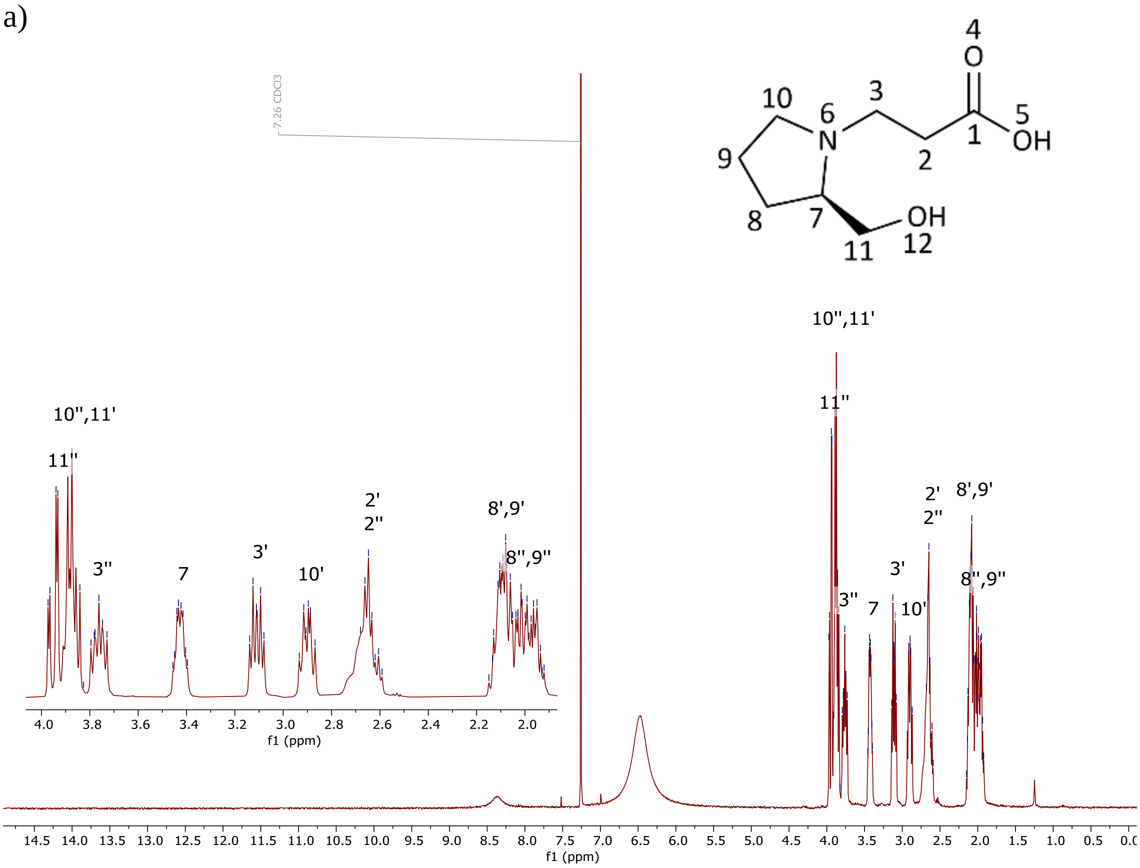

b)

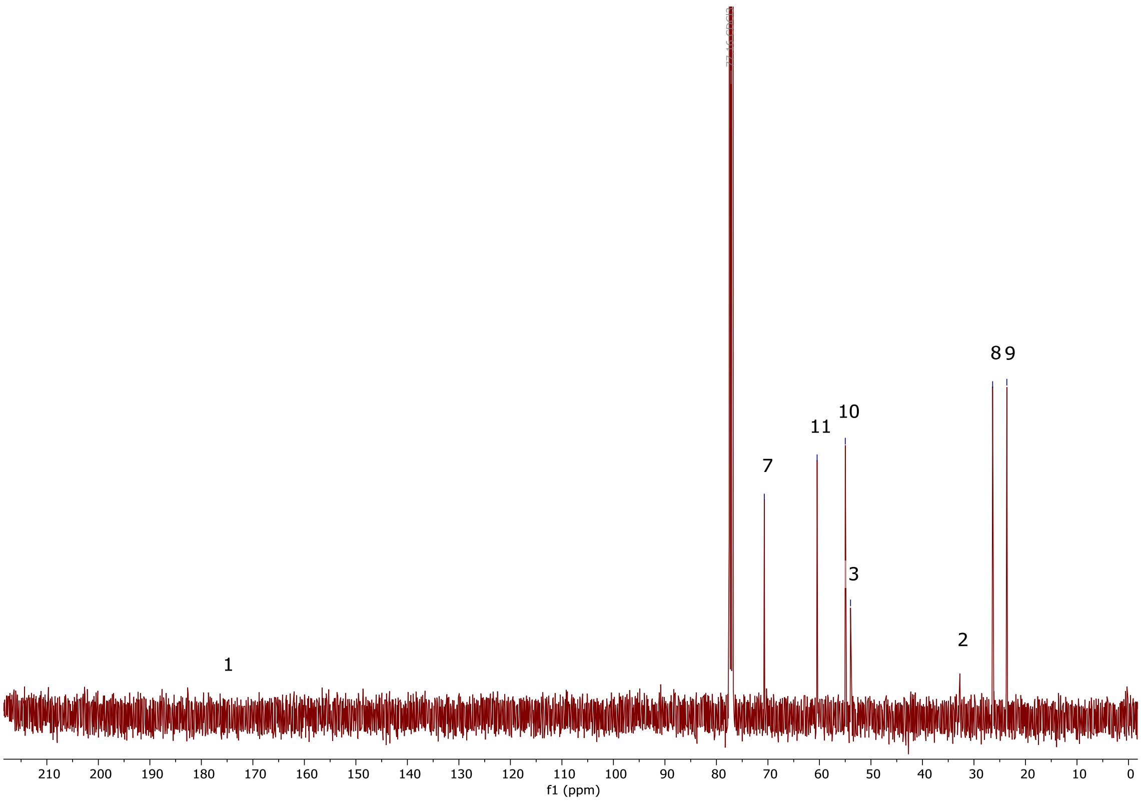

1
2
3
7
11
10
8 9
210 200 190 180 170 160 150 140 130 120 110 100 90 80 70 60 50 40 30 20 10 0
f1 (ppm)

c)

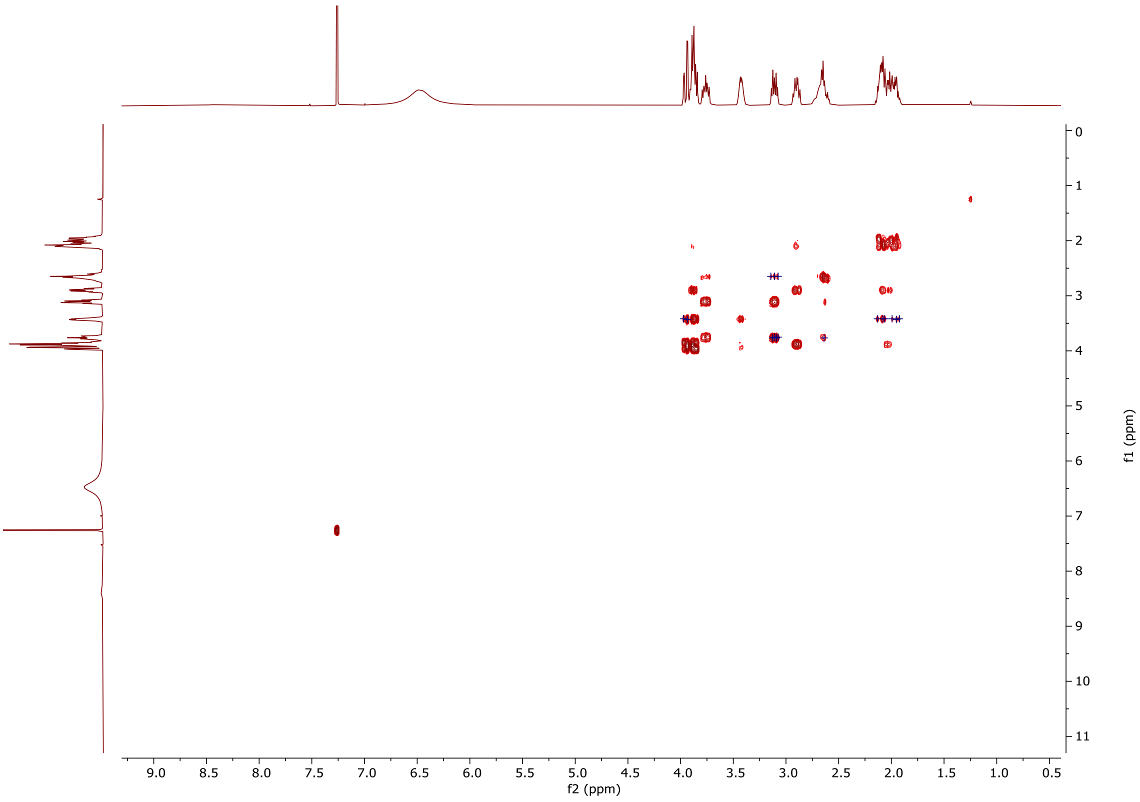

9.0 8.5 8.0 7.5 7.0 6.5 6.0 5.5 5.0 4.5 4.0 3.5 3.0 2.5 2.0 1.5 1.0 0.5
f2 (ppm)
0 1 2 3 4 5 6 7 8 9 10 11
f1 (ppm)

d)

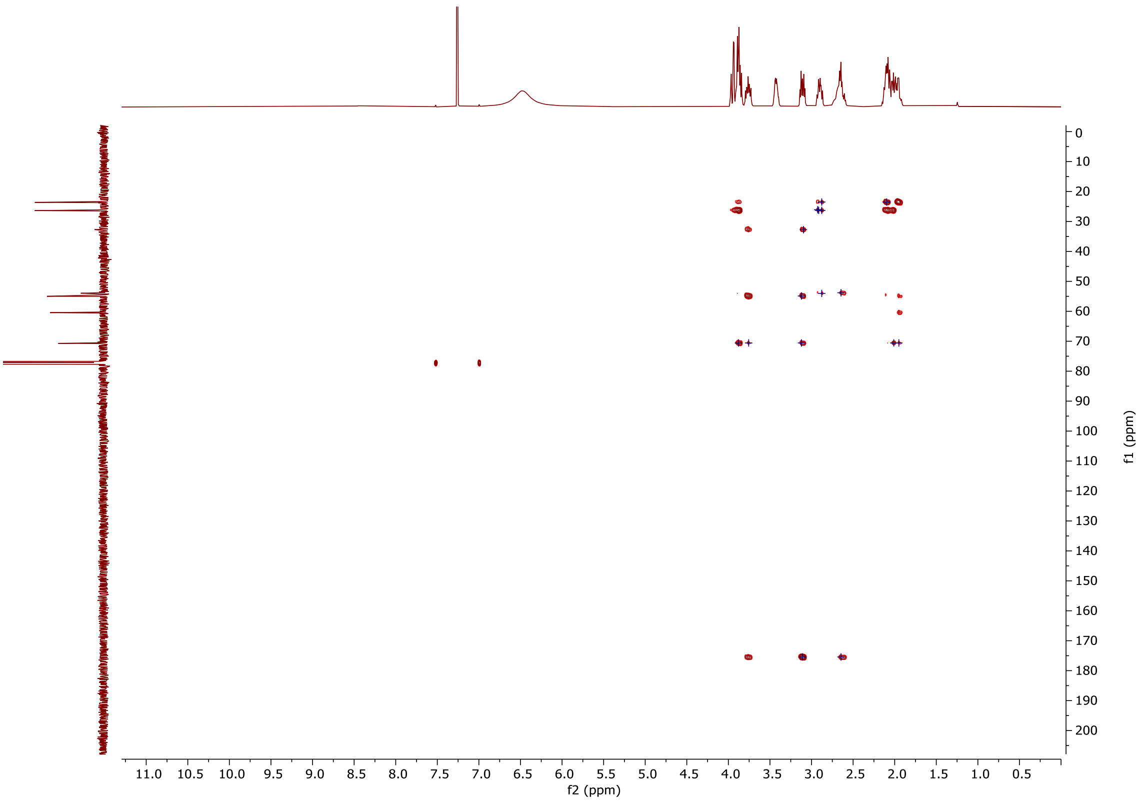

e)

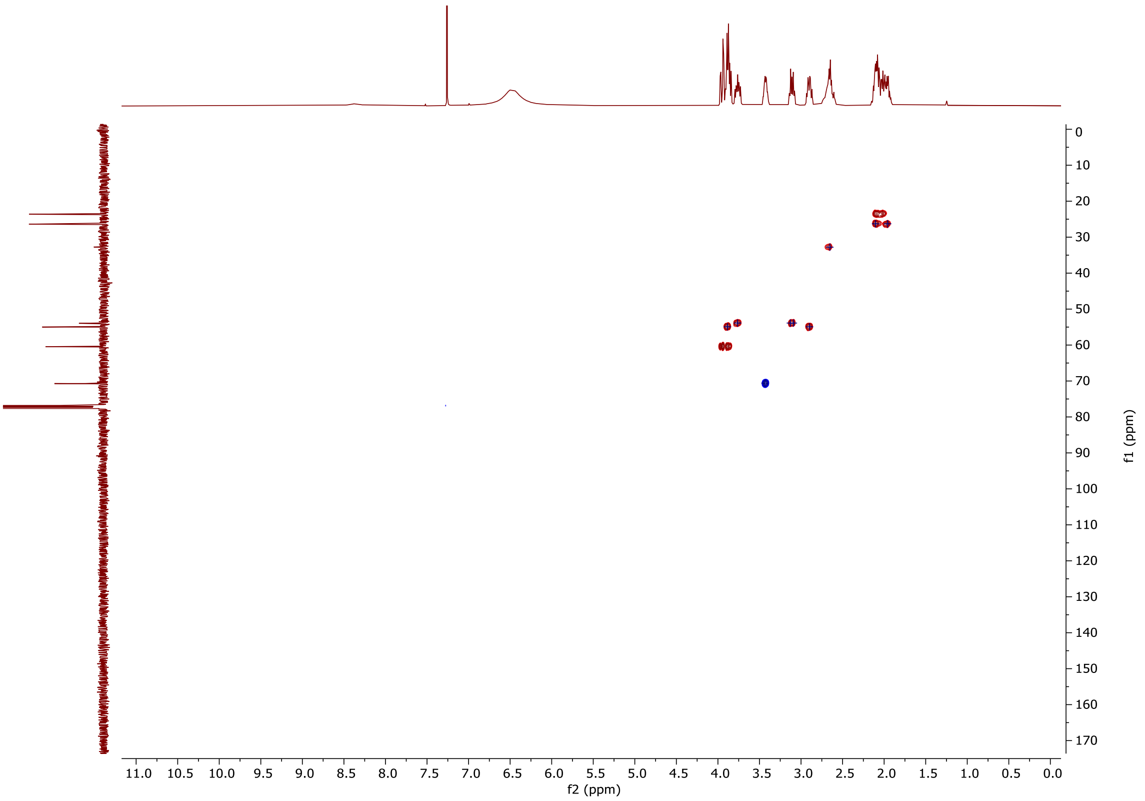

Figure S20. NMR spectra ($CDCl_3$) of **7d**: a) ^{1}H, b) ^{13}C, c) COSY, d) HMBC and e) HSQC.

a)

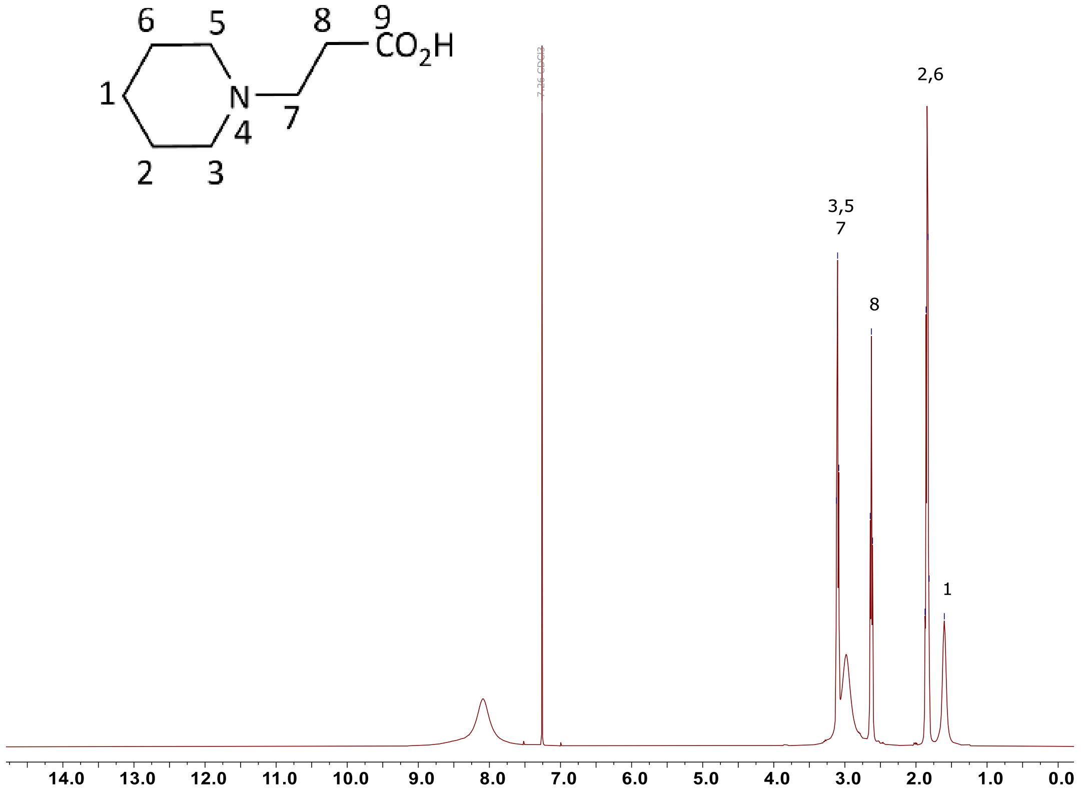

6
5
8
9
CO_2H
1
N
7
4
2
3
7.26 CDCl3
2,6
3,5
7
8
1
14.0
13.0
12.0
11.0
10.0
9.0
8.0
7.0
6.0
5.0
4.0
3.0
2.0
1.0
0.0

b)

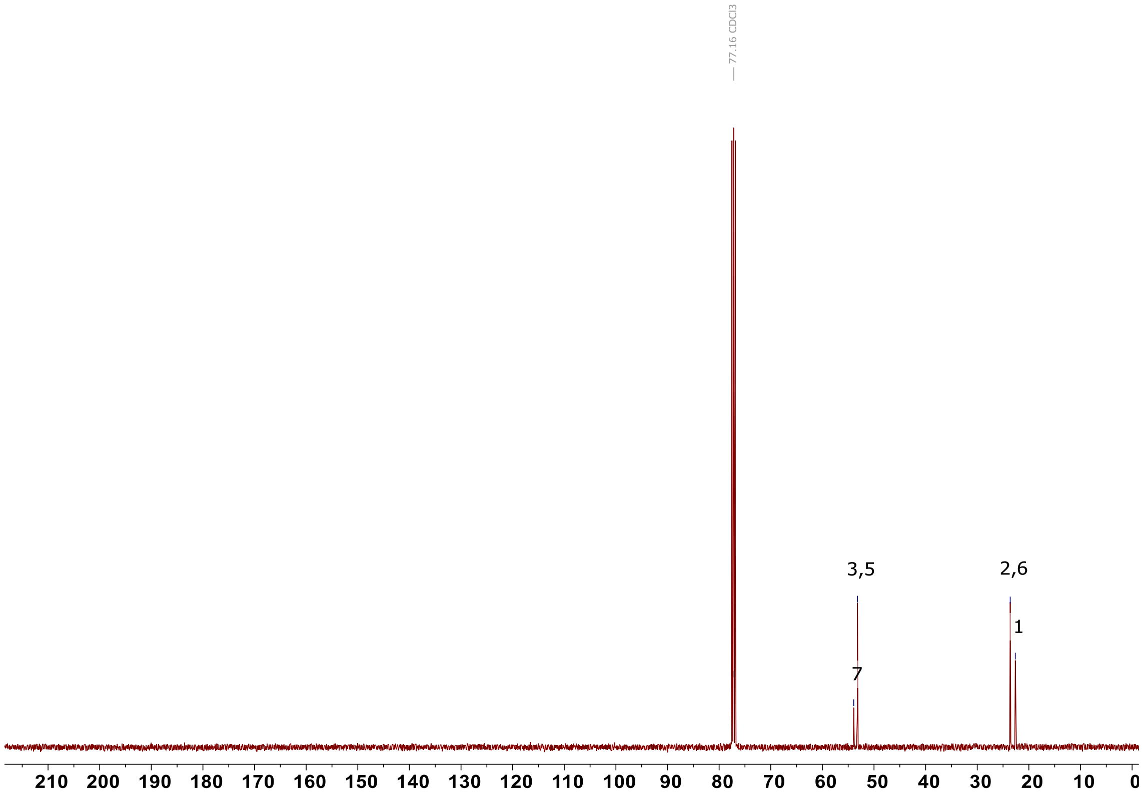

77.16 CDCl3
3,5
2,6
1
7
210
200
190
180
170
160
150
140
130
120
110
100
90
80
70
60
50
40
30
20
10
0

c)

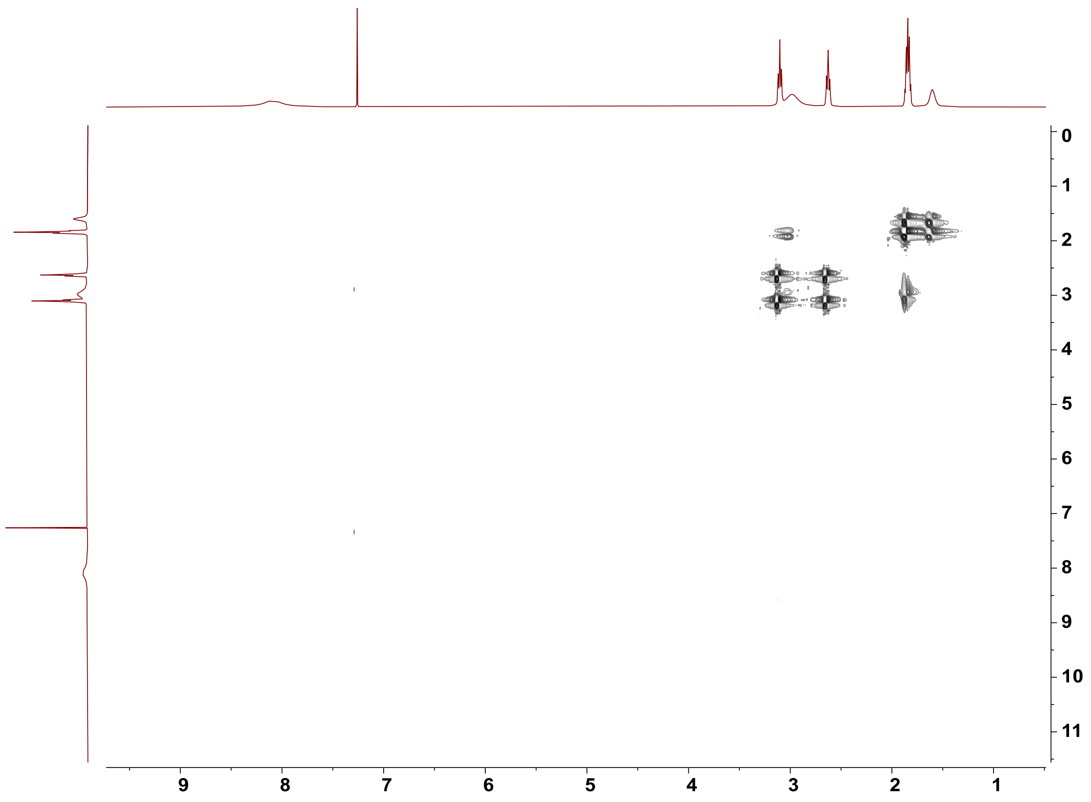
0
1
2
3
4
5
6
7
8
9
10
11
9
8
7
6
5
4
3
2
1

d)

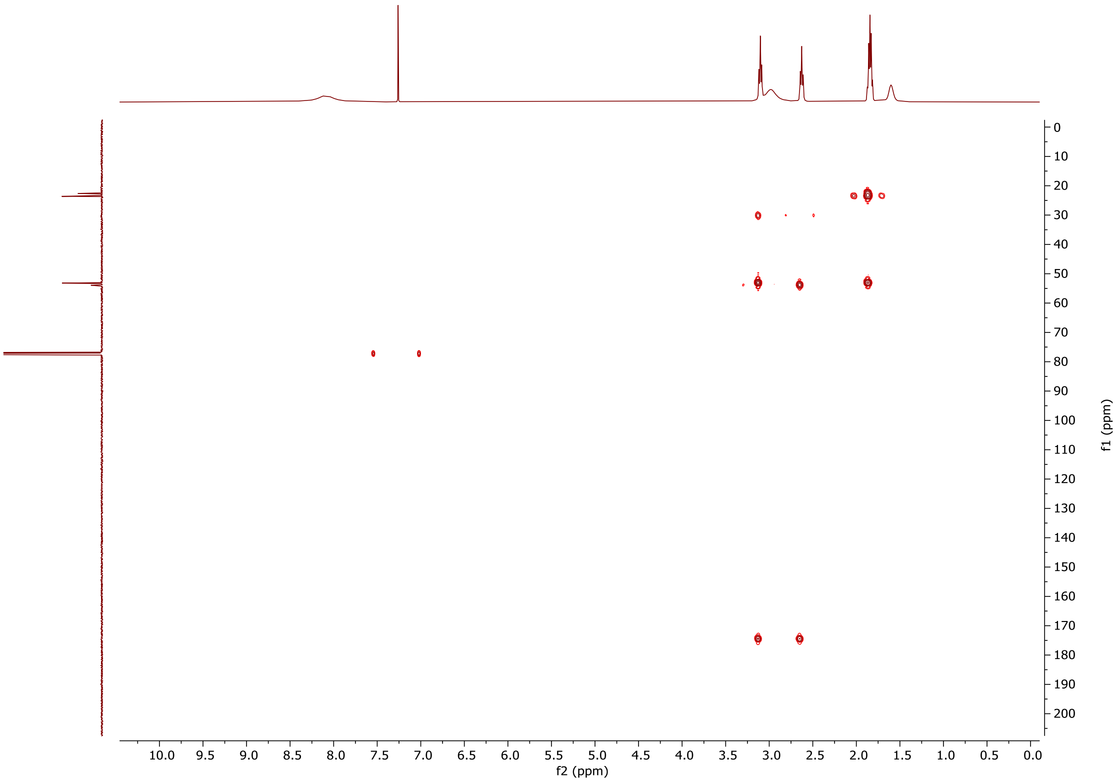
0
10
20
30
40
50
60
70
80
90
100
110
120
130
140
150
160
170
180
190
200
f1 (ppm)
10.0
9.5
9.0
8.5
8.0
7.5
7.0
6.5
6.0
5.5
5.0
4.5
4.0
3.5
3.0
2.5
2.0
1.5
1.0
0.5
0.0
f2 (ppm)

e)

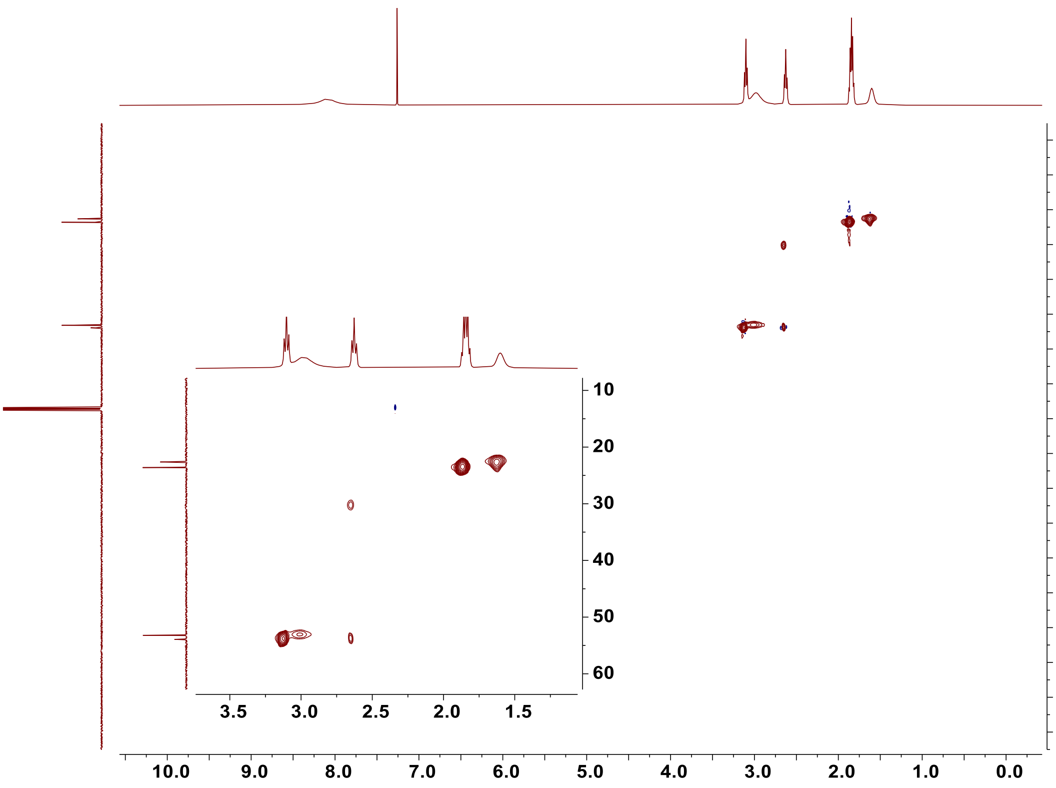

Figure S21. NMR spectra ($CDCl_3$) of **7g**: a) ^{1}H, b) ^{13}C, c) COSY, d) HMBC and e) HSQC.

a)

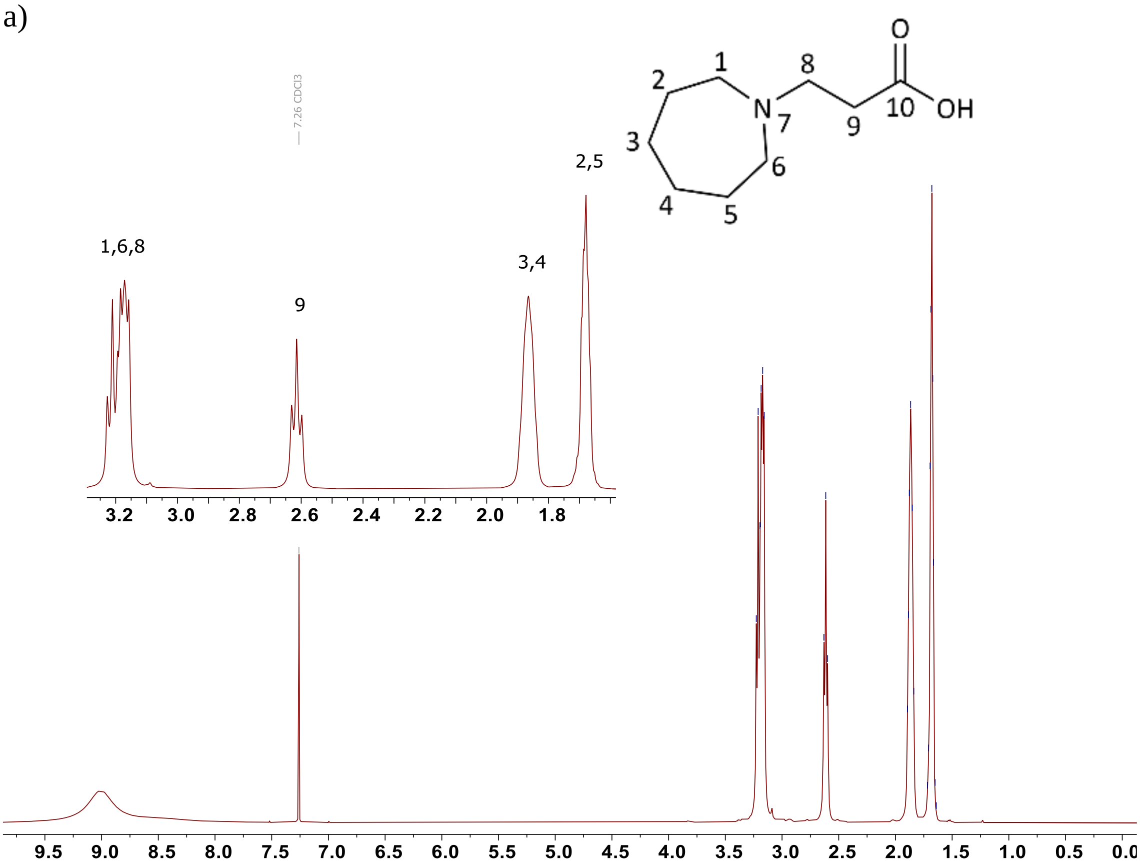

b)

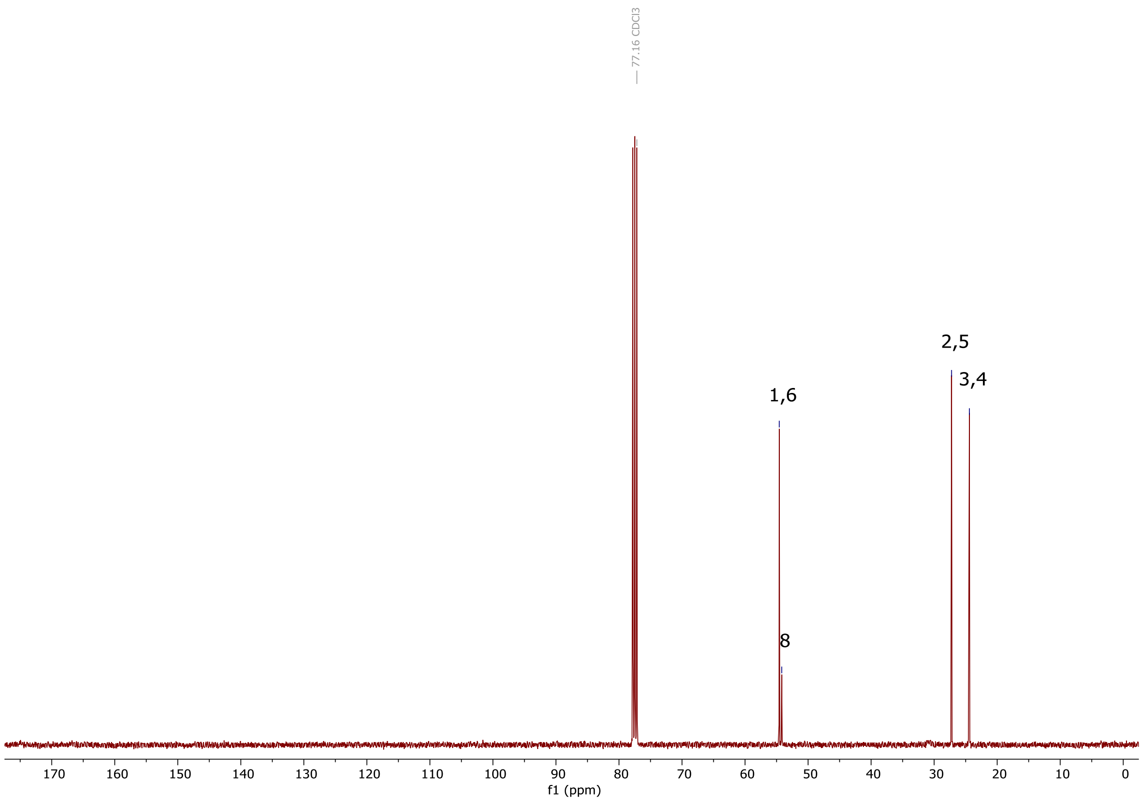

— 77.16 CDCl3
1,6
8
2,5
3,4
170 160 150 140 130 120 110 100 90 80 70 60 50 40 30 20 10 0
f1 (ppm)

c)

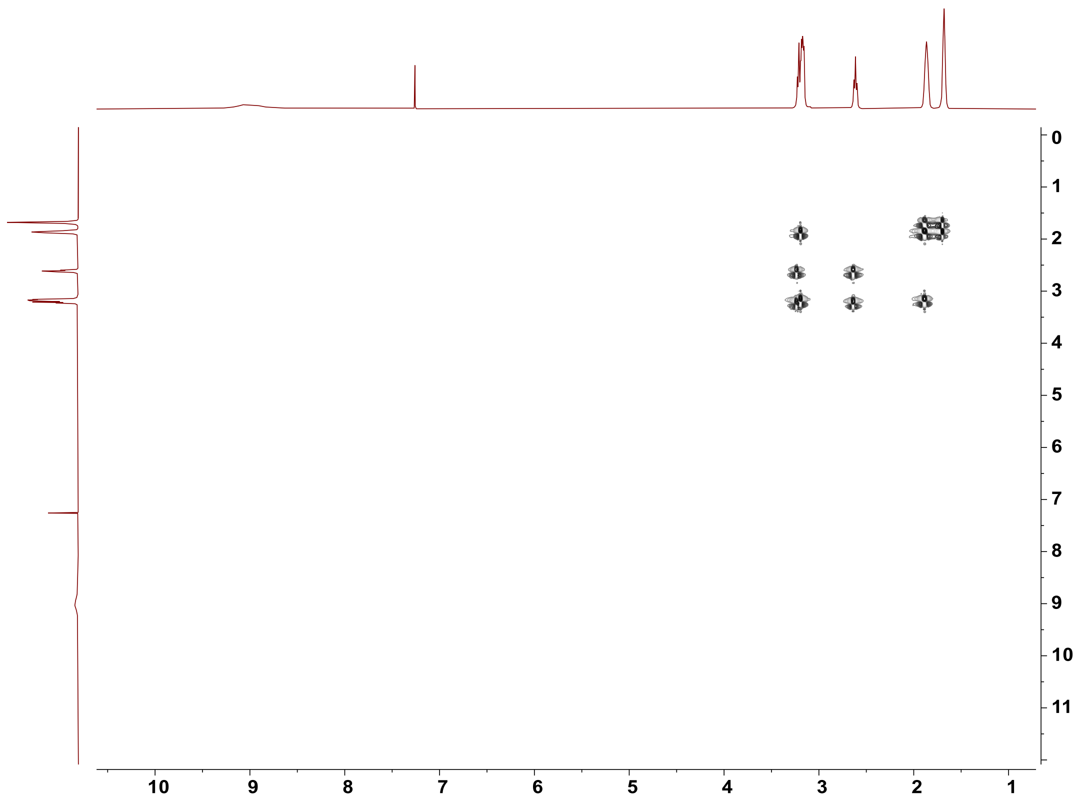

10 9 8 7 6 5 4 3 2 1
0 1 2 3 4 5 6 7 8 9 10 11

d)

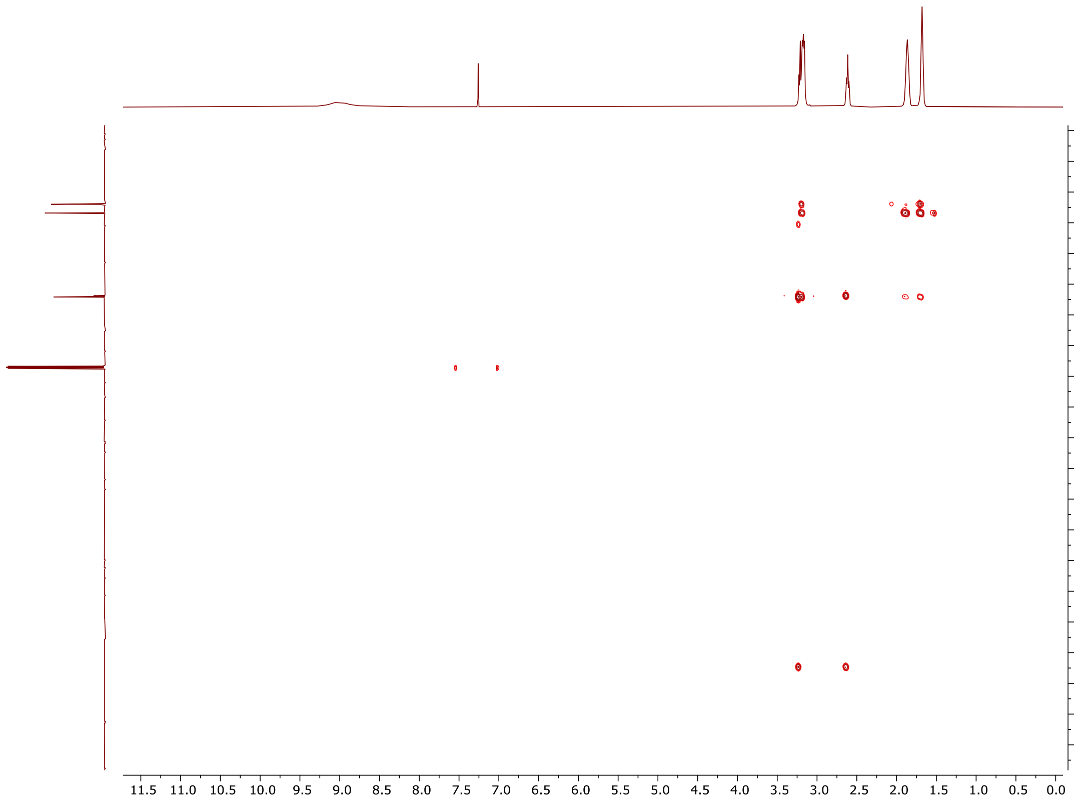

e)

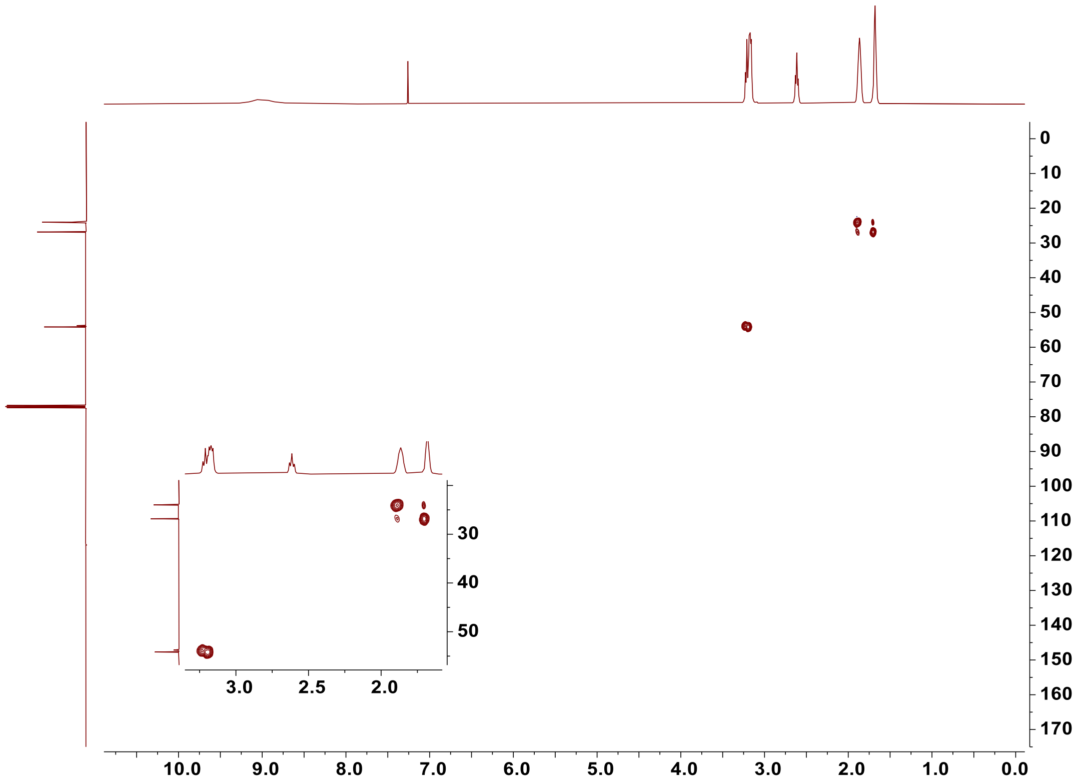

Figure S22. NMR spectra ($CDCl_3$) of **7i**: a) ^{1}H, b) ^{13}C, c) COSY, d) HMBC and e) HSQC.

a)

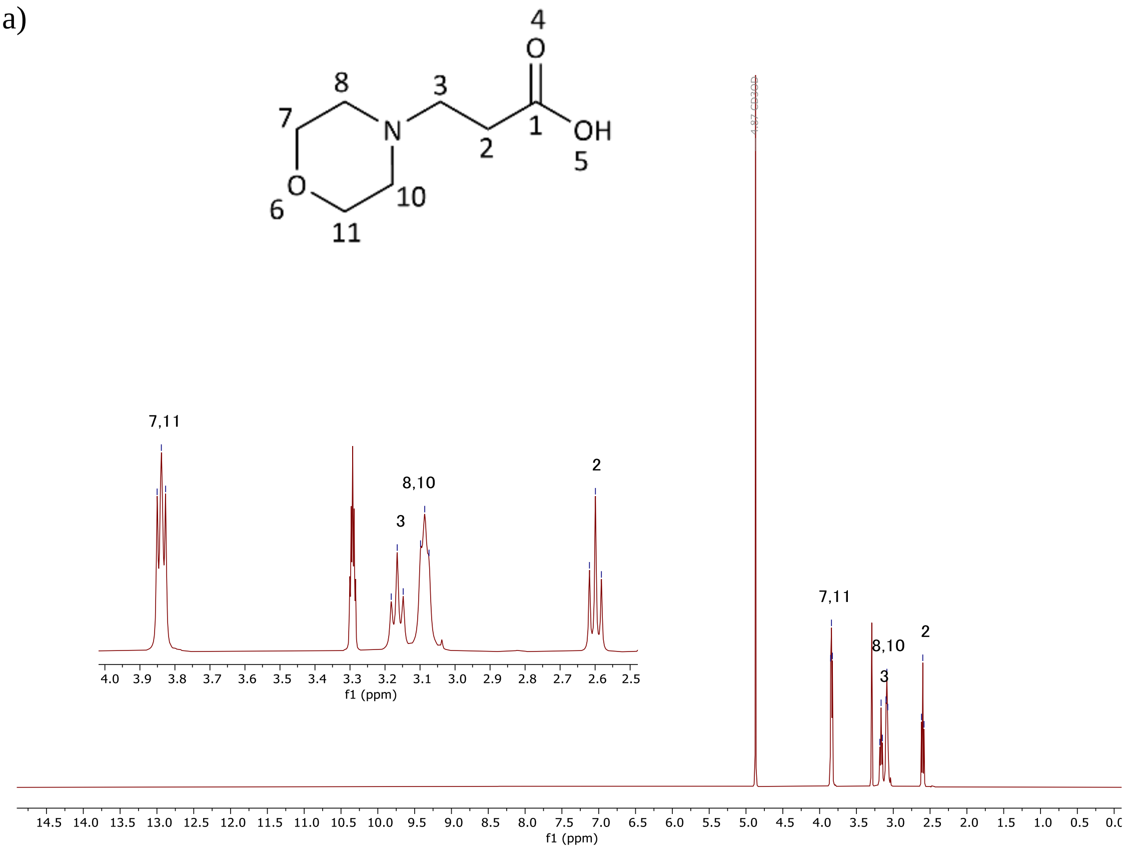

b)

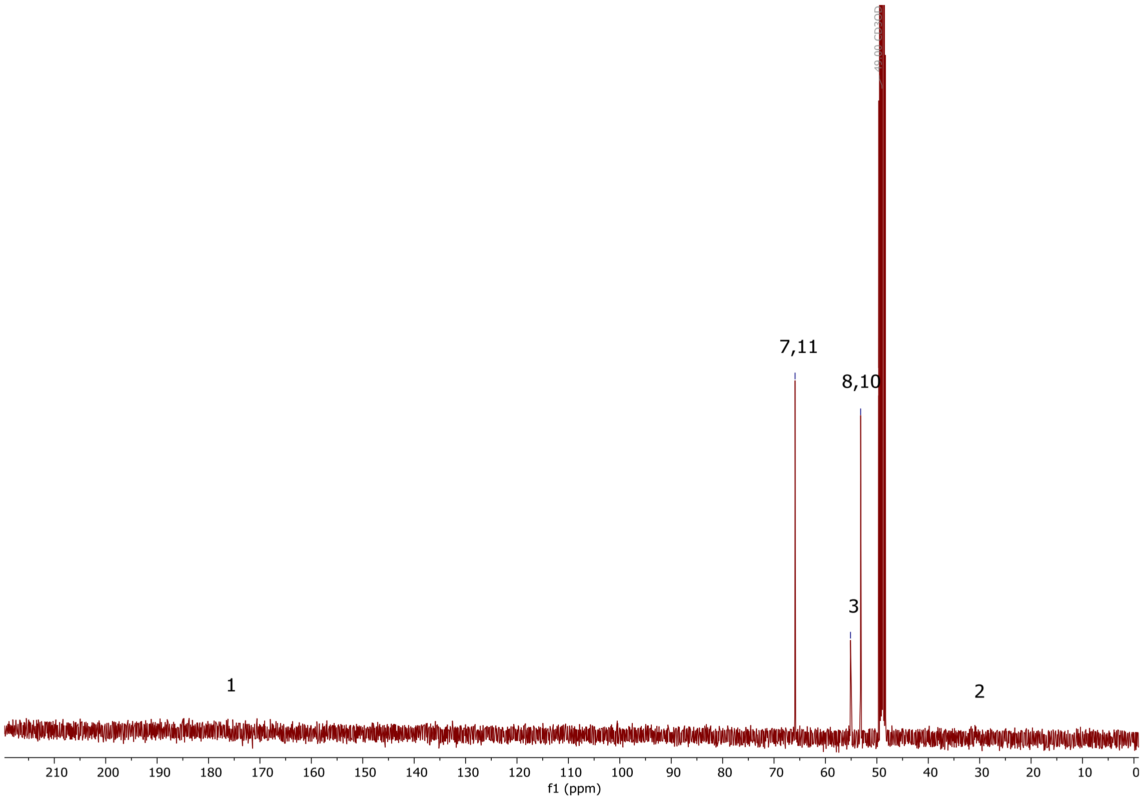

c)

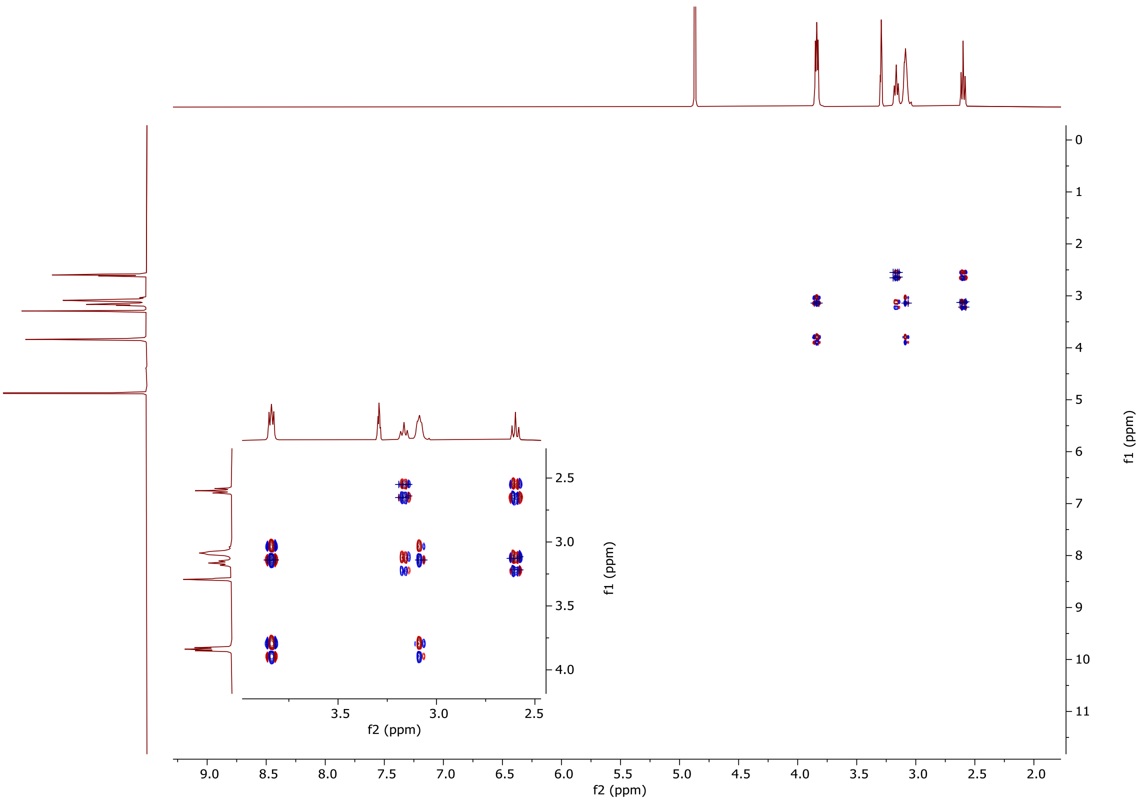

d)

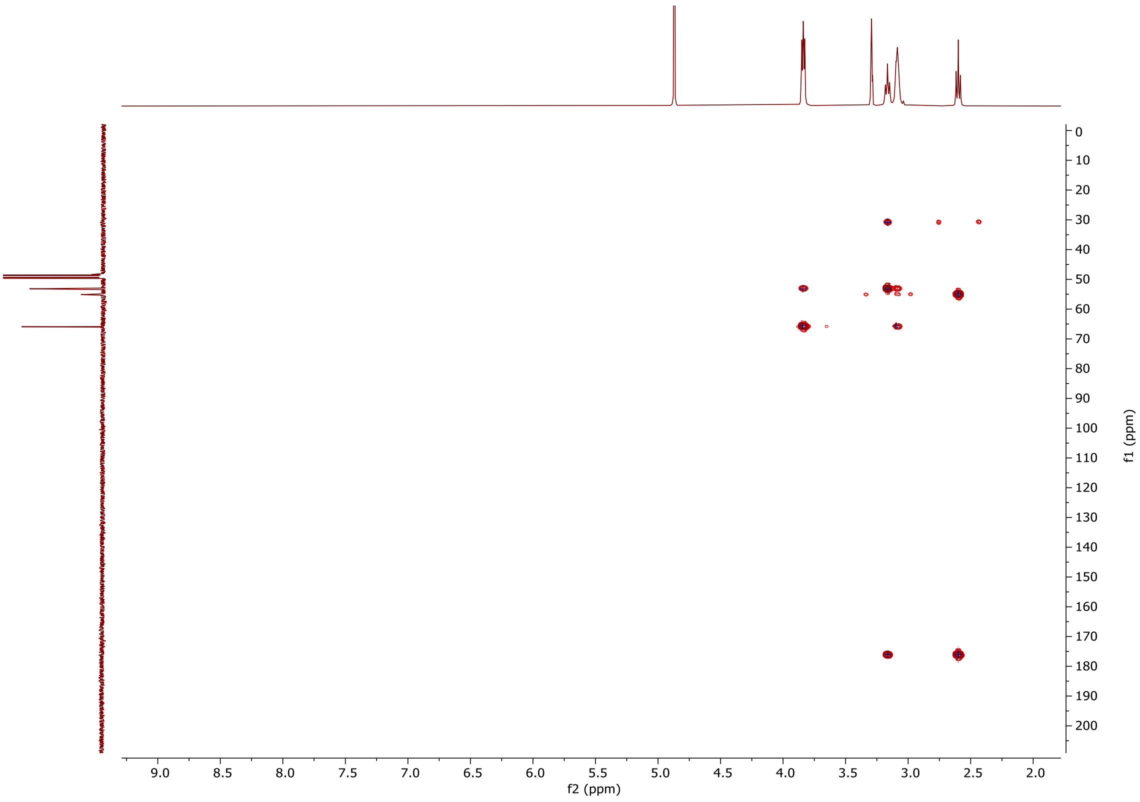

e)

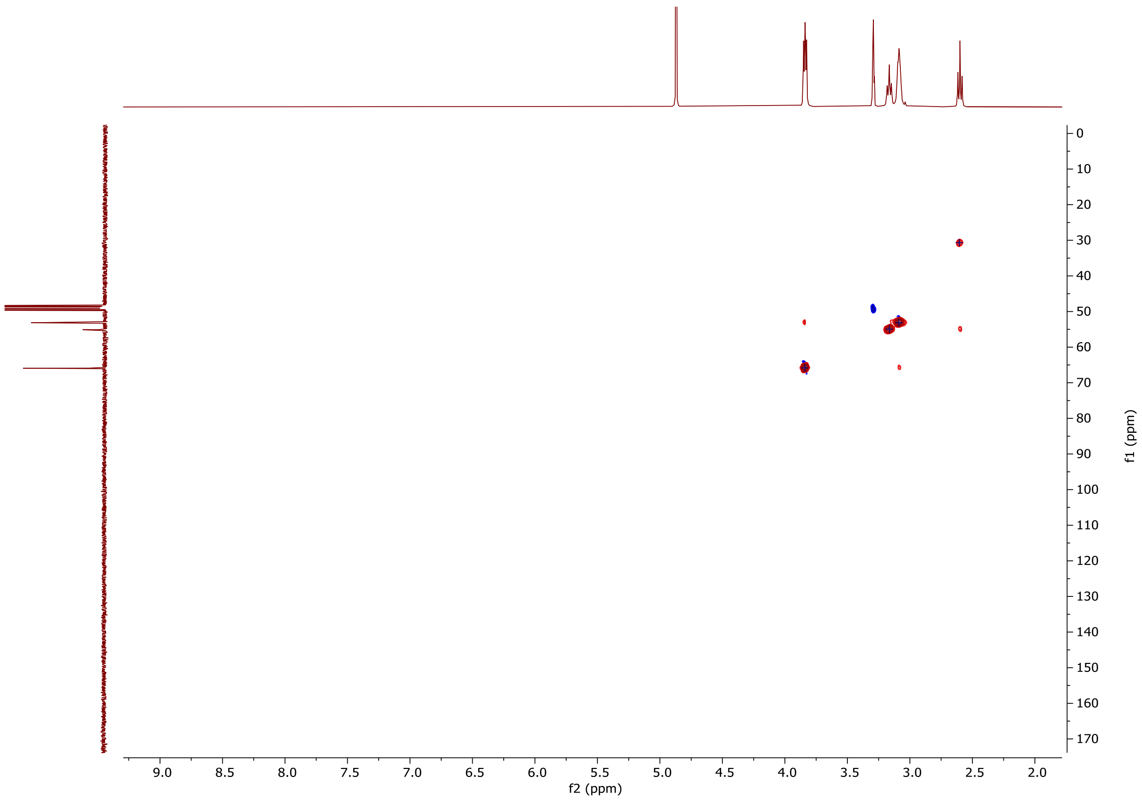

Figure S23. NMR spectra (CD_3OD) of**7j**: a) ^{1}H, b) ^{13}C, c) COSY, d) HMBC and e) HSQC.

a)

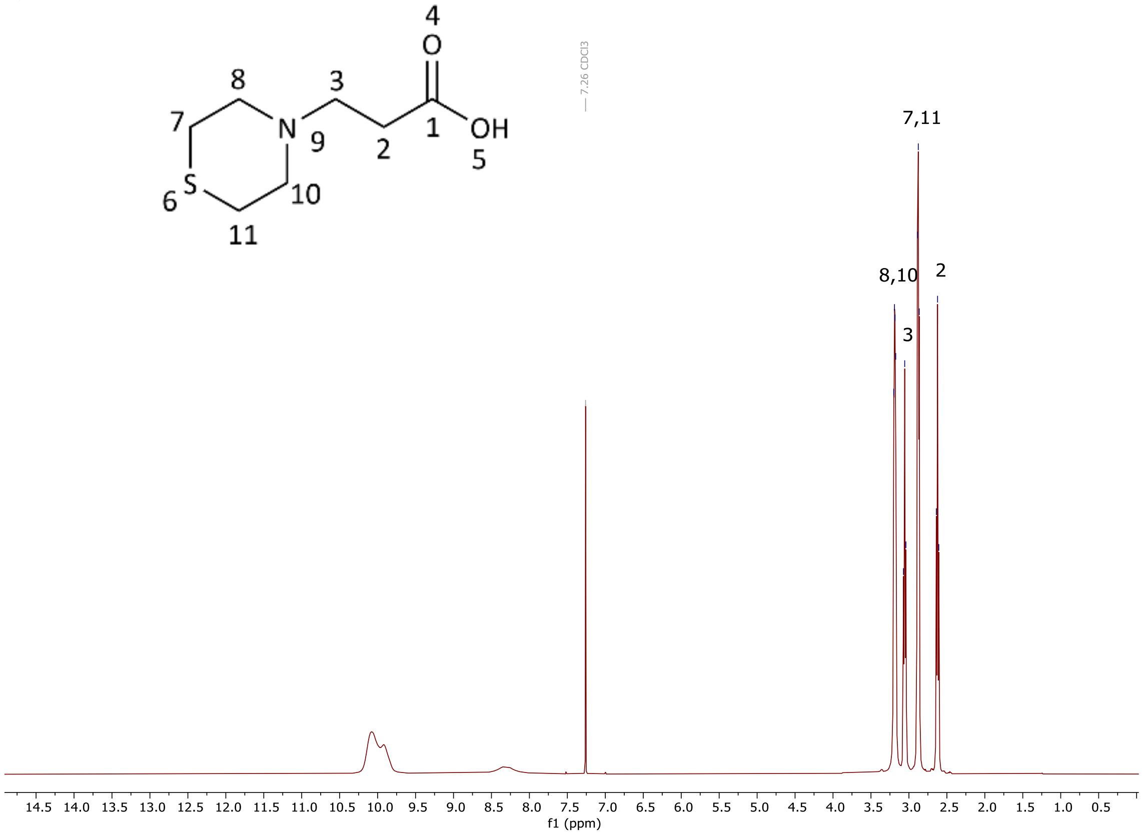

b)

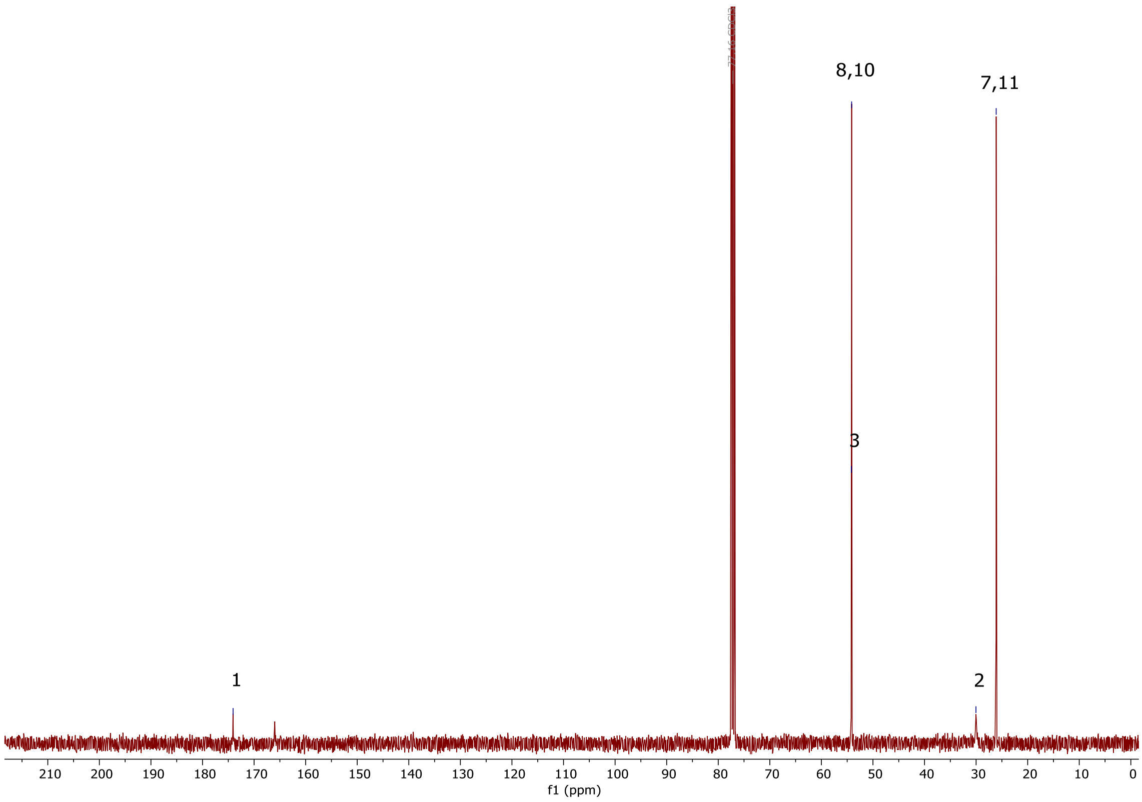
8,10
7,11
3
1
2
210
200
190
180
170
160
150
140
130
120
110
100
90
80
70
60
50
40
30
20
10
0
f1 (ppm)

c)

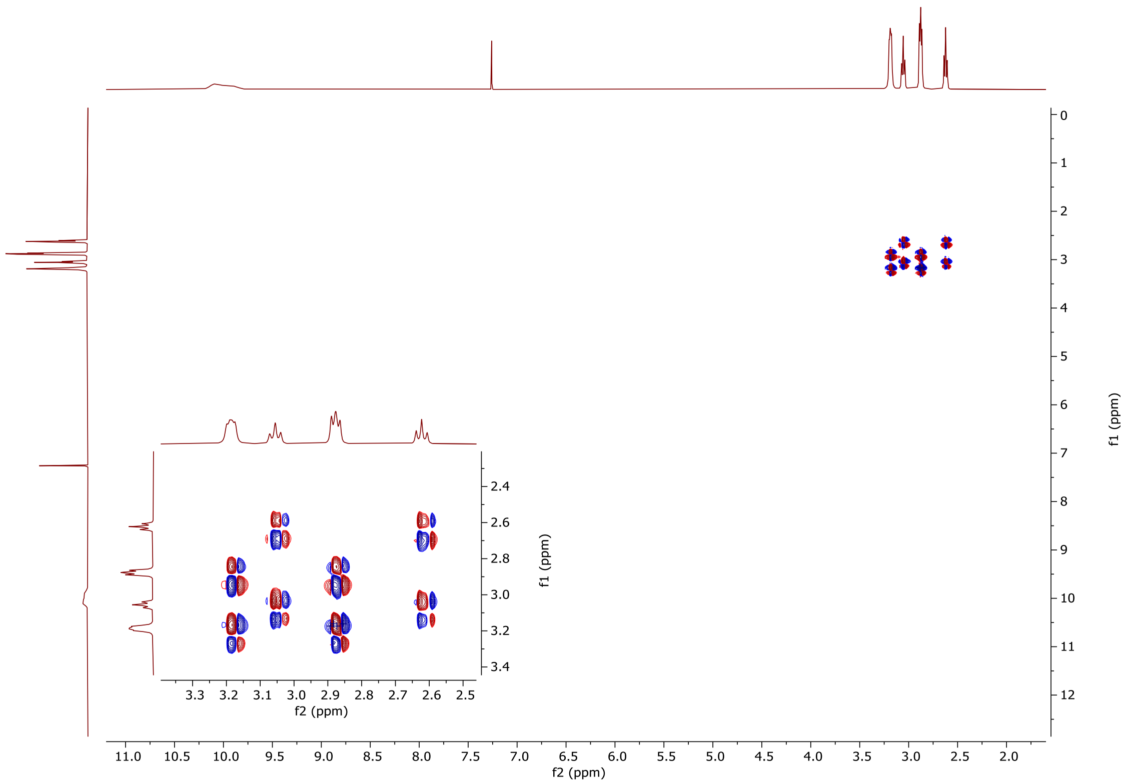
f1 (ppm)
f2 (ppm)
11.0
10.5
10.0
9.5
9.0
8.5
8.0
7.5
7.0
6.5
6.0
5.5
5.0
4.5
4.0
3.5
3.0
2.5
2.0

d)

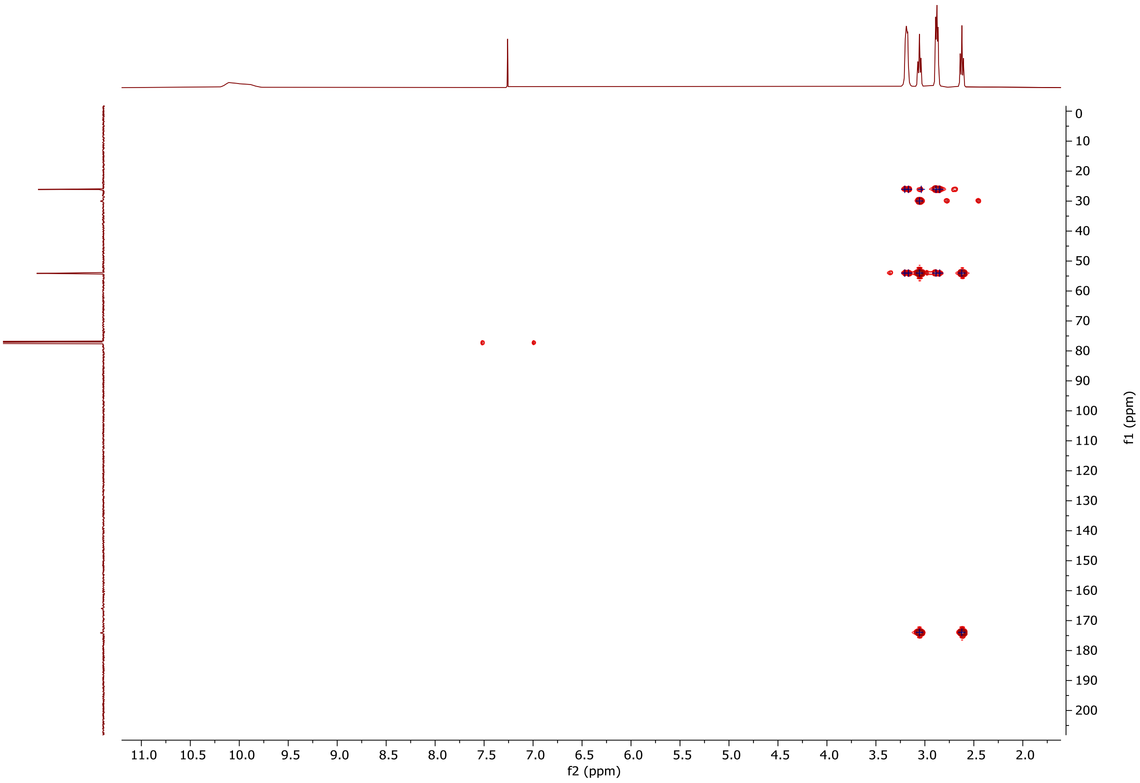

e)

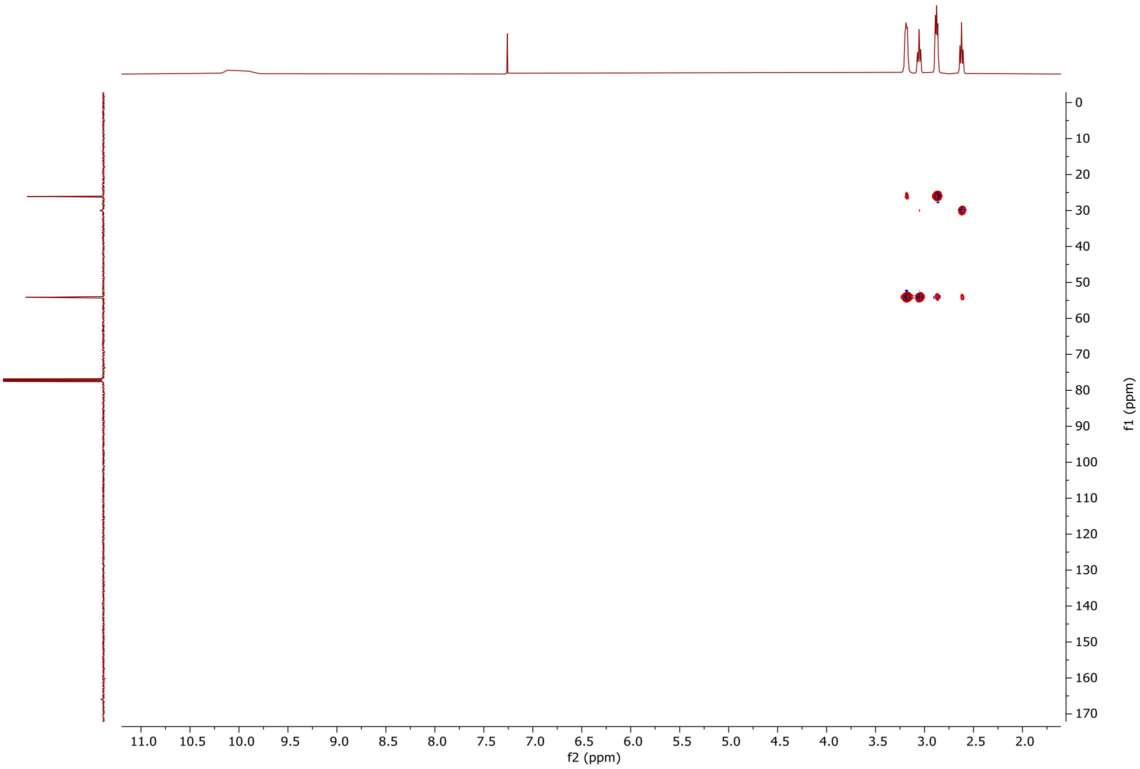

Figure S13. NMR spectra ($CDCl_3$) of **7k**: a) ^1H, b) ^{13}C, c) COSY, d) HMBC and e) HSQC.

a)

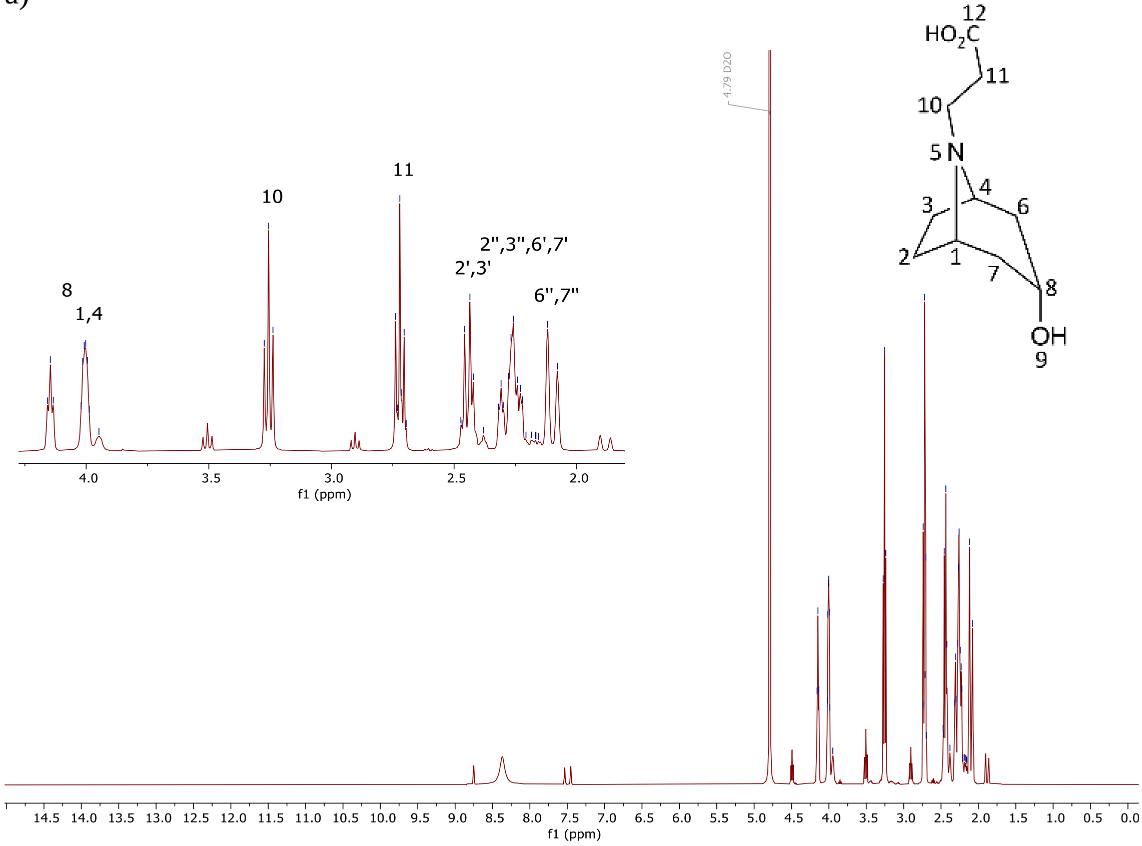

8
1,4
10
11
2",3",6',7'
2',3'
6",7"
f1 (ppm)
4.79 D2O
HO2C
12
11
10
5 N
4
3
6
2
1
7
8
OH
9

b)

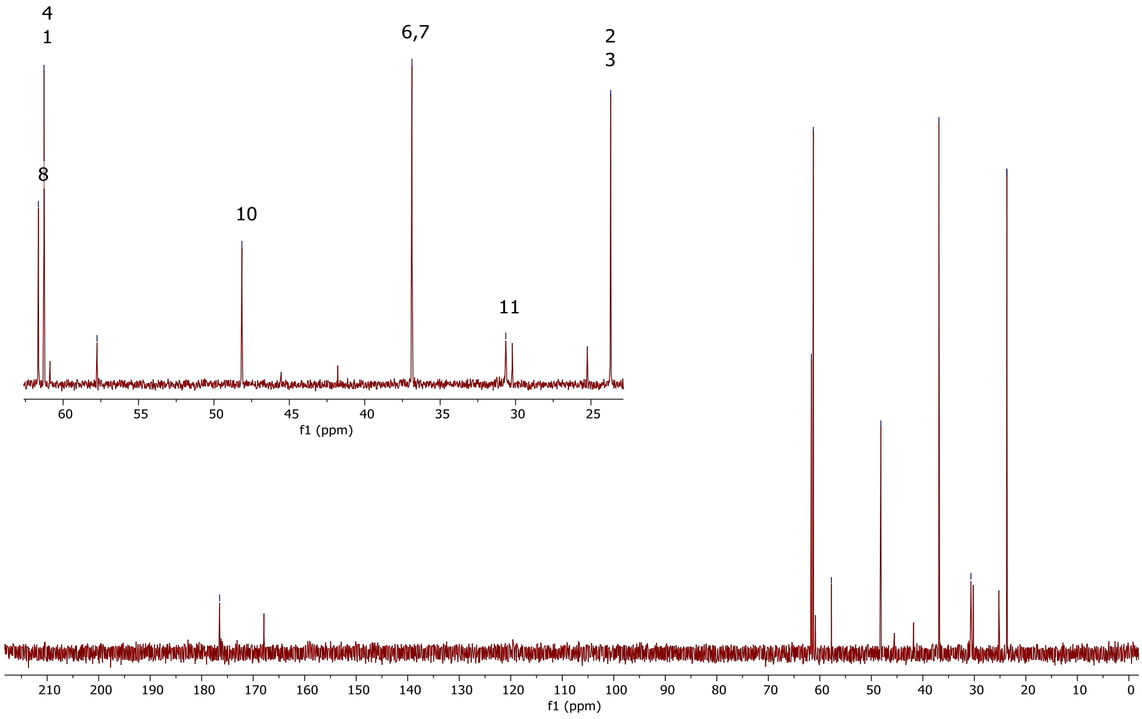

4
1
8
6,7
2
3
10
11
f1 (ppm)

c)

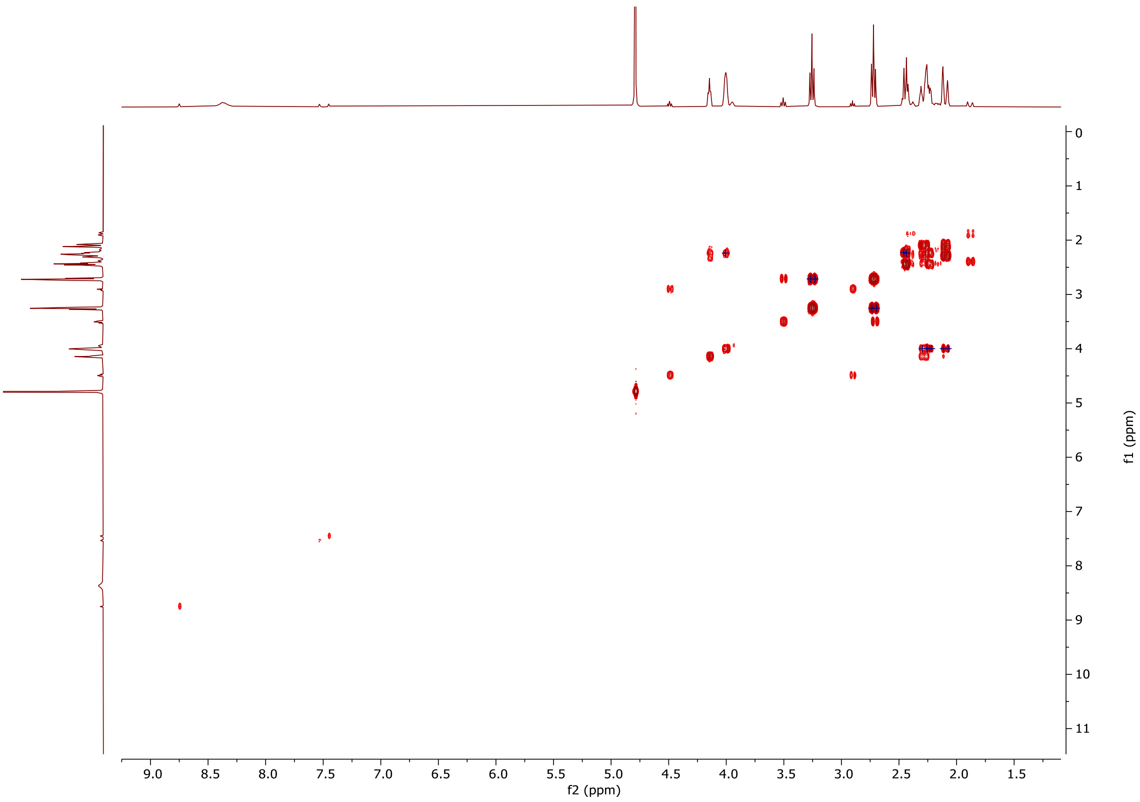

d)

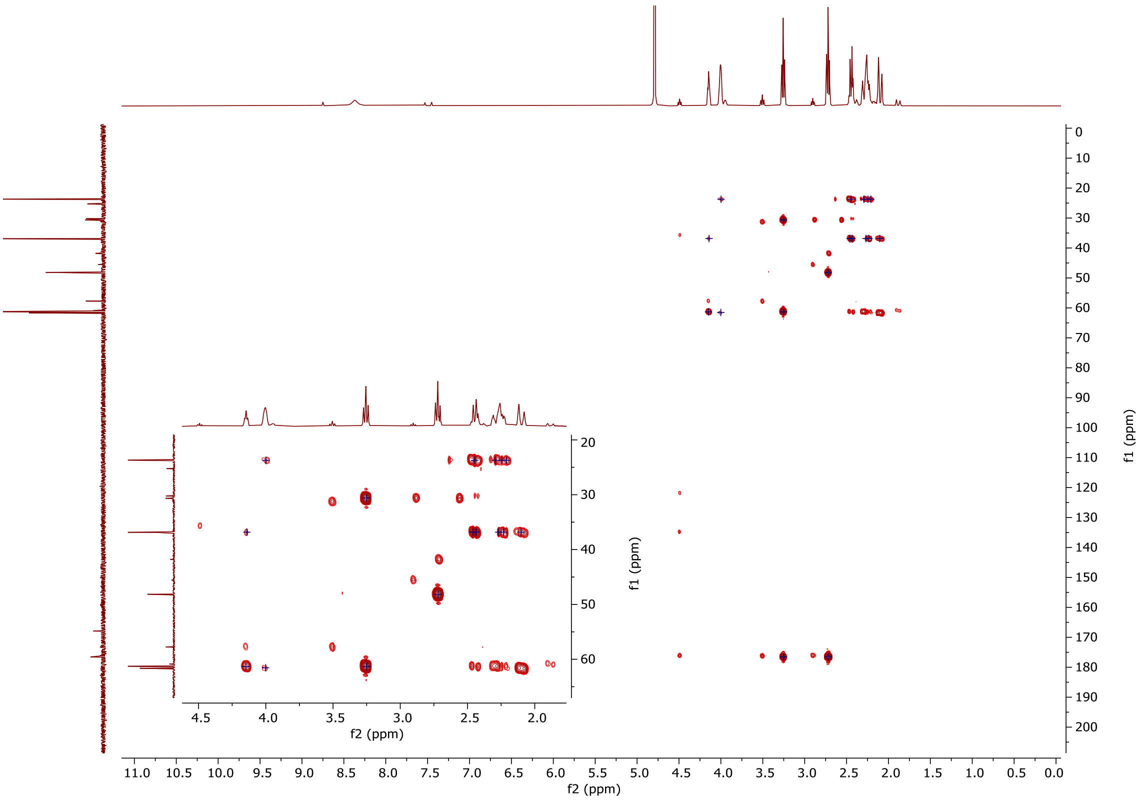

e)

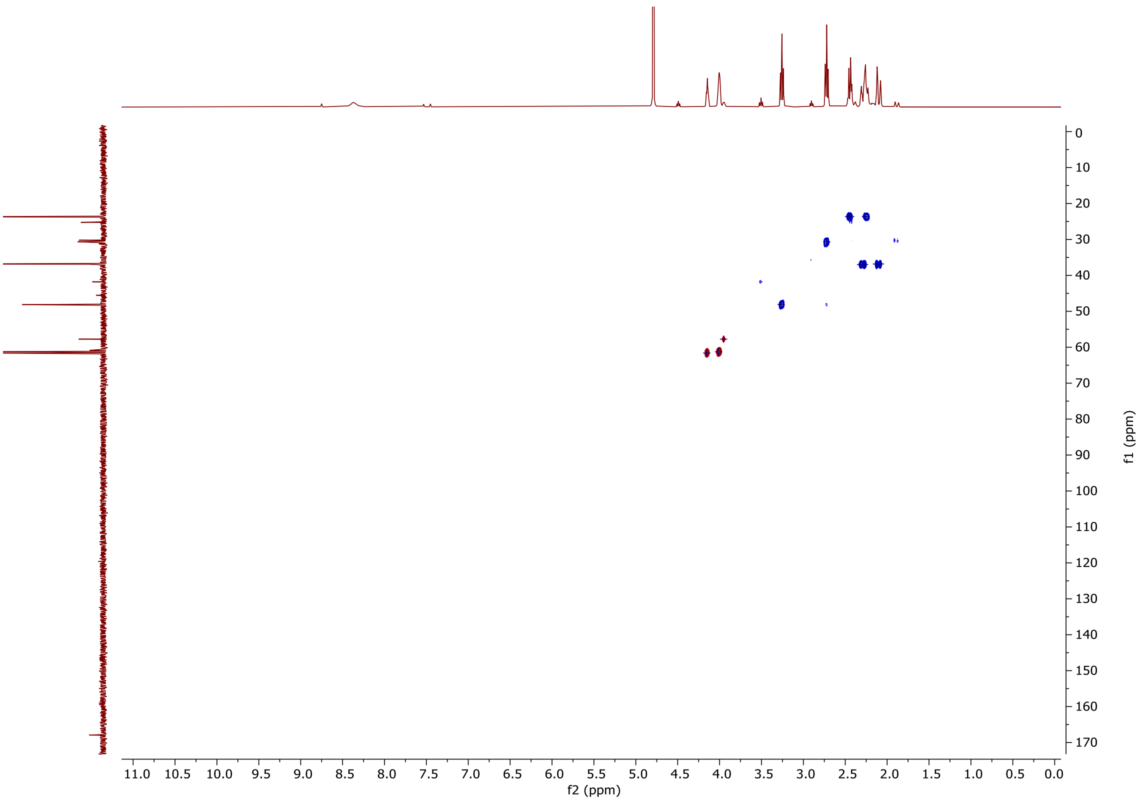

Figure S14. NMR spectra (D_2O) of **7l**: a) ^1H, b) ^{13}C, c) COSY, d) HMBC and e) HSQC.

a)

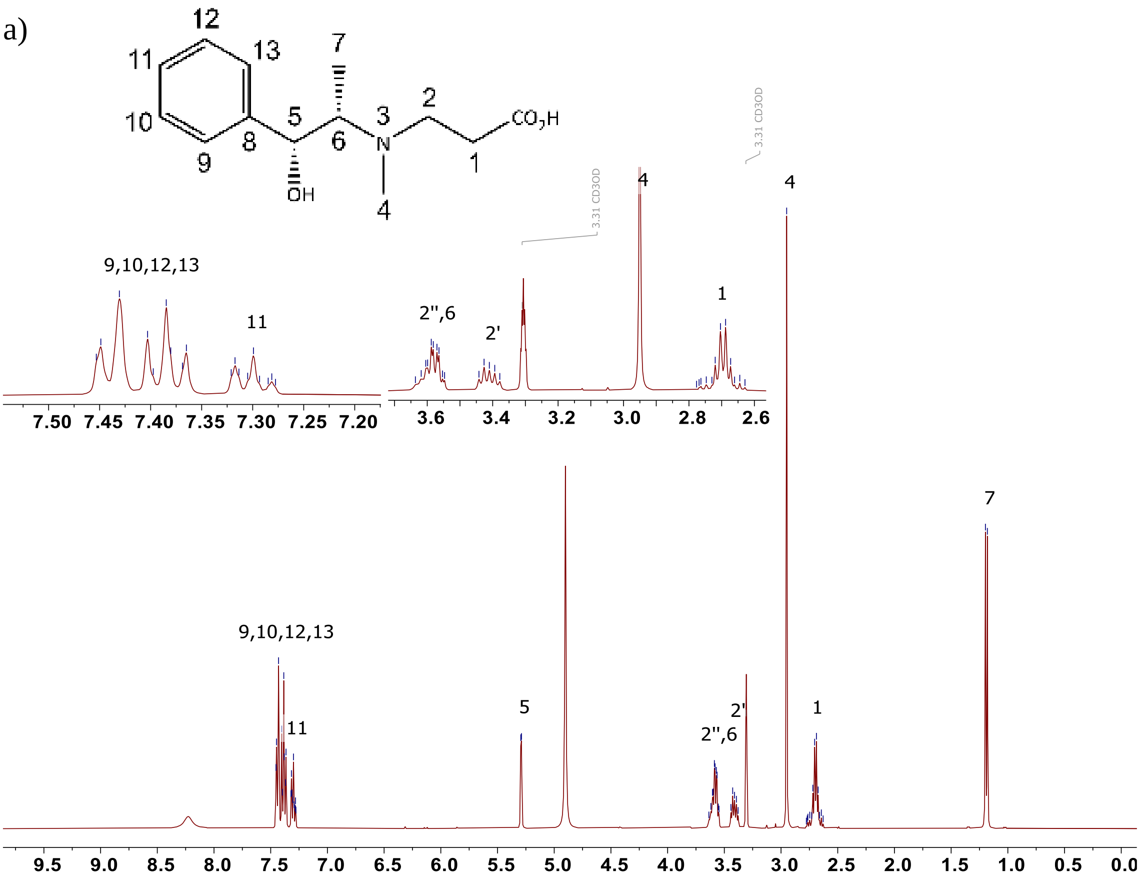

b)

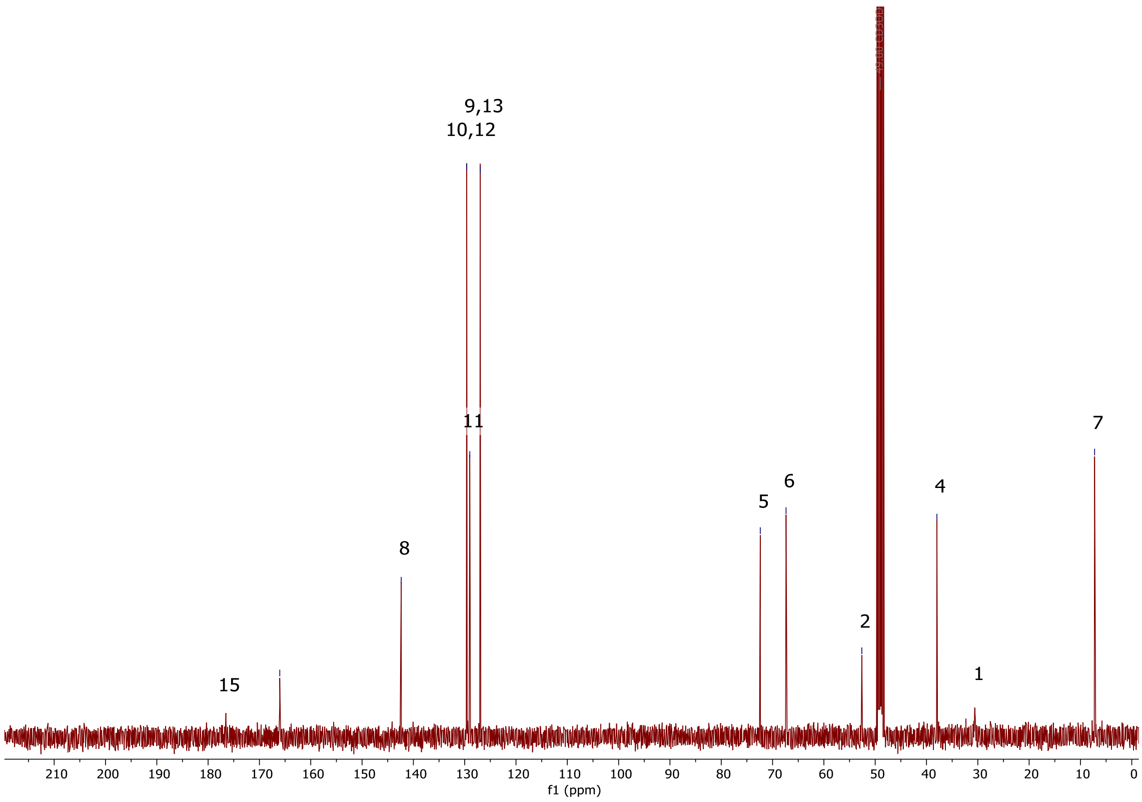
9,13
10,12
11
8
15
5
6
2
4
1
7
210 200 190 180 170 160 150 140 130 120 110 100 90 80 70 60 50 40 30 20 10 0
f1 (ppm)

c)

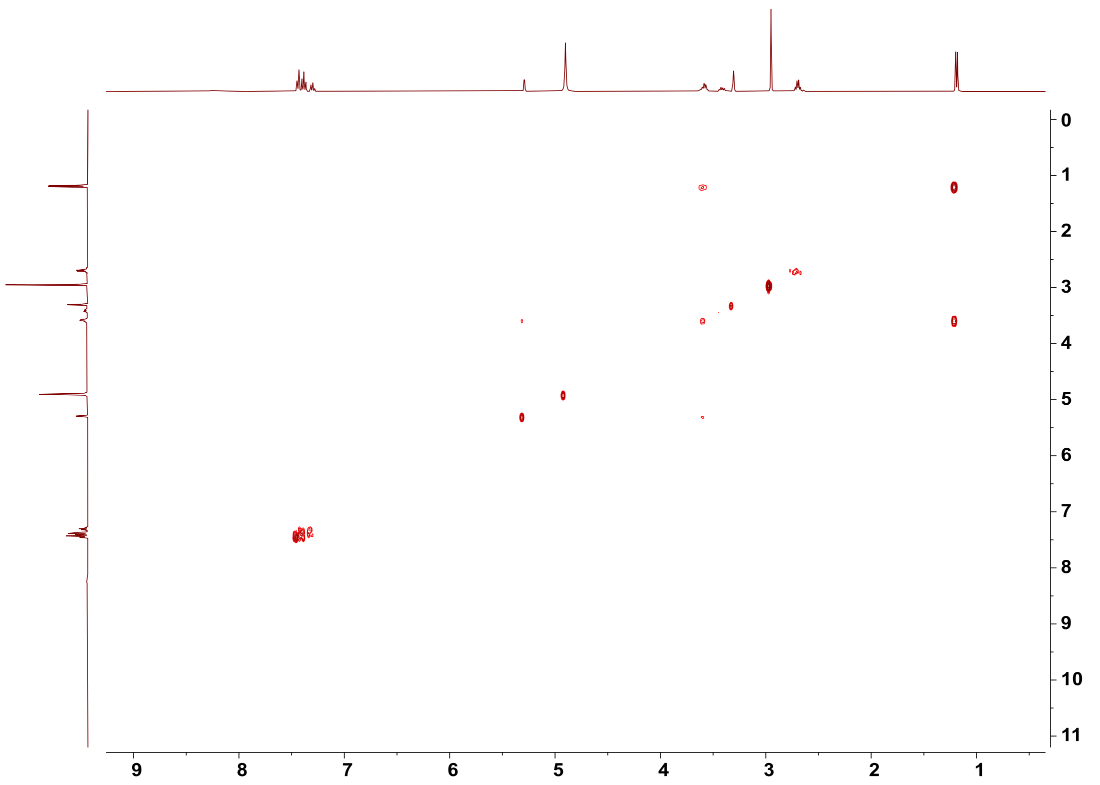
0 1 2 3 4 5 6 7 8 9 10 11
9 8 7 6 5 4 3 2 1

d)

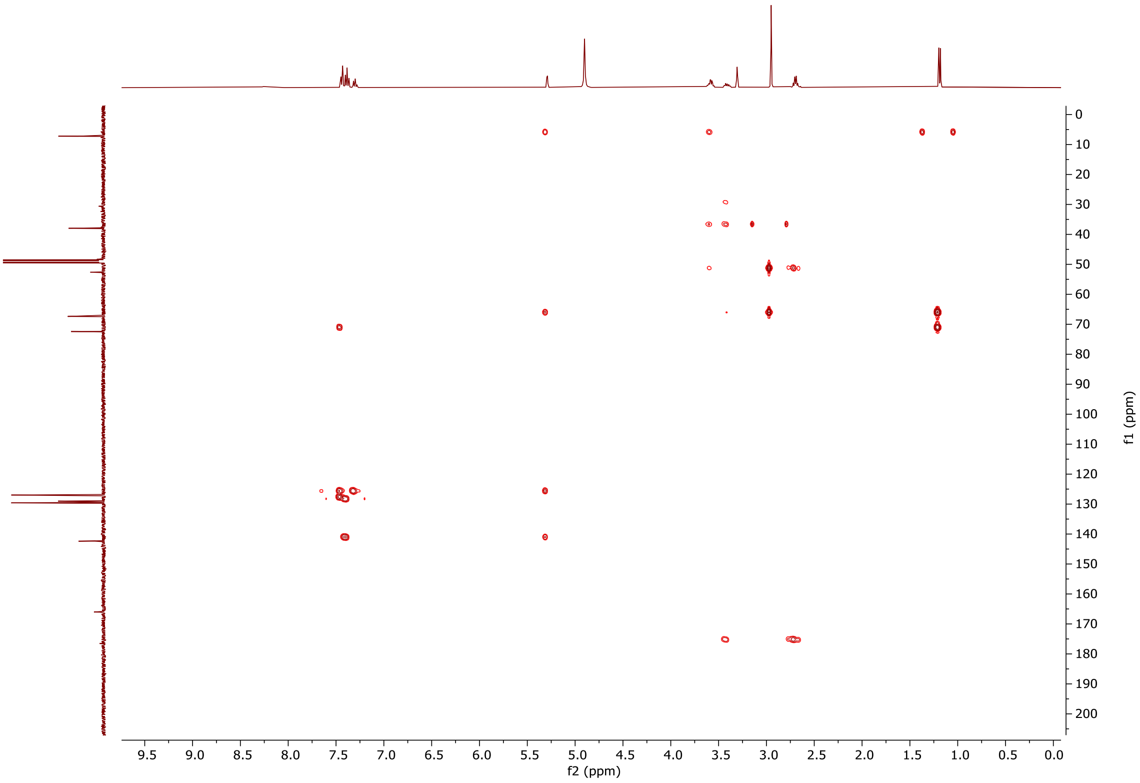

e)

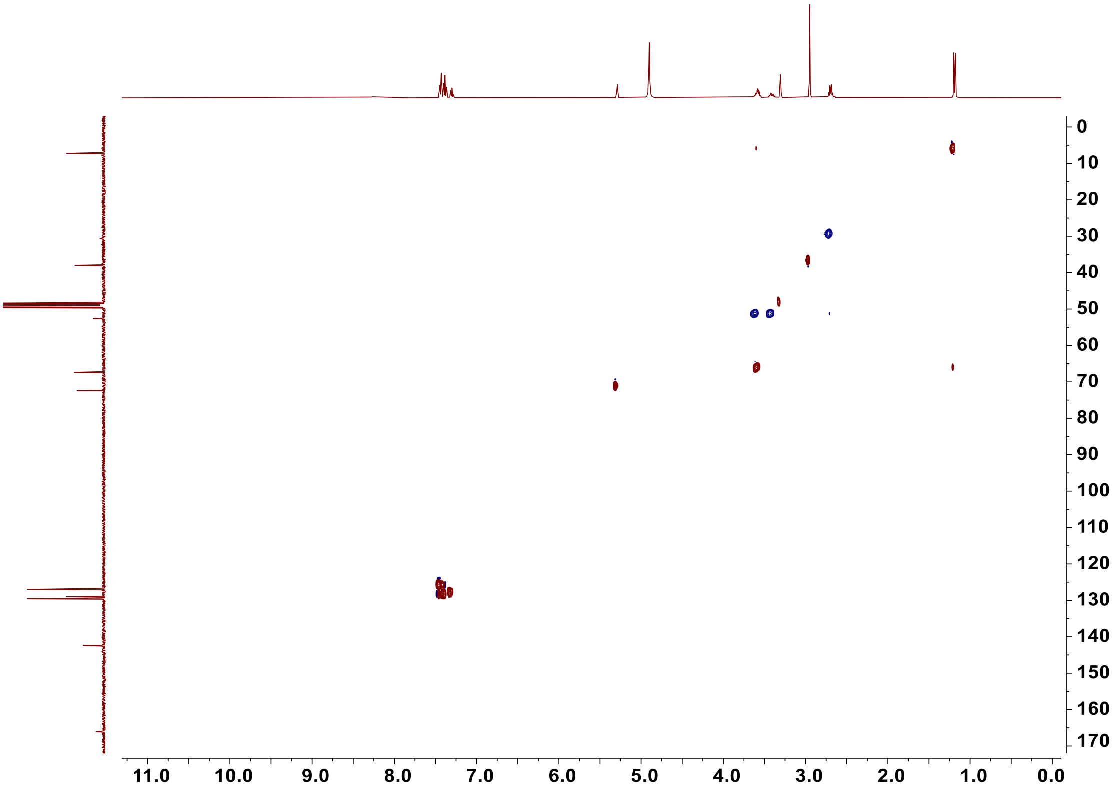

Figure S15. NMR spectra (CD_3OD) of **7m**: a) ^1H, b) ^{13}C, c) COSY, d) HMBC and e) HSQC.

a)

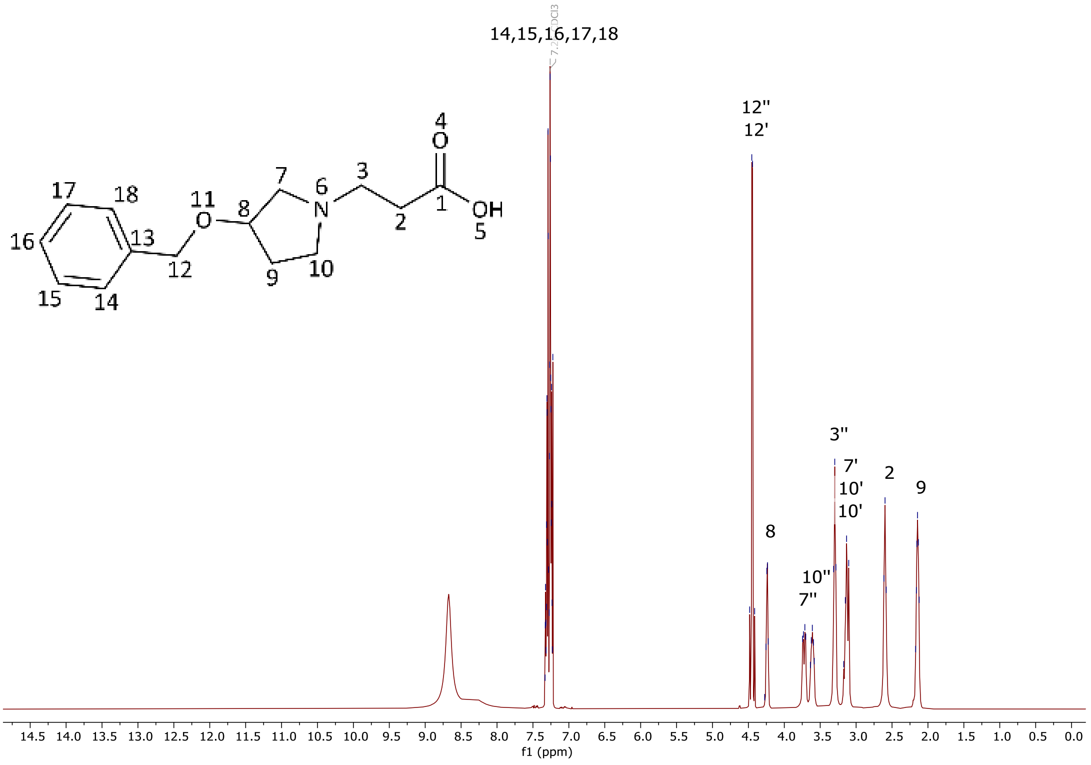
14,15,16,17,18
12''
12'
8
10''
7''
3''
7'
10'
10'
2
9
17
18
11
8
7
6
3
4
O
1
OH
5
16
13
O
N
12
9
10
2
15
14
14.5 14.0 13.5 13.0 12.5 12.0 11.5 11.0 10.5 10.0 9.5 9.0 8.5 8.0 7.5 7.0 6.5 6.0 5.5 5.0 4.5 4.0 3.5 3.0 2.5 2.0 1.5 1.0 0.5 0.0
f1 (ppm)

b)

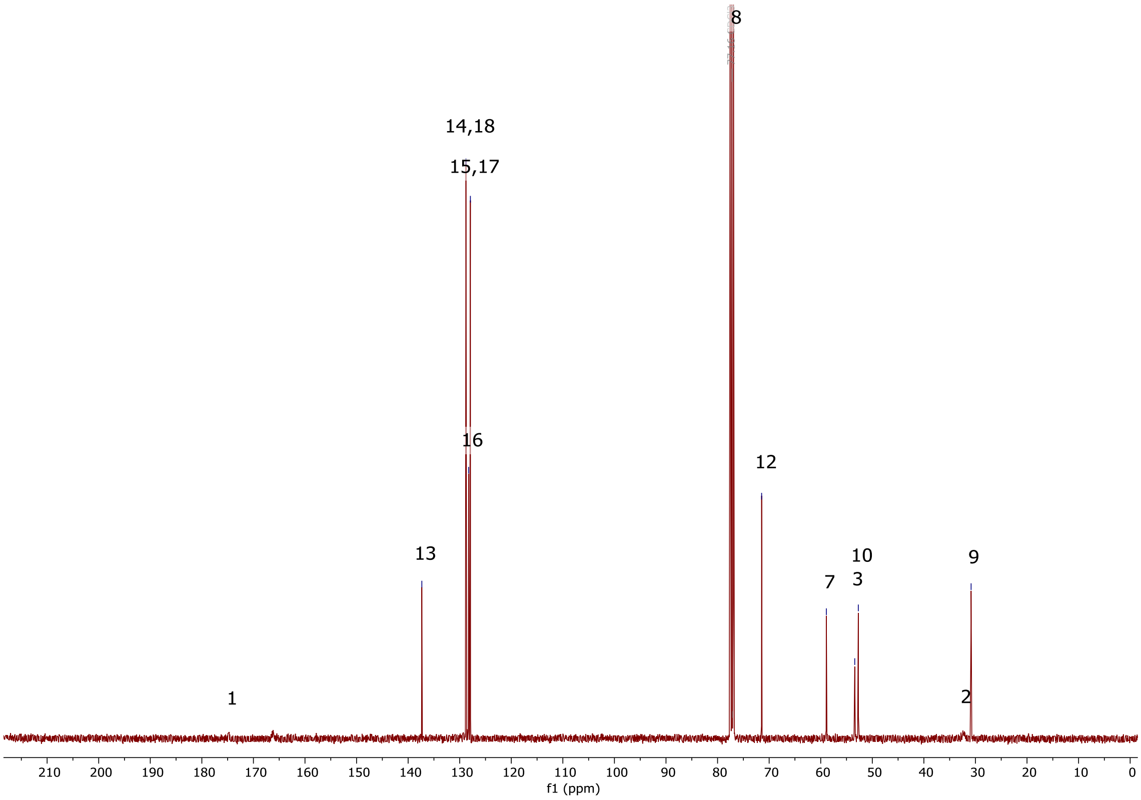
8
14,18
15,17
16
12
13
10
3
7
9
1
2
210 200 190 180 170 160 150 140 130 120 110 100 90 80 70 60 50 40 30 20 10 0
f1 (ppm)

c)

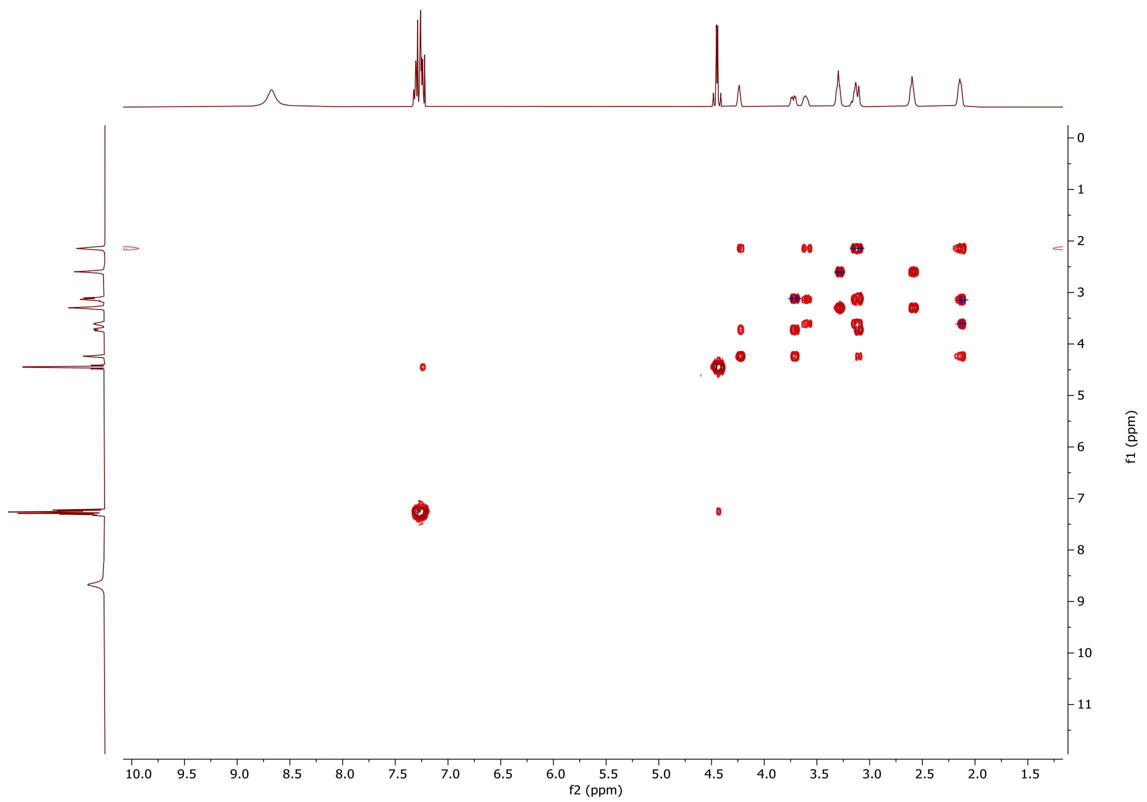

d)

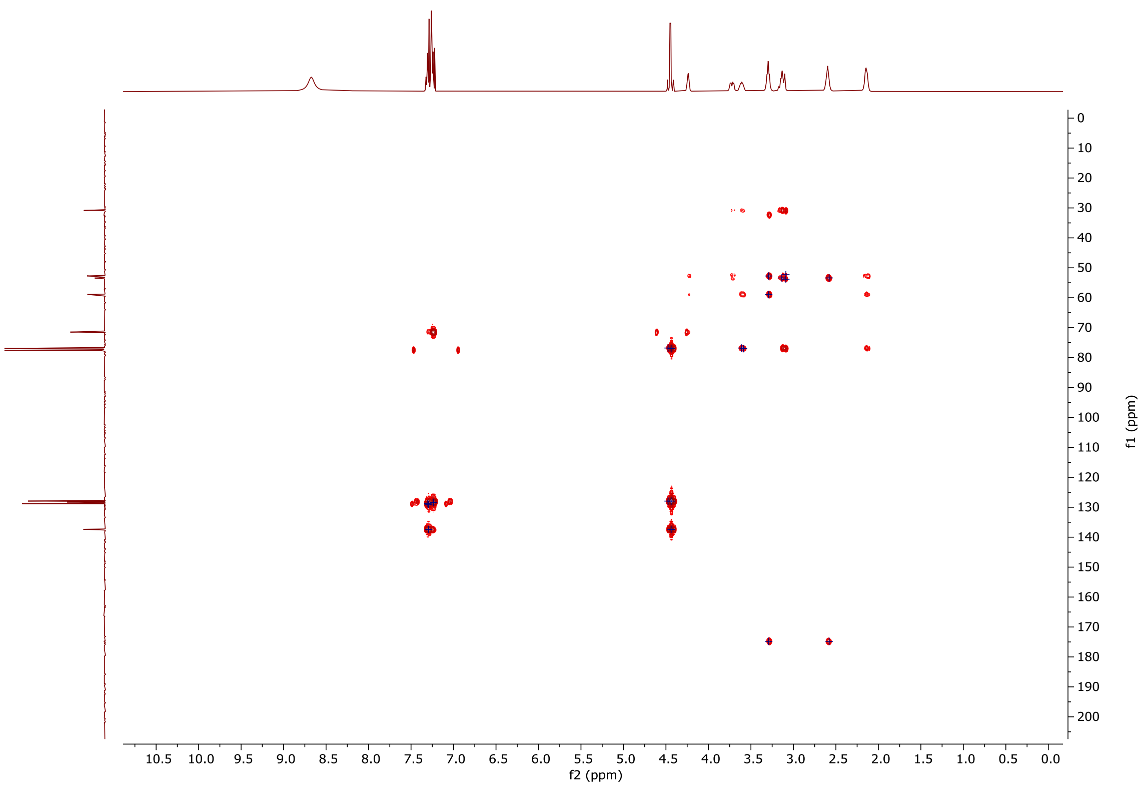

e)

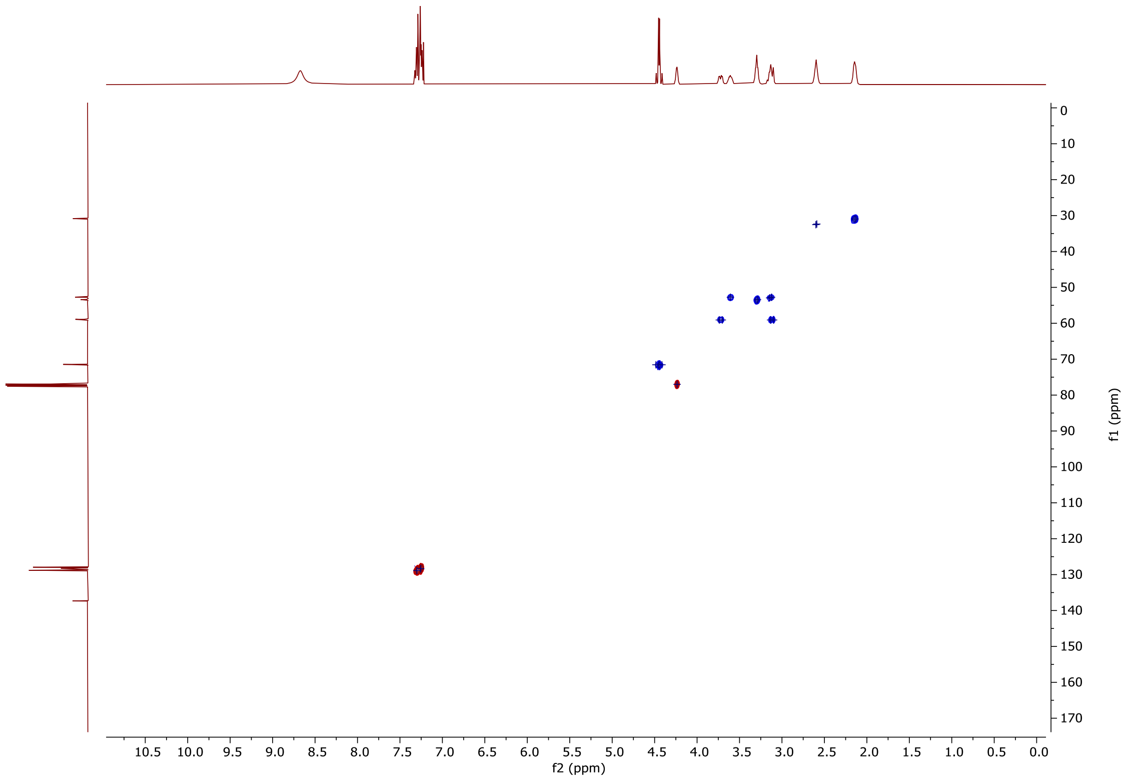

Figure S16. NMR spectra ($CDCl_3$) of **7p**: a) ^{1}H, b) ^{13}C, c) COSY, d) HMBC and e) HSQC.

a)

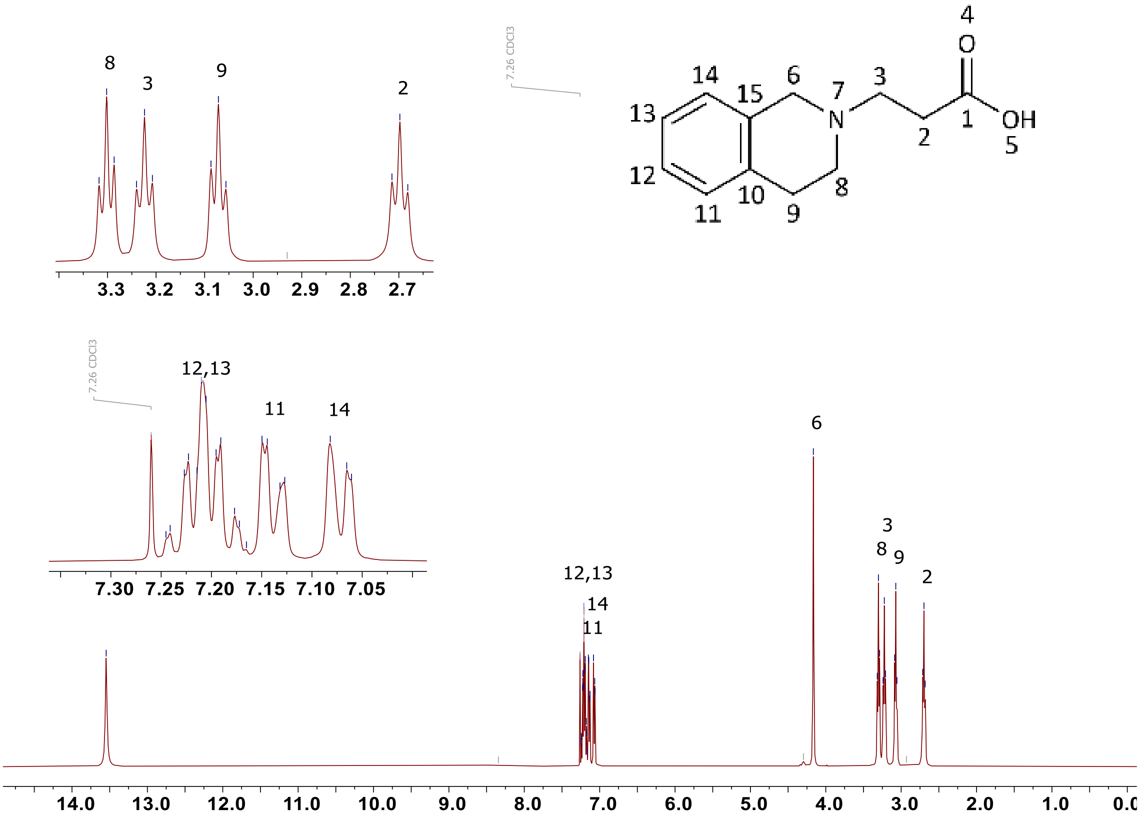

b)

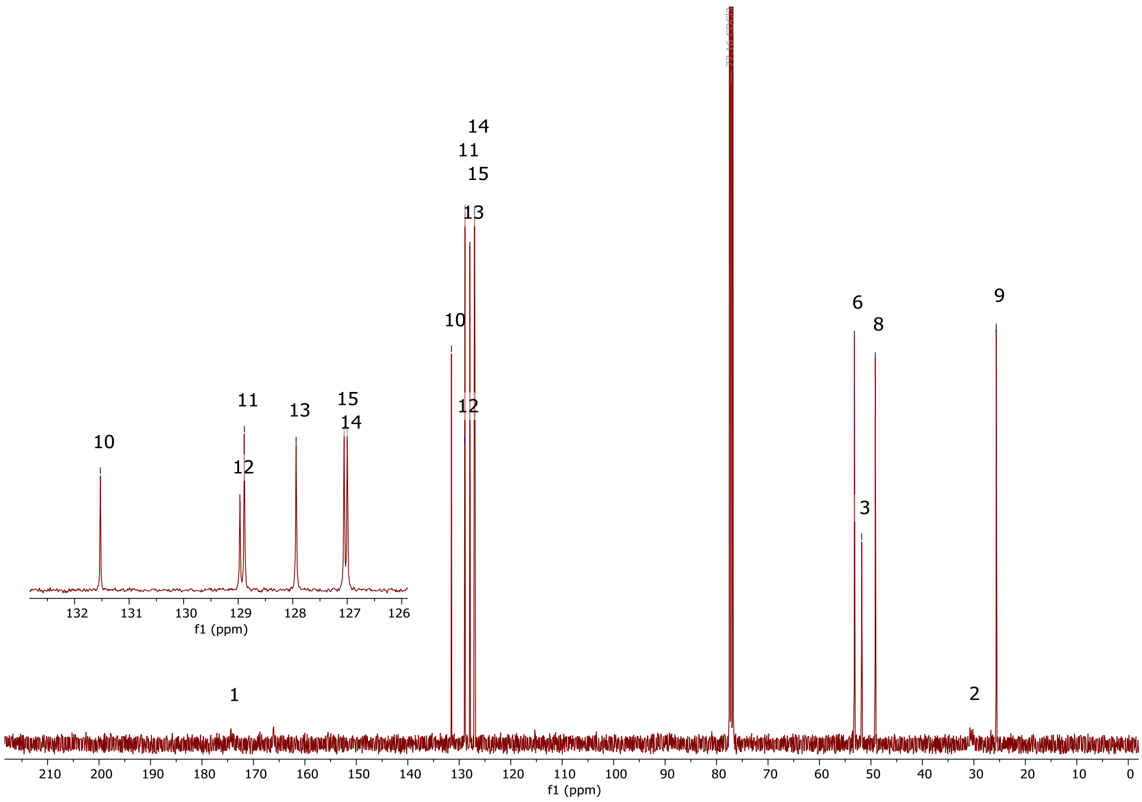

c)

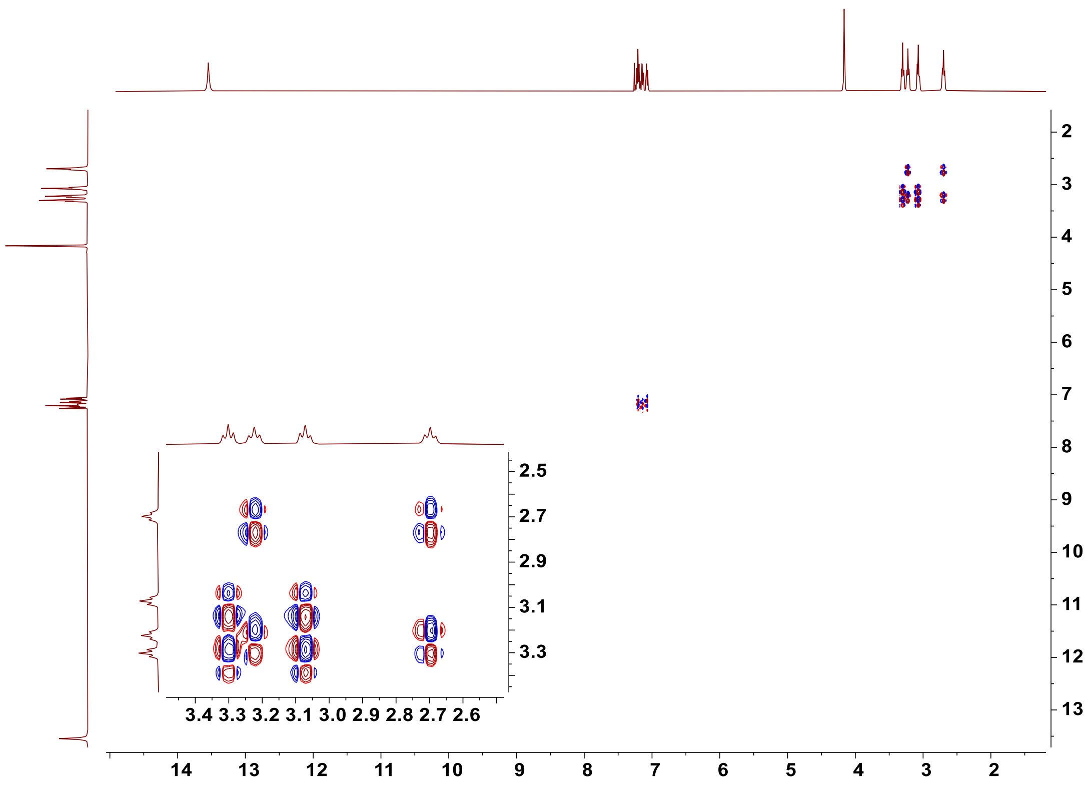

d)

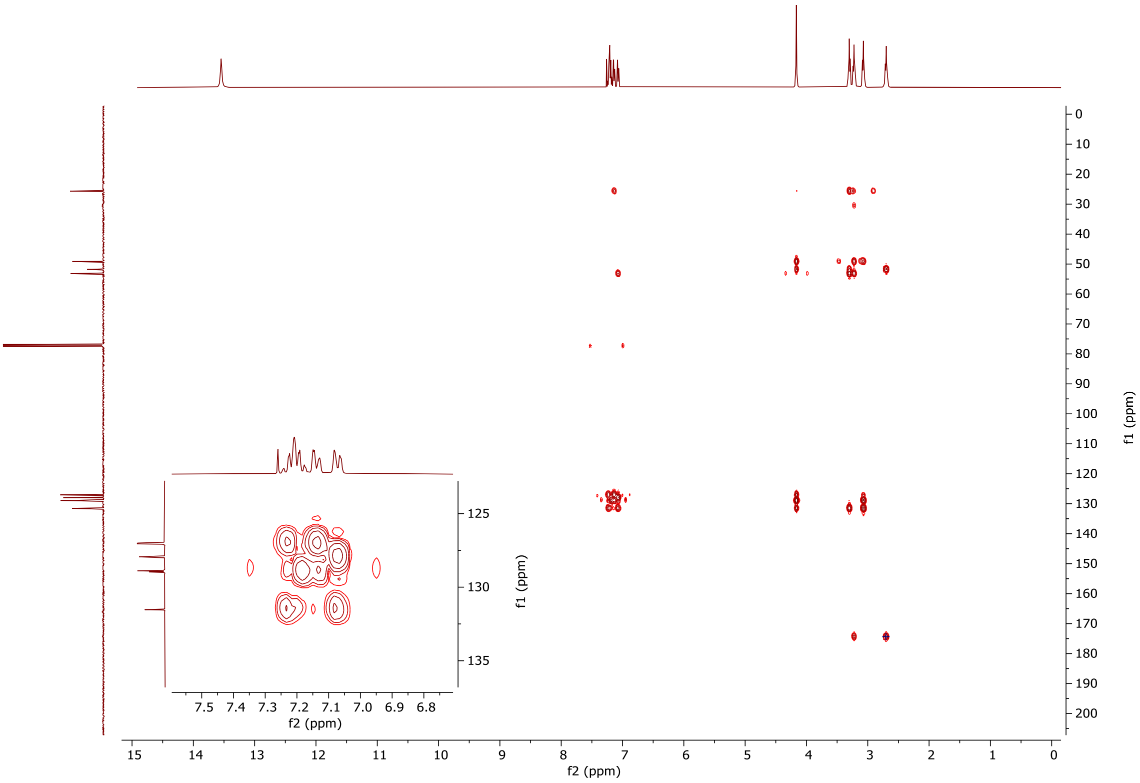

e)

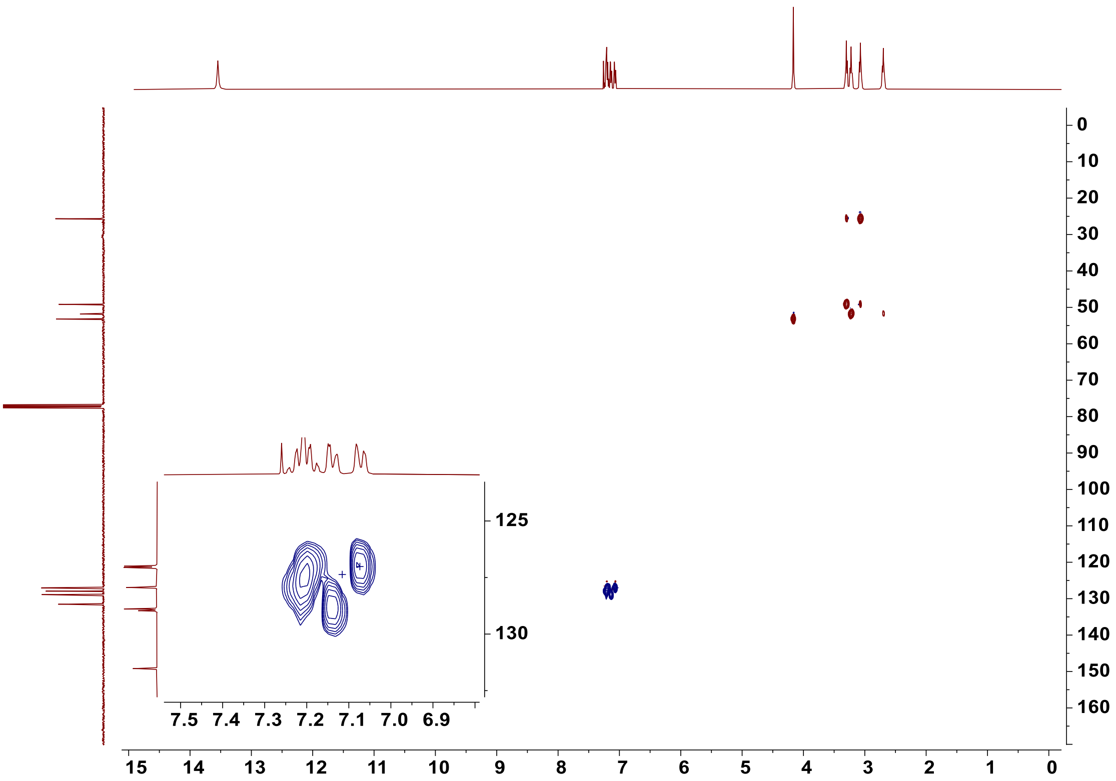

Figure S17. NMR spectra ($CDCl_3$) of **7s**: a) ^{1}H, b) ^{13}C, c) COSY, d) HMBC and e) HSQC.

a)

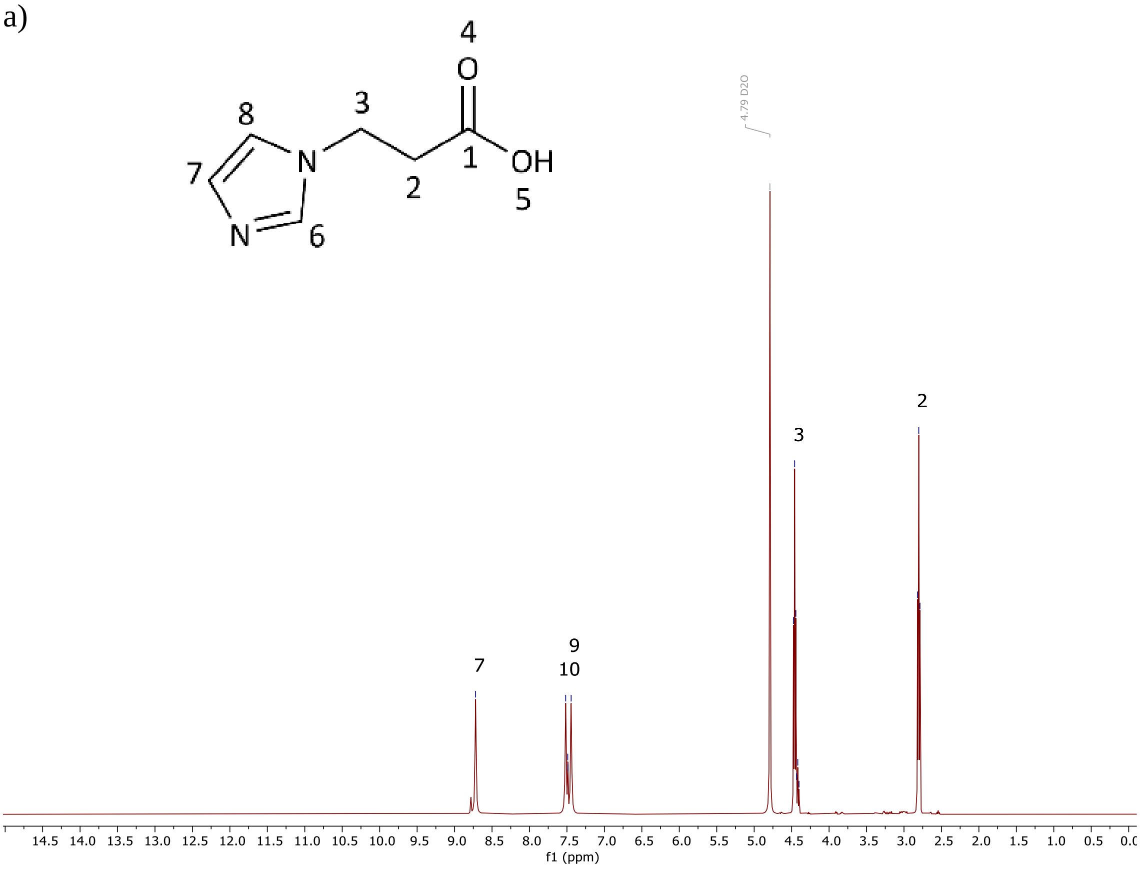
4
O
3
8
N
7
1
OH
2
5
N
6
4.79 D2O
3
2
7
9
10
14.5 14.0 13.5 13.0 12.5 12.0 11.5 11.0 10.5 10.0 9.5 9.0 8.5 8.0 7.5 7.0 6.5 6.0 5.5 5.0 4.5 4.0 3.5 3.0 2.5 2.0 1.5 1.0 0.5 0.0
f1 (ppm)

b)

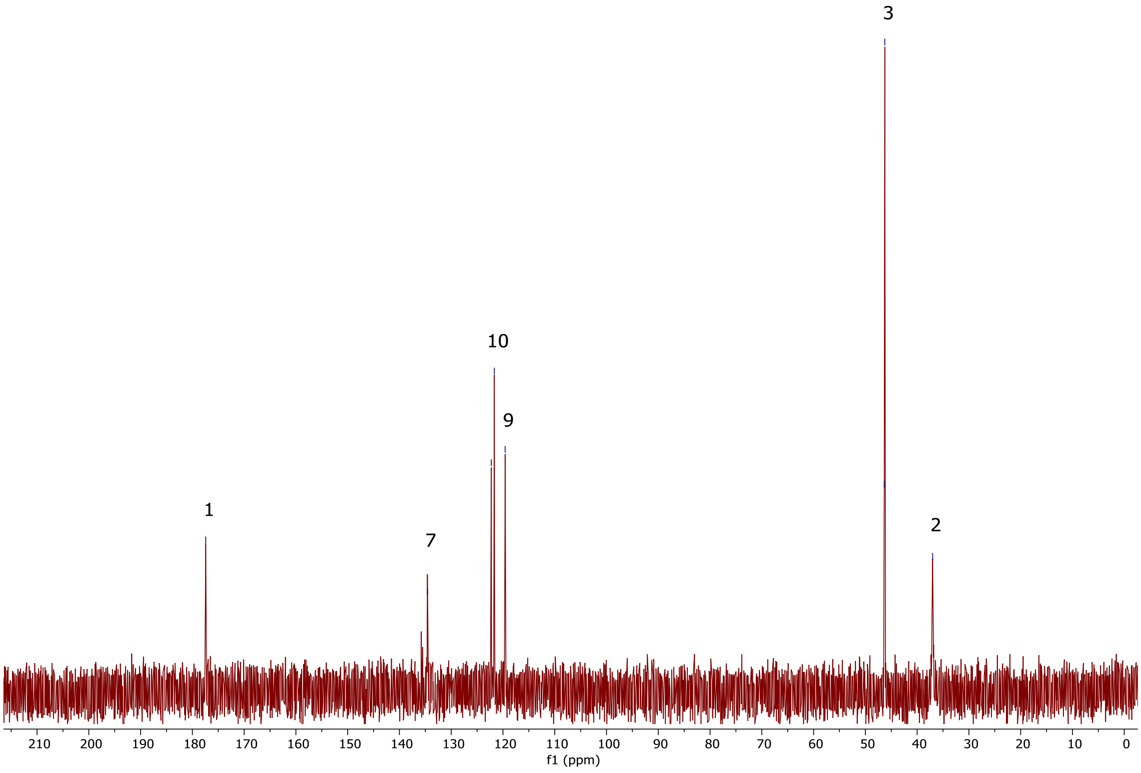
3
10
9
1
7
2
210 200 190 180 170 160 150 140 130 120 110 100 90 80 70 60 50 40 30 20 10 0
f1 (ppm)

c)

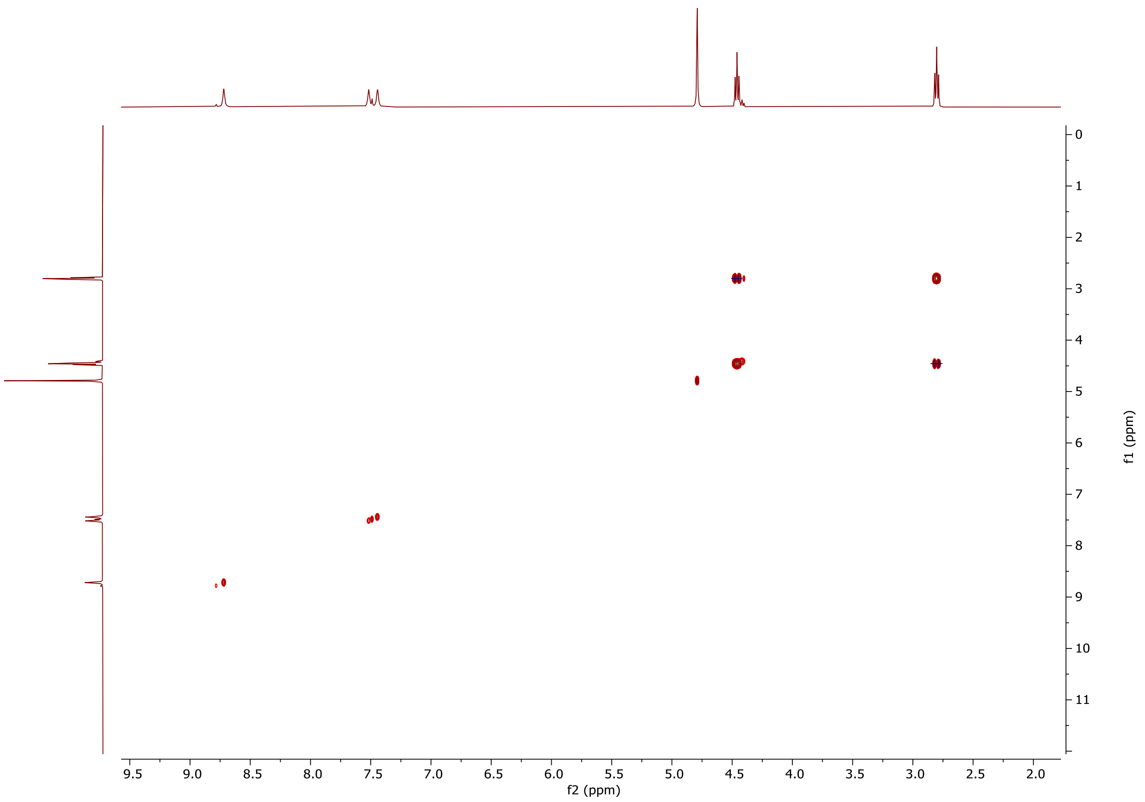

d)

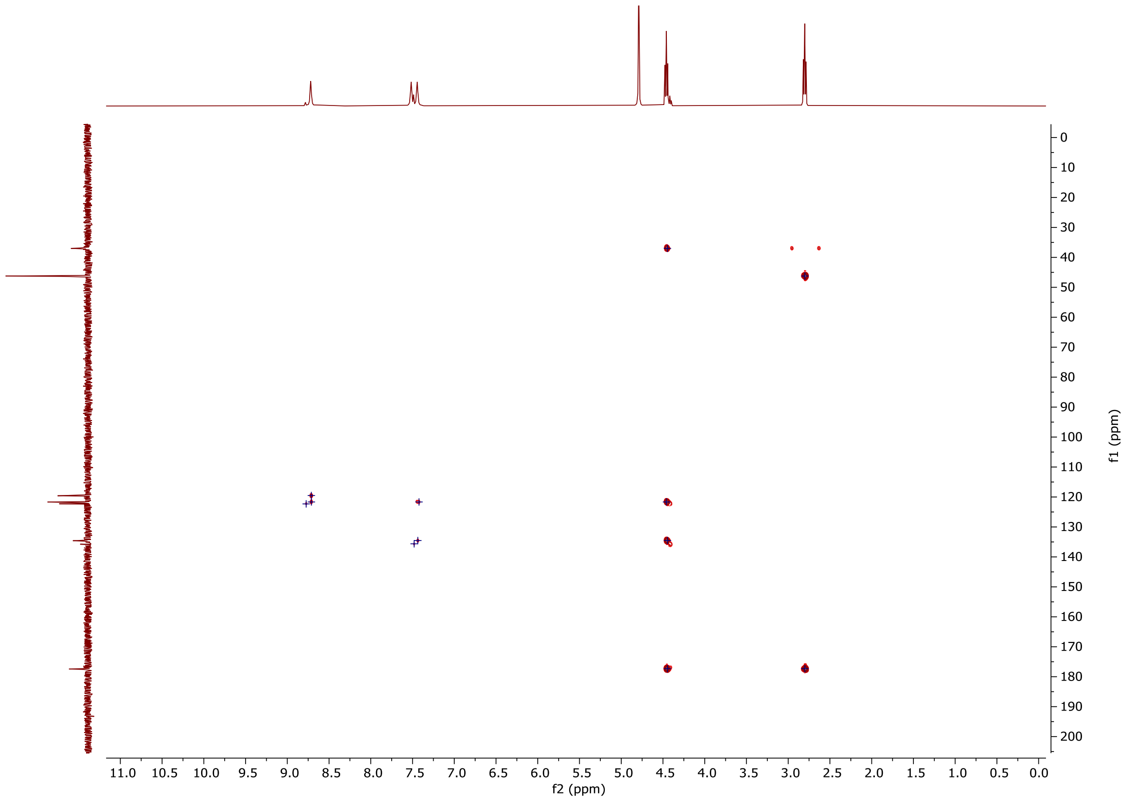

e)

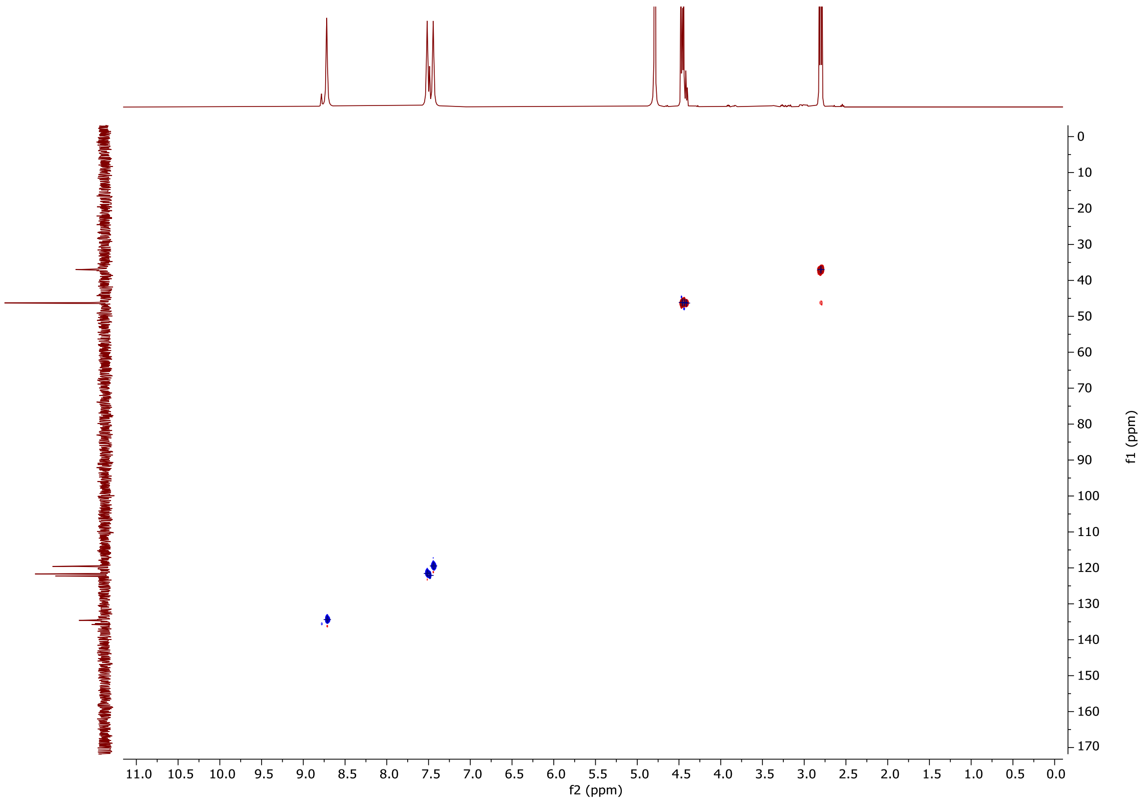

Figure S29. NMR spectra (D_2O) of **7u**: a) ^{1}H, b) ^{13}C, c) COSY, d) HMBC and e) HSQC.

a)

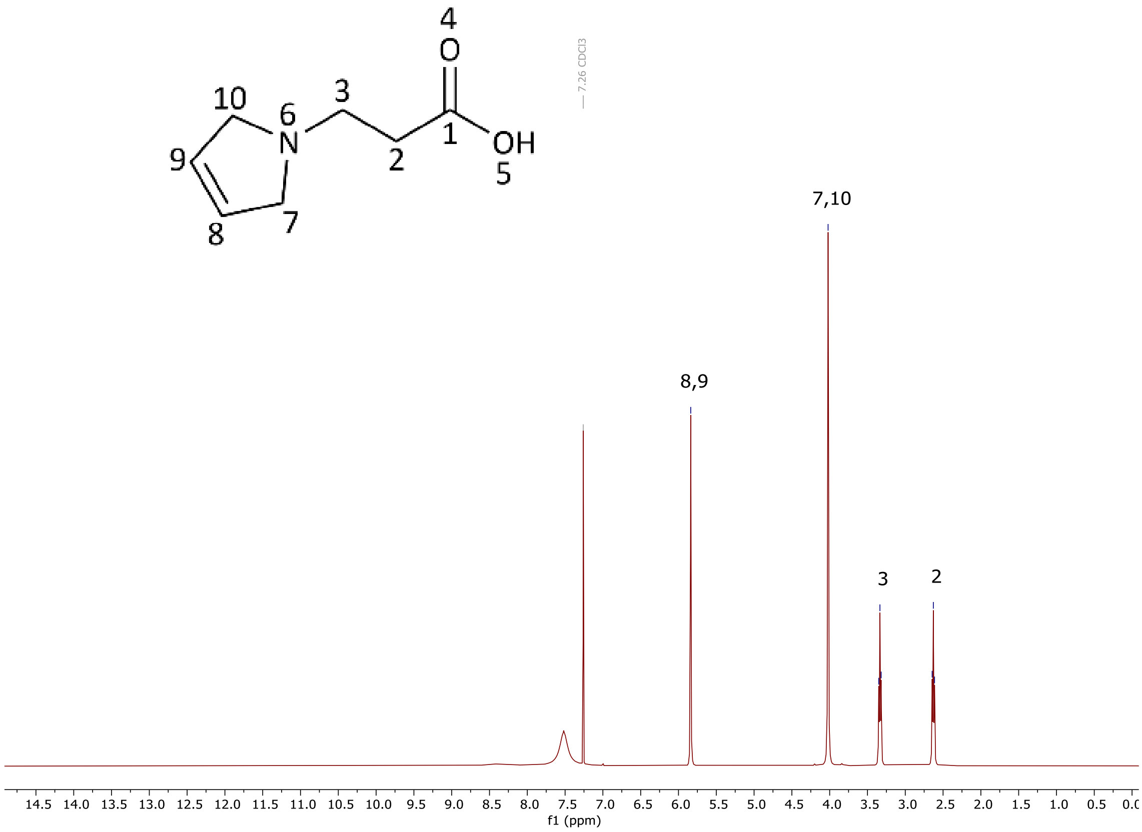

b)

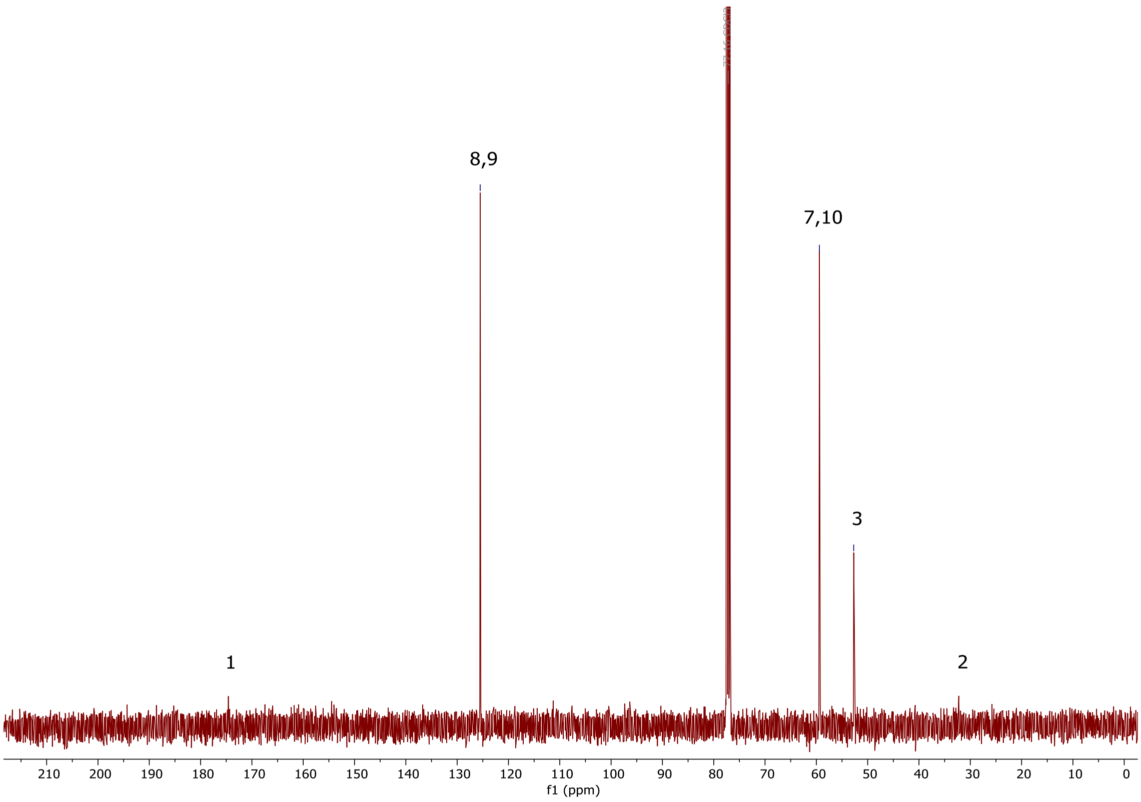
8,9
7,10
3
1
2
210 200 190 180 170 160 150 140 130 120 110 100 90 80 70 60 50 40 30 20 10 0
f1 (ppm)

c)

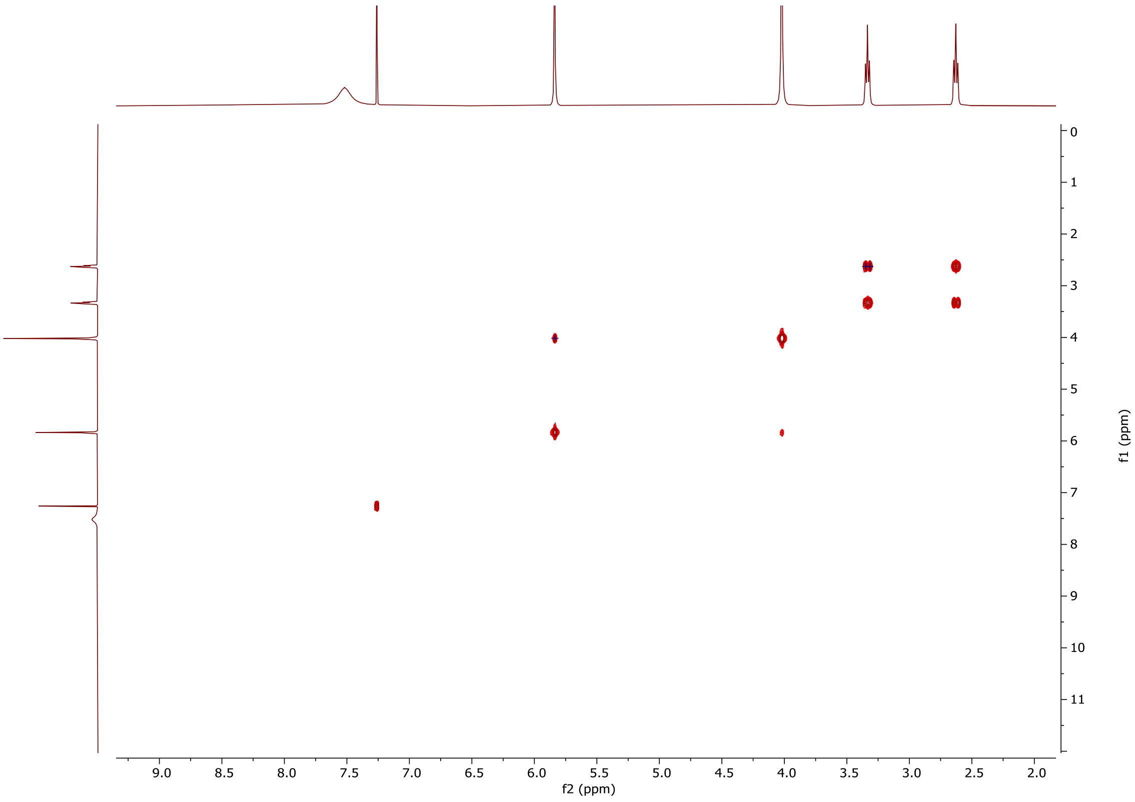
9.0 8.5 8.0 7.5 7.0 6.5 6.0 5.5 5.0 4.5 4.0 3.5 3.0 2.5 2.0
f2 (ppm)
0 1 2 3 4 5 6 7 8 9 10 11
f1 (ppm)

d)

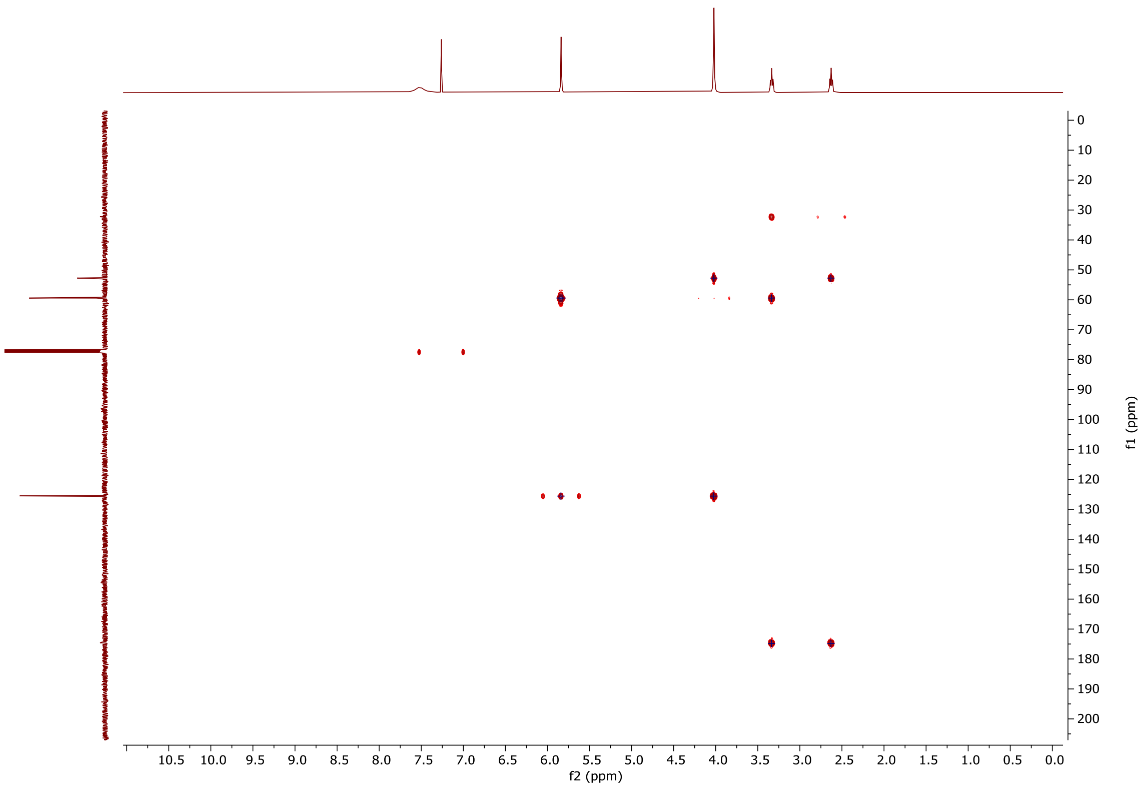

e)

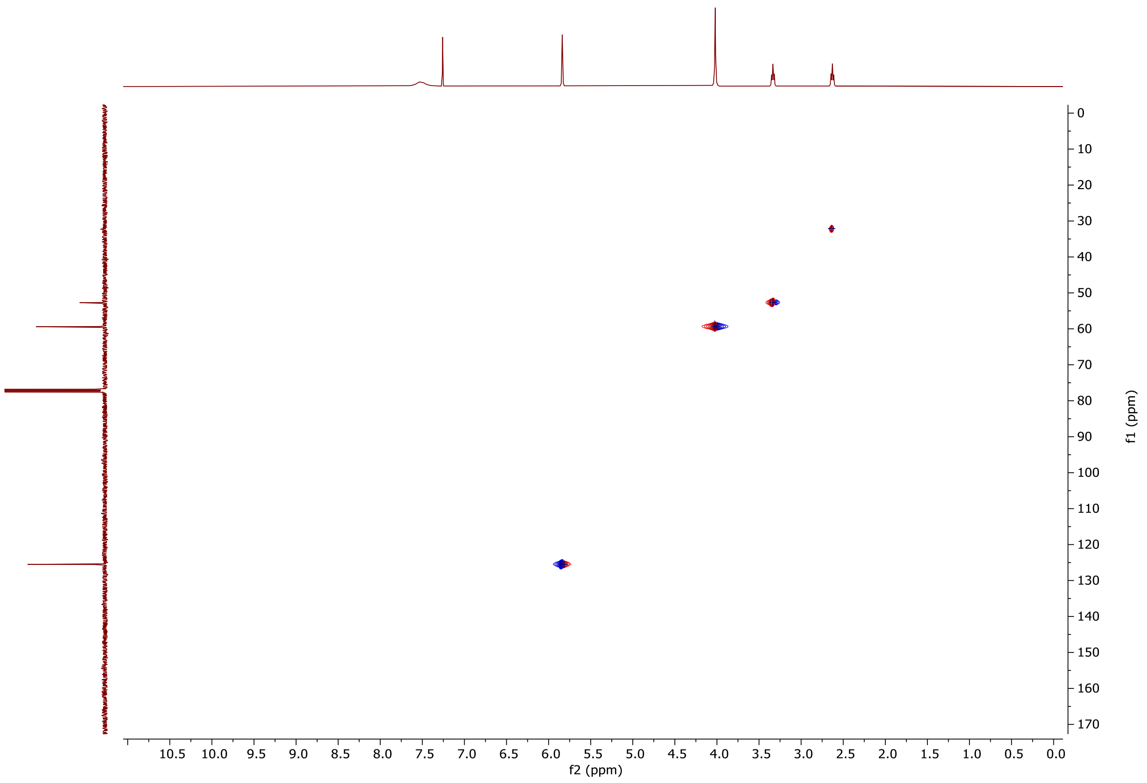

Figure S30. NMR spectra ($CDCl_3$) of **7ad**: a) ^{1}H, b) ^{13}C, c) COSY, d) HMBC and e) HSQC.

a)

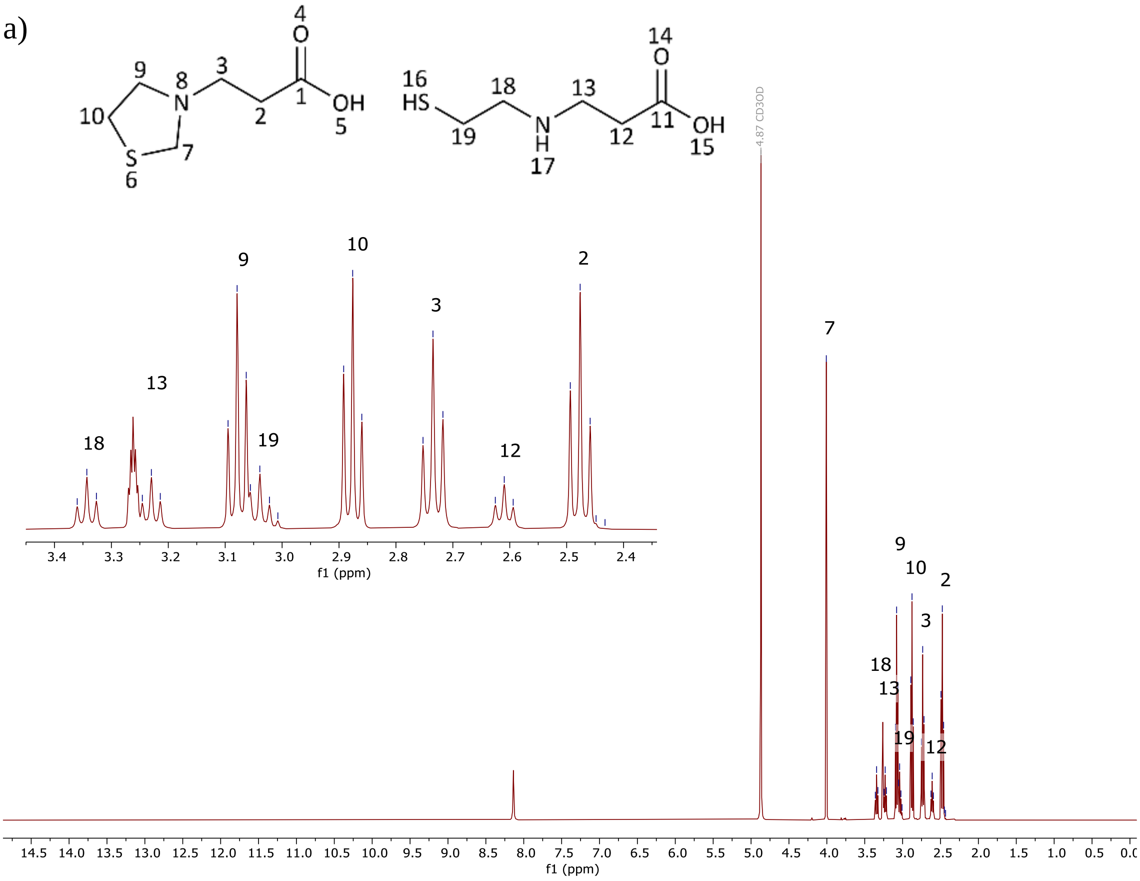

4
O
OH
5
1
2
3
8
N
9
10
S
6
7
16
HS
19
18
N
H
17
13
12
11
14
O
OH
15
4.87 CD3OD
7
9
10
2
3
18
13
19
12
f1 (ppm)

b)

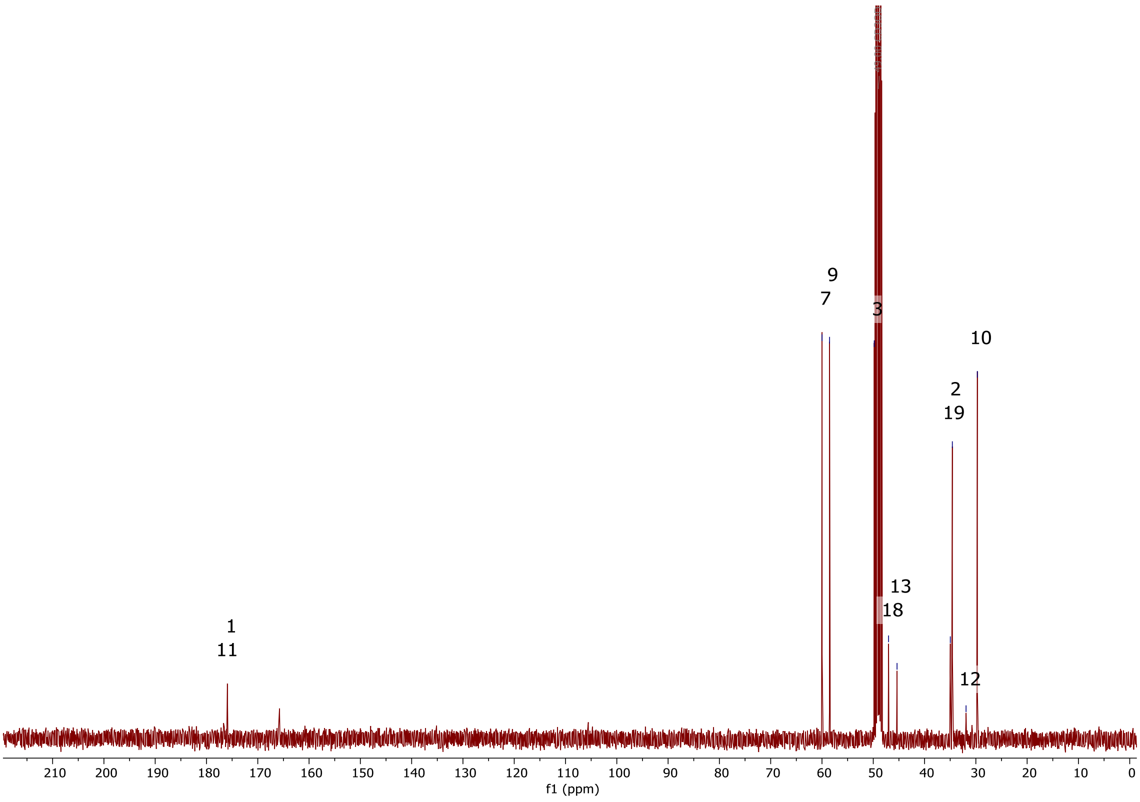

1
11
9
7
3
13
18
2
19
10
12
f1 (ppm)

c)

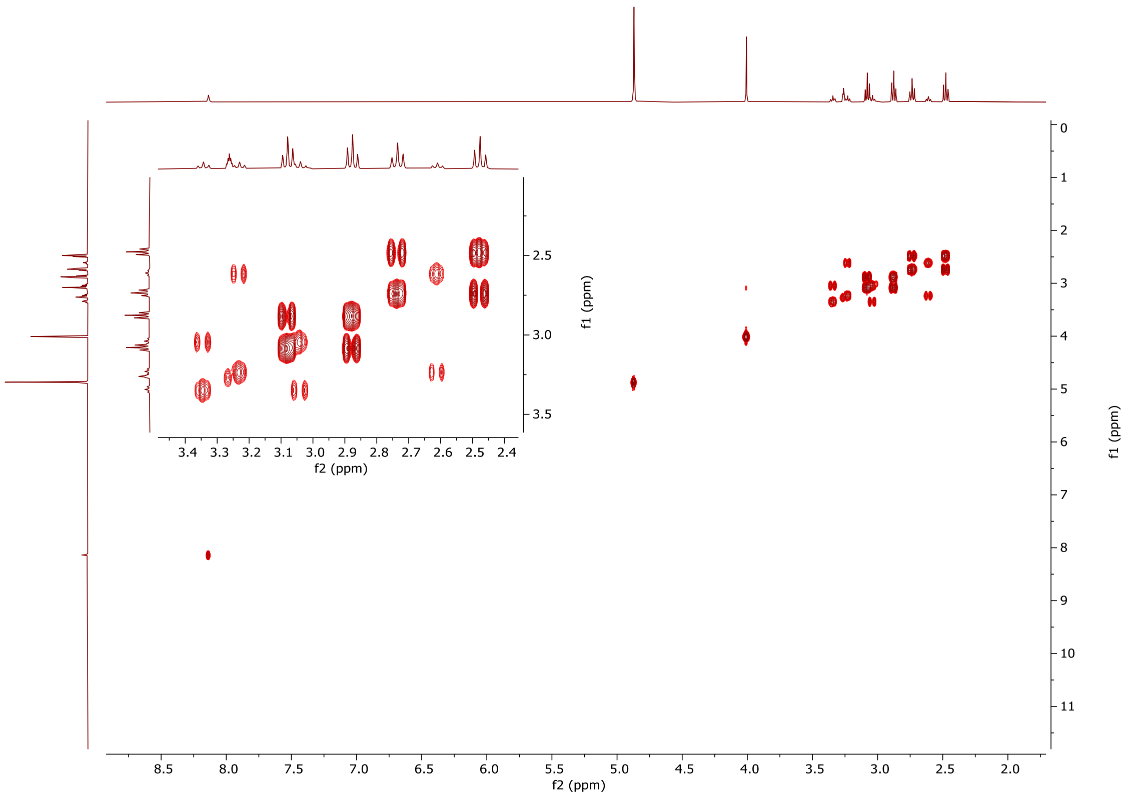

d)

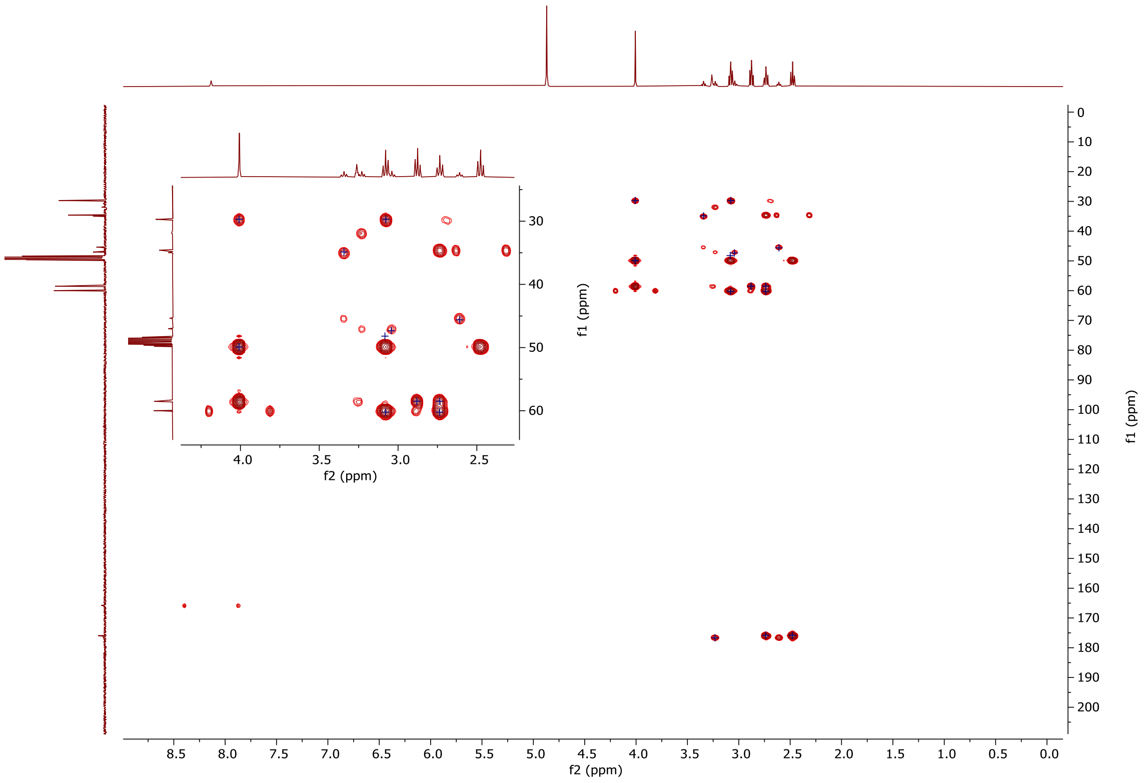

e)

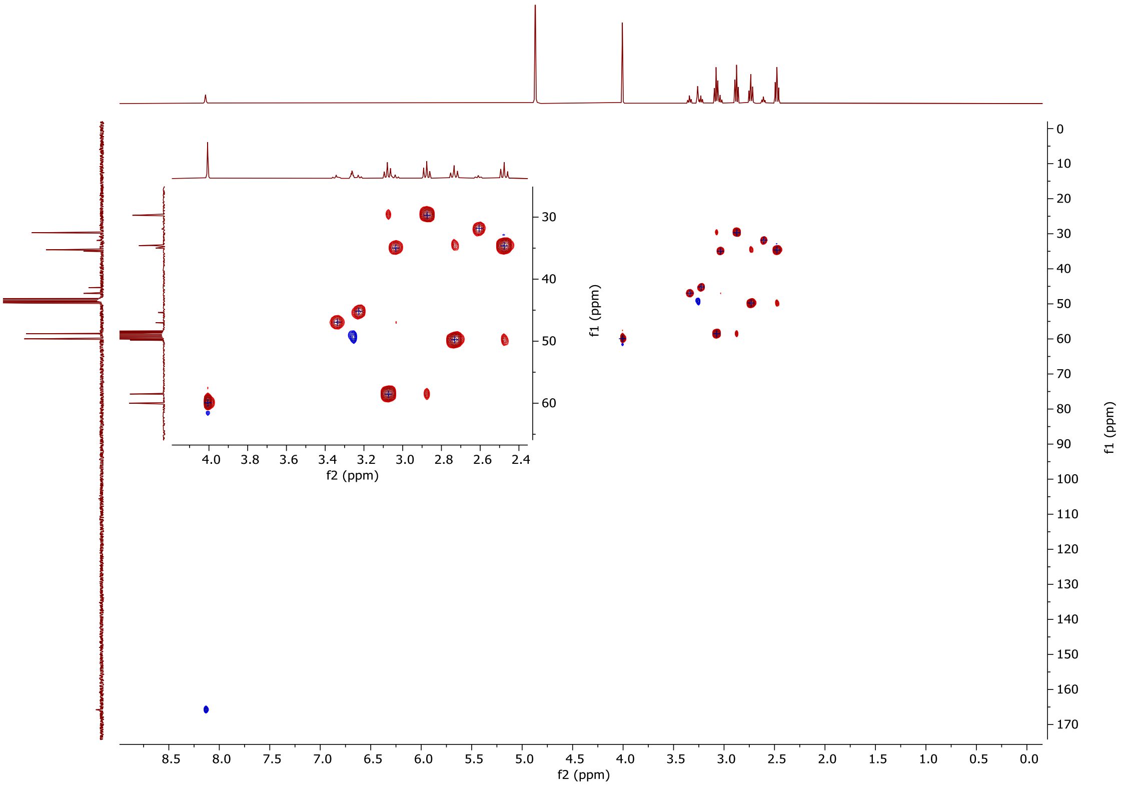

f)

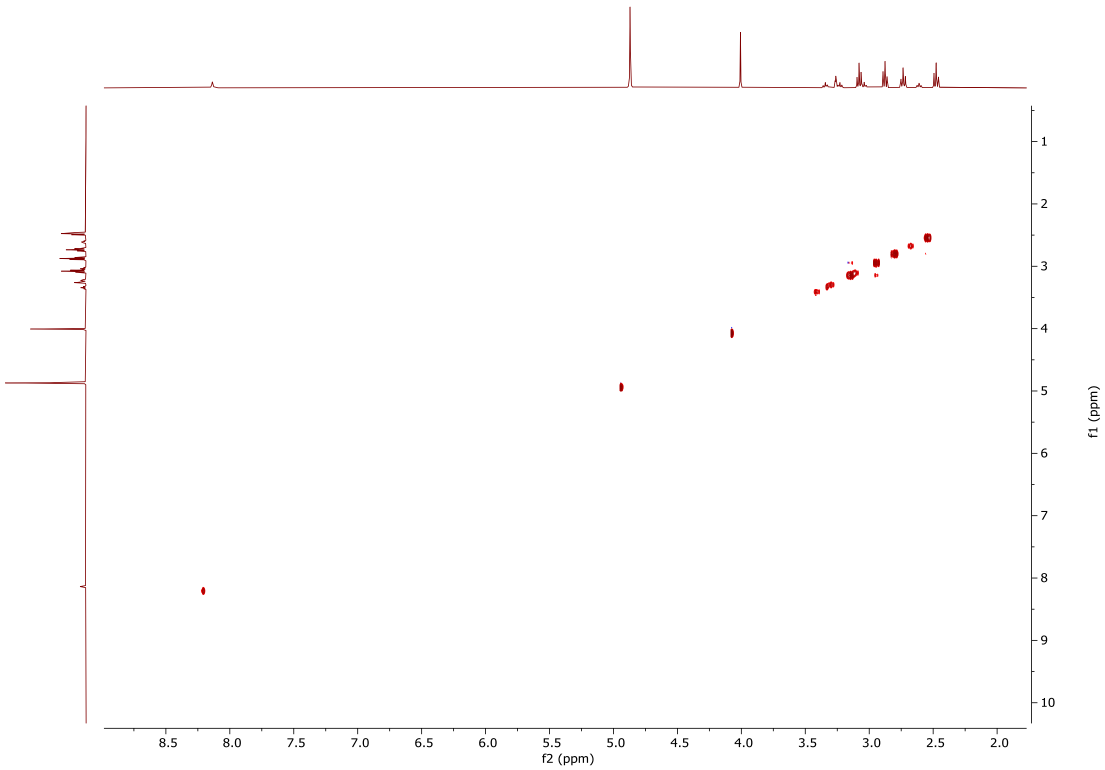

Figure S31. NMR spectra (CD_3OD) of **7ag**: a) ^1H, b) ^{13}C, c) COSY, d) HMBC, e) HSQC and f) NOESY.

a)

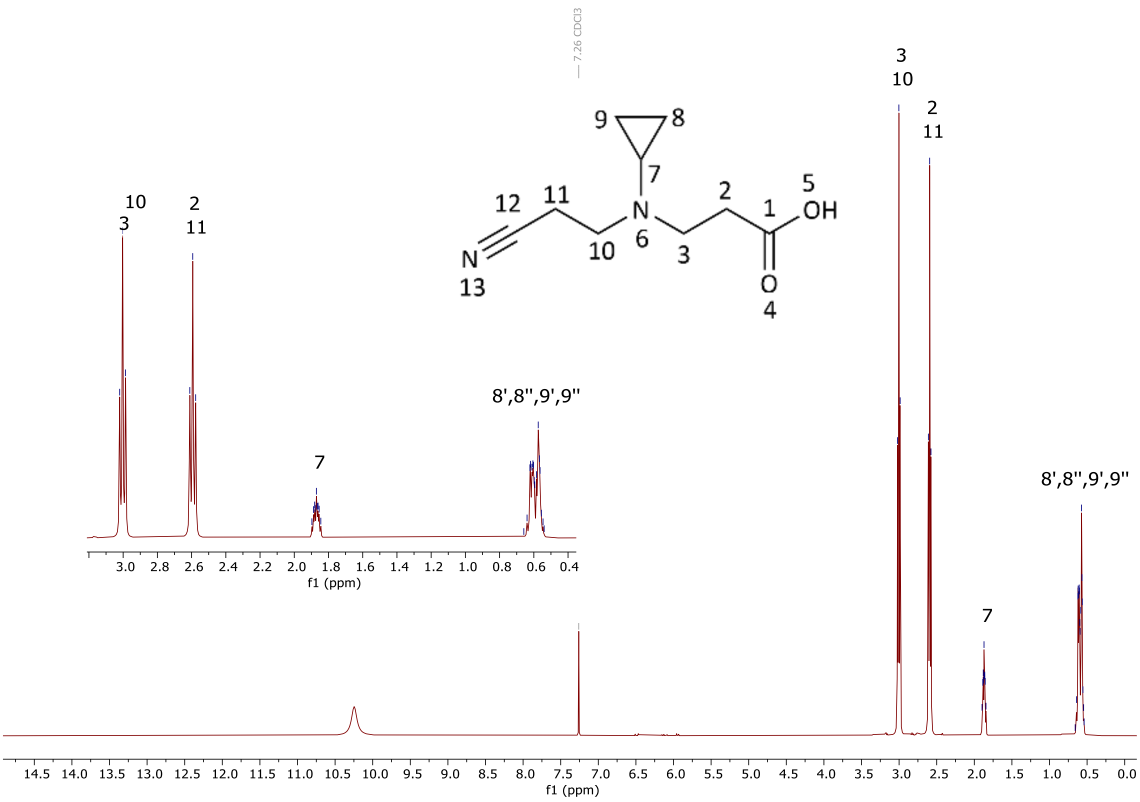

7.26 CDCl3
9
8
7
12
11
N
2
1
5
OH
N
13
10
6
3
O
4
10
3
2
11
7
8',8'',9',9''
3.0 2.8 2.6 2.4 2.2 2.0 1.8 1.6 1.4 1.2 1.0 0.8 0.6 0.4
f1 (ppm)
3
10
2
11
7
8',8'',9',9''
14.5 14.0 13.5 13.0 12.5 12.0 11.5 11.0 10.5 10.0 9.5 9.0 8.5 8.0 7.5 7.0 6.5 6.0 5.5 5.0 4.5 4.0 3.5 3.0 2.5 2.0 1.5 1.0 0.5 0.0
f1 (ppm)

b)

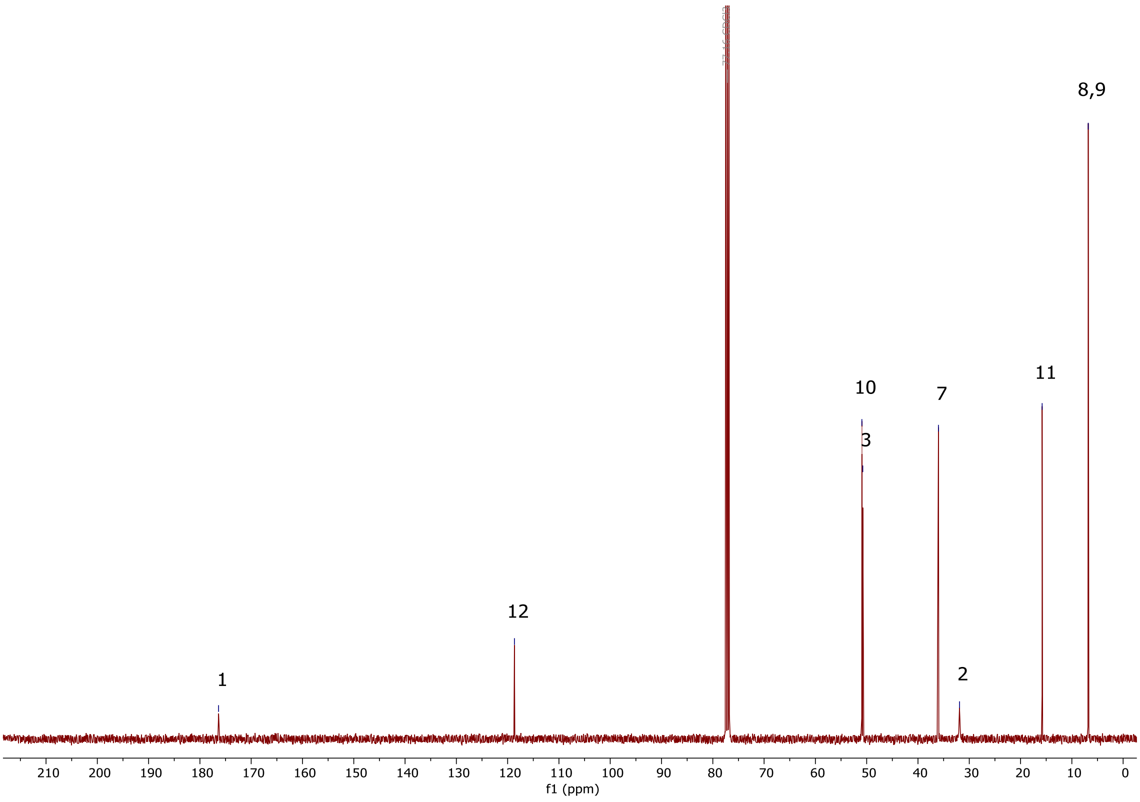

8,9
10
3
7
11
12
1
2
210 200 190 180 170 160 150 140 130 120 110 100 90 80 70 60 50 40 30 20 10 0
f1 (ppm)

c)

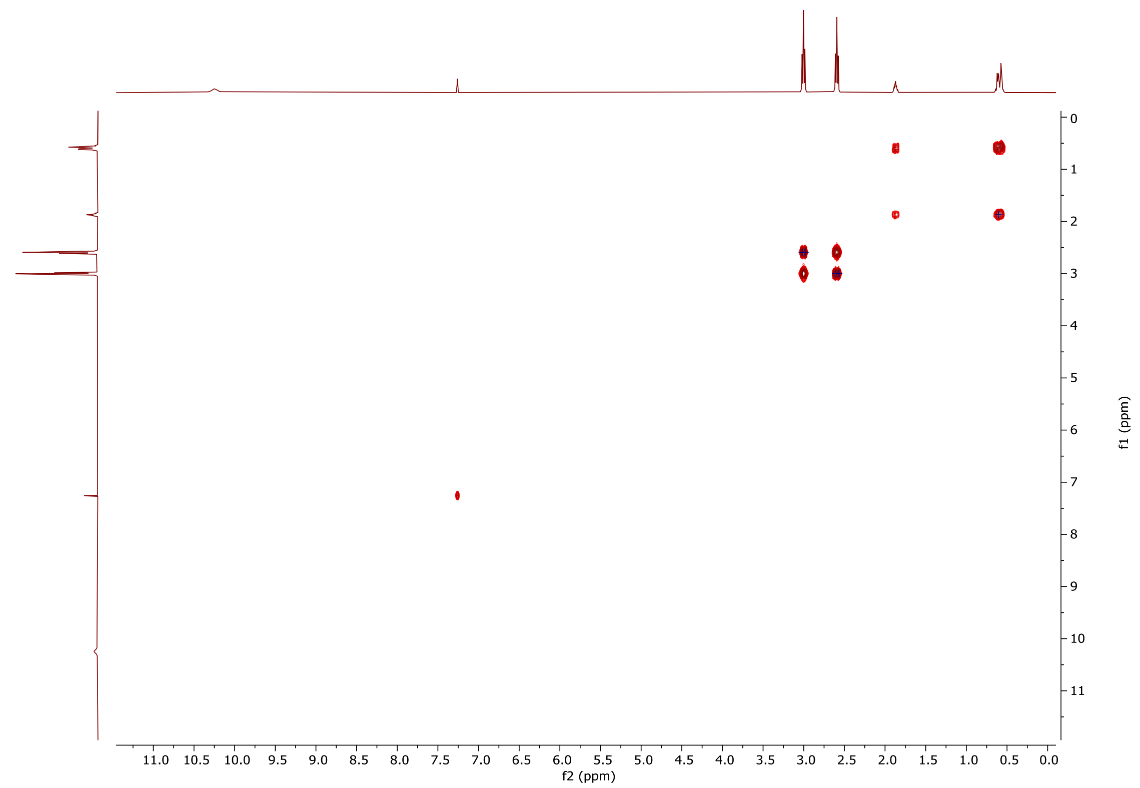

11.0 10.5 10.0 9.5 9.0 8.5 8.0 7.5 7.0 6.5 6.0 5.5 5.0 4.5 4.0 3.5 3.0 2.5 2.0 1.5 1.0 0.5 0.0
f2 (ppm)
0 1 2 3 4 5 6 7 8 9 10 11
f1 (ppm)

d)

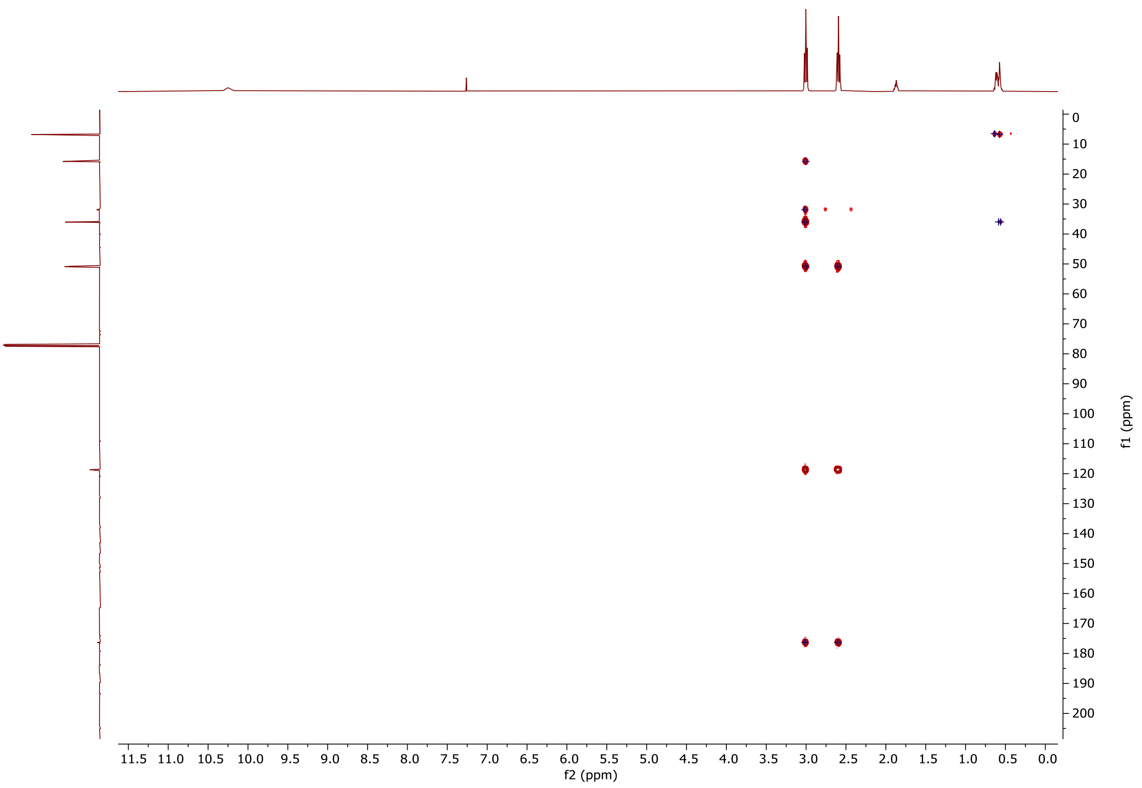

11.5 11.0 10.5 10.0 9.5 9.0 8.5 8.0 7.5 7.0 6.5 6.0 5.5 5.0 4.5 4.0 3.5 3.0 2.5 2.0 1.5 1.0 0.5 0.0
f2 (ppm)
0 10 20 30 40 50 60 70 80 90 100 110 120 130 140 150 160 170 180 190 200
f1 (ppm)

e)

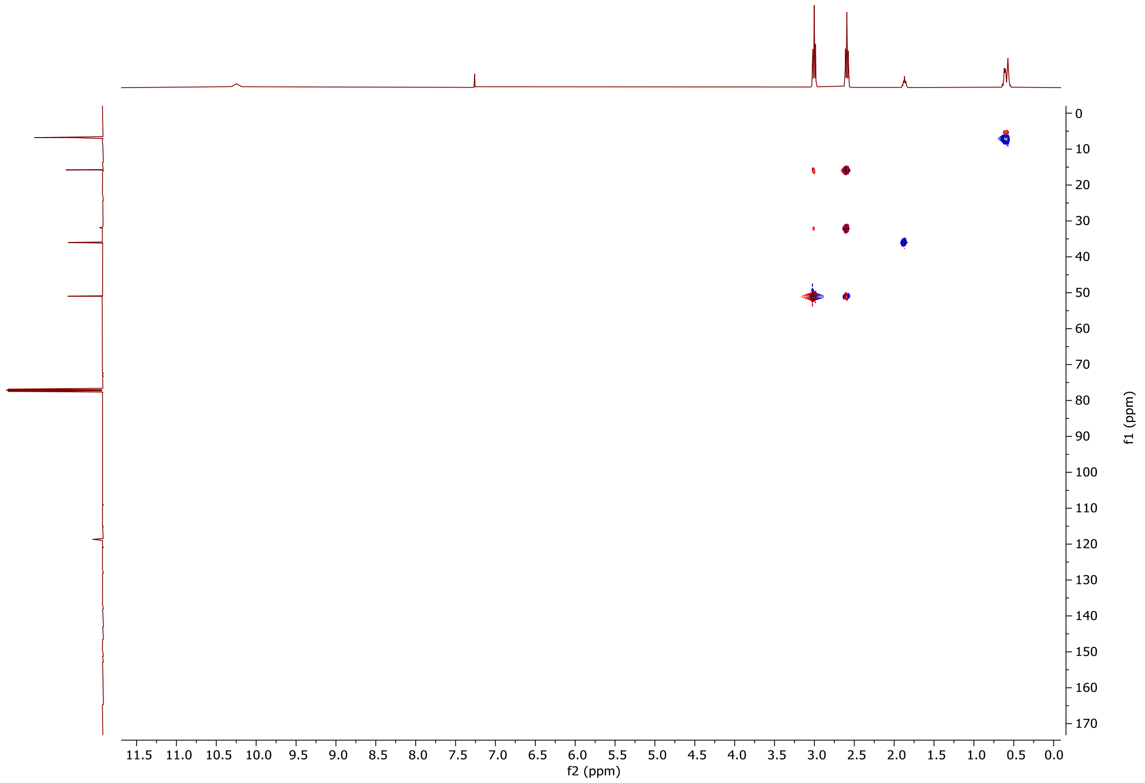

Figure S32. NMR spectra ($CDCl_3$) of **7ah**: a) ^1H, b) ^{13}C, c) COSY, d) HMBC and e) HSQC.

a)

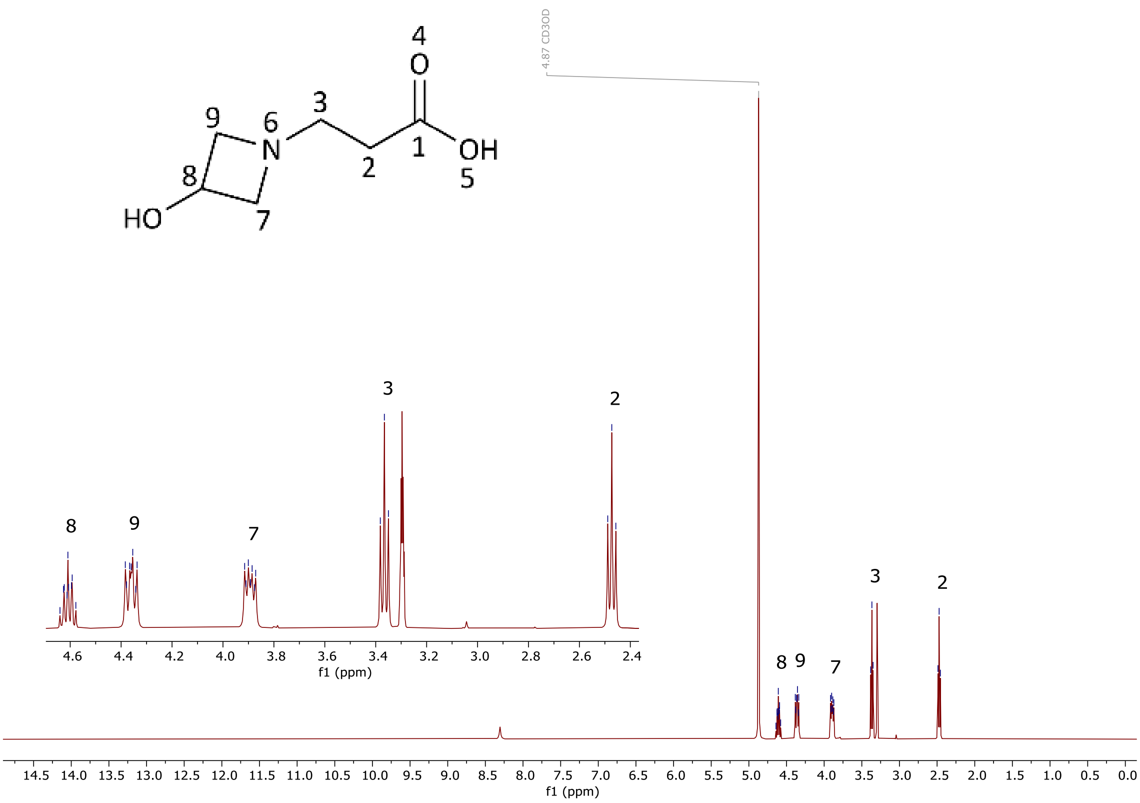

b)

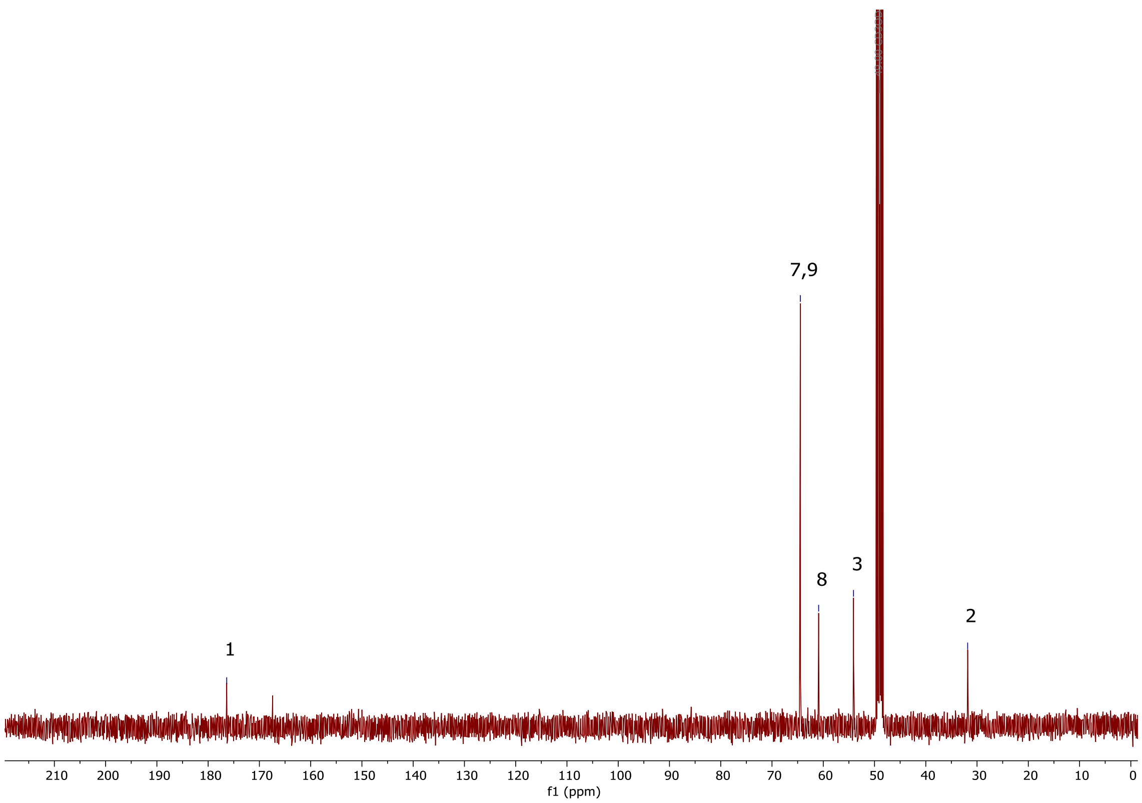
7,9
8
3
2
1
210
200
190
180
170
160
150
140
130
120
110
100
90
80
70
60
50
40
30
20
10
0
f1 (ppm)

c)

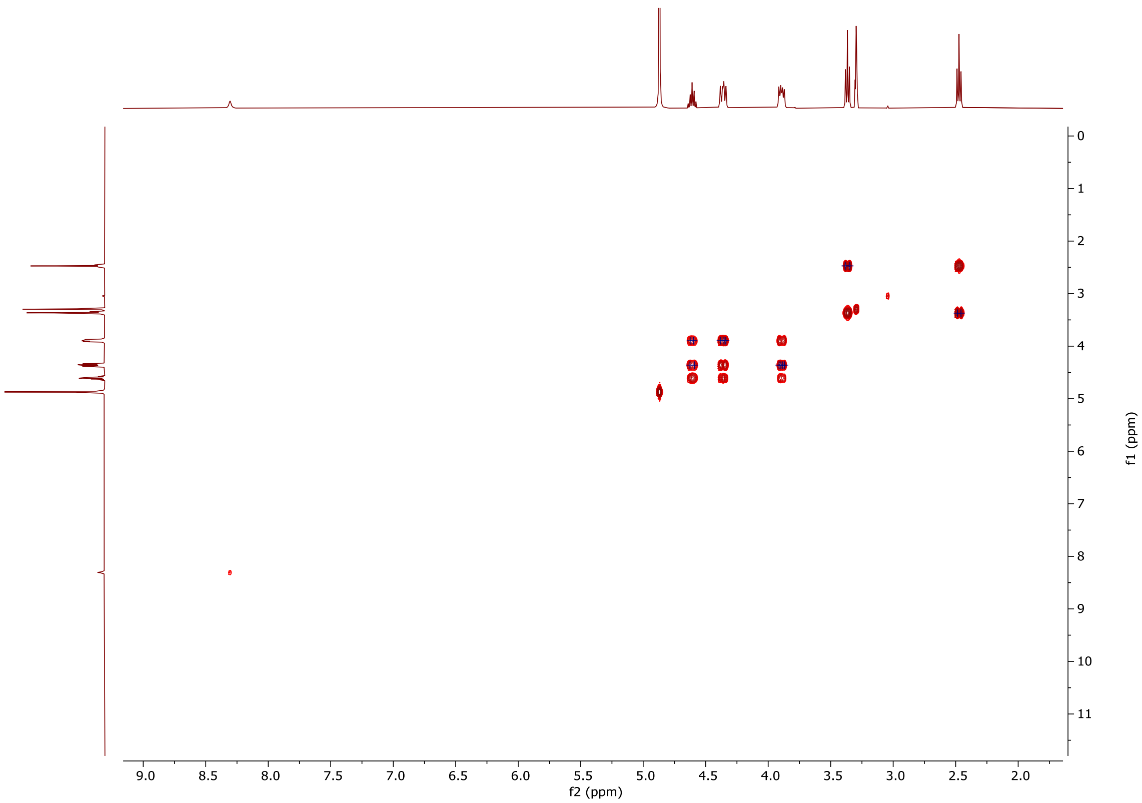
9.0
8.5
8.0
7.5
7.0
6.5
6.0
5.5
5.0
4.5
4.0
3.5
3.0
2.5
2.0
f2 (ppm)
f1 (ppm)

d)

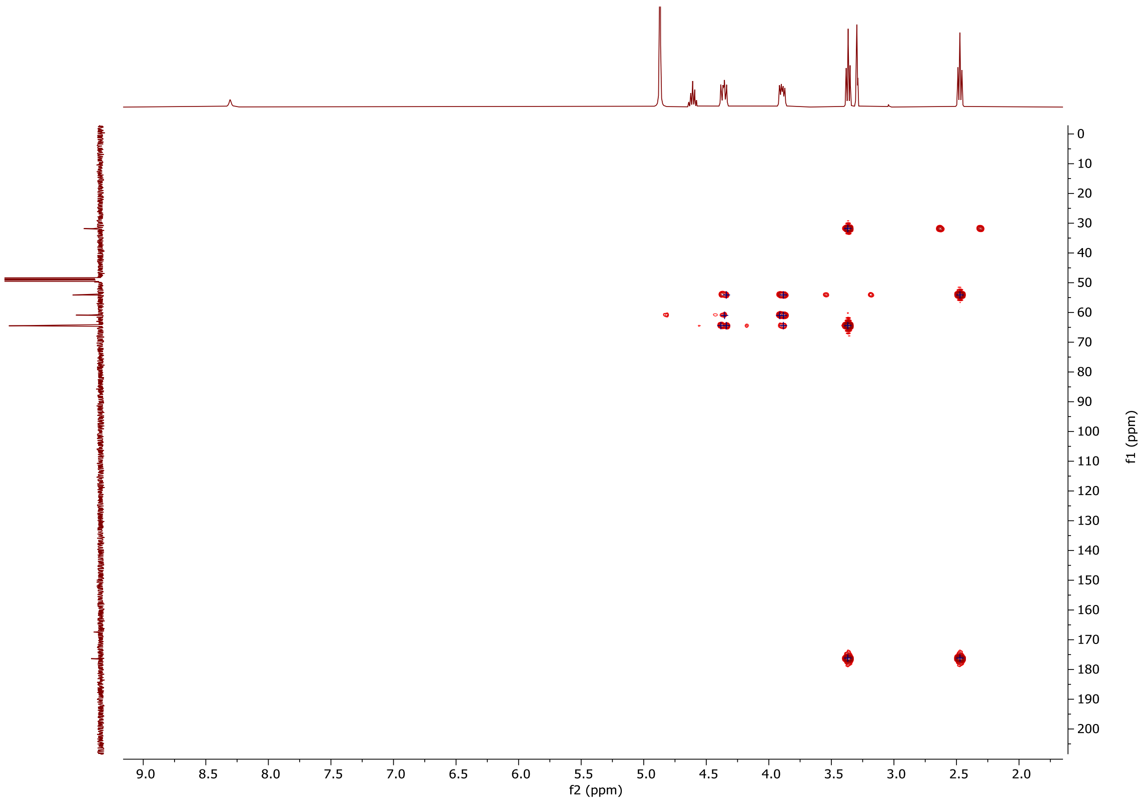

e)

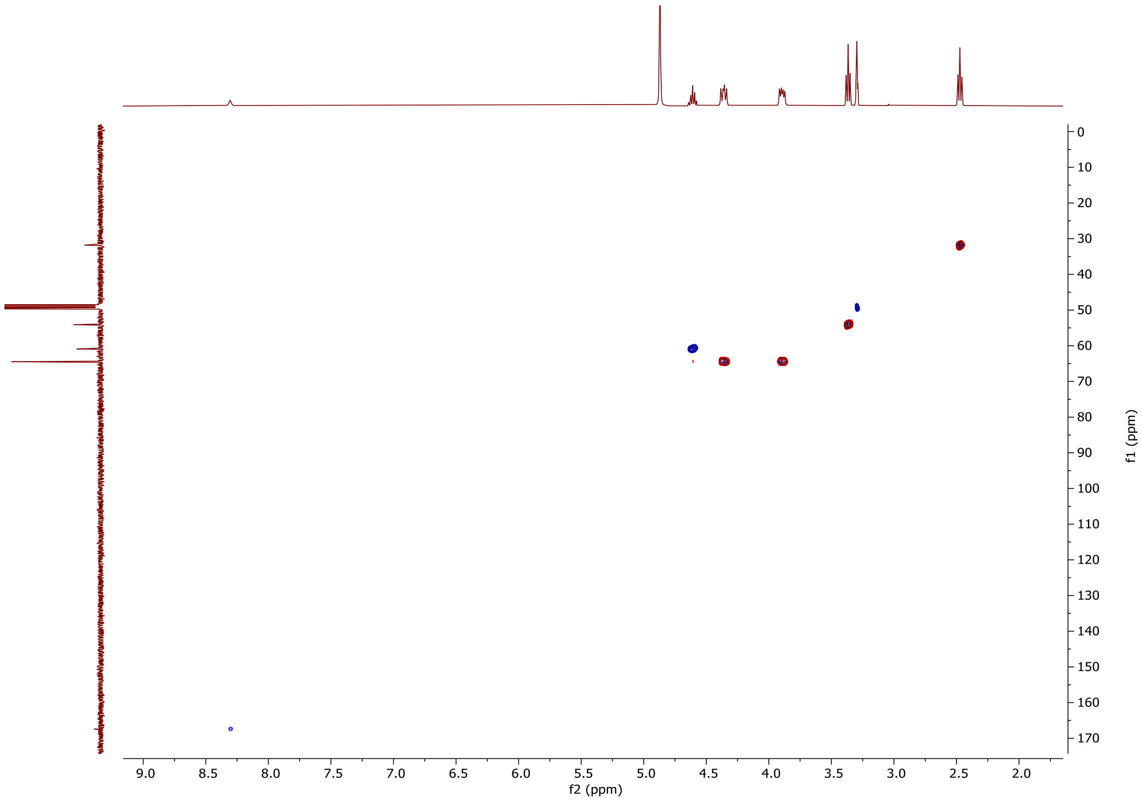

Figure S33. NMR spectra (CD_3OD) of **7aj**: a) ^{1}H, b) ^{13}C, c) COSY, d) HMBC and e) HSQC.

a)

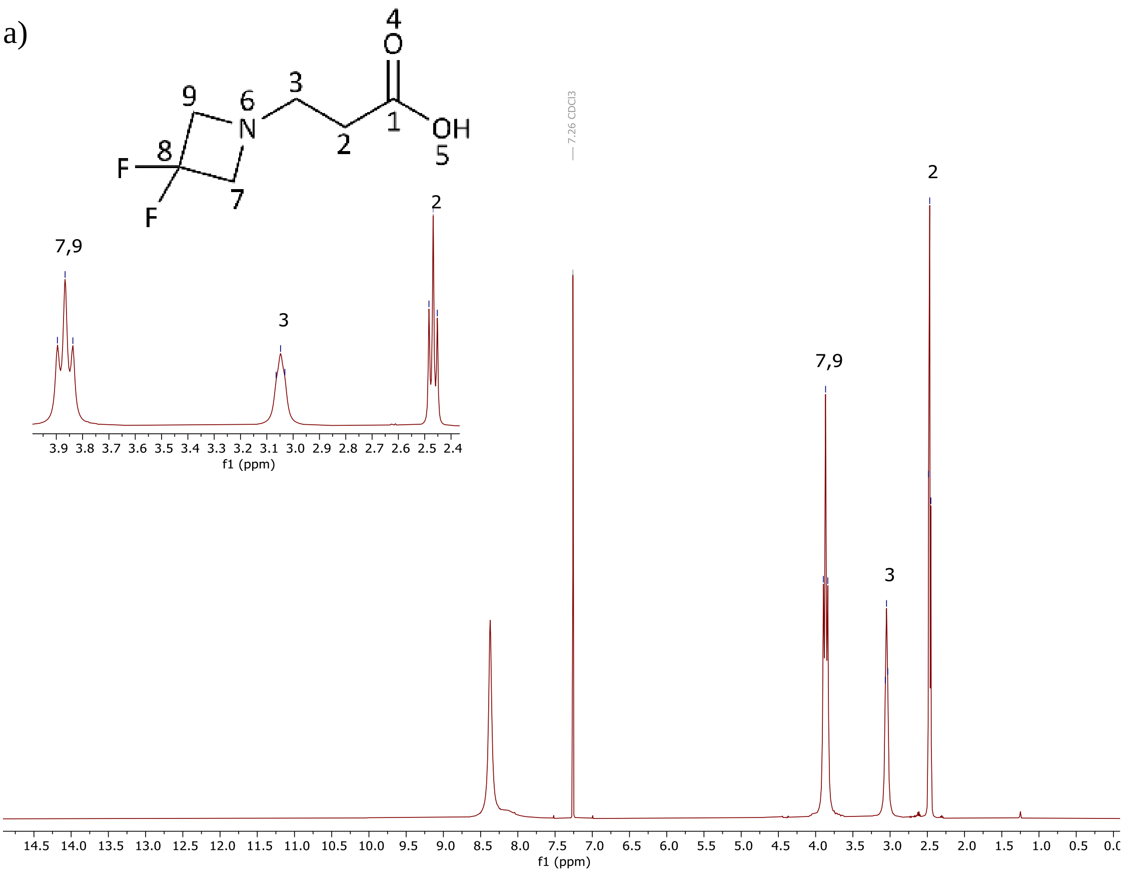

b)

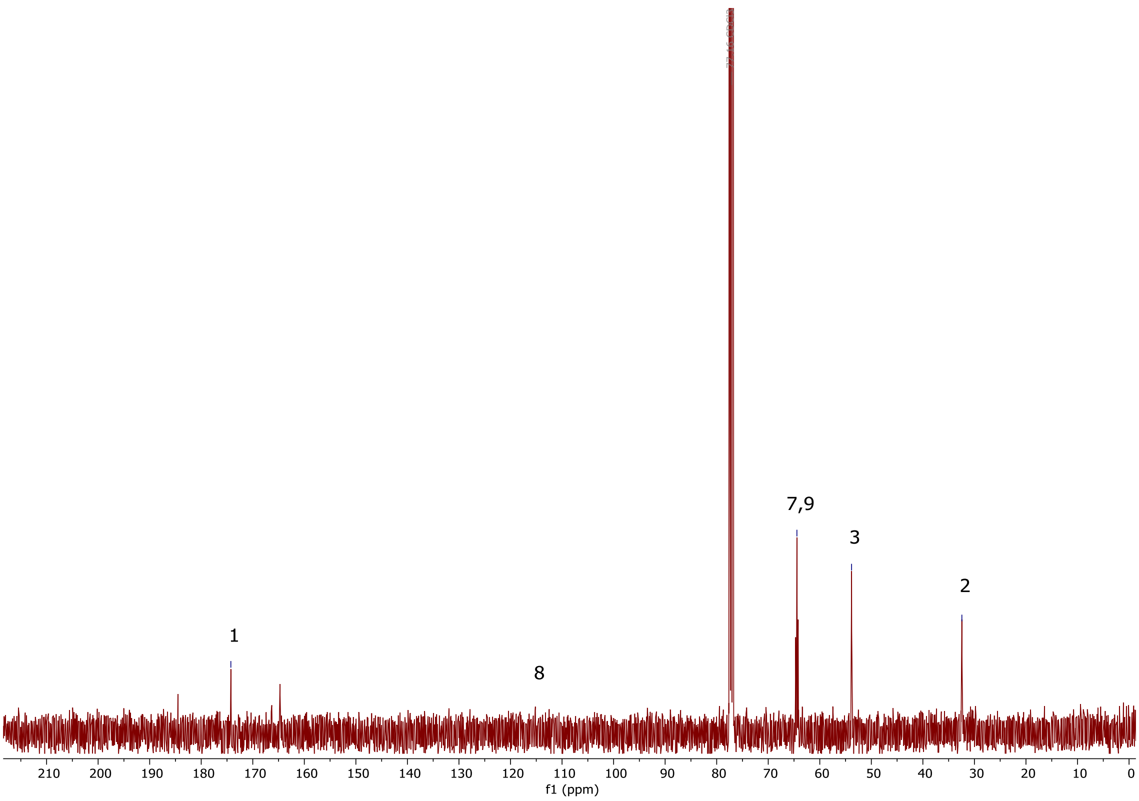

c)

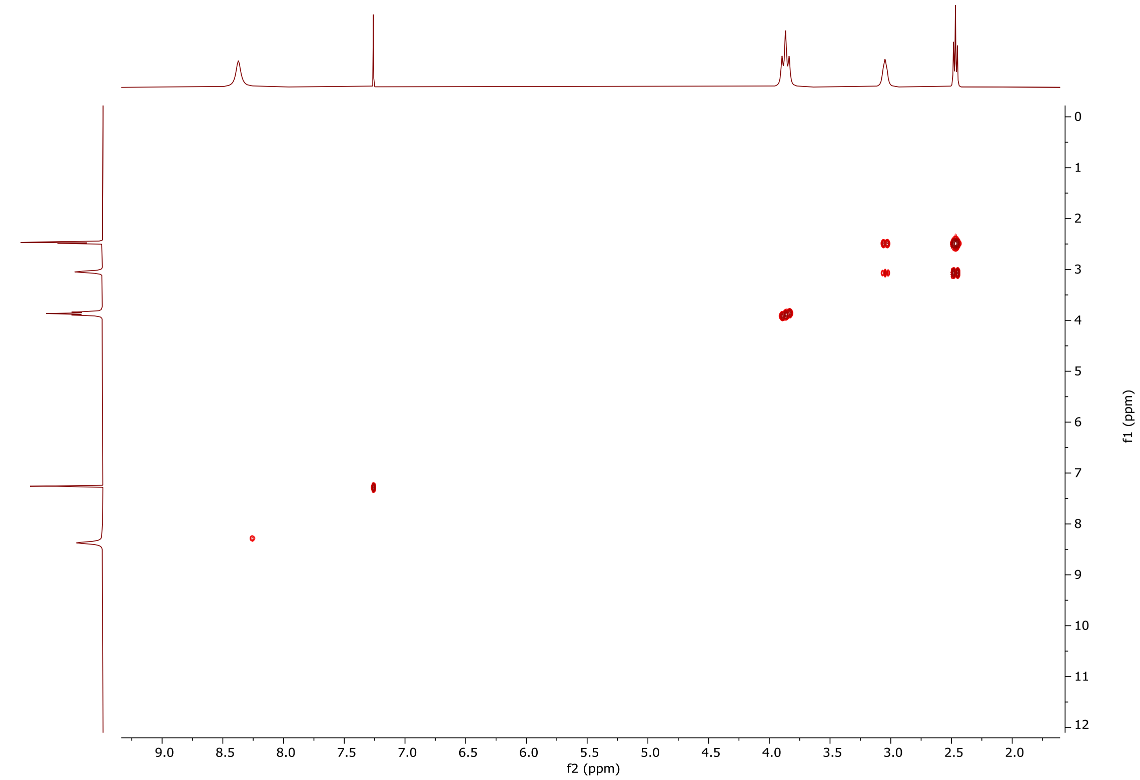

9.0 8.5 8.0 7.5 7.0 6.5 6.0 5.5 5.0 4.5 4.0 3.5 3.0 2.5 2.0
f2 (ppm)
0 1 2 3 4 5 6 7 8 9 10 11 12
f1 (ppm)

d)

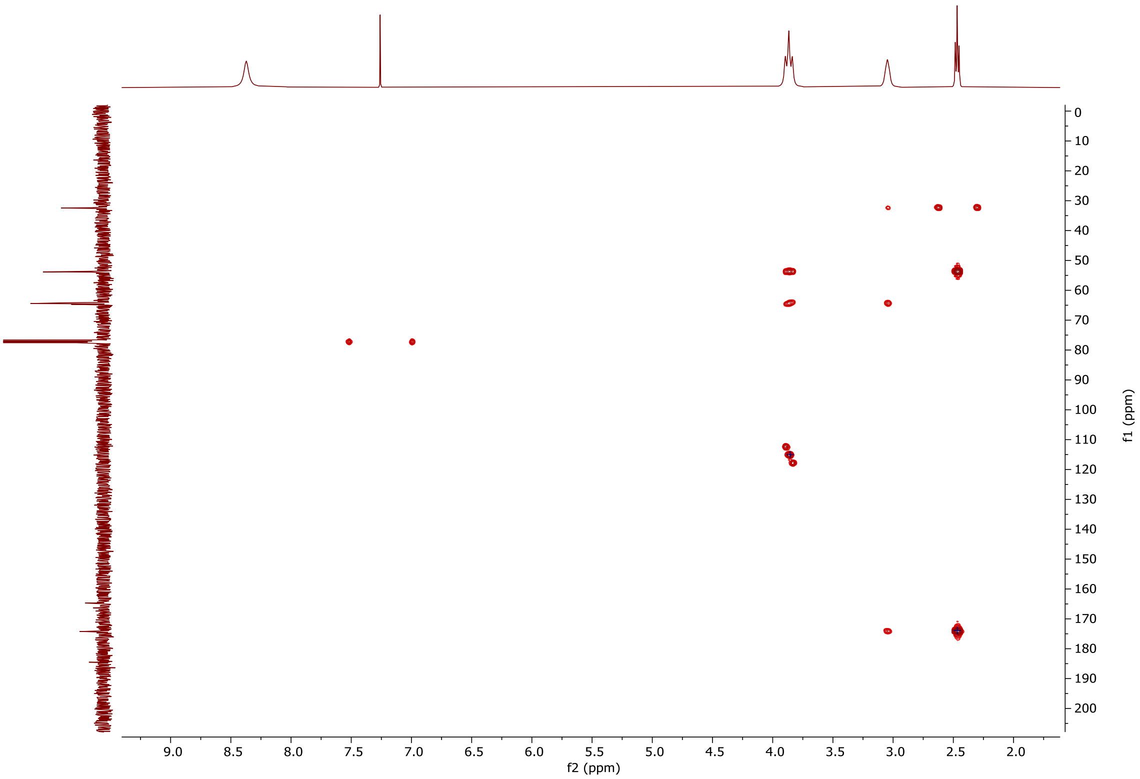

9.0 8.5 8.0 7.5 7.0 6.5 6.0 5.5 5.0 4.5 4.0 3.5 3.0 2.5 2.0
f2 (ppm)
0 10 20 30 40 50 60 70 80 90 100 110 120 130 140 150 160 170 180 190 200
f1 (ppm)

e)

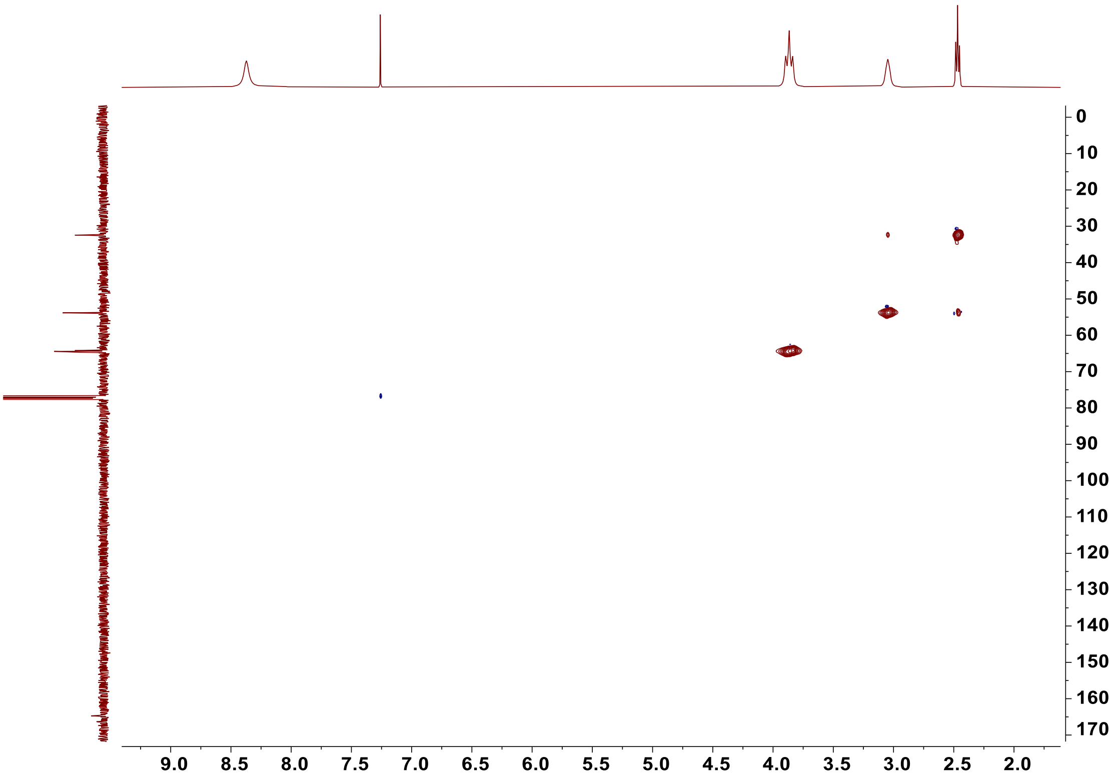

Figure S34. NMR spectra ($CDCl_3$) of **7ak**: a) ^1H, b) ^{13}C, c) COSY, d) HMBC and e) HSQC.

a)

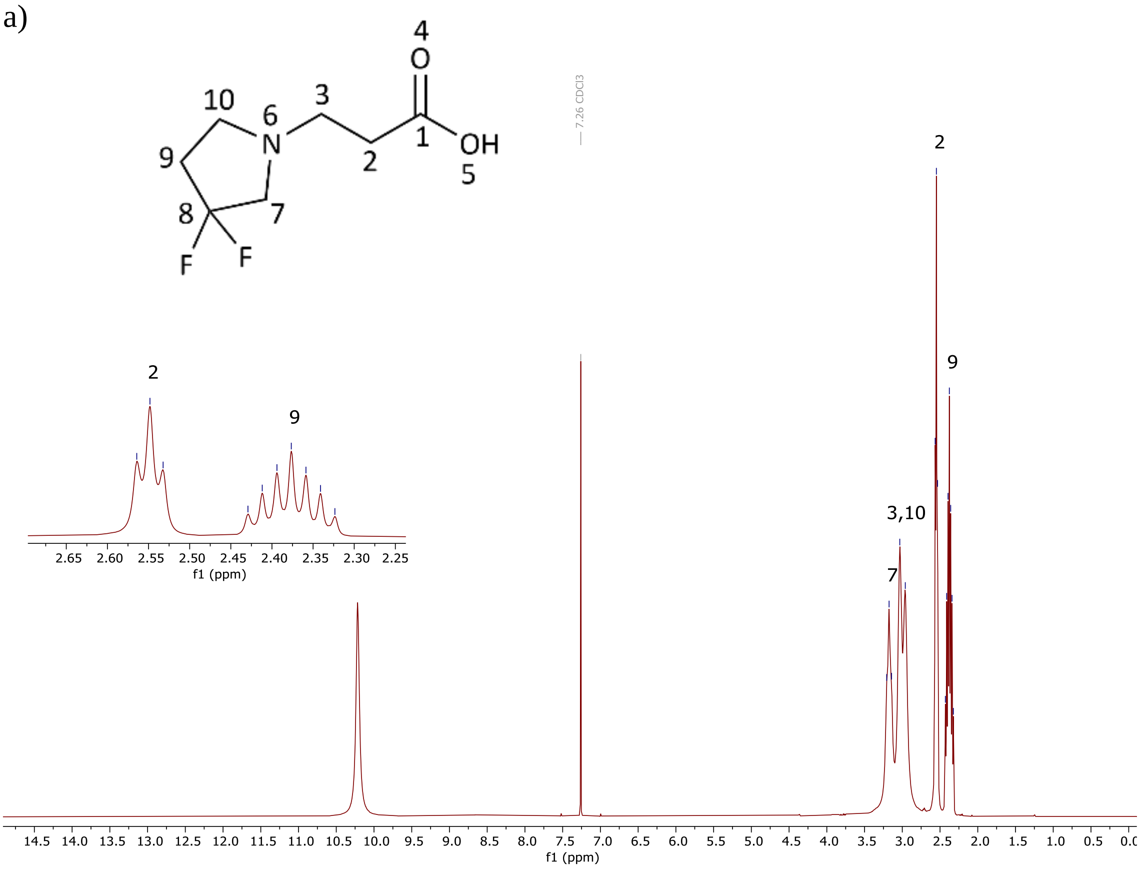

b)

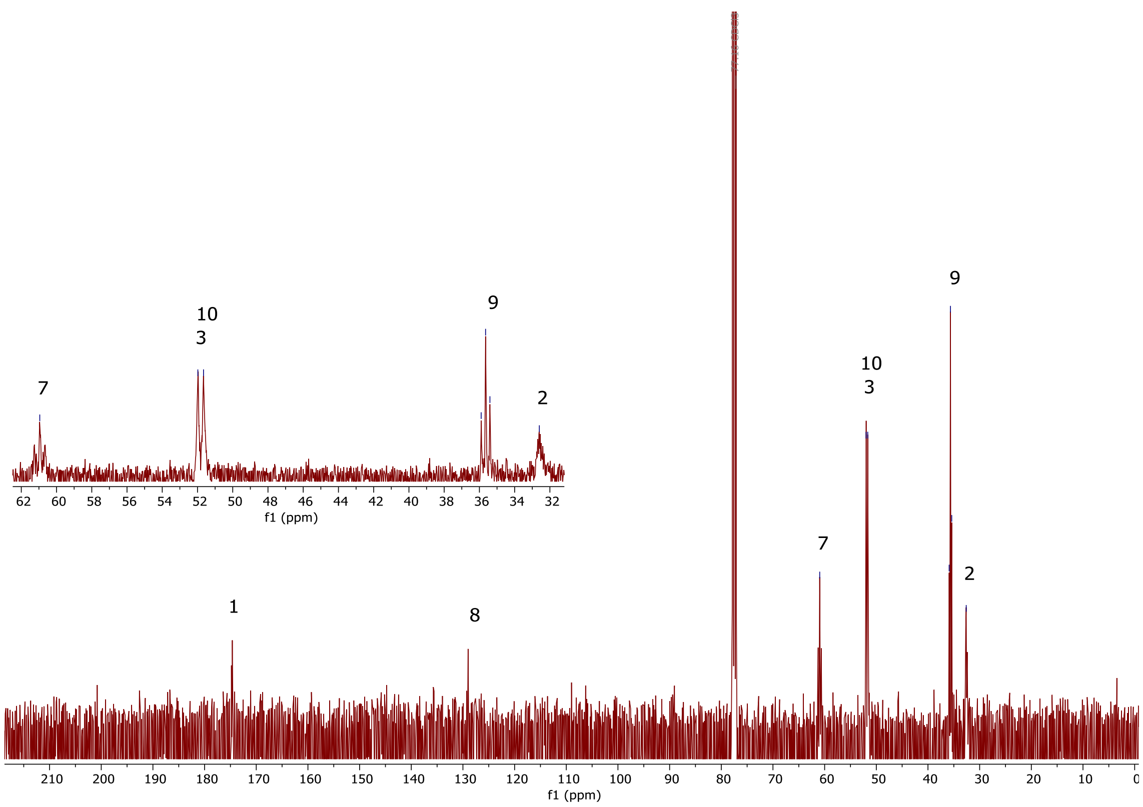
7
10
3
9
2
62 60 58 56 54 52 50 48 46 44 42 40 38 36 34 32
f1 (ppm)
1
8
7
10
3
9
2
210 200 190 180 170 160 150 140 130 120 110 100 90 80 70 60 50 40 30 20 10 0
f1 (ppm)

c)

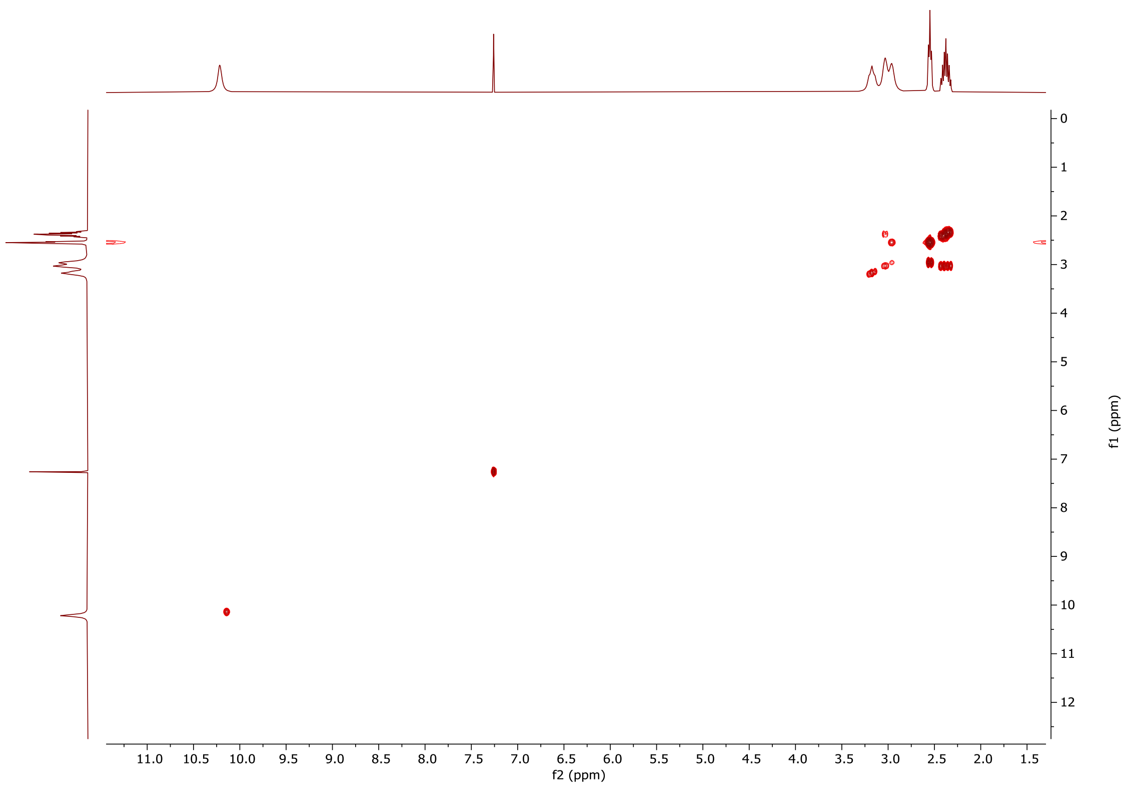
11.0 10.5 10.0 9.5 9.0 8.5 8.0 7.5 7.0 6.5 6.0 5.5 5.0 4.5 4.0 3.5 3.0 2.5 2.0 1.5
f2 (ppm)
0 1 2 3 4 5 6 7 8 9 10 11 12
f1 (ppm)

d)

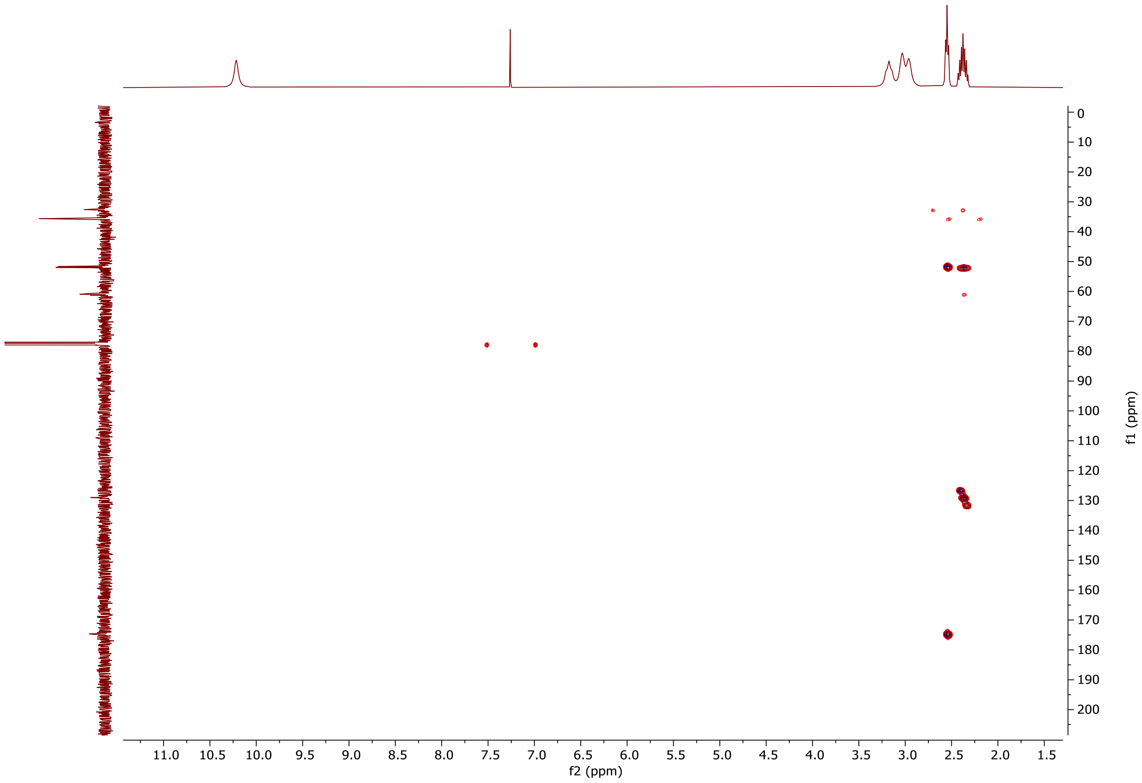

e)

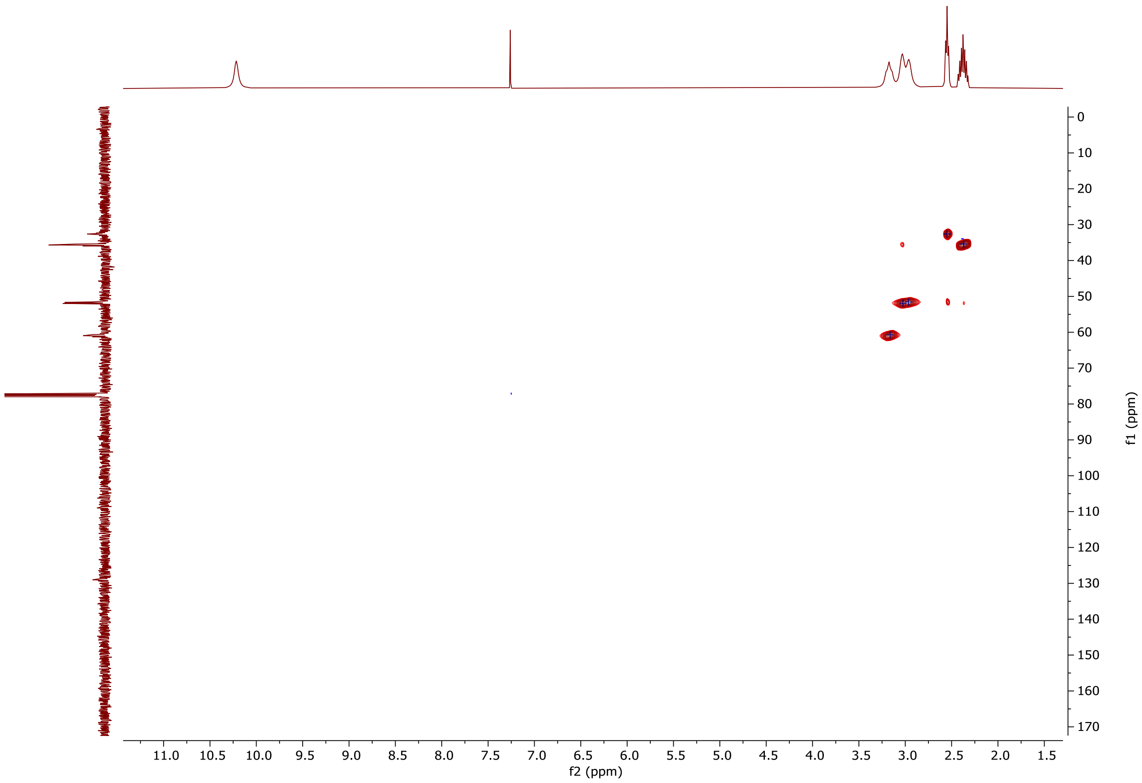

Figure S35. NMR spectra ($CDCl_3$) of **7al**: a) ^{1}H, b) ^{13}C, c) COSY, d) HMBC, e) HSQC.

a)

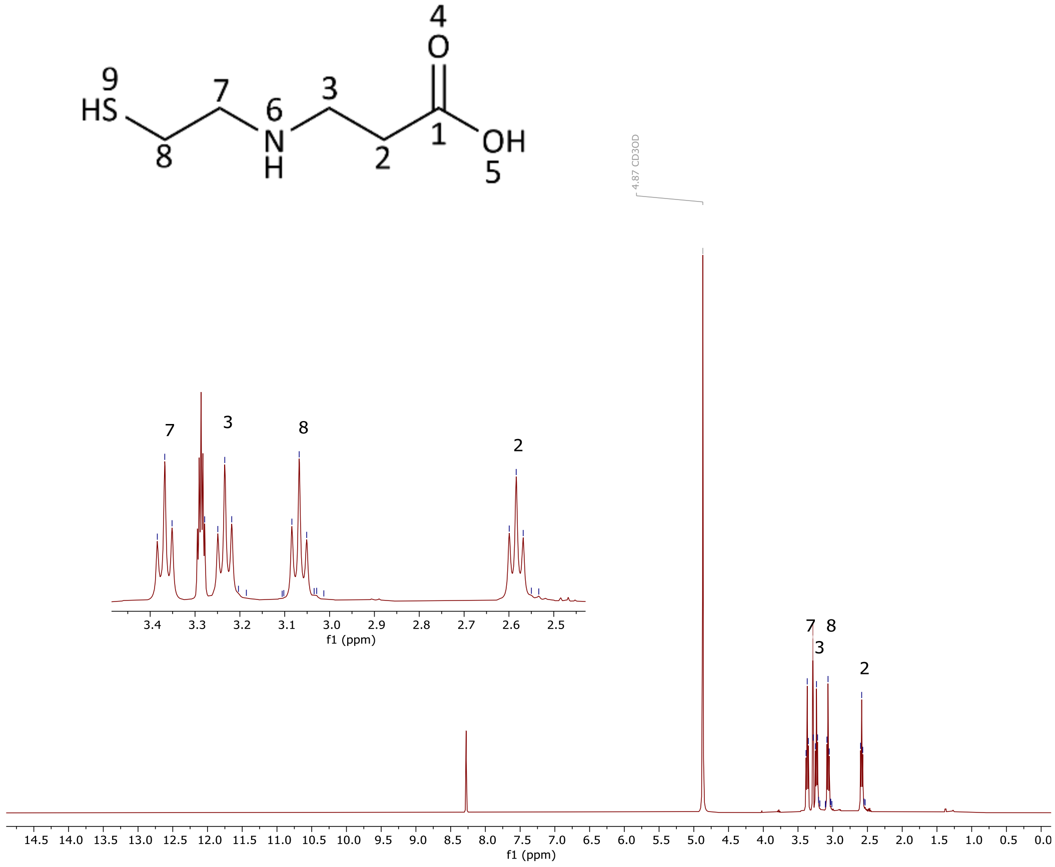

b)

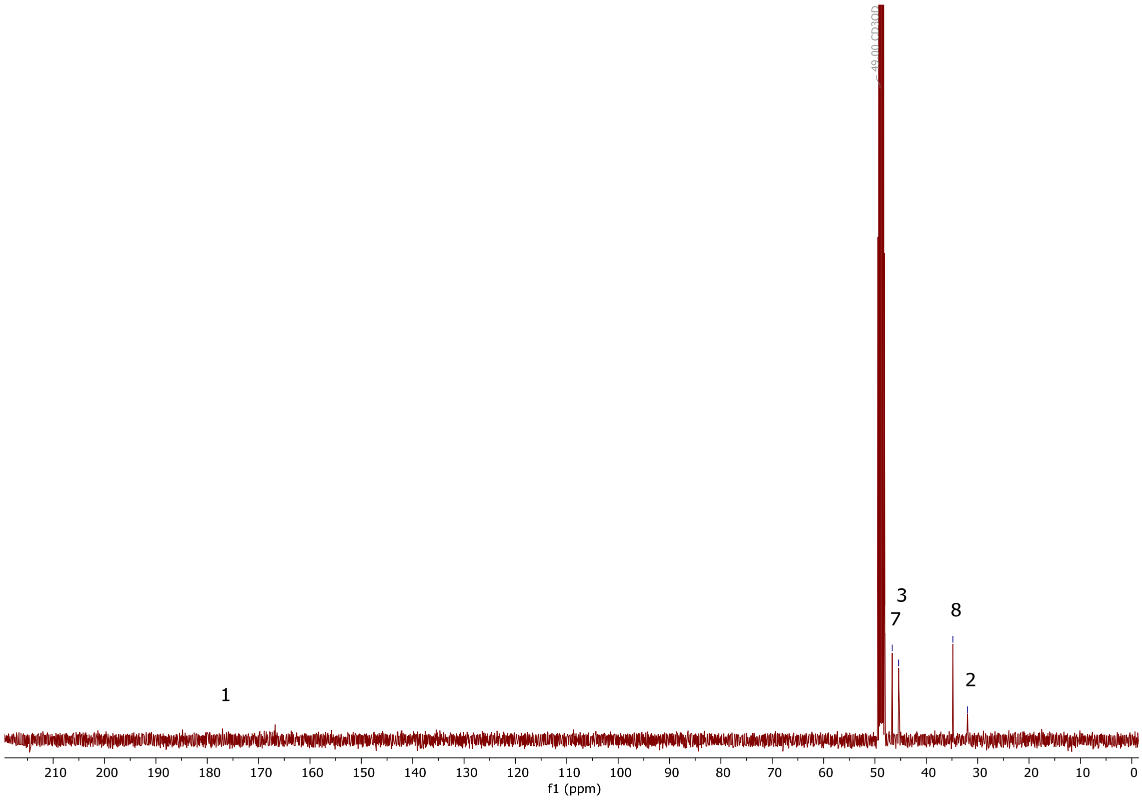

c)

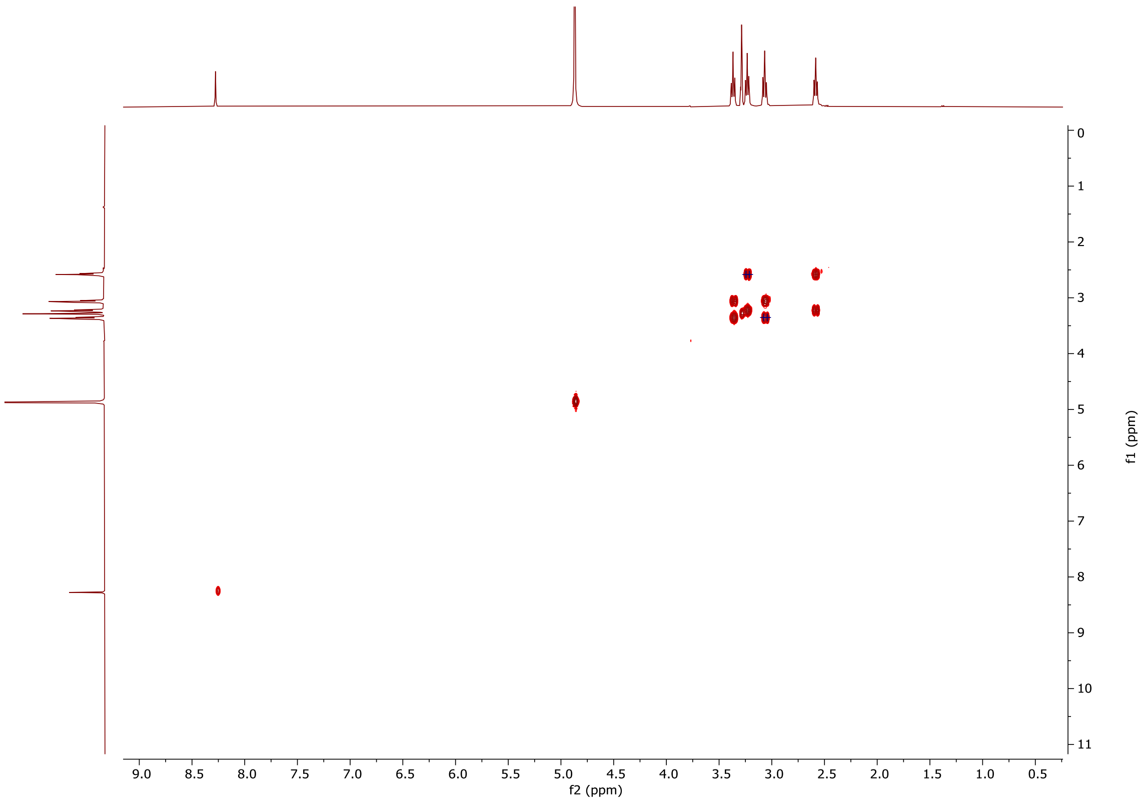

d)

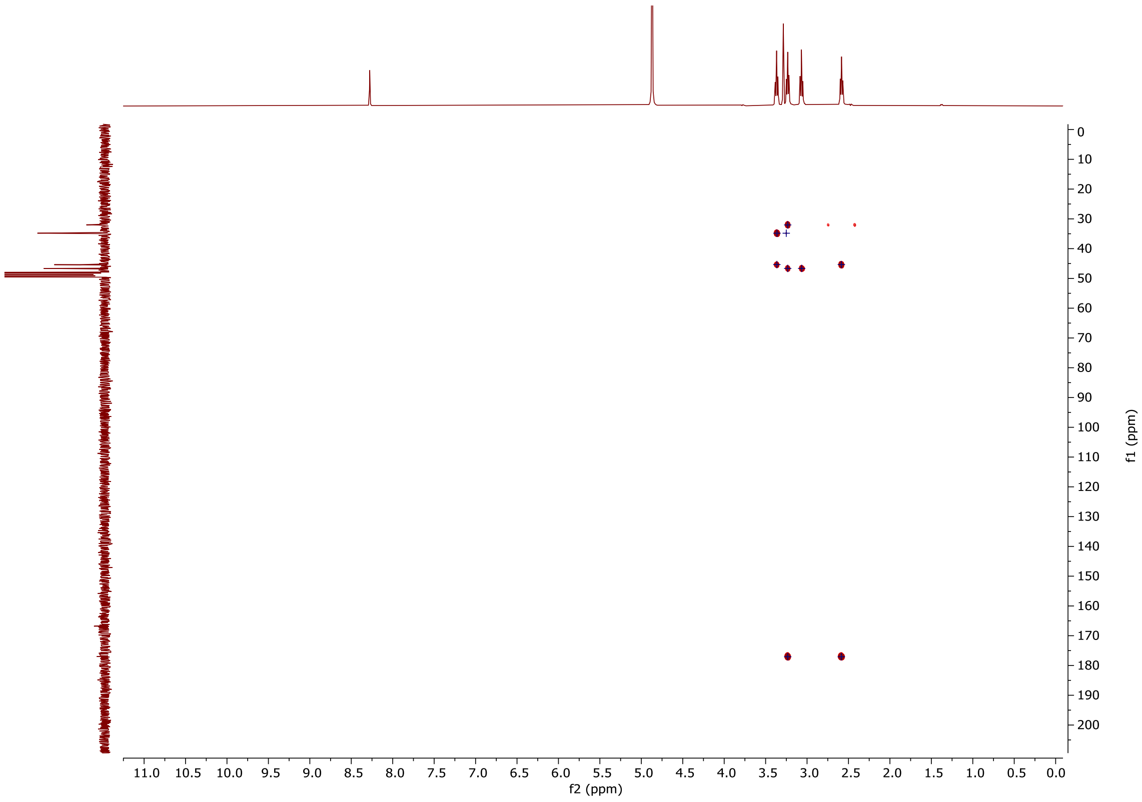

e)

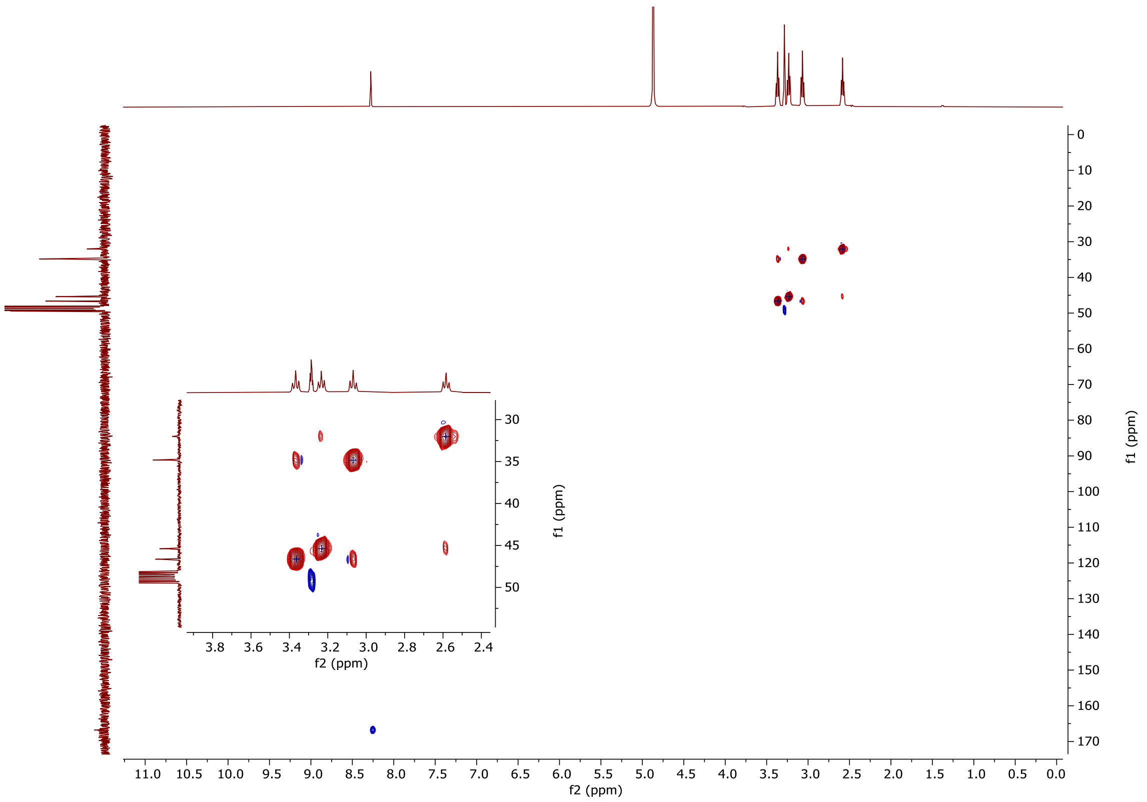

Figure S36. NMR spectra (CD_3OD) of **7am**: a) ^{1}H, b) ^{13}C, c) COSY, d) HMBC and e) HSQC.

a)

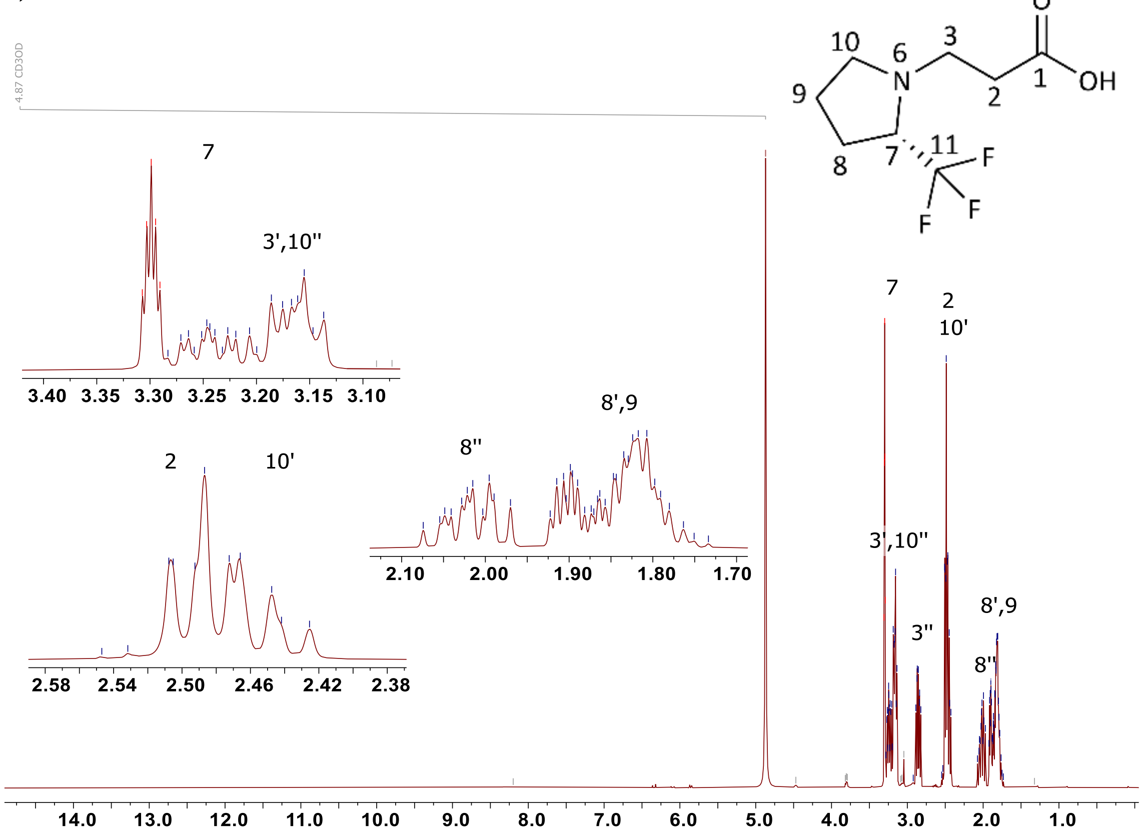

b)

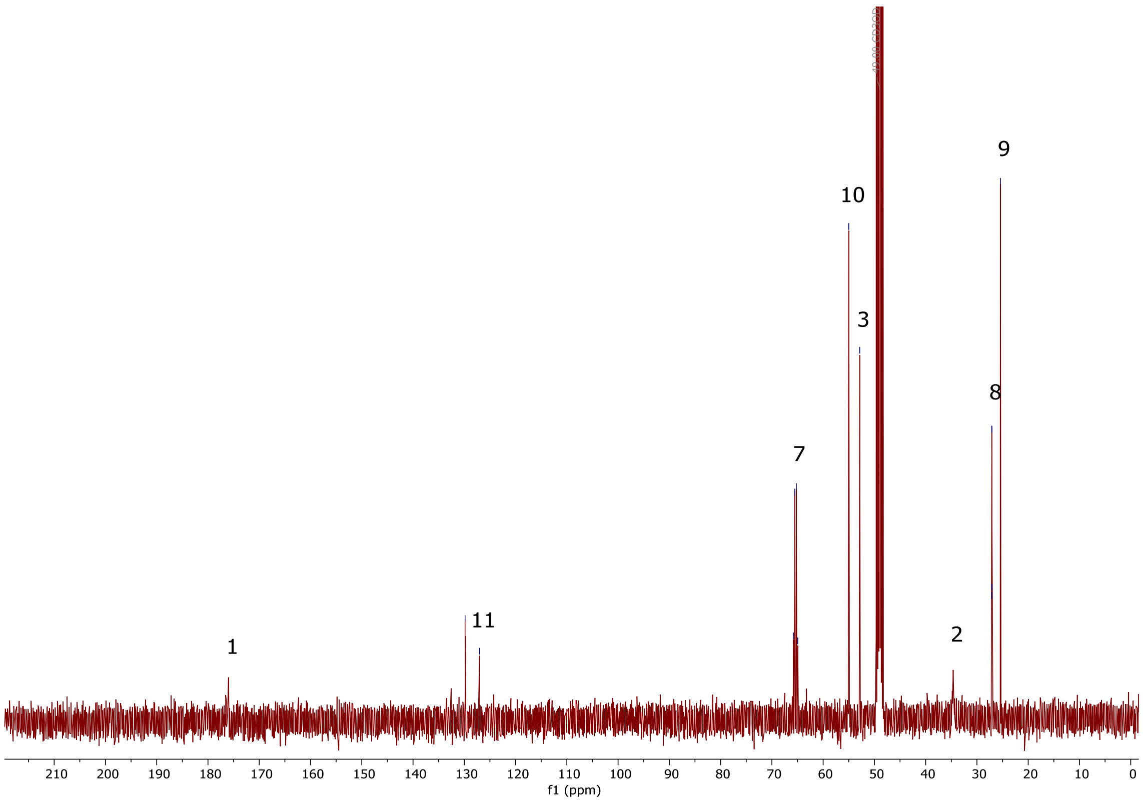

1
11
7
10
3
2
8
9
210
200
190
180
170
160
150
140
130
120
110
100
90
80
70
60
50
40
30
20
10
0
f1 (ppm)

c)

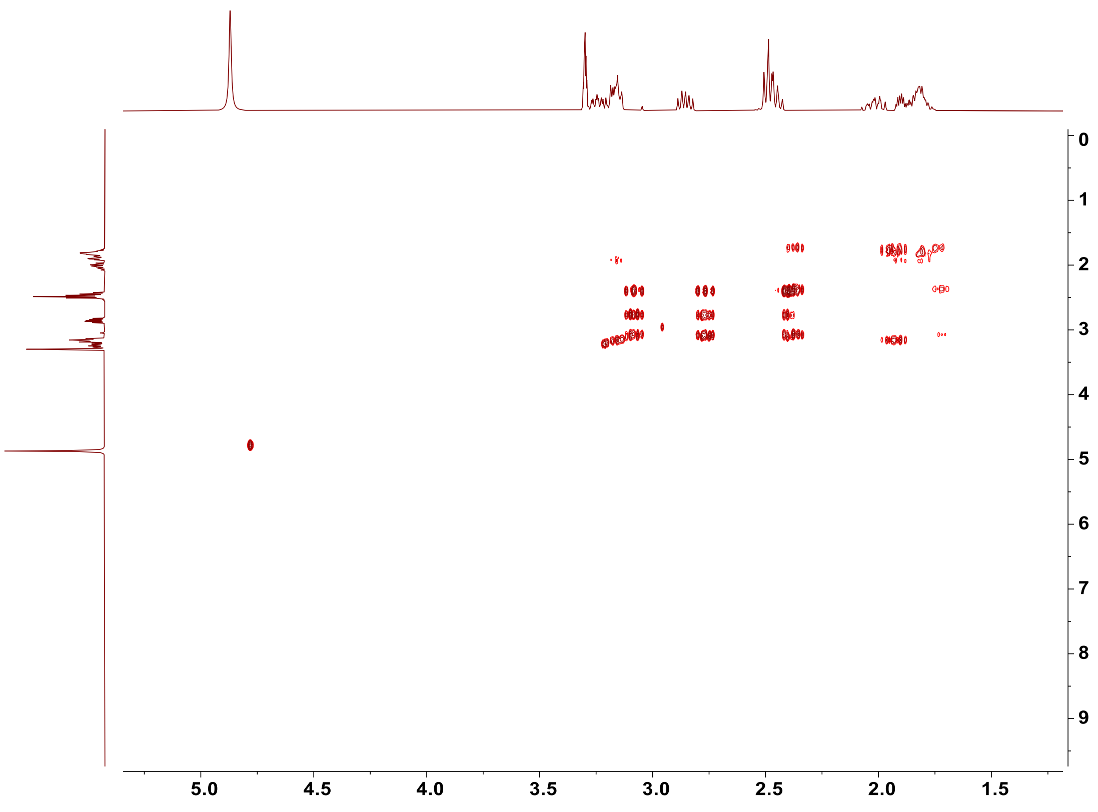

0
1
2
3
4
5
6
7
8
9
5.0
4.5
4.0
3.5
3.0
2.5
2.0
1.5

d)

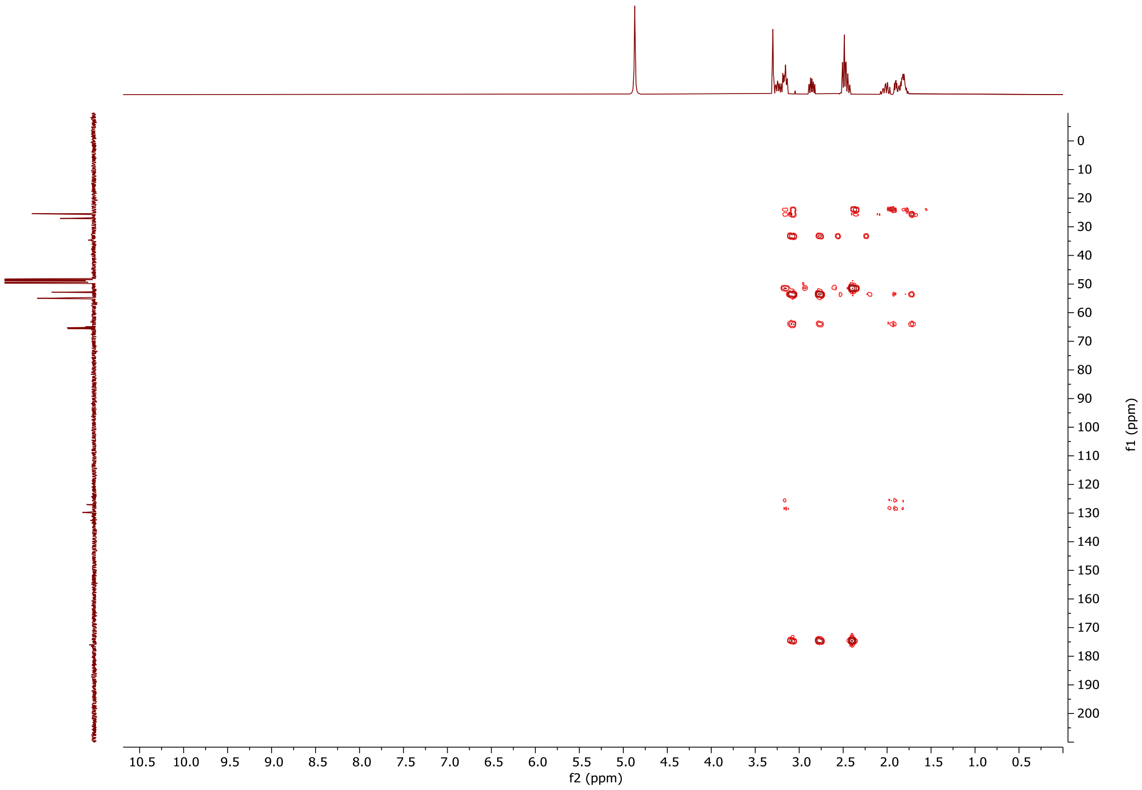

e)

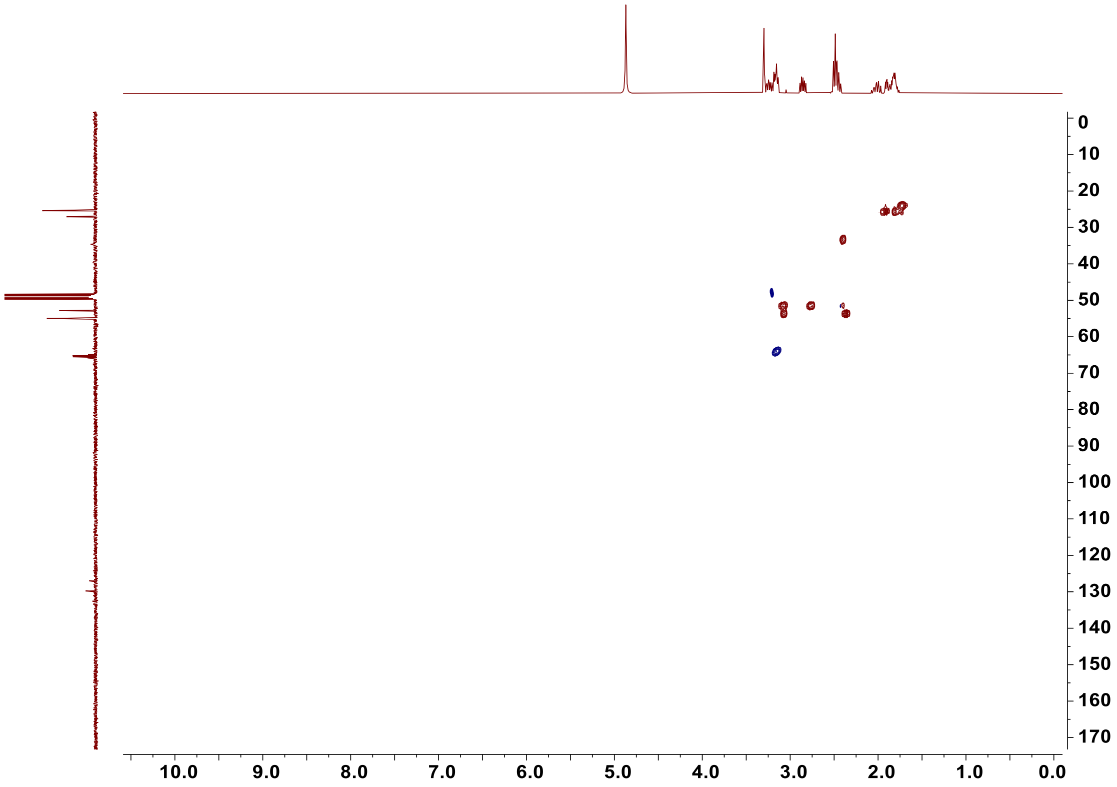

Figure S37. NMR spectra (CD_3OD) of **7an**: a) ^{1}H, b) ^{13}C, c) COSY, d) HMBC and e) HSQC.

a)

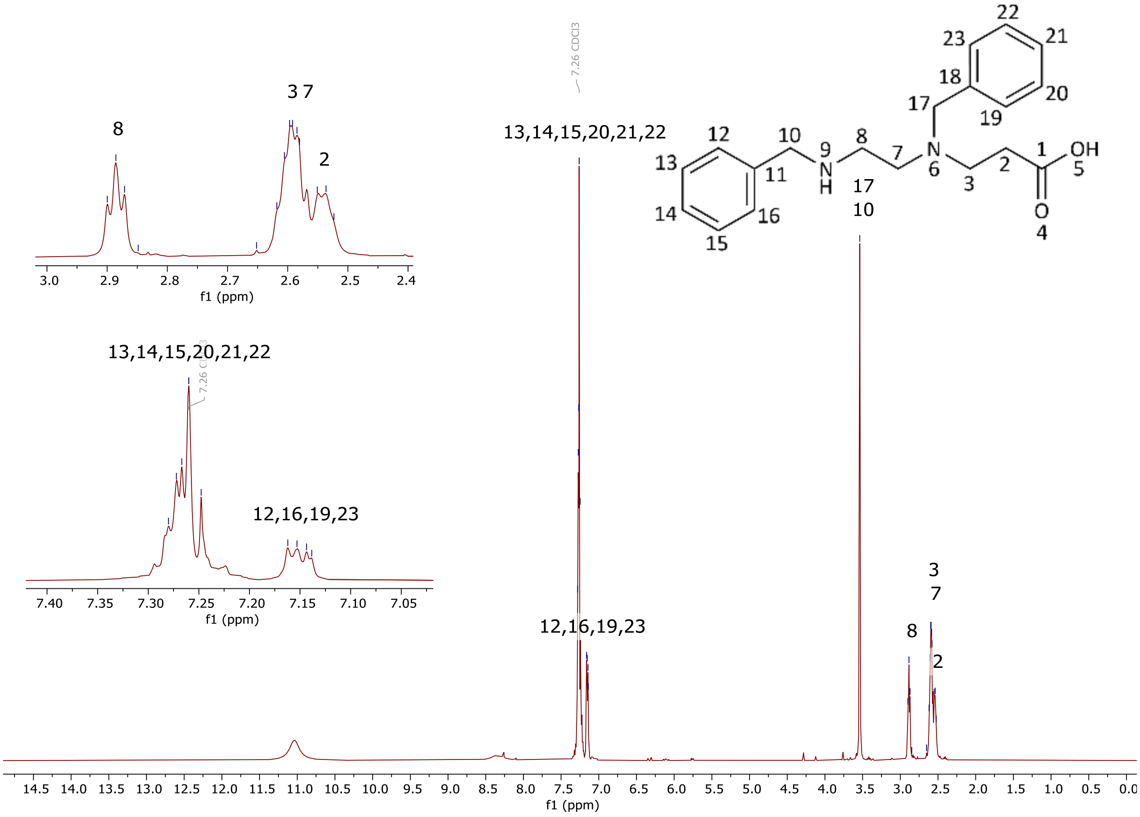

7.26 CDCl3
13,14,15,20,21,22
12,16,19,23
8
3
7
2
17
10
3 7
f1 (ppm)

b)

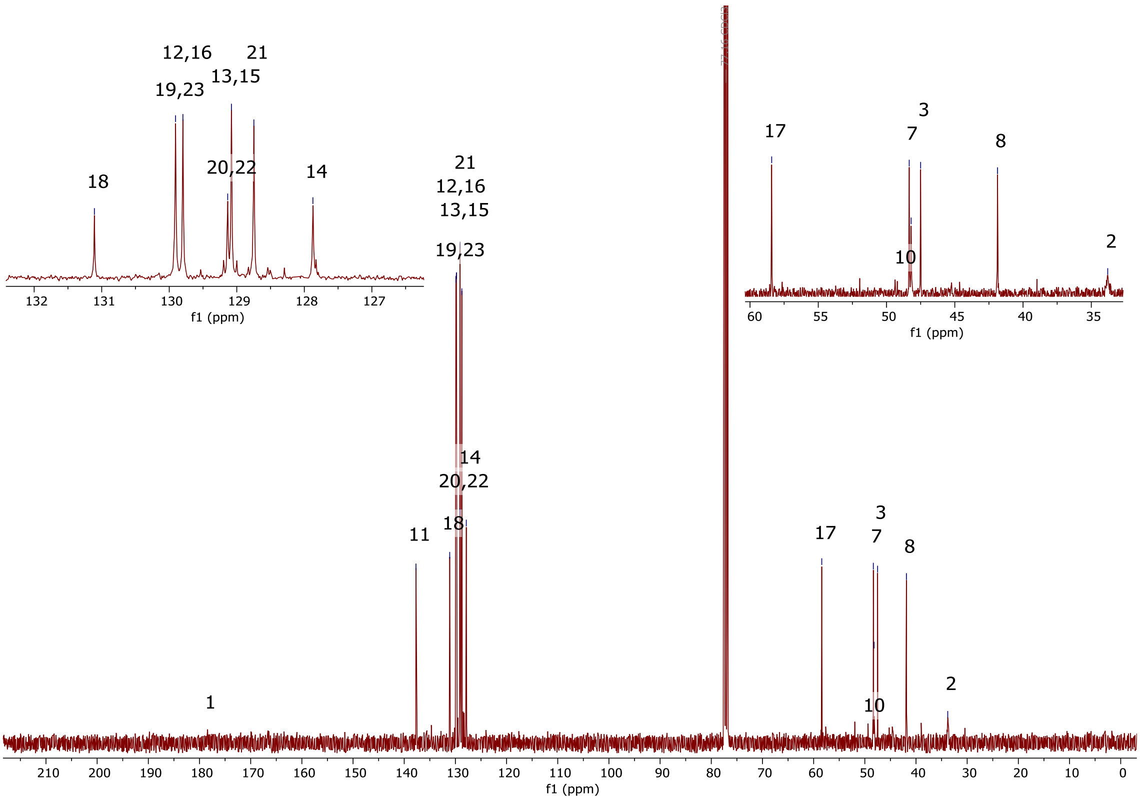

12,16
19,23
21
13,15
20,22
18
14
17
3
7
10
8
2
11
1
f1 (ppm)

c)

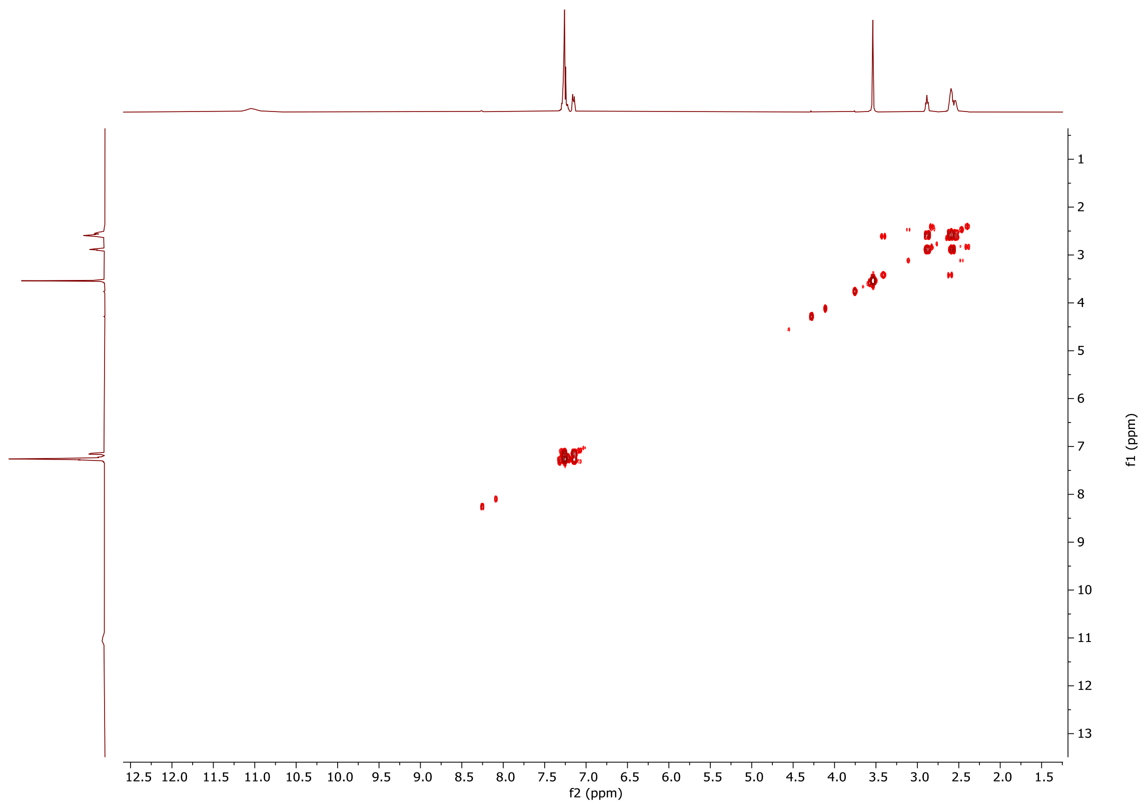

d)

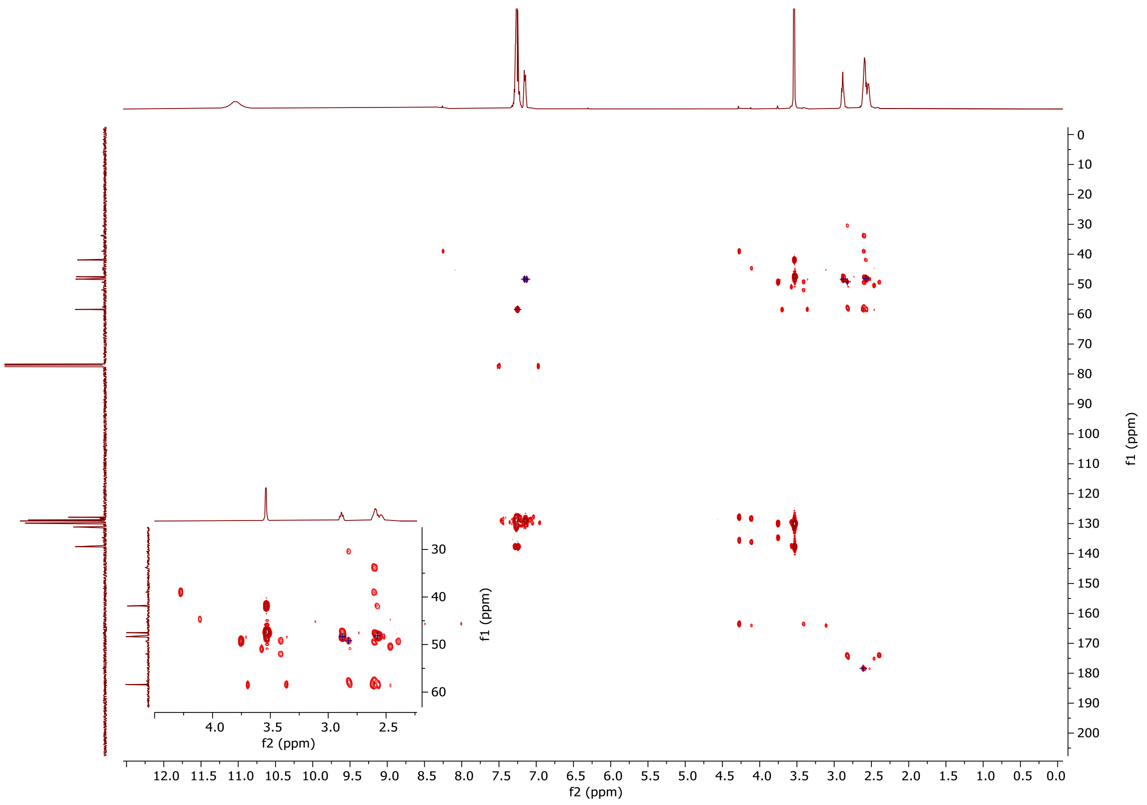

e)

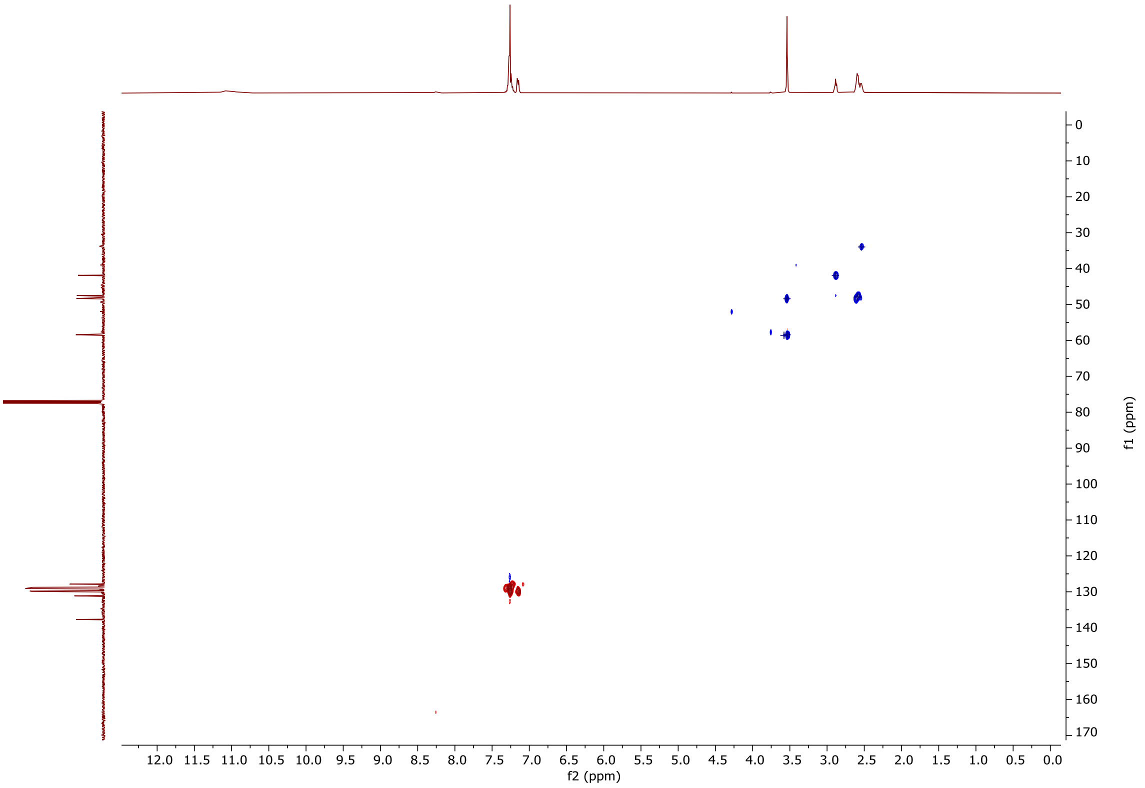

Figure S38. NMR spectra ($CDCl_3$) of **7ap**: a) ^1H, b) ^{13}C, c) COSY, d) HMBC and e) HSQC.

a)

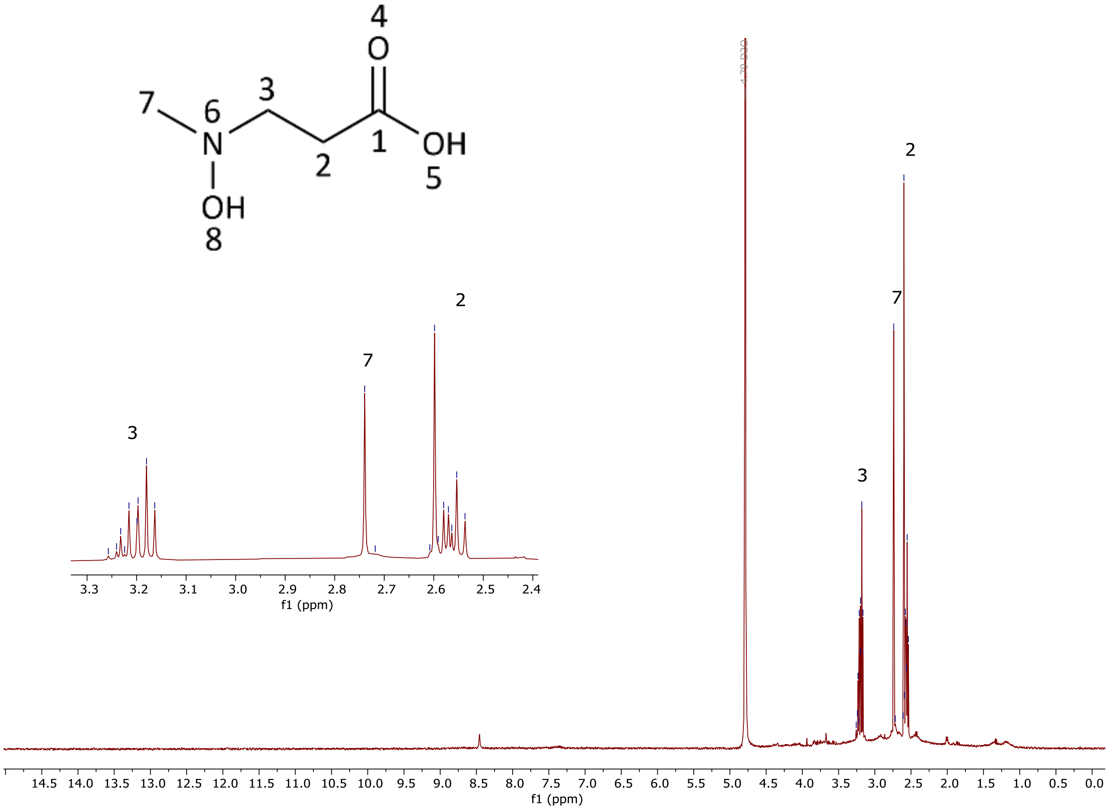

b)

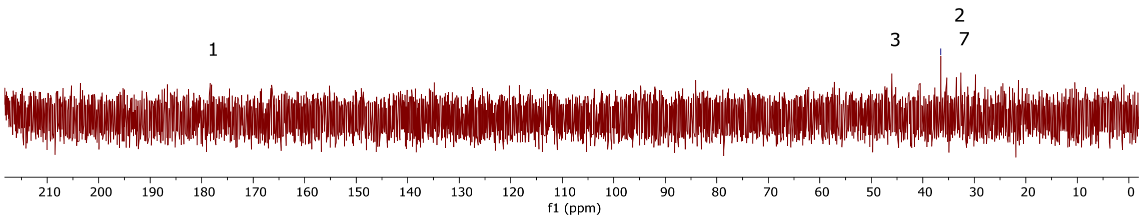
1
3
2
7
210 200 190 180 170 160 150 140 130 120 110 100 90 80 70 60 50 40 30 20 10 0
f1 (ppm)

c)

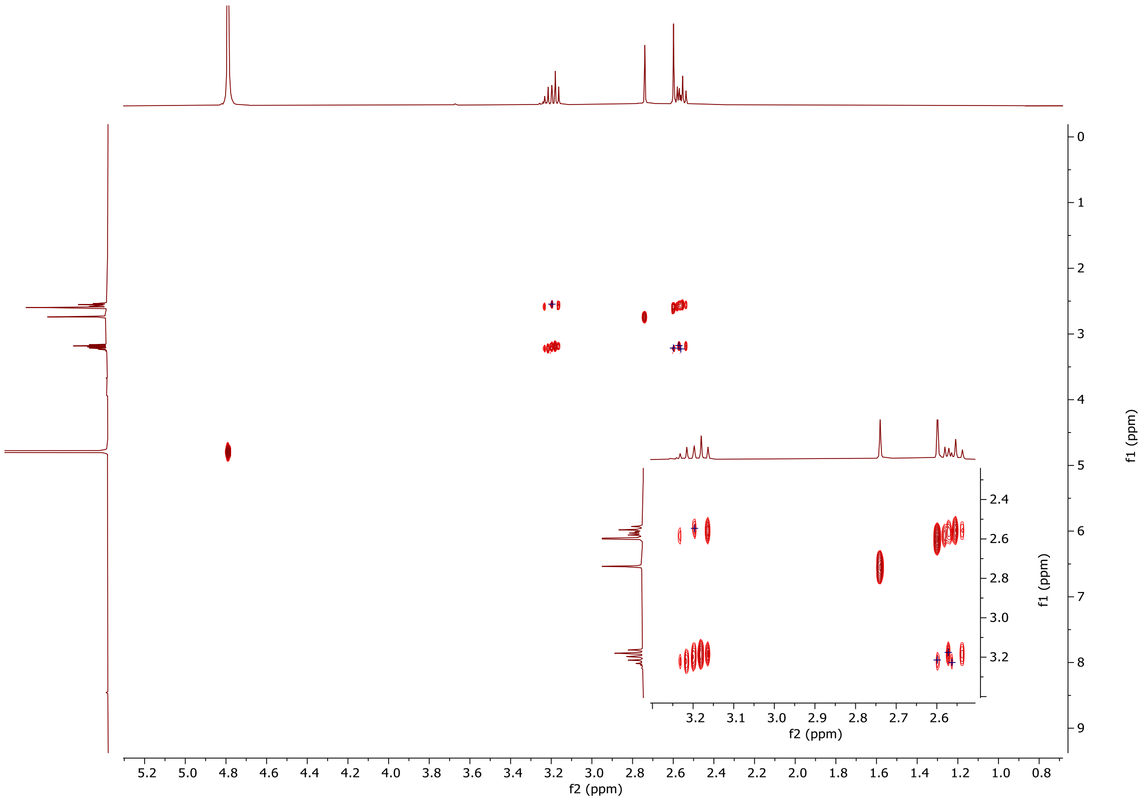
f2 (ppm)
f1 (ppm)
5.2 5.0 4.8 4.6 4.4 4.2 4.0 3.8 3.6 3.4 3.2 3.0 2.8 2.6 2.4 2.2 2.0 1.8 1.6 1.4 1.2 1.0 0.8
0 1 2 3 4 5 6 7 8 9
3.2 3.1 3.0 2.9 2.8 2.7 2.6
2.4 2.6 2.8 3.0 3.2

d)

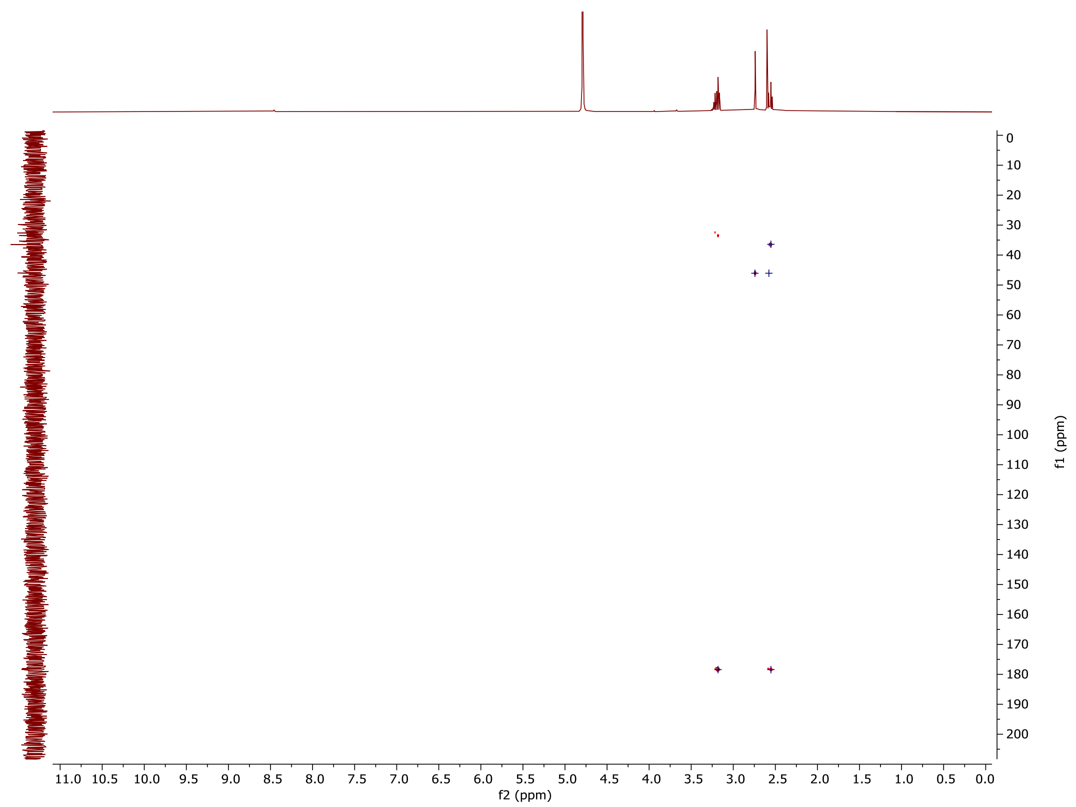

e)

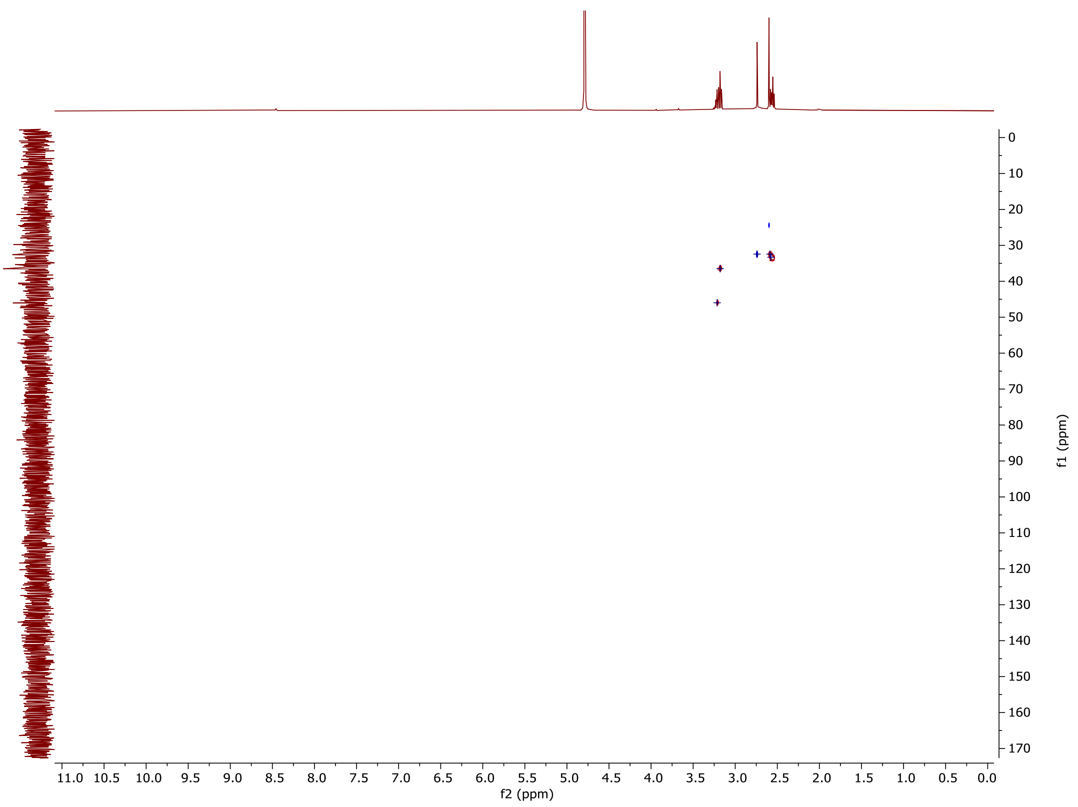

Figure S39. NMR spectra (D_2O) of **7aq**: a) ^1H, b) ^{13}C, c) COSY, d) HMBC and e) HSQC.

a)

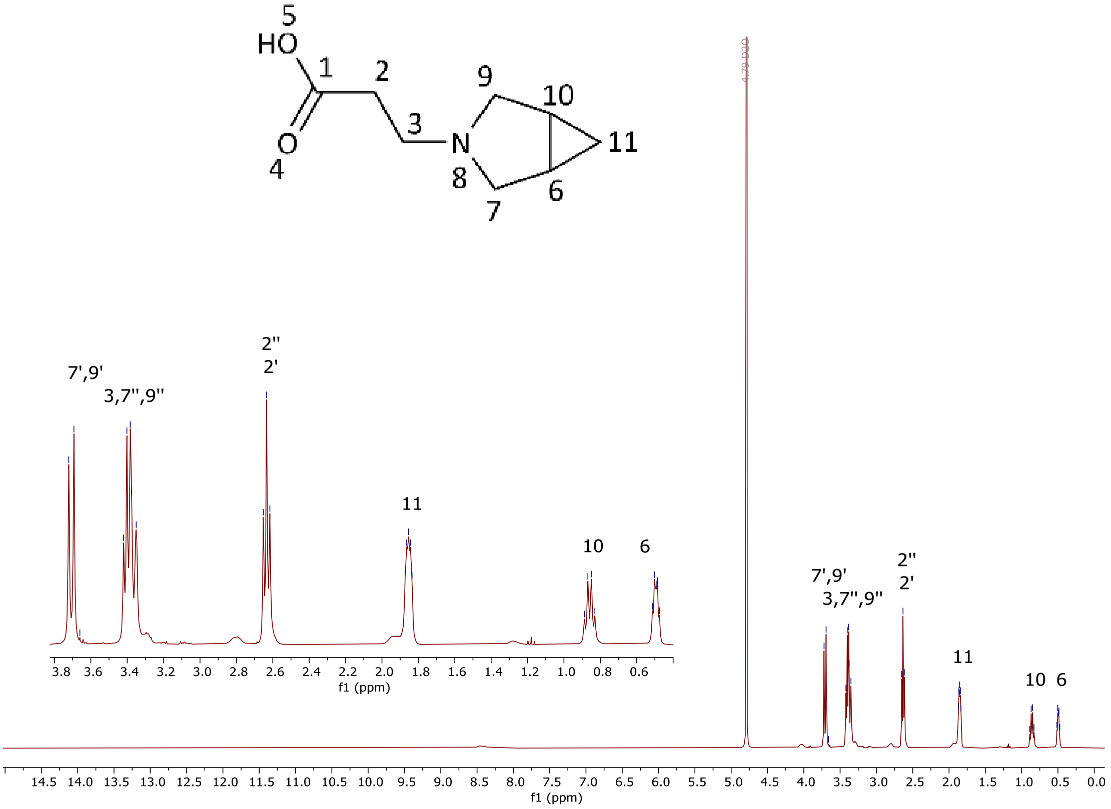

b)

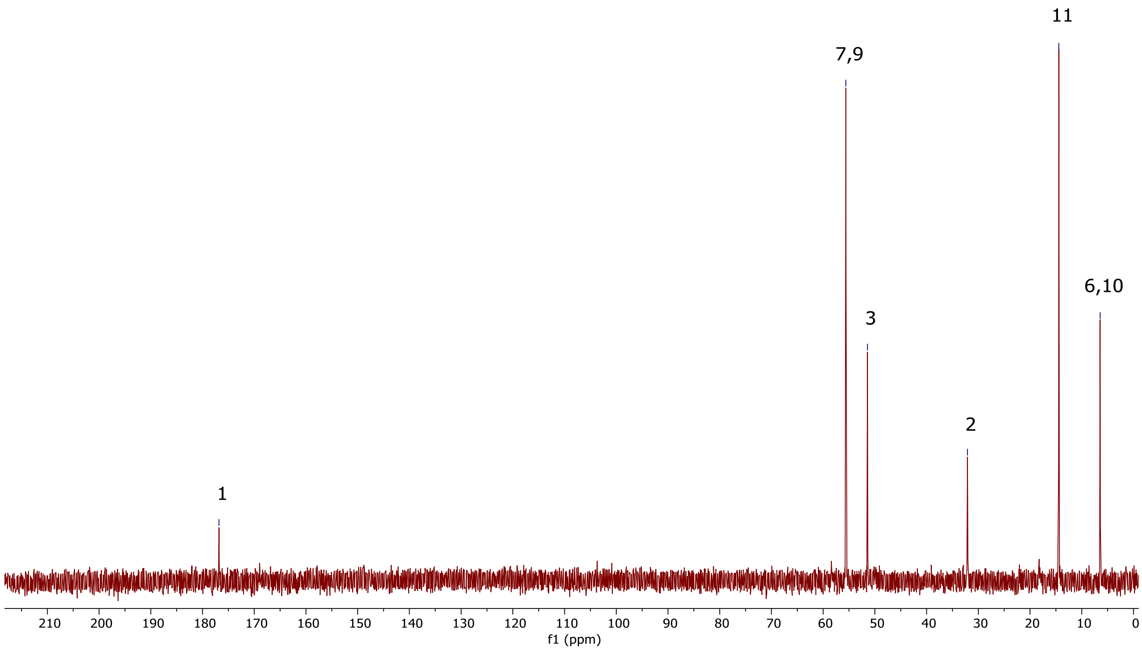

c)

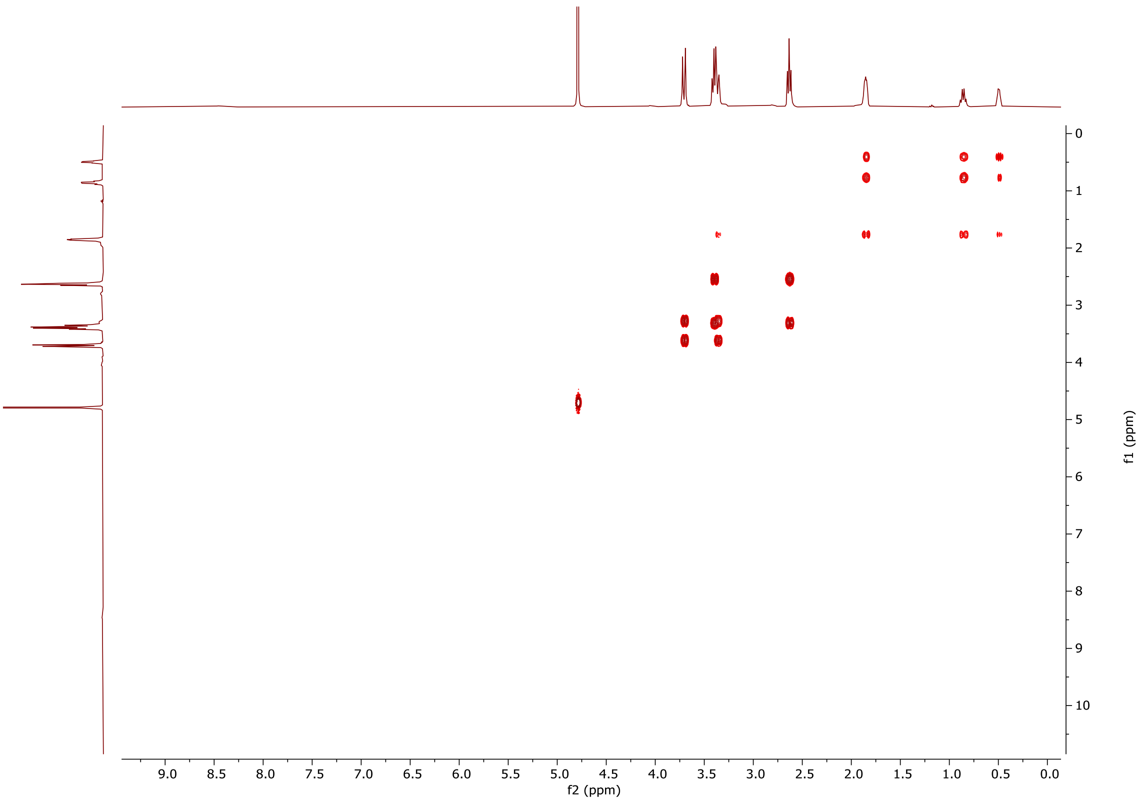

d)

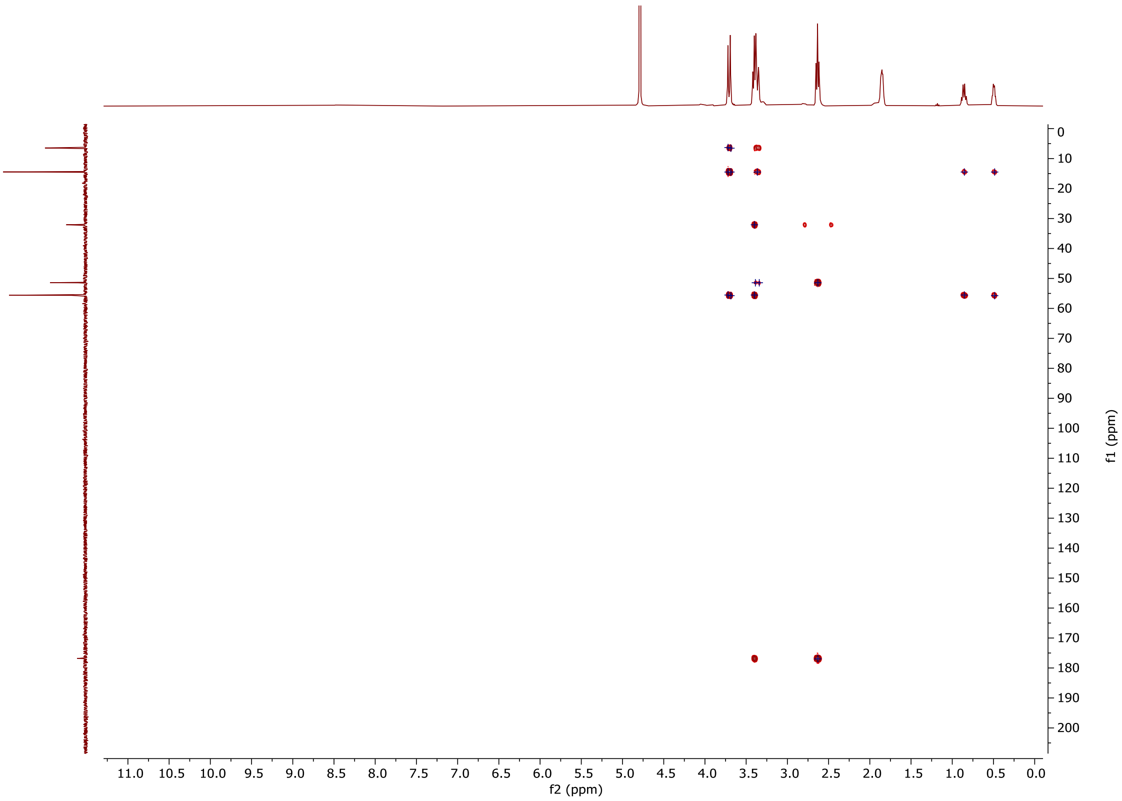

e)

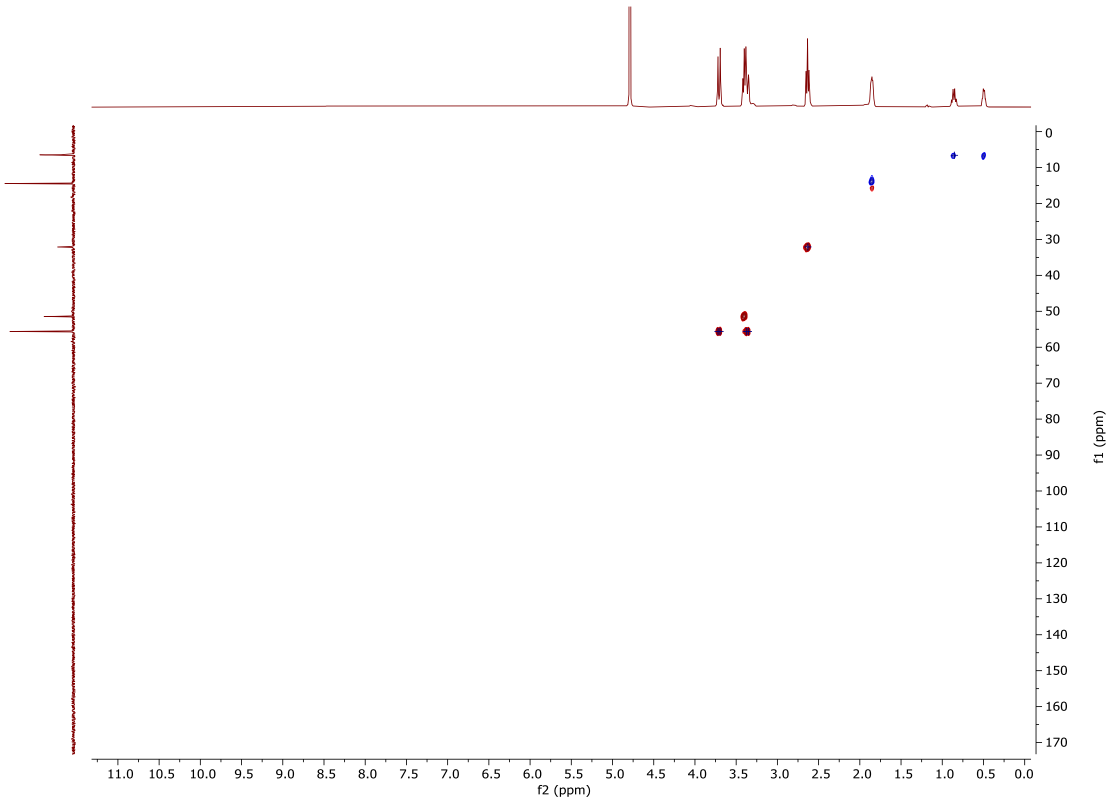

Figure S40. NMR spectra (D_2O) of **7ar**: a) ^1H, b) ^{13}C, c) COSY, d) HMBC and e) HSQC.

a)

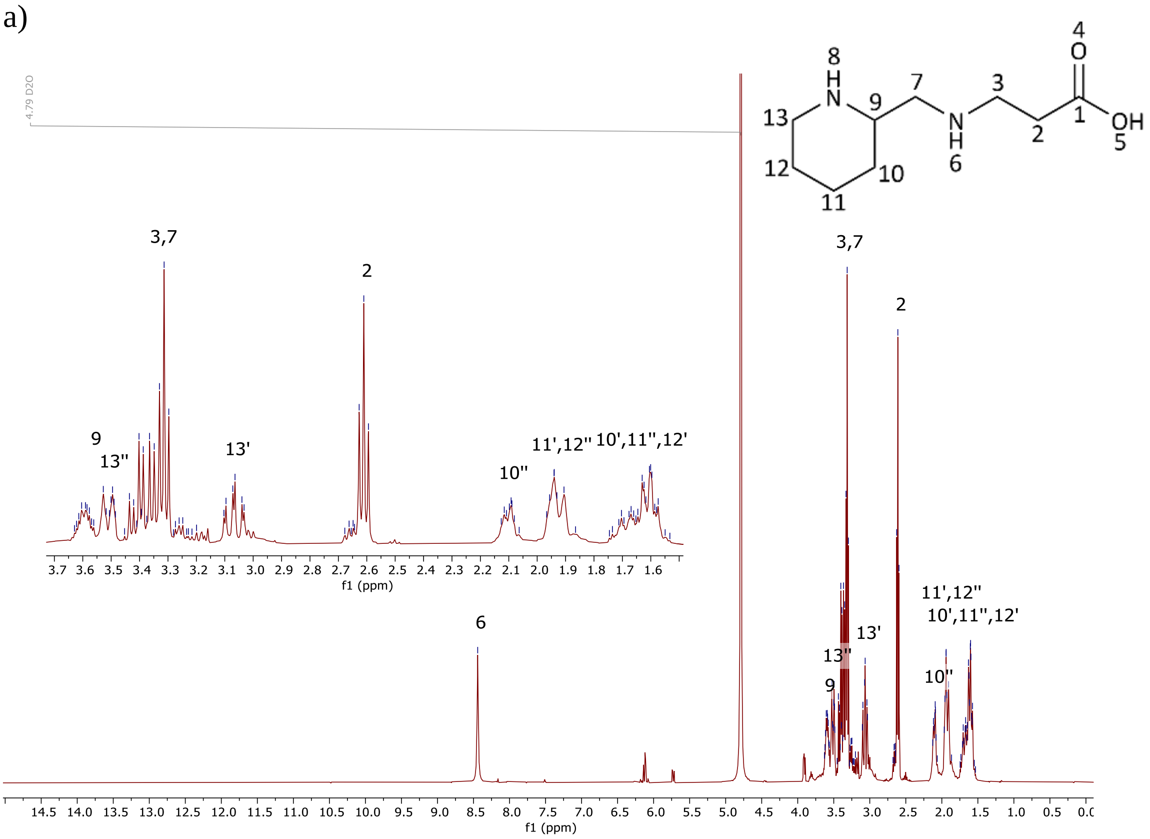

b)

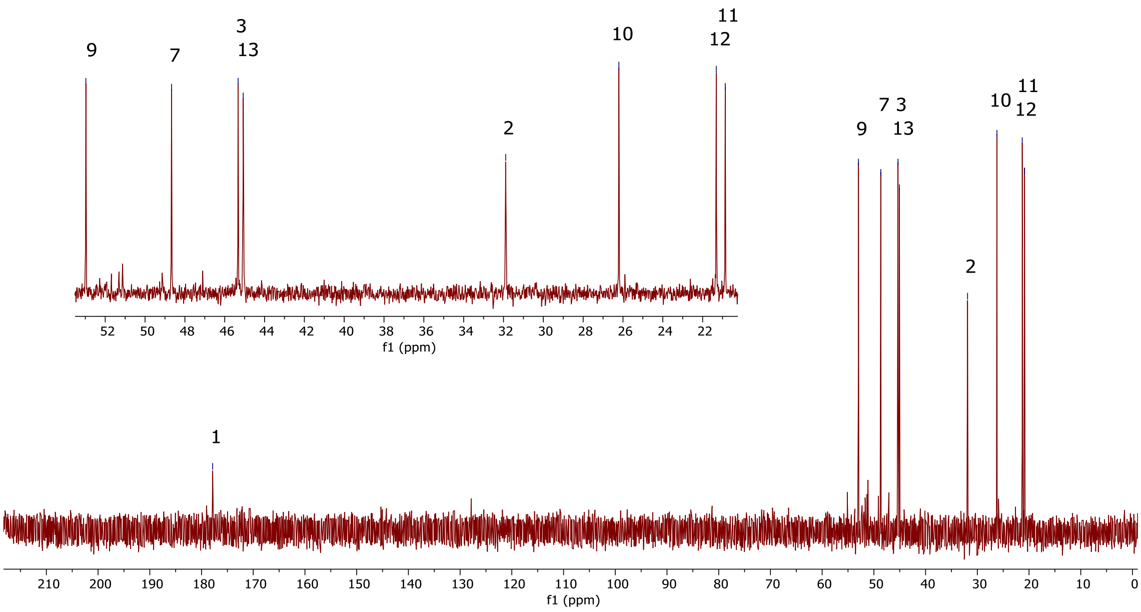

c)

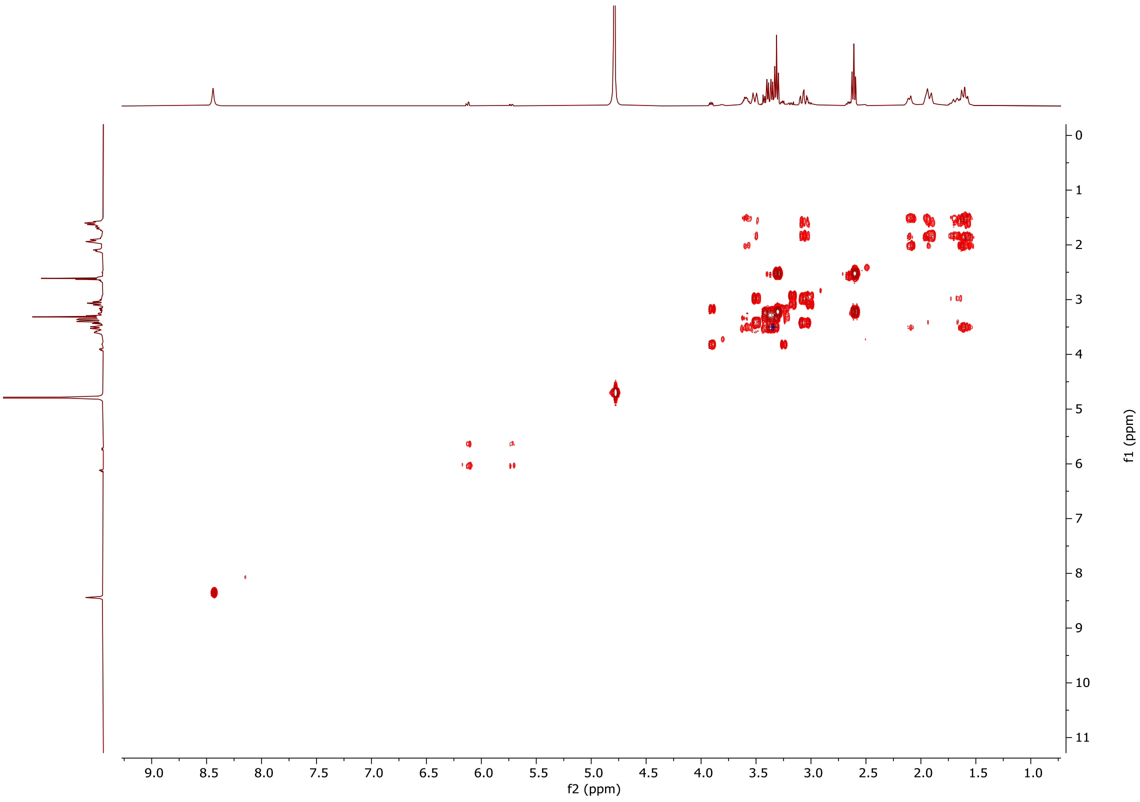

d)

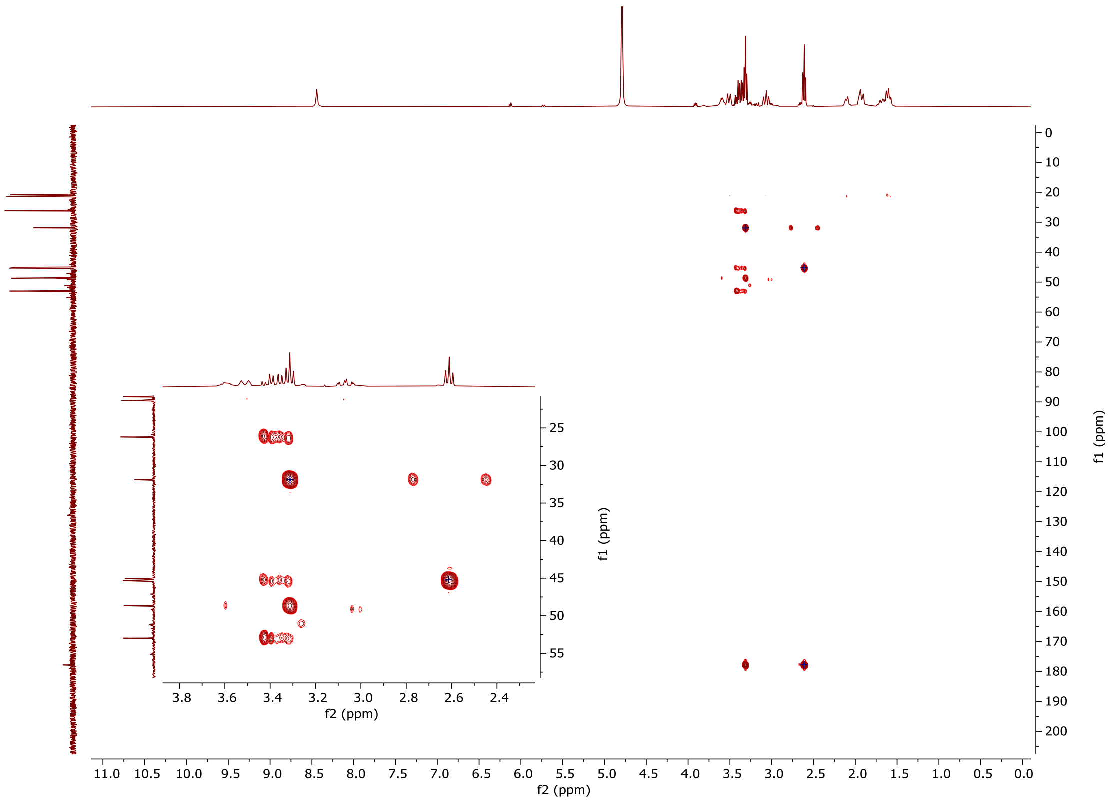

e)

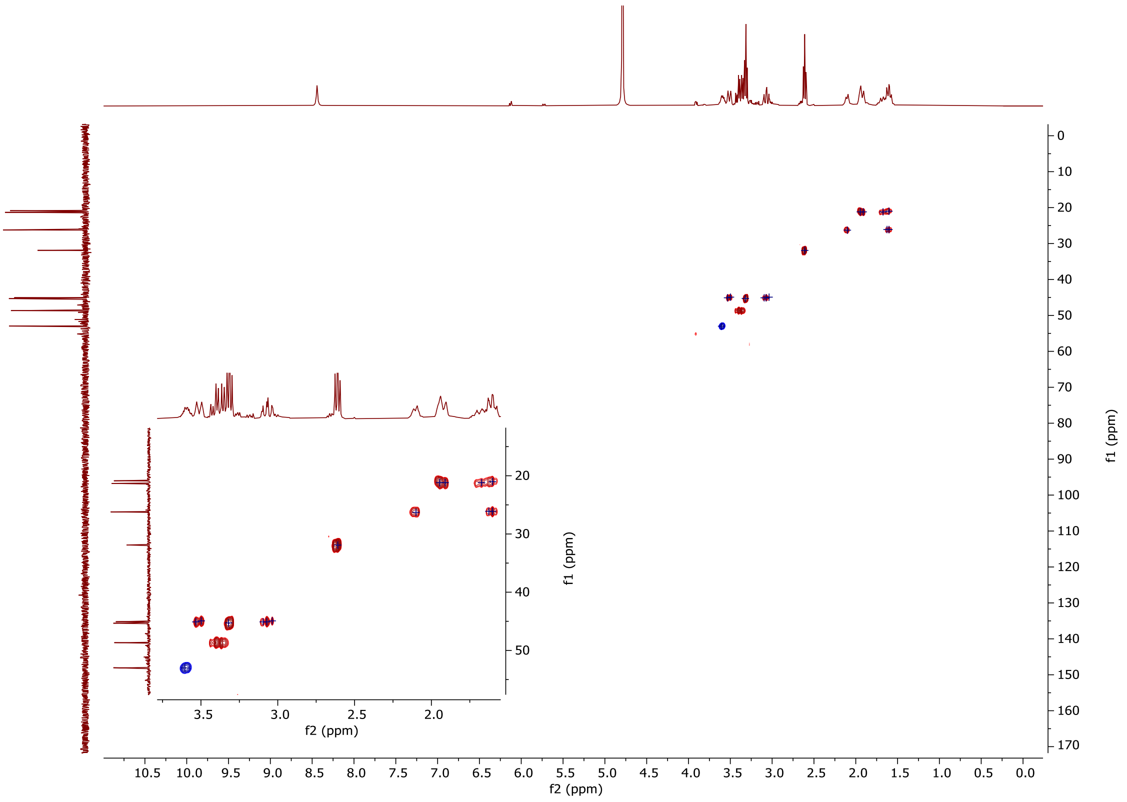

Figure S41. NMR spectra (D_2O) of **12**: a) ^{1}H, b) ^{13}C, c) COSY, d) HMBC and e) HSQC.

a)

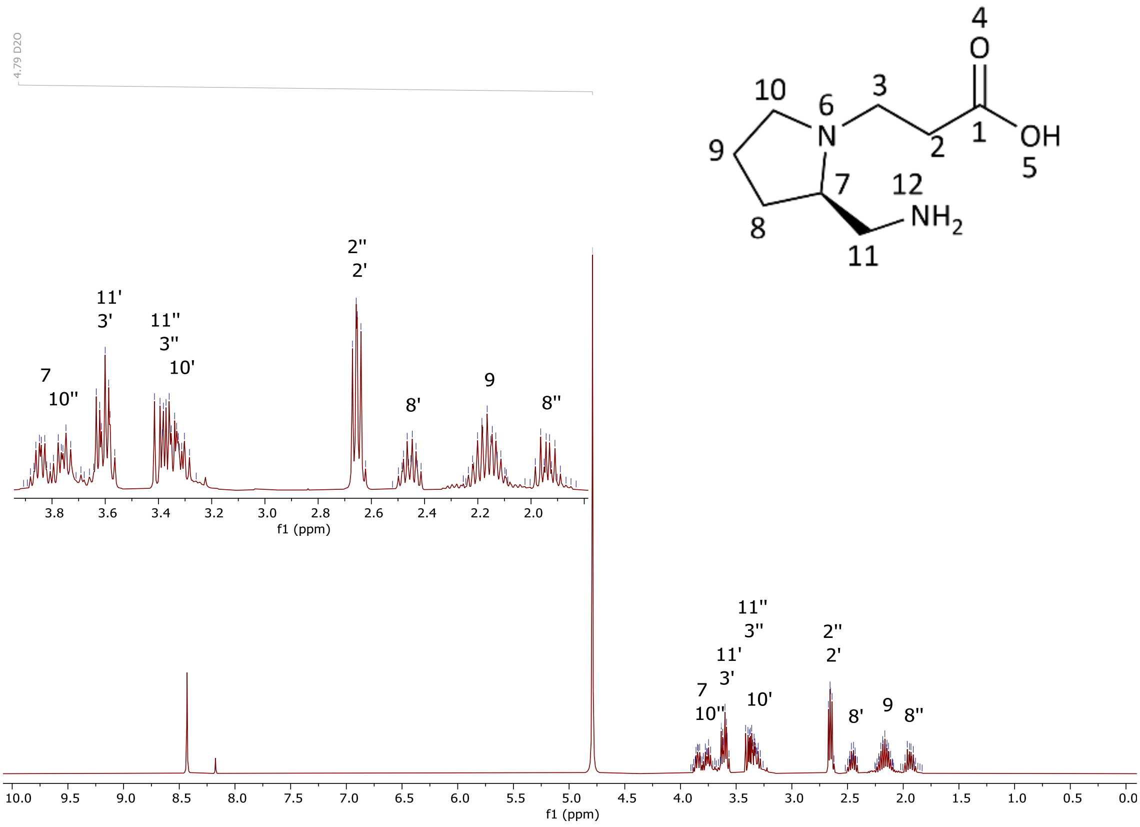
4.79 D2O
4
O
10
6
3
N
1
OH
9
2
5
7
12
NH_2
8
11
2''
2'
11'
3'
11''
3''
10'
7
10''
9
8'
8''
f1 (ppm)

b)

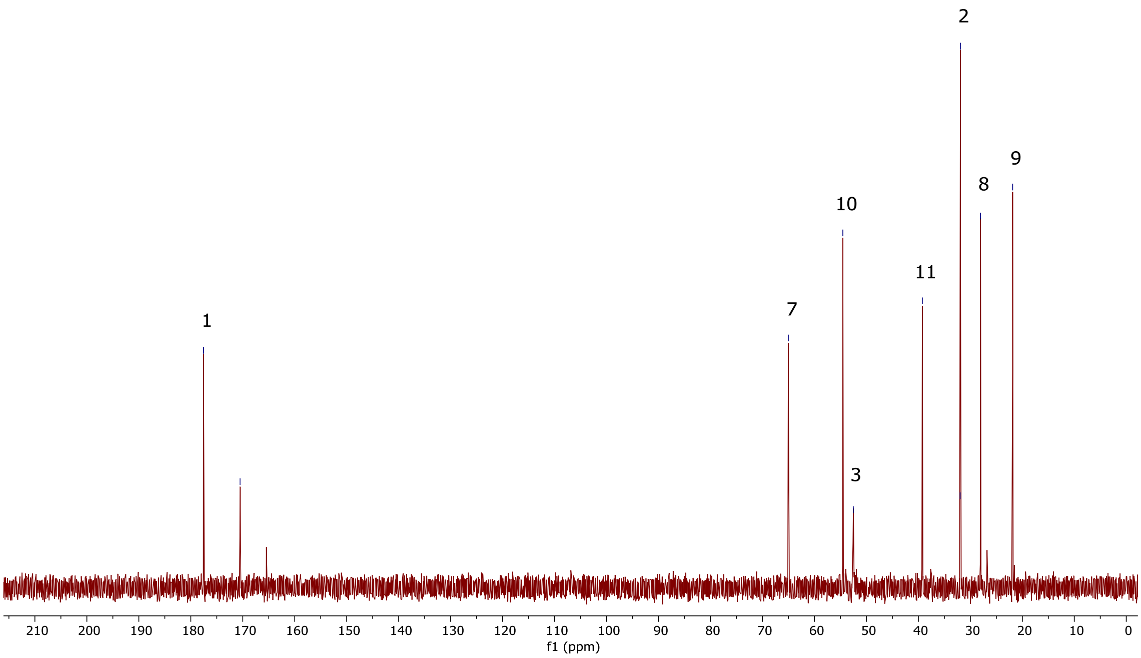
2
9
8
10
11
7
1
3
f1 (ppm)

c)

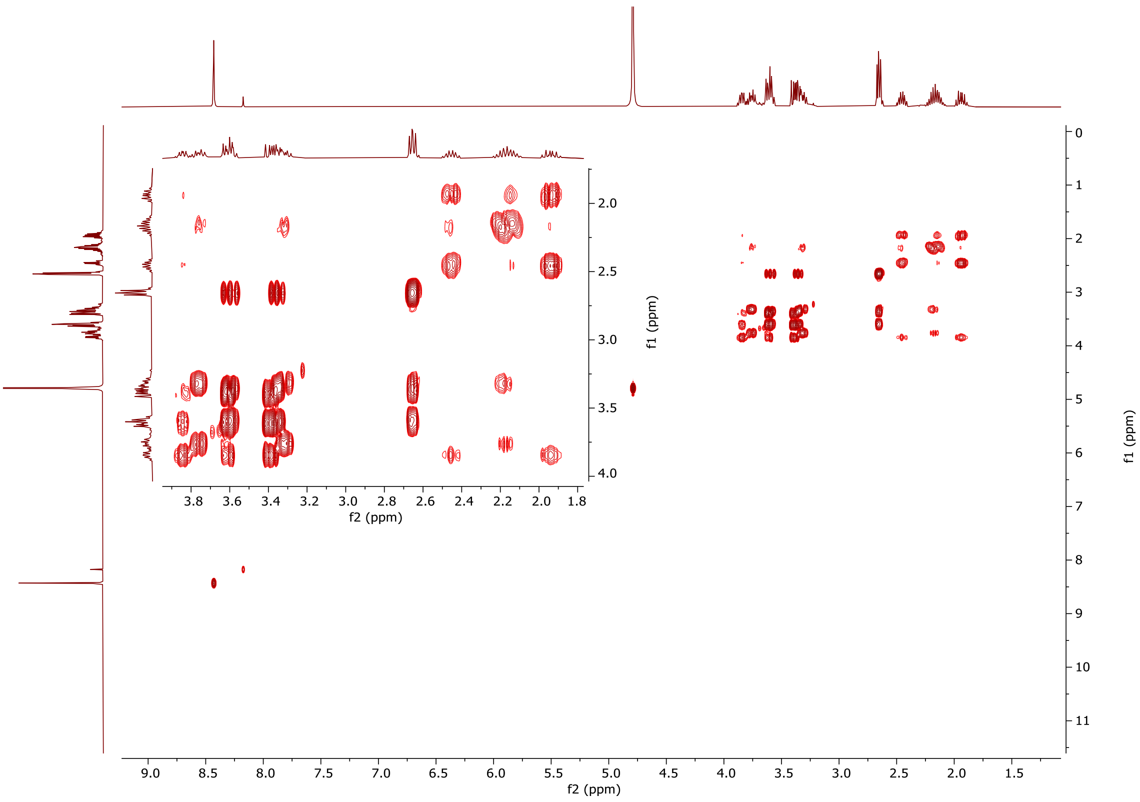

f2 (ppm)
f1 (ppm)
9.0 8.5 8.0 7.5 7.0 6.5 6.0 5.5 5.0 4.5 4.0 3.5 3.0 2.5 2.0 1.5
0 1 2 3 4 5 6 7 8 9 10 11
3.8 3.6 3.4 3.2 3.0 2.8 2.6 2.4 2.2 2.0 1.8
2.0 2.5 3.0 3.5 4.0

d)

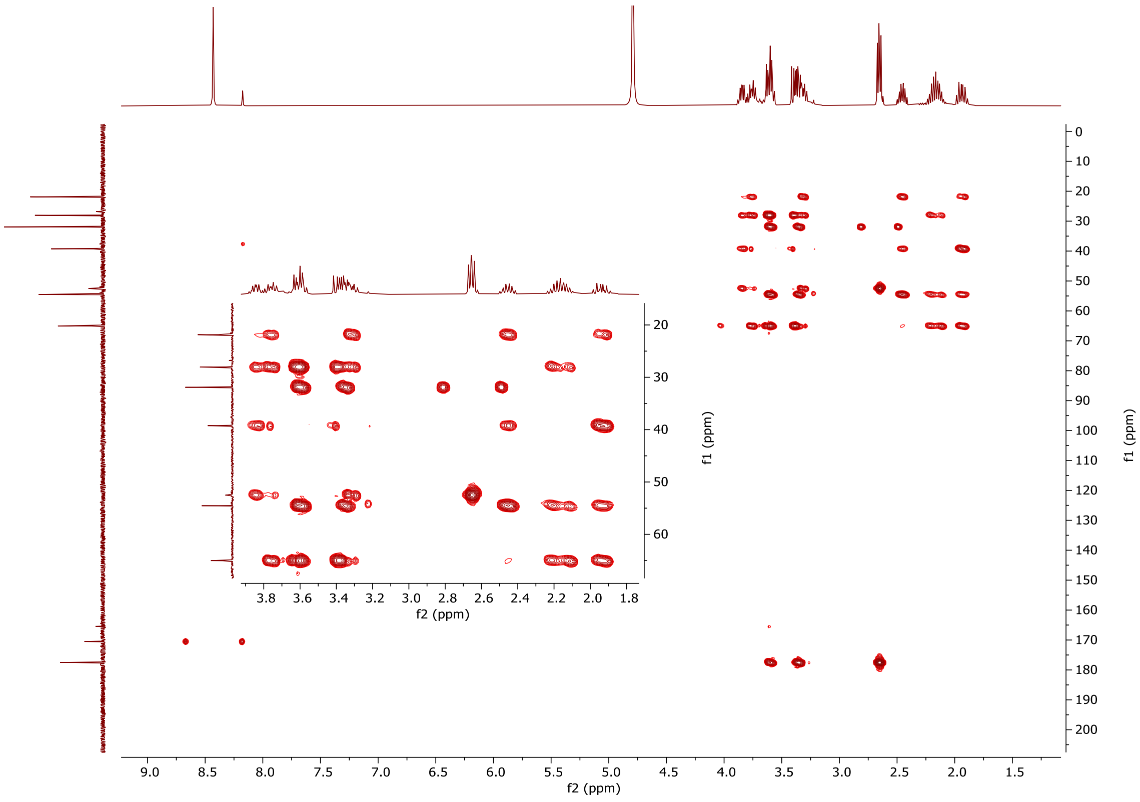

f2 (ppm)
f1 (ppm)
9.0 8.5 8.0 7.5 7.0 6.5 6.0 5.5 5.0 4.5 4.0 3.5 3.0 2.5 2.0 1.5
0 10 20 30 40 50 60 70 80 90 100 110 120 130 140 150 160 170 180 190 200
3.8 3.6 3.4 3.2 3.0 2.8 2.6 2.4 2.2 2.0 1.8
20 30 40 50 60

e)

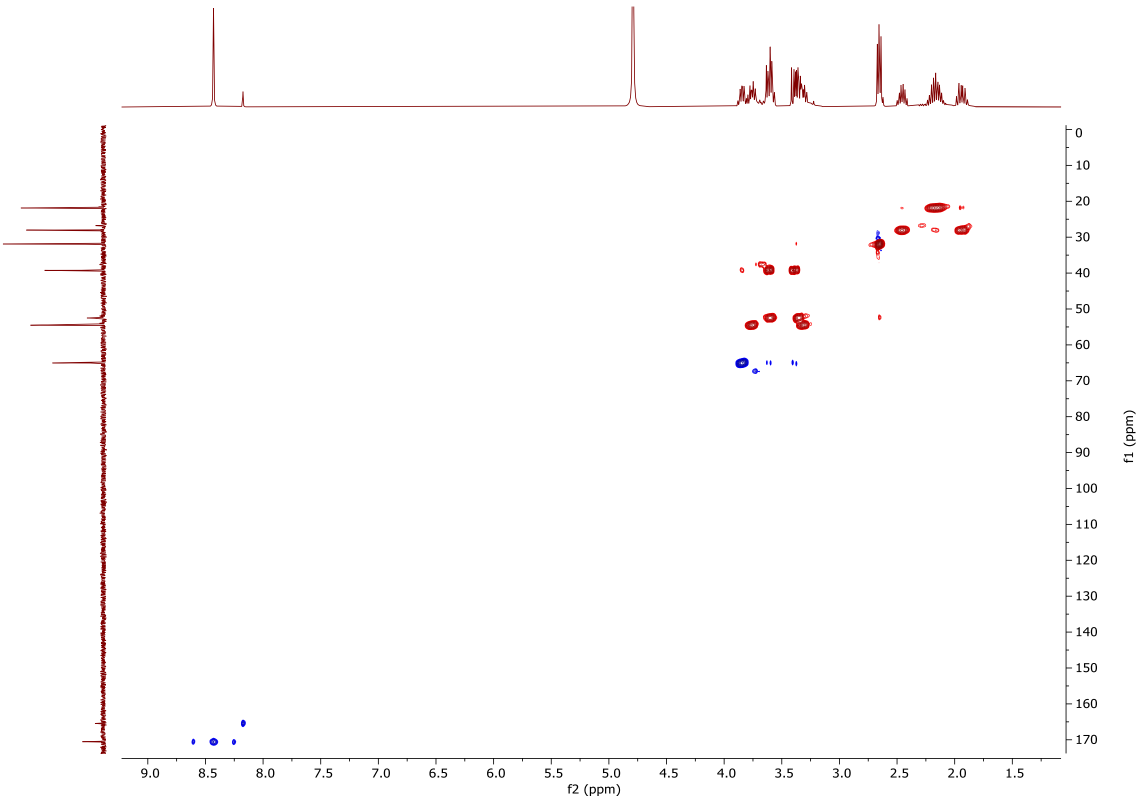

Figure S42. NMR spectra (D_2O) of (*R*)-**10**: a) ^{1}H, b) ^{13}C, c) COSY, d) HMBC and e) HSQC.

a)

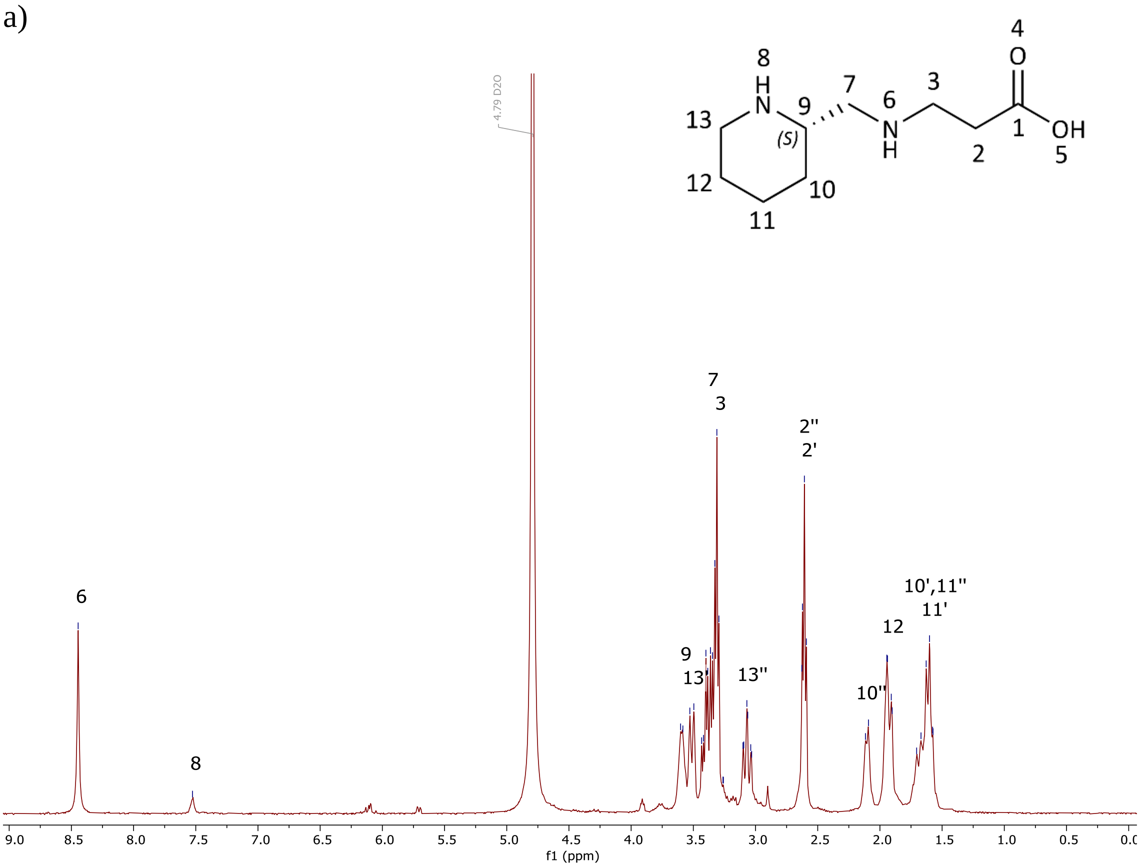

b)

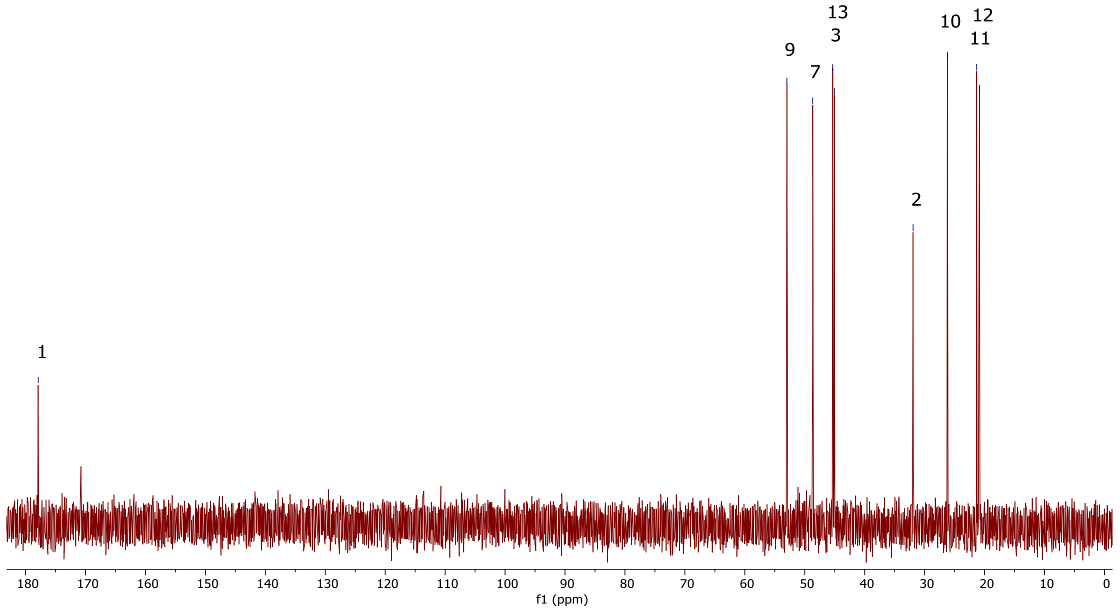

1
9
7
13
3
2
10
12
11
180
170
160
150
140
130
120
110
100
90
80
70
60
50
40
30
20
10
0
f1 (ppm)

c)

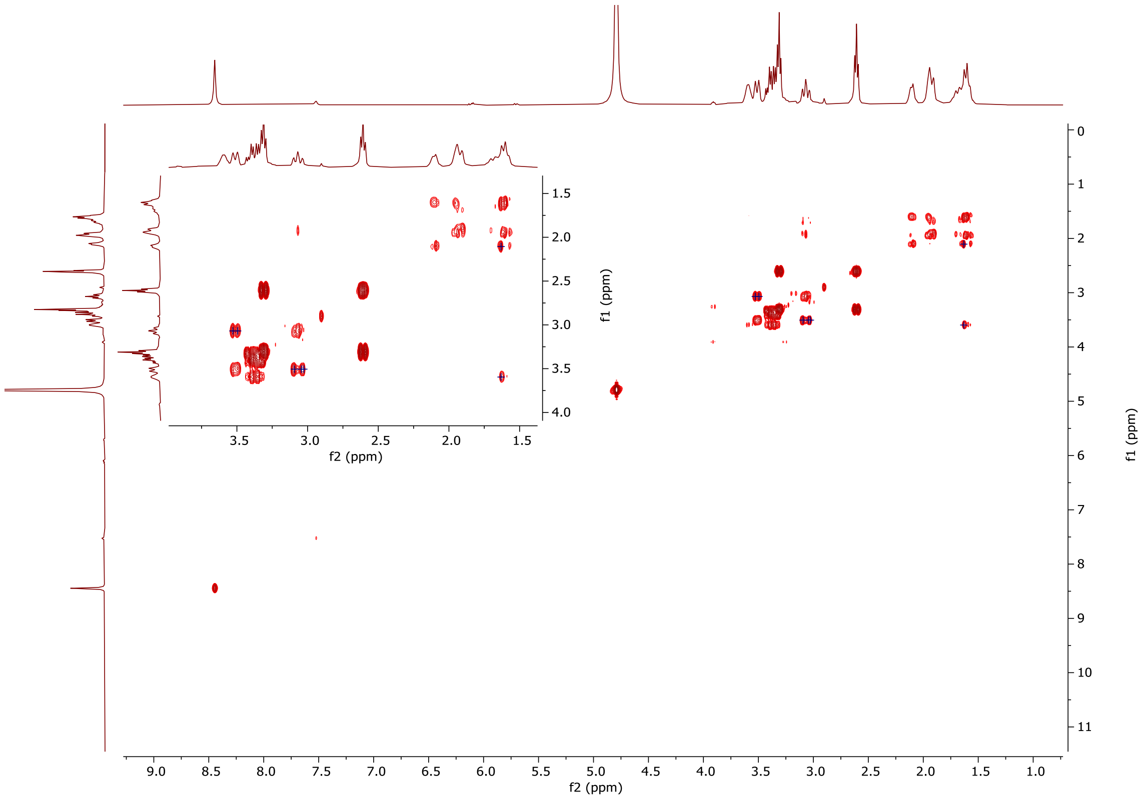

f1 (ppm)
f2 (ppm)

d)

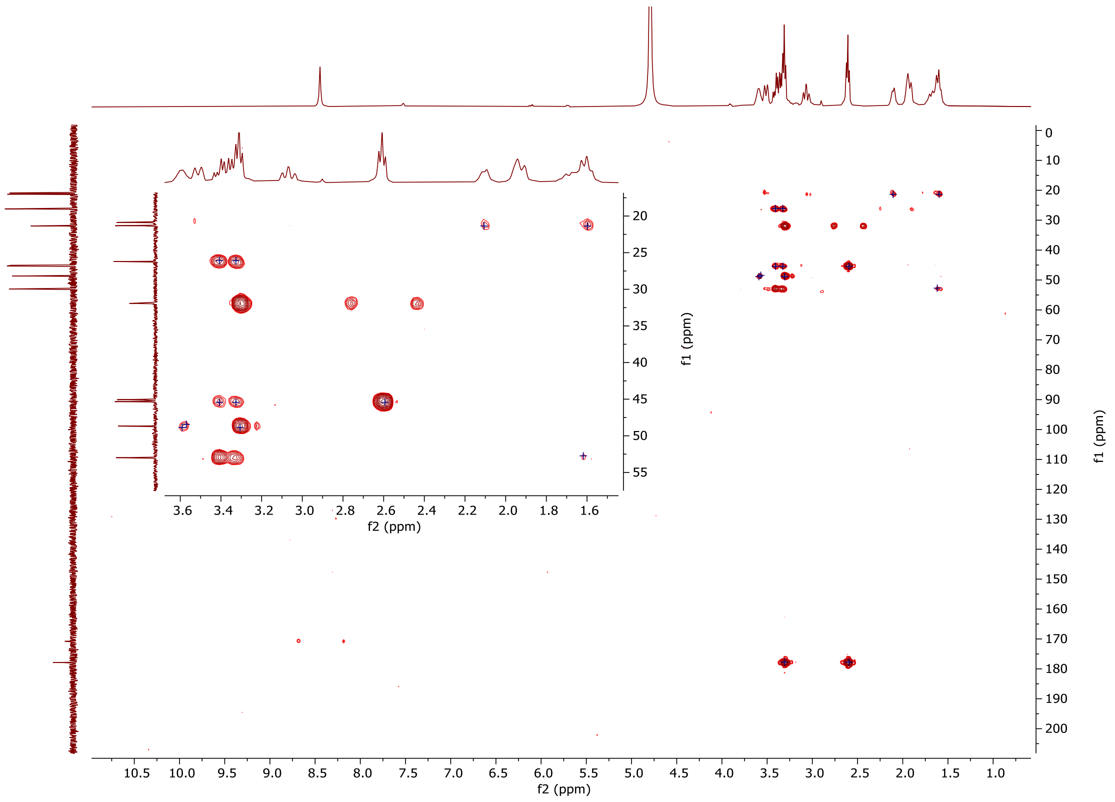

e)

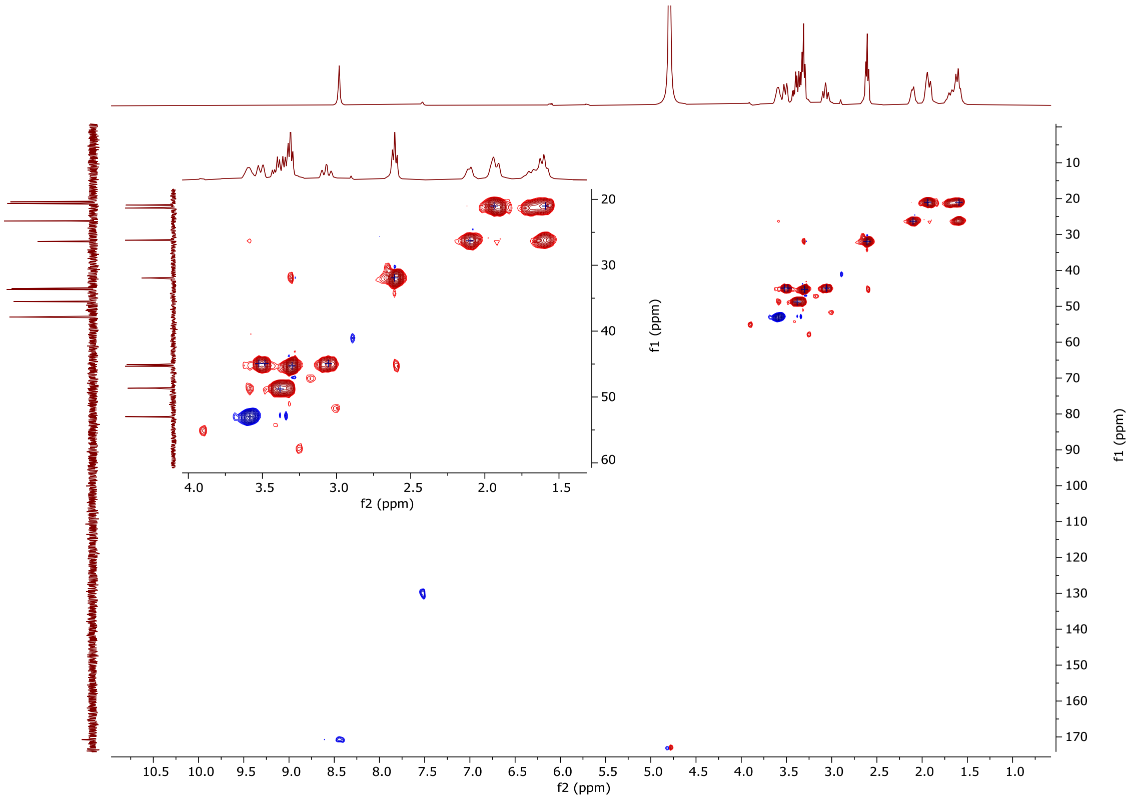

Figure S43. NMR spectra (D_2O) of (*S*)-**12**: a) ^{1}H, b) ^{13}C, c) COSY, d) HMBC and e) HSQC.

References

[1] N. J. Schnicker, S. M. De Silva, J. D. Todd, M. Dey, "Structural and Biochemical Insights into Dimethylsulfoniopropionate Cleavage by Cofactor-Bound DddK from the Prolific Marine Bacterium Pelagibacter" *Biochemistry* **2017**, *56*, 2873-2885.

[2] A. Qiaexpressionist, "A handbook for high-level expression and purification of 6xhis-tagged proteins" *Qiagen. p1-125* **2002**.

[3] M. T. Marty, A. J. Baldwin, E. G. Marklund, G. K. A. Hochberg, J. L. P. Benesch, C. V. Robinson, "Bayesian Deconvolution of Mass and Ion Mobility Spectra: From Binary Interactions to Polydisperse Ensembles" *Anal. Chem.* **2015**, *87*, 4370-4376.

[4] M. D. Palkowitz, B. Tan, H. Hu, K. Roth, R. A. Bauer, "Synthesis of Diverse N-Acryloyl Azetidines and Evaluation of Their Enhanced Thiol Reactivities" *Org. Lett.* **2017**, *19*, 2270-2273.

[5] S. Nazabadioko, R. J. Pérez, R. Brieva, V. Gotor, "Chemoenzymatic synthesis of (S)-2-cyanopiperidine, a key intermediate in the route to (S)-pipecolic acid and 2-substituted piperidine alkaloids" *Tetrahedron: Asymmetry* **1998**, *9*, 1597-1604.

[6] Schrödinger, *Release 2024-2*, Schrödinger, LLC, New York, NY, **2024**.

[7] Schrödinger, *Release 2024-2: Maestro, version 14.0*, Schrödinger, LLC, New York, NY, **2024**.

[8] G. M. Sastry, M. Adzhigirey, T. Day, R. Annabhimoju, W. Sherman, "Protein and ligand preparation: parameters, protocols, and influence on virtual screening enrichments" *J. Comput. Aided Mol. Des.* **2013**, *27*, 221-234.

[9] M. H. M. Olsson, C. R. Søndergard, M. Rostkowski, J. H. Jensen, "PROPKA3: Consistent Treatent of Internal and Surface Residues in Empirical pKa predictions." *J. Chem. Theor. Comput.* **2011**, *7*, 525-537.

[10] C. Lu, C. Wu, D. Ghoreishi, W. Chen, L. Wang, W. Damm, G. A. Ross, M. K. Dahlgren, E. Russell, C. D. Von Bargen, R. Abel, R. A. Friesner, E. D. Harder, "OPLS4: Improving Force Field Accuracy on Challenging Regimes of Chemical Space" *J Chem Theory Comput* **2021**.

[11] Schrödinger, *Release 2024-2: MacroModel, version 14.4*, Schrödinger, LLC, New York, NY, **2024**.

[12] W. C. Still, A. Tempczyk, R. C. Hawley, T. Hendrickson, "Semianalytical Treatment of Solvation for Molecular Mechanics and Dynamics" *J. Am. Chem. Soc.* **1990**, *112*, 6127-6129.

[13] T. A. Halgren, R. B. Murphy, R. A. Friesner, H. S. Beard, L. L. Frye, W. T. Pollard, J. L. Banks, "Glide: a new approach for rapid, accurate docking and scoring. 2. Enrichment factors in database screening" *J. Med. Chem.* **2004**, *47*, 1750-1759.

[14] R. A. Friesner, J. L. Banks, R. B. Murphy, T. A. Halgren, J. J. Klicic, D. T. Mainz, M. P. Repasky, E. H. Knoll, M. Shelley, J. K. Perry, D. E. Shaw, P. Francis, P. S. Shenkin, "Glide: a new approach for rapid, accurate docking and scoring. 1. Method and assessment of docking accuracy" *J. Med. Chem.* **2004**, *47*, 1739-1749.

[15] Schrödinger, *Release 2024-2: QSite*, Schrödinger, LLC, New York, NY, **2024**.

[16] D. M. Philipp, R. A. Friesner, "Mixed ab initio QM/MM modeling using frozen orbitals and tests with alanine dipeptide and tetrapeptide" *J. Comp. Chem.* **1999**, *20*, 1468-1494.

[17] R. B. Murphy, D. M. Philipp, R. A. Friesner, "A mixed quantum mechanics/molecular mechanics (QM/MM) method for large-scale modeling of chemistry in protein environments" *J. Comp. Chem.* **2000**, *21*, 1442-1457.

[18] Y. Wang, S.-L. Chen, "Reaction mechanism of the PuDddK dimethylsulfoniopropionate lyase and cofactor effects of various transition metal ions" *Dalton Trans.* **2022**, *51*, 14664-14672.

[19] Schrödinger, *Release 2025-2: Jaguar*, Schrödinger, LLC, New York, NY, **2025-2**.

[20] A. D. Bochevarov, E. Harder, T. F. Hughes, J. R. Greenwood, D. A. Braden, D. M. Philipp, D. Rinaldo, M. D. Halls, J. Zhang, R. A. Friesner, "Jaguar: A high-performance quantum chemistry software program with strengths in life and materials sciences" *Int. J. Quantum Chem.* **2013**, *113*, 2110-2142.

[21] Y. Cao, T. Balduf, M. D. Beachy, M. C. Bennett, A. D. Bochevarov, A. Chien, P. A. Dub, K. G. Dyall, J. W. Furness, M. D. Halls, T. F. Hughes, L. D. Jacobson, H. S. Kwak, D. S. Levine, D. T. Mainz, K. B. Moore, 3rd, M. Svensson, P. E. Videla, M. A. Watson, R. A. Friesner, "Quantum chemical package Jaguar: A survey of recent developments and unique features" *J. Chem. Phys.* **2024**, *161*.

[22] S. Grimme, "Accurate description of van der Waals complexes by density functional theory including empirical corrections" *J. Comput. Chem.* **2004**, *25*, 1463-1473.

[23] S. Grimme, "Semiempirical GGA-type density functional constructed with a long-range dispersion correction." *J Comput Chem.* **2006**, *27*, 1787-1799.

[24] S. Grimme, J. Antony, S. Ehrlich, H. Krieg, "A consistent and accurate ab initio parametrization of density functional dispersion correction (DFT-D) for the 94 elements H-Pu." *J. Chem. Phys.* **2010**, *132*, 154104-154119.
